# Toward a Deeper Understanding of Gut Microbiome in Depression: The Promise of Clinical Applicability

**DOI:** 10.1002/advs.202203707

**Published:** 2022-10-26

**Authors:** Lanxiang Liu, Haiyang Wang, Hanping Zhang, Xueyi Chen, Yangdong Zhang, Ji Wu, Libo Zhao, Dongfang Wang, Juncai Pu, Ping Ji, Peng Xie

**Affiliations:** ^1^ Department of Neurology Yongchuan Hospital of Chongqing Medical University Chongqing 402160 China; ^2^ NHC Key Laboratory of Diagnosis and Treatment on Brain Functional Diseases The First Affiliated Hospital of Chongqing Medical University Chongqing 400016 China; ^3^ Department of Neurology The First Affiliated Hospital of Chongqing Medical University Chongqing 400016 China; ^4^ College of Stomatology and Affiliated Stomatological Hospital of Chongqing Medical University Chongqing 401147 China

**Keywords:** depression, gut microbiome, microbial biomarkers, microbiota‐based therapeutics, precision medicine

## Abstract

The emergence of the coronavirus disease 2019 pandemic has dramatically increased the global prevalence of depression. Unfortunately, antidepressant drugs benefit only a small minority of patients. Thus, there is an urgent need to develop new interventions. Accumulating evidence supports a causal relationship between gut microbiota dysbiosis and depression. To advance microbiota‐based diagnostics and therapeutics of depression, a comprehensive overview of microbial alterations in depression is presented to identify effector microbial biomarkers. This procedure generated 215 bacterial taxa from humans and 312 from animal models. Compared to controls, depression shows significant differences in *β*‐diversity, but no changes in microbial richness and diversity. Additionally, species‐specific microbial changes are identified like increased *Eggerthella* in humans and decreased *Acetatifactor* in rodent models. Moreover, a disrupted microbiome balance and functional changes, characterized by an enrichment of pro‐inflammatory bacteria (e.g., *Desulfovibrio* and *Escherichia/Shigella*) and depletion of anti‐inflammatory butyrate‐producing bacteria (e.g., *Bifidobacterium* and *Faecalibacterium*) are consistently shared across species. Confounding effects of geographical region, depression type, and intestinal segments are also investigated. Ultimately, a total of 178 species and subspecies probiotics are identified to alleviate the depressive phenotypes. Current findings provide a foundation for developing microbiota‐based diagnostics and therapeutics and advancing microbiota‐oriented precision medicine for depression.

## Graphical abstract

Microbiota‐based therapeutics is a promising intervention approach and might be the next breakthrough in depression treatment. By integrating and analyzing the microbial alterations in depression reported worldwide, a depression‐associated microbial database is constructed. Identifying the species‐specific and trans‐species microbial changes and explicating the confounding effects is critical to developing microbiota‐based diagnostics and therapeutics and advancing microbiota‐oriented precision medicine for depression.

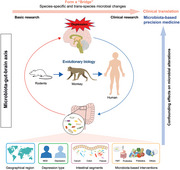

## 1 Introduction

Major depressive disorder (MDD) is a highly prevalent and seriously disabling mental illness that affects over 300 million individuals globally.^[^ ^1^ ^]^ The emergence of the coronavirus disease 2019 (COVID‐19) pandemic exacerbated the determinants of poor mental health and increased the global prevalence of MDD by 27.6%.^[^ ^2^ ^]^ During the COVID‐19 pandemic, MDD was one of the leading causes of global health‐related burdens. Clinically available antidepressants only benefit a small minority of MDD patients, attracting a cumulative remission rate of ≈50% after two treatment stages,^[^ ^3^ ^]^ and a relapse rate of 40–71% in responders during the naturalistic follow‐up period.^[^ ^3^, ^4^ ^]^ As such, novel interventions are needed for MDD.

The commensal microbiome, especially in the gut, is the largest and most complicated micro‐ecosystem in the human body. Imbalances in gut micro‐ecology can promote local and systemic pathology. Gut microbiota dysbiosis has been widely implicated in the pathogenesis of MDD.^[^ ^5^, ^6^, ^7^, ^8^, ^9^ ^]^ Fecal microbiota transplantation (FMT) from MDD patients into germ‐free mice and pseudo‐germ‐free rats induces depressive‐like behaviors in these animals,^[^ ^6^, ^10^ ^]^ strongly suggesting a causal role of gut microbiota dysbiosis in the development of depression; the specific causal microbial species are yet to be fully elucidated. In recent years, accumulating evidence has suggested that gut microbiota can trigger depression by regulating various signaling pathways implicated in the gut–brain axis, involving the host's metabolism, inflammatory responses, Ca2+/calmodulin‐dependent protien kinase II/cyclic AMP response element‐binding protein and mitogen‐activated protien kinase signaling, and the endocannabinoid system.^[^ ^5^, ^8^, ^11^, ^12^, ^13^ ^]^ Along with a deepening understanding of the gut microbiota–gut–brain axis,^[^ ^14^ ^]^ we have become aware that gut microbiota is likely important players in the diagnosis and therapy of depression through their involvement in the bidirectional communication system between the gastrointestinal tract and the brain. Modulation of gut microbes is thus likely a rational and effective etiology‐targeted and mechanism‐oriented therapeutic approach.^[^ ^15^ ^]^ The recent U.S. Food and Drug Administration approval of two live biotherapeutics *Parabacteroides distasonis* MRx0005 and *Megasphaera massiliensis* MRx0029 for the treatment of Parkinson's disease extended microbe‐based therapeutics into the field of neuropsychiatry.^[^ ^16^, ^17^ ^]^ Microbiota‐based therapeutics targeting the gut–brain axis might be the next breakthrough in depression treatment.

Identifying the effector microbial biomarkers in depression is the first step toward developing microbiota‐based therapeutics. Attempts to characterize the composition of commensal microbiota in depressed patients and relevant animal models have yielded plentiful but contradictory results.^[^ ^8^, ^9^, ^18^, ^19^ ^]^ An integrated analysis of large‐scale data would be conducive to identifying highly specific microbial biomarkers for depression. Multiple reviews have undertaken such efforts,^[^ ^20^, ^21^, ^22^, ^23^ ^]^ but they only included studies with depressed patients and neglected to incorporate data from animal models. Such animal studies are complementary and indispensable to deciphering the underlying mechanisms of gut microbiota regulation of depression and to help the screening process for microbial species that might alleviate depression phenotypes.^[^ ^24^ ^]^ Comparing the characteristic microbial biomarker differences between animal models and depressed patients is a crucial component of translational research efforts.

In this study, to construct a depression‐associated microbial database, provide a reference for future research, and identify relevant microbial biomarkers that have promise for clinical applications, we re‐analyzed relevant published articles and present a comprehensive overview of the commensal microbiome changes involved in the pathogenesis of depression. In particular, we evaluate species‐specific microbial alterations in depressed patients, rodents, and nonhuman primate models of depression, trans‐species changes across species, and emphasize their utility as targets for microbiota‐based diagnostics and therapeutics. Moreover, we explore the effects of various confounders on gut microbial alterations to help advance microbiota‐based precision medicine. In addition, we summarize recent advances in microbiota‐based interventions, such as FMT, probiotics, and prebiotics, and their potential utility in the treatment of depression.

## 2 Results

### 2.1 Study Selection Results

Among the 20 349 records yielded by our database search, 11 206 remained after the removal of duplicates. Based on our eligibility criteria, 317 articles were selected. Of these, 76 articles were excluded after a full‐text screening, resulting in the inclusion of 241 articles. In the second search, a further 28 articles were identified, combined with an additional 3 references from the included articles, resulting in 272 articles that were included in the final analyses: 66 involving depressed patients, 199 involving rat/mouse depression models, 4 with depressed patients and rodent models, and 3 utilizing nonhuman primate depression models (see Figure S1, Supporting Information).

### 2.2 Characteristics of the Included Studies

Of the 70 eligible articles investigating commensal microbial changes in individuals with MDD or depression, 35 (50%) were conducted in China, 11 in America (15.7%), and the remaining 24 in other countries including the United Kingdom, Europe, Japan, Norway, Australia, France, Italy, New Zealand, Ireland, Poland, Mexico, Korea, Belgium, and the Netherlands. Four studies included subgroups including MDD cases in the young (aged 18–29 years) and middle‐aged (30–59 years),^[^ ^25^ ^]^ females and males,^[^ ^26^ ^]^ the use of 16S or metagenomics,^[^ ^27^ ^]^ and 16S or PCR.^[^ ^28^ ^]^ Therefore, 54 case‐control studies generated 58 comparisons involving 2346 patients and 3926 controls; the study case sample size range was 7–122. Sixteen studies investigated the association between microbiota and the severity of depression symptoms. MDD cases were mainly diagnosed using the DSM‐IV, DSM‐V, and/or ICD‐10 criteria; the Hamilton Depression Scale and the Beck Depression Inventory were the most commonly used measures of symptom severity. Comorbidities and psychiatric medications varied substantially across studies but were not the focus subjects of the original studies. Feces formed the vast majority of biological samples (86.7%); five studies used other samples such as saliva (2.9%), sinonasal swab and mucus samples (1.4%), plasma (1.4%), and serum (1.4%). The methods of microbiome estimation varied widely; 16S was the most common method (78.6%), followed by metagenomics, quantitative polymerase chain reaction (qPCR), real‐time qPCR (RT‐qPCR), and metaproteomics. Further details are provided in Table S1, Supporting Information.

Of the 206 eligible articles investigating commensal microbial changes in animal models of depression, 136 used mice (e.g., C57BL/6, BALB/c, Kunming, CD‐1, and ICR), 67 used rats (e.g., Sprague–Dawley, Wistar, and Flinders sensitive line), 3 used *Macaca fascicularis* macaques, and 1 used Syrian hamsters. Most studies were conducted with adult male rodents. 134 articles focused on stress‐induced depressive‐like behaviors, such as chronic unpredictable mild stress, chronic social defeat stress, and chronic restraint stress; 41 focused on drug‐ and diet‐induced depression, such as antibiotics, dextran sulfate sodium, lipopolysaccharide, and high‐fat diet; and 21 focused on microbiota‐related depression, for example, transplanting fecal microbiota from patients with MDD or animals with depressive‐like behaviors, and *Escherichia coli* K1‐induced depression. The most frequently used indices of depressive‐like behaviors were anhedonia via sucrose preference in the sucrose preference test and despair via immobility time in the forced swimming test and the tail suspension test. The selection of biological samples also varied substantially: 144 of the 206 studies were conducted using fecal samples, 39 with cecum contents, 20 with colonic contents, 4 with small intestinal contents, and 3 with rectal contents. More than 90% of the included studies used 16S. Additional details are provided in Table S2, Supporting Information.

### 2.3 Bacterial *α*‐Diversity

**Figure 1:**
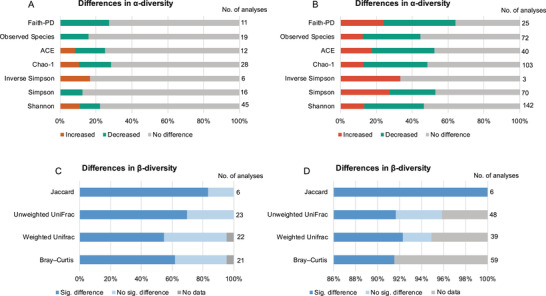
Differences in commensal microbial *α*‐ and *β*‐diversity in depression compared to controls across studies of patients and animal models. A) Differences in *α*‐diversity metrics between patients with depression and controls. B) Differences in *α*‐diversity metrics between animal models with depressive‐like behavior and controls. C) Differences in *β*‐diversity algorithms between patients with depression and controls. D) Differences in *β*‐diversity algorithms between animal models with depressive‐like behavior and controls.

For *α*‐diversity analysis, the most frequently reported metrics were Chao and Ace for richness, Shannon, Simpson, and invsimpson for diversity, and other indices such as observed species and phylogenetic diversity. In clinical studies, 49 studies reported *α*‐diversity, which generated 137 *α*‐diversity analyses (**Figure** **1**A and Figure S2, Supporting Information), while in preclinical studies, 155 studies reported *α*‐diversity and generated 455 *α*‐diversity analyses (Figure 1B and Figure S3, Supporting Information). The Shannon index was the most widely reported diversity metric in clinical and preclinical studies, and Chao was the most commonly examined richness metric. Over half of the analyses found no difference in *α*‐diversity between depression and control groups: 78.1% of the clinical and 51.0% of the preclinical studies. For richness, more analyses reported decreased (7/40) than increased (4/40) *α*‐diversity in depressed patients; 35.7% (51/143) showed lower and only 14.0% (20/143) showed higher *α*‐diversity in animal models of depression. For diversity, 30.2% of the analyses found a decrease, and only 18.1% found an increase in *α*‐diversity in animal models of depression; however, this difference was not observed in patients with depression. Thus, based on our synthesized data, we found no strong evidence for a difference in the *α*‐diversity of microbiota between depression and control groups.

### 2.4 Bacterial *β*‐Diversity

For *β*‐diversity analysis, the most frequently used algorithms in the included studies are Bray–Curtis, weighted and unweighted UniFrac, and Jaccard similarity. 72 *β*‐diversity analyses between patients and controls were reported in 42 studies (Figure 1C and Table S3, Supporting Information) and 152 analyses between animals with depression and control groups in 128 studies (Figure 1D and Table S4, Supporting Information). Two human and 9 animal analyses presented only the principal coordinates analysis (PCoA) without any statistical testing report. For statistical differences, ≈63.9% of the *β*‐diversity analyses found significant differences in the composition of commensal microbiota in patients with MDD/depression versus controls, as indicated by group clustering on visual ordination plots (e.g., PCoA, principal component analysis (PCA)). Differences were also reported in 92.1% of *β*‐diversity analyses between animals with depressive‐like behavior and controls. These data suggest considerable changes in the composition of the commensal microbiota in depressive conditions compared with controls.

### 2.5 Microbial Taxonomy

Bacteria with different relative abundances in depression versus control groups were reported at the phylum, class, order, family, genus, and species levels. To avoid the risk of false positives, we summarized microbial findings only when concordantly reported by ≥2 studies. Overall, 215 taxa were found to be differentially abundant in patients with MDD or depression, spanning 7 phyla, 5 classes, 12 orders, 37 families, 85 genera, and 69 species (Tables S5–S7, Supporting Information); in animal models of depression, 312 differentially abundant taxa were identified, spanning 9 phyla, 17 classes, 26 orders, 70 families, 148 genera, and 42 species (Tables S8–S10, Supporting Information), suggesting that a disrupted microbiome balance, rather than an altered single microorganism, is related to the pathogenesis of depression. The oral cavity and gut are the two largest microbial habitats,^[^ ^29^, ^30^ ^]^ due to limited published data on the oral microbiome, we focused on the gut and identified substantial divergences in the dominant taxa between patients and animal models.

#### 2.5.1 Species‐Specific Microbial Changes

**Figure 2:**
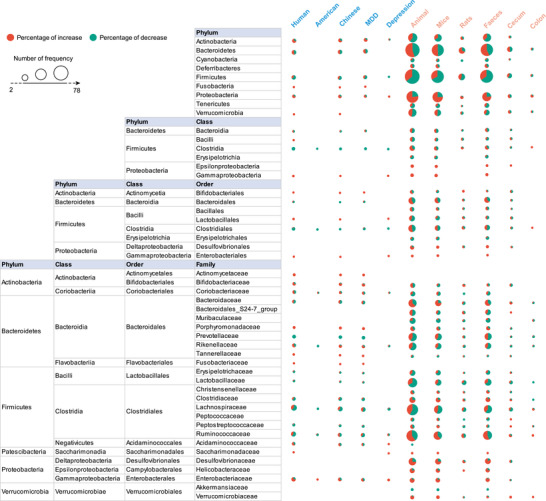
Differentially abundant taxa at the phylum, class, order, and family level in different subgroups of patients and animal models of depression. Differentially abundant taxa reported by ≥2 studies at each taxonomic level were collated in this study, and the main taxa are presented here. The size of the circle indicates the total number of studies that reported taxa, red indicates the percentage of studies reporting taxa with an increased abundance in depression, and green indicates the percentage of studies reporting taxa with a decreased abundance in depression. MDD, major depressive disorder.

Species specificity was observed for the consistent enrichment of phyla Verrucomicrobia in depressed patients, which tended to be depleted in animal models; phyla Cyanobacteria, Deferribacteres, and Tenericutes were only found in animals, especially in mice (**Figure** **2**). At a class level, consistently lower levels of Clostridia were observed in patients, but these tended to be higher in animals. Higher levels of Epsilonproteobacteria and a tendency for lower levels of Erysipelotrichia were found in mice, these patterns were neither reported in depressed patients nor rats. Regarding the order level, we found a significant depletion tendency for Clostridiales and Bacteroidales in patients and an enrichment tendency in animals, particularly in mice. Bacillales, Erysipelotrichales, and Desulfovibrionales were identified in animals but not in patients. Additionally, there was an increased tendency of family Tannerellaceae in MDD patients, while Christensenellaceae, Desulfovibrionaceae, and Helicobacteraceae were higher in rodents; consistently, increased Verrucomicrobiaceae and reduced Akkermansiaceae were observed in mice; an enrichment tendency for Rikenellaceae was reported in patients and a depletion tendency in animals. A depletion tendency for Ruminococcaceae was reported in patients and an enrichment tendency in animals.

**Figure 3:**
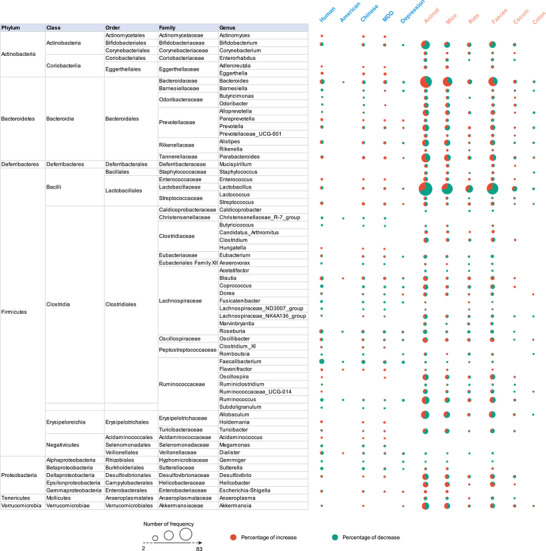
Differentially abundant taxa at the genus level in different subgroups of patients and animal models of depression. Differentially abundant taxa reported by ≥2 studies at the genus level were collated in this study, and the main taxa are presented here. The size of the circle indicates the total number of studies that reported taxa, red indicates the percentage of studies reporting taxa with an increased abundance in depression, and green indicates the percentage of studies reporting taxa with a decreased abundance in depression. MDD, major depressive disorder.

Consistently, at the genus level (**Figure** **3**), depressed patients showed increased abundances of *Eggerthella*, *Paraprevotella*, *Flavonifractor*, and *Holdemania*, and decreased abundances of *Christensenellaceae_R‐7_group*, *Coprococcus*, *Fusicatenibacter*, and *Lachnospiraceae_ND3007_group*. Interestingly, a decreased tendency was observed for *Paraprevotella* in mouse models of depression; alterations in *Eggerthella*, *Holdemania*, *Christensenellaceae_R‐7_group*, and *Lachnospiraceae_ND3007_group* were not identified in animal models. A higher tendency for *Helicobacter* and *Candidatus_Arthromitus* was only identified in animals but not in patients. Additionally, the abundance of *Acetatifactor* was consistently decreased in mouse and rat models but not in patients, and *Caldicoprobacter* and *Roseburia* were consistently decreased only in rats.

**Figure 4:**
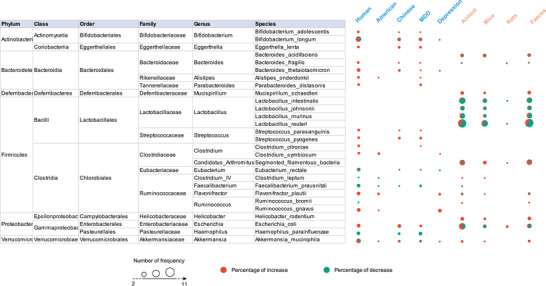
Differentially abundant taxa at the species level in different subgroups of patients and animal models of depression. Differentially abundant taxa reported by ≥2 studies at the species level were collated in this study, and the main taxa are presented here. The size of the circle indicates the total number of studies that reported taxa, red indicates the percentage of studies reporting taxa with an increased abundance in depression, and green indicates the percentage of studies reporting taxa with a decreased abundance in depression. MDD, major depressive disorder.

At a species level (**Figure** **4**), the abundances of many pathogenic bacteria, such as *Bacteroides_fragilis*, *Eggerthella_lenta*, and *Ruminococcus_gnavus* were enriched only in patients with depression, while *Mucispirillum_schaedleri* and *Helicobacter_rodentium* were enriched in depressed mice. Beneficial bacteria, such as *Faecalibacterium_prausnitzii*, were decreased in patients. Paradoxically, pathogenic bacteria, such as *Haemophilus_parainfluenzae*, were decreased in patients and beneficial bacteria, such as *Bifidobacterium_adolescentis*, *Bacteroides_thetaiotaomicron*, and *Parabacteroides_distasonis*, were increased in patients; this finding blurs the clear boundary between beneficial and pathogenic bacteria and suggests that a balance is likely more important than any single bacterium. Additionally, some species belonging to *Lactobacillus*, including *Lactobacillus_intestinalis*, *Lactobacillus_johnsonii*, *Lactobacillus_murinus*, and *Lactobacillus_reuteri*, showed a reduced tendency in animal models, especially in mice. Due to the limited studies on nonhuman primates, only one microbial species (*Helicobacter_macacae*) was reported to be decreased.

#### 2.5.2 Trans‐Species Microbial Alterations

Our present findings indicated an overlap between species. In considering the trans‐species microbial alterations, the most consistent findings involved the enrichment of class Gammaproteobacteria, order Enterobacteriales, and family Saccharimonadaceae, the depletion of genus *Gemmiger* in depressed patients and mice, and the enrichment of *Escherichia_coli* in patients and rats. There was also evidence for an increase in the family Porphyromonadaceae and genus *Flavonifractor* in patients and an increased tendency in rodents. A consistent reduction in Prevotellaceae was identified in patients and a reduced tendency was also seen in mice. Furthermore, increased tendencies for phyla Proteobacteria, family Enterobacteriaceae, genera *Desulfovibrio*, and *Escherichia/Shigella* were observed in patients and animal models, and reduced tendencies for phyla Firmicutes, family Lactobacillaceae, Lachnospiraceae, genera *Bifidobacterium*, *Barnesiella*, *Faecalibacterium*, and *Dialister* across all studied species. At the genus level, *Bacteroides*, *Prevotella*, *Lactobacillus*, and *Ruminococcus* genera were the dominant taxa, those were frequently reported but inconsistent between species.

#### 2.5.3 Effect of Confounders on Microbial Alterations

A cross‐country analysis identified differential dietary patterns,^[^ ^31^ ^]^ which formed the main factor affecting the gut microbiome.^[^ ^32^ ^]^ Thus, in depressed patients, we first analyzed whether cases originating from different countries harbored discrepant microbial alterations. Since geographic subgroups were heavily imbalanced (most studies were conducted in America and China and fewer were from the United Kingdom, Europe, and Japan), we clustered microbial alterations in these two countries. Clustering analysis identified several discrepancies in the gut microbiota composition between American and Chinese patients: increased *Eggerthella* and decreased Prevotellaceae, *Coprococcus*, and *Fusicatenibacter* in individuals from China. These discrepancies were driven entirely by studies from China. These findings strongly suggest that additional studies are required from geographically diverse nations to properly build microbiome databases that are matched to specific patient characteristics.

To explore the association between depression type and microbial changes in patients, we analyzed discrepant microbial changes between clinical MDD and subthreshold depression. Several discrepancies were identified: there were higher levels of genera *Eggerthella*, *Flavonifractor*, and *Holdemania* in MDD, lower levels of family Prevotellaceae, and genus *Christensenellaceae_R‐7_group* in MDD; higher levels of order Enterobacteriales in depression, and lower levels of genus *Gemmiger* in depression. Due to the imbalance of the included studies (most focused on MDD), these conclusions require further validation.

It has been shown that different gastrointestinal segments exhibit different microbial profiles.^[^ ^33^ ^]^ To investigate this, we compared the microbial changes in the feces, cecum, and colon in depressive conditions. We found that increases in the class Gammaproteobacteria and order Enterobacteriales and decreases in the genera *Caldicoprobacter*, *Acetatifactor*, and *Dialister* were reported in feces, which were not reported in the cecum and colon. The genera *Ruminiclostridium* and *Alloprevotella* showed an increased tendency in feces and a decrease in the cecum and colon, respectively. Furthermore, there was a decreased tendency for the family Lachnospiraceae in the feces and cecum and an increased tendency in the colon. Bacteria in the feces were classed at the species level but this was not the case in the cecum and colon, this may stem from the limited number of studies that have assessed the cecum and colon.

#### 2.5.4 Identification of Microbial Biomarkers in Depression

**Figure 5:**
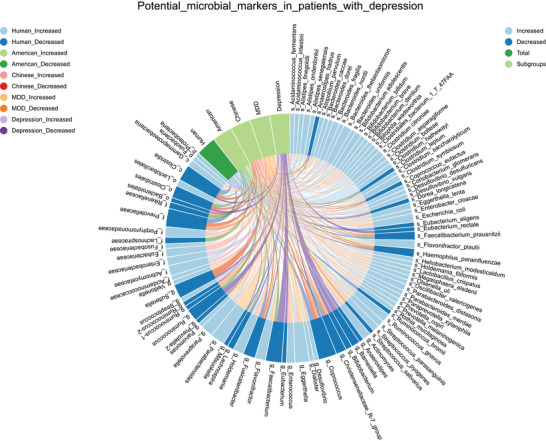
Potential microbial biomarkers in different subgroups of patients with depression. Microbial biomarkers reported by ≥2 studies with consistent changes in each subgroup of patients with depression are collated here. Marks represent the bacterial taxa, and lines represent the changes of taxa in subgroups of patients with depression. MDD, major depressive disorder.

**Figure 6:**
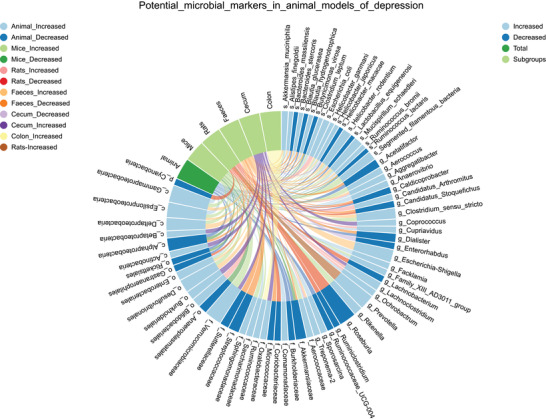
Potential microbial biomarkers in different subgroups of animal models of depression. Microbial biomarkers reported by ≥3 studies with consistent changes in each subgroup of animal models of depression are collated here. Marks represent the bacterial taxa, and lines represent the changes of taxa in subgroups of patients with depression.

A comprehensive understanding of characteristic gut microbiome changes in depression is required to develop microbiota‐oriented precision medicine for this disorder. To identify potential microbiota‐based therapeutic targets, we first analyzed microbial biomarkers that were reported by ≥2 studies and which were shown to display consistent changes in each subgroup of patients with depression. This process found 91 potential taxa, including 2 phyla, 2 classes, 3 orders, 8 families, 21 genera, and 55 species (**Figure** **5**). These consistent taxonomic changes highlight the characteristic microbial biomarkers in the diagnosis of depression and the development of microbiota‐based therapeutics. For example, microbial signatures of patients with depression were characterized by lower levels of *Faecalibacterium_prausnitzii*, a major butyrate‐producing bacteria in the gut, its administration was shown to prevent chronic stress‐induced depressive‐like behaviors in animals.^[^ ^34^ ^]^ Due to the inclusion of a larger number of animal studies, we included taxa that were consistently reported by ≥3 studies in each subgroup, and 69 microbial biomarkers were identified (**Figure** **6**). For instance, higher levels of *Segmented_filamentous_bacteria* (SFB) in mice were shown to promote T helper 17 (Th17) cell production, which was required to induce depressive‐like behaviors.^[^ ^35^ ^]^ This suggests that SFB is a novel target for microbiota‐based therapeutics for depression. These animal model findings suggest microbial markers that may prove to be clinically useful.

### 2.6 The Development of Microbiota‐Targeted Interventions for Depression

**Figure 7:**
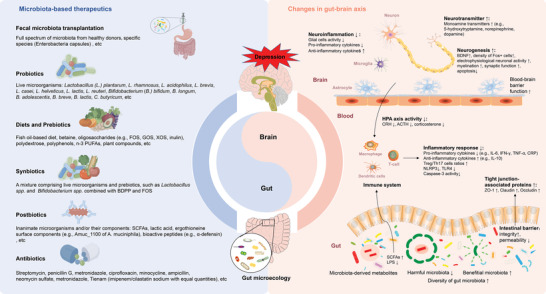
The microbiota‐targeted interventions for depression and the related changes in the gut–brain axis. FOS, fructo‐oligosaccharides; GOS, galacto‐oligosaccharides; XOS, xylo‐oligosaccharide; PUFAs, polyunsaturated fatty acids; BDPP, bioactive dietary polyphenol preparation; SCFAs, short chain fatty acids; BDNF, brain‐derived neurotrophic factor; HPA, hypothalamic‐pituitary‐adrenal; CRH, corticotrophin releasing hormone, ACTH, adrenocorticotropic hormone; IL‐6, interleukin‐6; IL‐10, interleukin‐10; IFN‐*γ*, interferon‐*γ*; TNF‐*α*, tumor necrosis factor‐*α*; CRP, C‐reactive protein; NLRP3, NOD‐like receptor thermal protein domain associated protein 3; TLR4, toll‐like receptor 4; LPS, lipopolysaccharide.

Homeostasis in the brain–gut axis is essential to maintaining mental health.^[^ ^36^, ^37^ ^]^ Modulation of the gut microbiome by microbiota‐based interventions might offer a novel therapeutic strategy for the treatment of depression. Here, we summarize the efficacy of various microbiota‐based interventions in alleviating depression symptoms, based on data from 210 eligible studies (Figure S4, Supporting Information); 45 studies involving patients (Table S11, Supporting Information), and 165 involving animal models (Table S12, Supporting Information). The most frequently used interventions include transplantation of the gut microbiome from healthy donors or inoculation with specific bacteria, probiotics, prebiotics, synbiotics, postbiotics, and antibiotics; of these, probiotics were the most frequently documented (**Figure** **7**). A total of 178 species and subspecies probiotics were identified as having the ability to attenuate the depressive phenotype (Table S13, Supporting Information); *Lactobacillus spp*. and *Bifidobacterium spp*. were the most studied. *Lactobacillus_acidophilus*, *Bifidobacterium_bifidum*, and *Bifidobacterium_longum* were the top three frequently used in patients with depression, while *Lactobacillus_plantarum*, *Lactobacillus_rhamnosus*, *Bifidobacterium_longum*, *Lactobacillus_helveticus*, and *Lactococcus_lactis* were the top five frequently used in animal models. Moreover, the prebiotics–substrates that are selectively utilized by host microorganisms to confer health benefits^[^ ^38^ ^]^—reduce depressive symptoms by promoting the growth of probiotics. Interestingly, antibiotics can exert antidepressant effects by regulating the gut microbiota. Whether antibiotics show pro‐ or anti‐depressive effects mainly depends on their pharmacological action and which combination is utilized. These microbiota‐based interventions alleviated the depressive phenotypes by regulating the gut–brain axis, this mainly involved inhibition of the inflammatory response, promotion of neurogenesis, regulation of hypothalamic‐pituitary‐adrenal axis activity, neurotransmitter release, intestinal microenvironment, and barrier function (Figure 7).

## 3 Discussion

This is the first comprehensive review, to date, to investigate commensal microbiota changes in patients with depression and animal models. Our main aim was to draw a microbial map of depression and identify effector microbial biomarkers to advance microbiota‐based diagnostics and therapeutics in depression. There is an assumption that higher microbial diversity is more beneficial to health,^[^ ^39^ ^]^ but unexpectedly, we found no strong evidence for a difference in *α*‐diversity in patients with depression and animal models. However, clinical studies have found a negative association between microbial *α*‐diversity and depression severity;^[^ ^40^ ^]^ *α*‐diversity is an important indicator of treatment response as non‐responders show lower diversity than responders.^[^ ^41^ ^]^ For *β*‐diversity, patients with MDD or depression and animal models consistently clustered separately from their corresponding controls. However, whether the within‐species subgroups were uniquely clustered is not yet clear as no study has assessed the differences in *β*‐diversity between subgroups.

A previous study reported on species‐specific differences in the gut microbiota.^[^ ^42^ ^]^ A better understanding of species‐specific microbial changes in depression will improve our translational research efforts. Due to ethical and practical complexities, high heterogeneity, and the dynamics of gut microbiota in humans, animal models are frequently used to investigate host–microbiome interactions. Although the translation of these findings to humans remains a substantial challenge due to the species specificity of the microbiome, there is still no better alternative.^[^ ^43^ ^]^ Unsurprisingly, we found evidence of species specificity in gut microbiota in the present study. Specifically, the genus *Eggerthella* and species *Eggerthella_lenta* were enriched in patients with MDD, these bacteria induced intestinal inflammation by activating Th17 cells,^[^ ^44^ ^]^ suggesting that a Th17/Treg cell imbalance mediates the gut microbiota dysbiosis relationship with depression. Interestingly, beyond depression, *Eggerthella* was also found to be enriched in patients with other psychiatric disorders, such as bipolar disorder, schizophrenia, and psychosis,^[^ ^21^ ^]^ indicating a greater species effect than diagnostic effect. The lithocholic acid (LCA) producing bacteria *Acetatifactor* was specifically depleted in rodent models. Strikingly, the administration of LCA to mice regulates host immune responses, by reducing Th17 and increasing Treg cell differentiation.^[^ ^45^, ^46^ ^]^ These findings suggest that the same pathological alterations may be driven by different effector bacteria in different species, which complicates our ability to synthesize translational data.

However, we identified several trans‐species microbial alterations, which form a “bridge” between animal and human research. We observed that the beneficial genus *Gemmiger* was consistently lower in patients and animal models of depression. *Gemmiger spp*. are included in some probiotics that aim to inhibit inflammation.^[^ ^47^ ^]^ In addition, our findings also indicated an overlap between species in inconsistently changed taxa, including the increased tendency for the pathogenic bacteria genera (e.g., *Desulfovibrio* and *Escherichia/Shigella*) and a decreased tendency for the beneficial bacteria genera (e.g., *Bifidobacterium* and *Faecalibacterium*). *Escherichia/Shigella* are gram‐negative pro‐inflammatory pathogens that shed lipopolysaccharides (LPS) that can induce acute intestinal injury, increase blood–brain barrier permeability, and activate neuroinflammation.^[^ ^48^, ^49^ ^]^ *Bifidobacterium* and *Faecalibacterium* strains have anti‐inflammatory properties^[^ ^50^, ^51^ ^]^ that are likely mediated by short‐chain fatty acids, especially butyrate.^[^ ^52^, ^53^ ^]^ This occurs mainly through regulating intestinal epithelial cells to decrease pro‐inflammatory cytokines and increase anti‐inflammatory factors.^[^ ^54^, ^55^ ^]^ These bacteria are also associated with depression severity,^[^ ^18^, ^56^ ^]^ suggesting that the enrichment of pro‐inflammatory bacteria and the depletion of anti‐inflammatory bacteria may form a characteristic microbial biomarker of the depressive state, irrespective of species. These main findings are similar to several other human conditions which are linked to systemic and gut inflammation, and further confirm the inflammatory hypothesis of depression. Depression‐related gut microbial dysbiosis causes changes in the inflammatory markers,^[^ ^57^ ^]^ and conversely, the activation of pro‐inflammatory responses can indirectly induce bacterial translocation that can participate in the pathophysiology of depression.^[^ ^58^, ^59^ ^]^ In brief, gut‐derived systemic inflammation is a driver of depression. Additionally, compared with the inconsistent microbial alterations, these more consistent alterations were more promising for the development of microbiota‐based therapeutics for MDD.

Strains from *Lactobacillus* and *Bifidobacterium* were the most frequently used probiotic supplements to relieve the depressive phenotype. Interestingly, consistent with the previous meta‐analysis,^[^ ^21^ ^]^ we observed an enrichment tendency for the genus *Lactobacillus* in patients with MDD, suggesting a differential effect for different species from this genus. The increase in some beneficial bacteria in patients with MDD indicates that there is no clear boundary between beneficial and pathogenic bacteria in this disorder, a focus on balance appears to be more critical than the regulation of bacteria from a single category. It is, however, important to note that an increased abundance of *Lactobacillus* is associated with antidepressant use.^[^ ^60^, ^61^ ^]^ Thus, we speculate that antidepressants may impart physiological changes in the microbiome before they exert an antidepressant effect. However, two studies have reported that ingestion of *L) intestinalis*, *L. reuteri*, and *Lactobacillus helveticus* can cause depressive‐ and anhedonia‐like phenotypes^[^ ^62^ ^]^ and disrupt social behaviors.^[^ ^63^ ^]^ These findings suggest that probiotics must be administered judiciously to impart antidepressive effects, improper administration may prove to be harmful to health. Besides the strains from *Lactobacillus* and *Bifidobacterium*, species from other genera, for example, *Lactococcus*, *Streptococcus*, *Bacillus*, *Akkermansia*, and *Faecalibacterium*, also can attenuate the depressive phenotype. However, these findings require further clinical validation. In general, probiotics improve human health by modulating the composition and function of gut microbiota.^[^ ^64^, ^65^ ^]^ Furthermore, although FMT is a safe and effective treatment strategy for patients who do not respond to standard treatment,^[^ ^66^ ^]^ a similarly effective but less invasive and more standardized pill containing bacterial spores isolated from the feces of healthy donors is greatly desirable.^[^ ^67^ ^]^ Overall, in addition to the quantitative changes, the prevalent microbial genomic structural variants in the gut microbiome are also associated with host health.^[^ ^68^ ^]^ In the future, the quantitative and structural changes of gut microbiota should be comprehensively considered in developing microbiota‐based therapeutics.

Among the numerous confounders that can contribute to the inconsistencies in microbial composition between studies, based on available data, we were able to analyze two patient factors: country and depression type. Patients from different countries have different genetics and dietary patterns,^[^ ^31^ ^]^ which significantly affect the gut microbial composition.^[^ ^32^ ^]^ Some microbial changes were specific to China, for example, increased *Eggerthella* and *Acidaminococcus* and decreased *Coprococcus* and *Fusicatenibacter*. These microbial discrepancies across countries highlight a need for each country or geographic region to develop a unique microbiome database to guide future microbial research.^[^ ^69^ ^]^ Furthermore, although the division of patients into clinical MDD and subthreshold depressive symptoms is rather crude,^[^ ^70^ ^]^ analyzing the association between depression severity and gut microbiota proved impossible based on currently available data. Some alterations were found to be specific to MDD such as increased *Flavonifractor* and *Holdemania*. Others were influenced by a combination of geographical region and depression type. In addition, while we found a higher diversity of gut microbiota in feces compared with cecum and colon, we cannot exclude that this finding was biased by the skewed distribution of studies that mostly analyzed feces. We hope that future studies will delineate the exact effects of various confounders on depression‐associated microbial changes, especially on the alterations identified in this review. Beyond these specific alterations, stable species that show consistent changes between individuals can serve as characteristic microbial biomarkers of depression. Exploration of the individual specificity and genetic stability of the gut microbiome will help to understand the causal relationship between the gut microbiome and disease and provides a direct basis for the development of personalized medicine.^[^ ^71^, ^72^, ^73^ ^]^

In this comprehensive study, only two studies were found to have investigated the relationship between the gut microbiome and depression in pediatric patients,^[^ ^74^, ^75^ ^]^ and only one assessed the oral microbiome.^[^ ^76^ ^]^ These limited data precluded us from exploring the discrepancies in microbiota composition between pediatric and adult depressed patients. Further, as most studies included a mix of un‐medicated and medicated patients, we were unable to subgroup the microbial findings on this basis. As such, we did not attempt to explore the potential confounding effects of medication on gut microbial composition. Oral samples are particularly attractive given their clinical accessibility. However, more work needs to be conducted on the relationship between the oral and gut microbiomes to clarify the role of the oral–gut axis in depression.^[^ ^30^, ^77^ ^]^ Additionally, we did not discuss the differences in methodologies and reference database usage that may have caused inconsistencies in taxonomic findings between studies, as such efforts have been made by others.^[^ ^78^, ^79^, ^80^, ^81^ ^]^ In addition to the qualitative results for microorganisms, such as significance values reported by most studies, the quantitation of abundance is essential to enable meta‐analysis and the calculation of relevant biological effect sizes of confounders in microbial changes, and that of microbial markers in disease.^[^ ^82^ ^]^ To achieve this, we encourage future studies to implement strategies for data sharing with sufficient metadata to permit additional analyses to be performed. Finally, compared with rodents, non‐human primates offer a model that is genetically and physiologically proximal to humans; likely, they will better mimic the human depressive host–microbiome interaction.^[^ ^83^ ^]^ Unfortunately, we found only three studies that focused on gut microbiota changes in depressed non‐human primates, reducing the reliability and reproducibility of the findings in these animal species.

## 4 Conclusion

The present study provides a comprehensive overview of commensal microbial changes in depression and suggests key species‐specific microbial alterations and trans‐species changes. The disrupted microbiome balance and functional changes, characterized by an enrichment of pro‐inflammatory bacteria and a depletion of anti‐inflammatory butyrate‐producing bacteria, were the main cross‐species microbial characteristics of depression. Identifying microbial biomarkers and analyzing the effects of various confounders is critical to developing microbiota‐based diagnostics and therapeutics and advancing microbiota‐oriented precision medicine for depression. The evidence summarized here provides a foundation for such developments.

## 5 Experimental Section

### Search Strategy

PubMed, Web of Science, Embase, MEDLINE, PsycINFO, and Cochrane Library databases were searched for published articles that investigated commensal microbiota changes in depression from the database inception up to 04 November 2021, by combining the terms “microb*” and “depress*.” The search strings used are detailed in Table S14, Supporting Information. The reference lists of relevant literature reviews and the included publications were screened. A total of 20 380 records were identified; this included 20 349 records from databases, 28 recently published papers identified in the second search (PubMed on April 6, 2022), and 3 references from the included articles. The study was conducted by following the Preferred Reporting Items for Systematic reviews and Meta‐analyses (PRISMA) statement recommendations.^[^ ^84^ ^]^

### Eligibility Criteria and Study Selection

Retrieved records were imported into Endnote X9 software, duplicates were removed, and the remaining records were then manually reviewed based on the following inclusion criteria: 1) profiling the entire commensal microbiota using 16S rRNA gene sequencing (16S), whole‐genome shotgun metagenomic sequencing (metagenomics), and metaproteomics, or identifying specific taxa of interest using PCR and culture‐based methods; 2) assessing the commensal microbiota composition in patients with a clinical diagnosis of MDD or participants with depression symptoms or animal depression models (analyzing the microbiota compositional differences between cases and controls), or 3) investigating the associations between commensal microbiota and depression symptom measures in relevant conditions (MDD/depression) or healthy participants; and 4) peer‐reviewed, full‐text articles published in English. Only baseline data from intervention studies were included and excluded studies with negative results.

Titles and abstracts were reviewed independently by two investigators (LLX and CXY) to screen potential articles, then full‐text screening was performed to further assess whether these articles met the inclusion and exclusion criteria. Discrepancies were resolved via discussion and consultation with a third investigator (WHY).

### Data Extraction

Two investigators independently extracted the data from the eligible articles with a predesigned data extraction sheet by reviewing the main text and supplementary materials. The extracted relevant information included: publication details, study design, participant demographics and clinical characteristics for human studies, animal species and stress categories for animal studies, depression definition and severity measure, potential confounding factors, biological sample type and processing, commensal microbiome estimation methods, and commensal microbiota outcome data (e.g., *α*‐ and *β*‐diversity, taxonomic findings at different levels).

### Synthesis

Bacterial *α*‐diversity is a quantitative measure of the microbial community within individual samples and can be used to evaluate the effects of relevant conditions on the richness (number of species), evenness (relative abundance of each species), and diversity (composite of richness and evenness) of bacteria by comparing across groups.^[^ ^85^, ^86^ ^]^ A series of metrics were adopted for estimating the microbial communities' *α*‐diversity, such as Chao, Ace, and Sobs indices for richness; simpsoneven and shannoneven indices for evenness; Shannon, Simpson, and invsimpson indices for diversity; and other metrics such as observed species and phylogenetic diversity. Generally, *α*‐diversity reflected the community stability and function,^[^ ^39^ ^]^ and a lower diversity was considered a marker of disease states.^[^ ^87^ ^]^ This study investigated whether MDD or depressive conditions influence the richness, evenness, and diversity of commensal bacteria compared to a healthy state.

Bacterial *β*‐diversity is an inter‐individual measure that provides a summary of the similarity of microbial communities between groups.^[^ ^88^ ^]^ The *β*‐diversity analysis consisted of distance calculation and visualization. The 3rd English edition of “Numerical ecology” edited by Legendre et al. suggested more than 30 algorithms to calculate the similarity and ecological distances between microbial communities,^[^ ^89^ ^]^ the most frequently used approaches in the included studies were Bray–Curtis and UniFrac (weighted and unweighted) distance measures. Dimension reduction strategies were employed to visualize the calculated data using PCA, PCoA, and non‐metric multidimensional scaling , which allowed to see whether disease samples were significantly clustered separately from those of controls, to indicate the microbiota compositional divergence between groups.^[^ ^88^ ^]^ This study investigated whether MDD or depressive conditions alter commensal microbiota composition compared with controls.

For microbial taxonomy synthesis, taxa at phylum, class, order, family, genus, and species levels, were identified and included microbiota with significantly different relative abundances compared to controls and taxa that discriminated MDD or depressive conditions from controls, and those that were associated with depression severity. To avoid the false positive findings identified in a previous meta‐analysis,^[^ ^90^ ^]^ microbial results were excluded that were reported only by a single study. To identify characteristic microbial biomarkers in depression, within‐ and between‐species (i.e., human, rat, mouse, and nonhuman primate) comparisons were performed. Taxa reported by at least two studies were primarily summarized within‐species. Similar to the standards used by Nikolova et al.,^[^ ^21^ ^]^ consistent findings by two studies were considered as potential markers for further validation, while findings reported by three or more studies were considered to be characteristic microbial biomarkers for depression. Taxa altered only in a single species, or increased or decreased in one species, in a direction opposite to changes in other species, were regarded as candidates for species‐specific response in depression. Alternatively, taxa that showed consistent alterations among species (increased or decreased) or consistent expression trends among species, were considered trans‐species alterations; these consistent alterations were key for the translation of animal findings to human studies.

### Species Specificity and Confounder Analysis

A variety of confounding factors, such as genetic predisposition, geographic region, environmental factors, diet, and antibiotic or non‐antibiotic drug use, can affect microbial composition.^[^ ^91^, ^92^, ^93^ ^]^ Different species with different genetic backgrounds presented considerable differences in gut microbiota composition.^[^ ^43^ ^]^ Thus, species specificity analysis (i.e., human, rat, mouse, and nonhuman primate) was first performed. For depressed patients, the composition of commensal microbiota of patients from different countries (e.g., America, China, and the United Kingdom) and with different depression types (i.e., MDD and depression) were analyzed. In addition, different gastrointestinal segments presented with different microbial profiles; differences were also found in the intestinal mucosal versus luminal microbiome.^[^ ^33^ ^]^ Thus, subgroup analyses for microbiome habitat were performed in different gastrointestinal locations from the oral cavity to the rectum, and feces.

### Microbiota‐Targeted Interventions for Depression

To gain an understanding of the development of gut microbiota‐targeted intervention strategies for depression, PubMed (the most complete database) was searched for published articles until April 6, 2022 with the search strings shown in Table S15, Supporting Information. Articles that investigated the efficacy of microbiota‐targeted interventions (e.g., probiotics, prebiotics, synbiotics, and FMT) to alleviate depression in patients and animal models were included; the data could include comparisons between‐group differences between treatment and control groups, or within‐group differences from baseline to post‐intervention. 3633 records were identified. The study selection procedure was performed as described above and the following information was extracted: object, depression types, classification of microbiota‐targeted interventions, administration methods, effective outcomes, and underlying mechanisms in the gut–brain axis. The methodological quality of each included clinical study was assessed using the Oxford Centre for Evidence‐Based Medicine (OCEBM) 2011 Levels of Evidence.^[^ ^94^ ^]^

## Conflict of Interest

The authors declare no conflict of interest.

## Authors Contribution

L.L., H.W., and H.Z. contributed equally to this work. Conceptualization: P.X. and L.L. Methodology: H.W., X.C., and Y.Z. Investigation: L.L. and H.Z. Visualization: J.W., D.W., and J.P. Funding acquisition: P.X., L.L., and H.W. Supervision: P.X. and P.J. Writing–original draft: L.L. Writing–review & editing: P.X. and L.Z.

## Supporting information

Supporting Information

Supporting Information

Supporting Information

## Data Availability

The data that support the findings of this study are available in the supplementary material of this article.
