# Supplementary material for: Toward a Deeper Understanding of Gut Microbiome in Depression: The Promise of Clinical Applicability
Source: Adv Sci (Weinh). 2022 Oct 26;9(35):2203707. doi: 10.1002/advs.202203707 (PMC9762301; doi:10.1002/advs.202203707)
Supplement: Supplementary file 1 — Supporting Information [file ADVS-9-2203707-s001.pdf]

ADVANCED SCIENCE
Open Access

# Supporting Information

for *Adv. Sci.*, DOI 10.1002/advs.202203707

Toward a Deeper Understanding of Gut Microbiome in Depression: The Promise of Clinical Applicability

*Lanxiang Liu, Haiyang Wang, Hanping Zhang, Xueyi Chen, Yangdong Zhang, Ji Wu, Libo Zhao, Dongfang Wang, Juncai Pu, Ping Ji and Peng Xie**

# Supporting Information

**Supplementary Figures**

**Figure S1.** PRISMA flowchart.

**Figure S2.** Microbial α-diversity in patients with depression.

**Figure S3.** Microbial α-diversity in animal models of depression.

**Figure S4.** PRISMA flowchart for microbiota-based interventions of depression.

**Supplementary Tables**

**Table S1.** Characteristics of studies investigating gut microbiota composition in patients with depression.

**Table S2.** Characteristics of studies investigating gut microbiota composition in animal models of depression.

**Table S3.** Microbiota β-diversity in patients with depression.

**Table S4.** Microbial β-diversity in animal models of depression.

**Table S5.** Commensal microbiota alterations at Phylum, Class, Order, and Family levels in patients with depression.

**Table S6.** Commensal microbiota alterations at Genus level in patients with depression.

**Table S7.** Commensal microbiota alterations at Species level in patients with depression.

**Table S8.** Commensal microbiota alterations at Phylum, Class, Order, and Family levels in animal models of depression.

**Table S9.** Commensal microbiota alterations at Genus level in animal models of depression.

**Table S10.** Commensal microbiota alterations at Species level in animal models of depression.

**Table S11.** Characteristics of studies investigating the efficiency of gut microbiotabased therapeutics in patients with depression.

**Table S12.** Characteristics of studies investigating the efficiency of gut microbiotabased therapeutics in animal models of depression.

**Table S13.** A summary of probiotics that alleviate depression symptoms.

**Table S14.** Search strategy for electronic databases.

**Table S15.** Search strategy for microbiota-based interventions of depression from PubMed.
