# Supplementary material for: Toward a Deeper Understanding of Gut Microbiome in Depression: The Promise of Clinical Applicability
Source: Adv Sci (Weinh). 2022 Oct 26;9(35):2203707. doi: 10.1002/advs.202203707 (PMC9762301; doi:10.1002/advs.202203707)

ADVANCED SCIENCE
Open Access

# Supporting Information

for *Adv. Sci.*, DOI 10.1002/advs.202203707

Toward a Deeper Understanding of Gut Microbiome in Depression: The Promise of Clinical Applicability

*Lanxiang Liu, Haiyang Wang, Hanping Zhang, Xueyi Chen, Yangdong Zhang, Ji Wu, Libo Zhao, Dongfang Wang, Juncai Pu, Ping Ji and Peng Xie**

Supplementary Tables

# Towards a deeper understanding of gut microbiome in depression: the promise of clinical applicability

*Lanxiang Liu[1,3,4]†, Haiyang Wang[2,3]†, Hanping Zhang[3,4]†, Xueyi Chen[3], Yangdong Zhang[3,4], Ji Wu[3,4], Libo Zhao[1], Dongfang Wang[3], Juncai Pu[3,4], Ping Ji[2], Peng Xie[1,2,3,4]**

**Figure S1.** PRISMA flowchart.

**Figure S2.** Microbial α-diversity in patients with depression.

**Figure S3.** Microbial α-diversity in animal models of depression.

**Figure S4.** PRISMA flowchart for microbiota-based interventions of depression.

**Figure S1.** PRISMA flowchart.

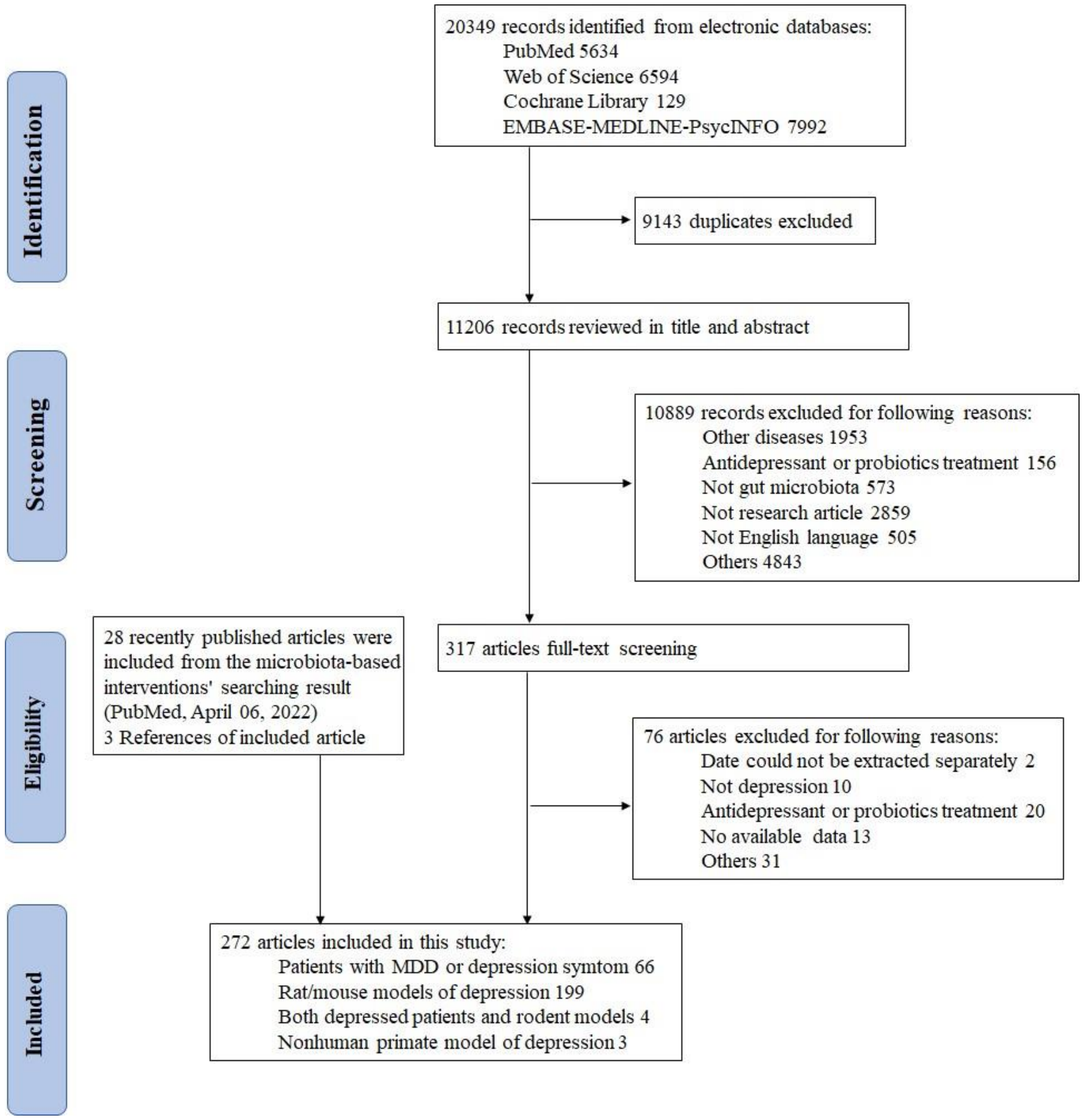

**Figure S2.** Microbial α-diversity in patients with depression.

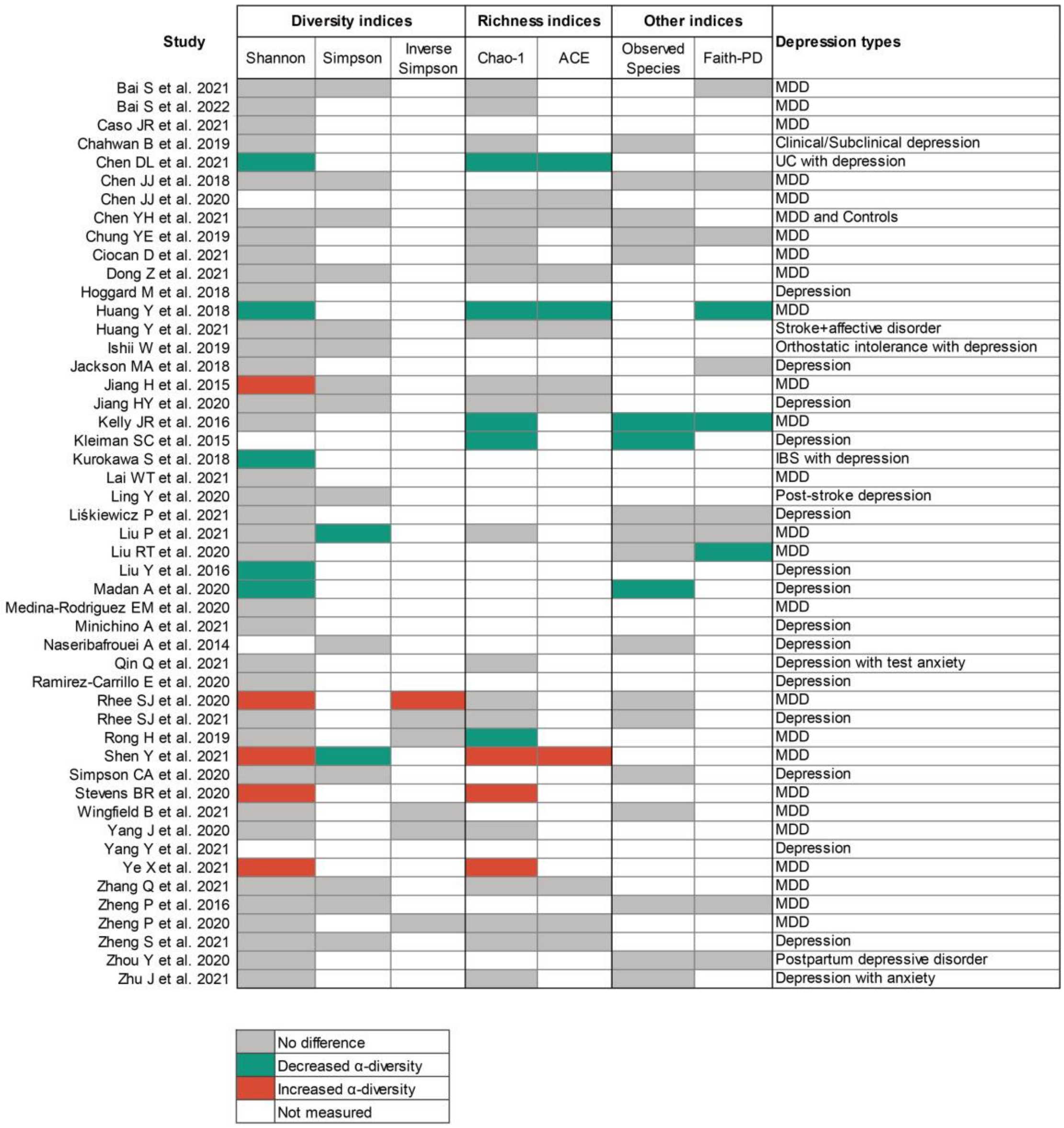

**Figure S3.** Microbial α-diversity in animal models of depression.

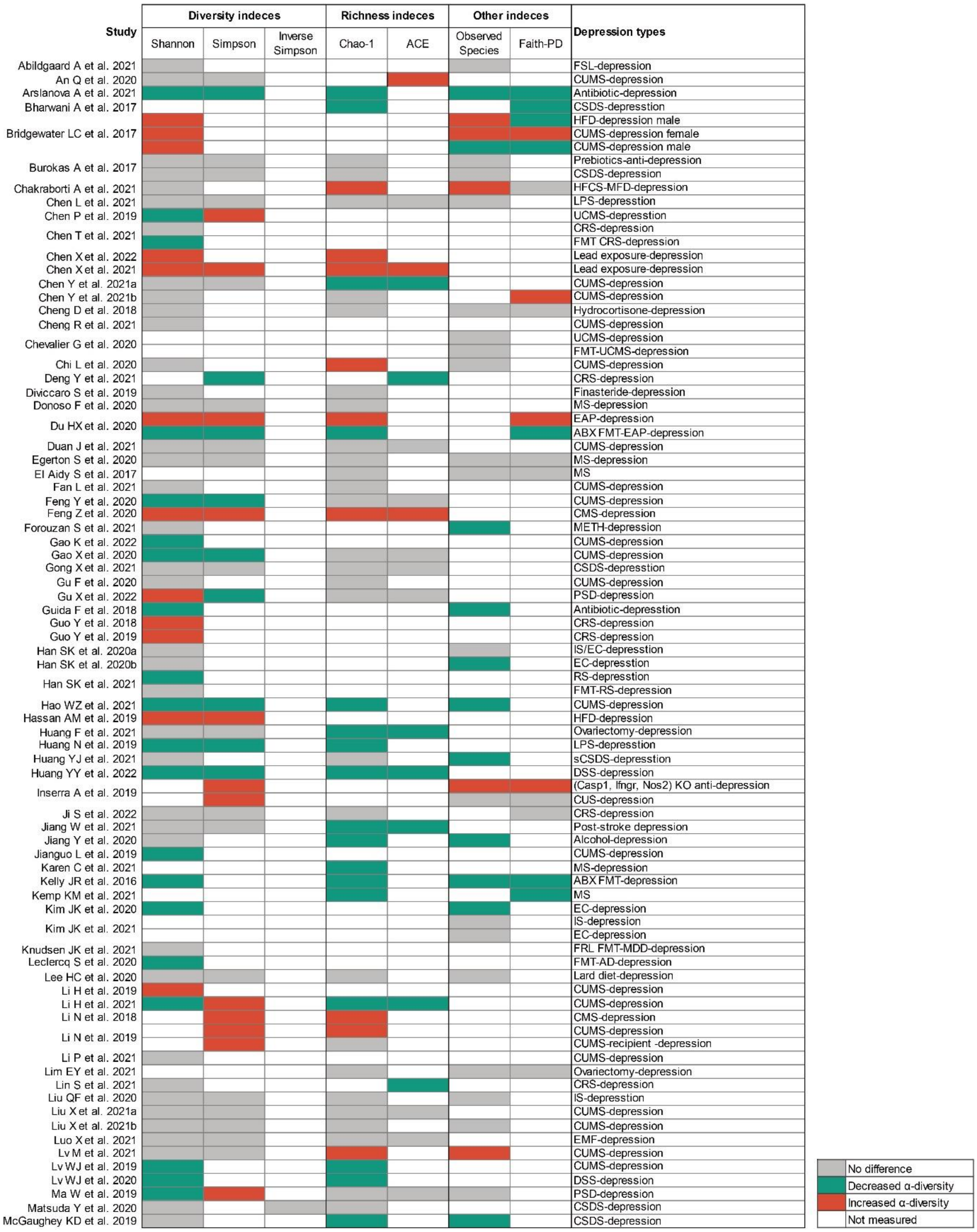


*(Continue)*

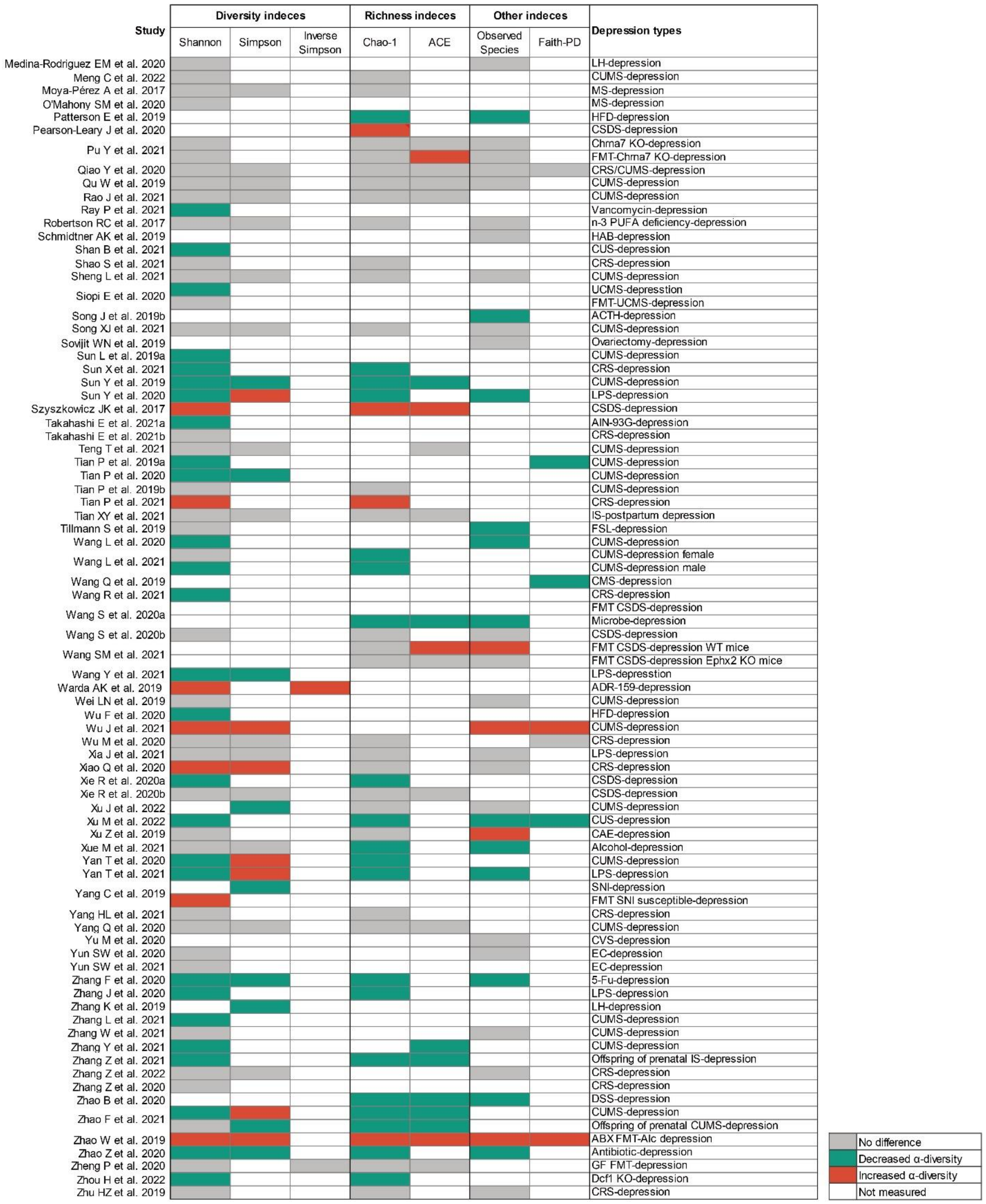
Study
Diversity indeces
Richness indeces
Other indeces
Shannon
Simpson
Inverse Simpson
Chao-1
ACE
Observed Species
Faith-PD
Depression types
Medina-Rodriguez EM et al. 2020
Meng C et al. 2022
Moya-Pérez A et al. 2017
O'Mahony SM et al. 2020
Patterson E et al. 2019
Pearson-Leary J et al. 2020
Pu Y et al. 2021
Qiao Y et al. 2020
Qu W et al. 2019
Rao J et al. 2021
Ray P et al. 2021
Robertson RC et al. 2017
Schmidtner AK et al. 2019
Shan B et al. 2021
Shao S et al. 2021
Sheng L et al. 2021
Siopi E et al. 2020
Song J et al. 2019b
Song XJ et al. 2021
Sovijit WN et al. 2019
Sun L et al. 2019a
Sun X et al. 2021
Sun Y et al. 2019
Sun Y et al. 2020
Szyszkowicz JK et al. 2017
Takahashi E et al. 2021a
Takahashi E et al. 2021b
Teng T et al. 2021
Tian P et al. 2019a
Tian P et al. 2020
Tian P et al. 2019b
Tian P et al. 2021
Tian XY et al. 2021
Tillmann S et al. 2019
Wang L et al. 2020
Wang L et al. 2021
Wang Q et al. 2019
Wang R et al. 2021
Wang S et al. 2020a
Wang S et al. 2020b
Wang SM et al. 2021
Wang Y et al. 2021
Warda AK et al. 2019
Wei LN et al. 2019
Wu F et al. 2020
Wu J et al. 2021
Wu M et al. 2020
Xia J et al. 2021
Xiao Q et al. 2020
Xie R et al. 2020a
Xie R et al. 2020b
Xu J et al. 2022
Xu M et al. 2022
Xu Z et al. 2019
Xue M et al. 2021
Yan T et al. 2020
Yan T et al. 2021
Yang C et al. 2019
Yang HL et al. 2021
Yang Q et al. 2020
Yu M et al. 2020
Yun SW et al. 2020
Yun SW et al. 2021
Zhang F et al. 2020
Zhang J et al. 2020
Zhang K et al. 2019
Zhang L et al. 2021
Zhang W et al. 2021
Zhang Y et al. 2021
Zhang Z et al. 2021
Zhang Z et al. 2022
Zhang Z et al. 2020
Zhao B et al. 2020
Zhao F et al. 2021
Zhao W et al. 2019
Zhao Z et al. 2020
Zheng P et al. 2020
Zhou H et al. 2022
Zhu HZ et al. 2019
LH-depression
CUMS-depression
MS-depression
MS-depression
HFD-depression
CSDS-depression
Chrna7 KO-depression
FMT-Chrna7 KO-depression
CRS/CUMS-depression
CUMS-depression
CUMS-depression
Vancomycin-depression
n-3 PUFA deficiency-depression
HAB-depression
CUS-depression
CRS-depression
CUMS-depression
UCMS-depresstion
FMT-UCMS-depression
ACTH-depression
CUMS-depression
Ovariectomy-depression
CUMS-depression
CRS-depression
CUMS-depression
LPS-depression
CSDS-depression
AIN-93G-depression
CRS-depression
CUMS-depression
CUMS-depression
CUMS-depression
CUMS-depression
CRS-depression
IS-postpartum depression
FSL-depression
CUMS-depression
CUMS-depression female
CUMS-depression male
CMS-depression
CRS-depression
FMT CSDS-depression
Microbe-depression
CSDS-depression
FMT CSDS-depression WT mice
FMT CSDS-depression Ephx2 KO mice
LPS-depresstion
ADR-159-depression
CUMS-depression
HFD-depression
CUMS-depression
CRS-depression
LPS-depression
CRS-depression
CSDS-depression
CSDS-depression
CUMS-depression
CUS-depression
CAE-depression
Alcohol-depression
CUMS-depression
LPS-depression
SNI-depression
FMT SNI susceptible-depression
CRS-depression
CUMS-depression
CVS-depression
EC-depression
EC-depression
5-Fu-depression
LPS-depression
LH-depression
CUMS-depression
CUMS-depression
CUMS-depression
Offspring of prenatal IS-depression
CRS-depression
CRS-depression
DSS-depression
CUMS-depression
Offspring of prenatal CUMS-depression
ABX FMT-Alc depression
Antibiotic-depression
GF FMT-depression
Dcf1 KO-depression
CRS-depression
No difference
Decreased α-diversity
Increased α-diversity
Not measured

**Figure S4.** PRISMA flowchart for microbiota-based interventions of depression.

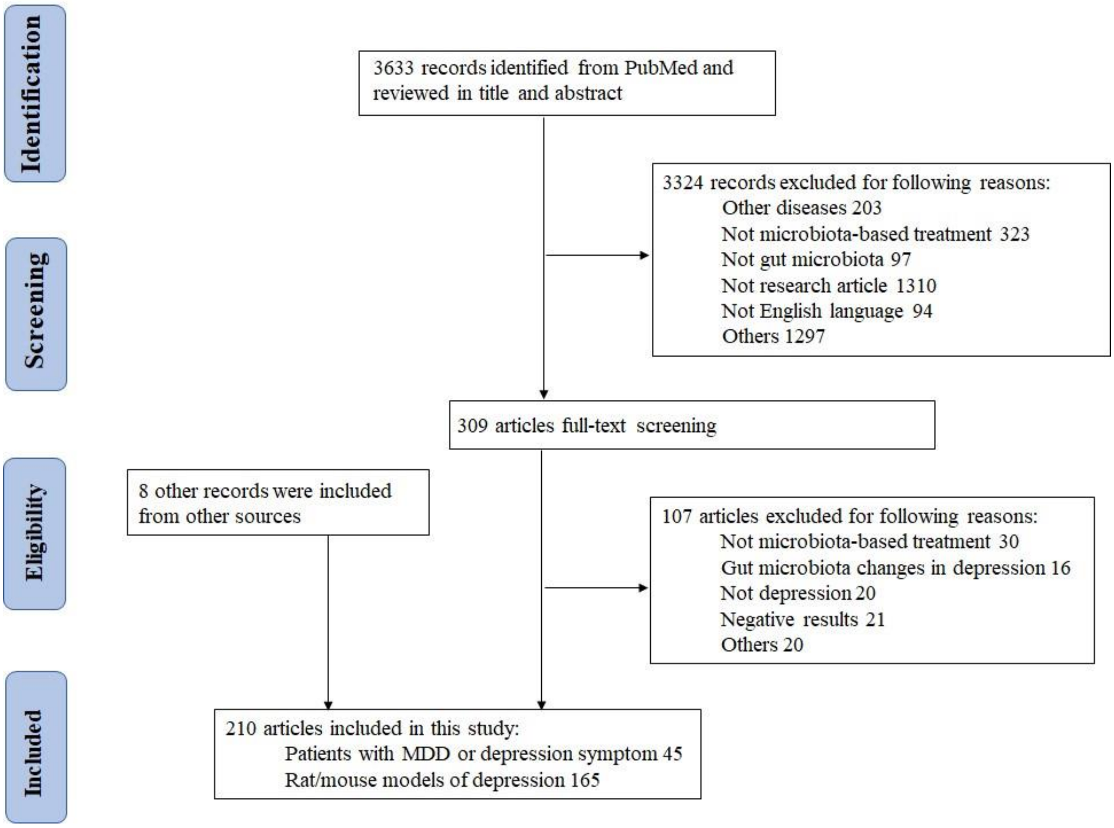

Supplement: Supplementary file 2 — Supporting Information [file ADVS-9-2203707-s002.pdf]
