# Supplementary material for: Toward a Deeper Understanding of Gut Microbiome in Depression: The Promise of Clinical Applicability
Source: Adv Sci (Weinh). 2022 Oct 26;9(35):2203707. doi: 10.1002/advs.202203707 (PMC9762301; doi:10.1002/advs.202203707)
Supplement: Supplementary file 3 — Supporting Information [file ADVS-9-2203707-s003.pdf]

ADVANCED SCIENCE
Open Access

Supporting Information

for *Adv. Sci.*, DOI 10.1002/advs.202203707

Toward a Deeper Understanding of Gut Microbiome in Depression: The Promise of Clinical Applicability

*Lanxiang Liu, Haiyang Wang, Hanping Zhang, Xueyi Chen, Yangdong Zhang, Ji Wu, Libo Zhao, Dongfang Wang, Juncai Pu, Ping Ji and Peng Xie**

Supplementary Tables

Towards a deeper understanding of gut microbiome in depression: the promise of clinical applicability

*Lanxiang Liu[1,3,4]†, Haiyang Wang[2,3]†, Hanping Zhang[3,4]†, Xueyi Chen[3], Yangdong Zhang[3,4], Ji Wu[3,4], Libo Zhao[1], Dongfang Wang[3], Juncai Pu[3,4], Ping Ji[2], Peng Xie[1,2,3,4]**

Table S1. Characteristics of studies investigating gut microbiota composition in patients with depression.

Table S2. Characteristics of studies investigating gut microbiota composition in animal models of depression.

Table S3. Microbiota β-diversity in patients with depression.

Table S4. Microbial β-diversity in animal models of depression.

Table S5. Commensal microbiota alterations at Phylum, Class, Order, and Family levels in patients with depression.

Table S6. Commensal microbiota alterations at Genus level in patients with depression.

Table S7. Commensal microbiota alterations at Species level in patients with depression.

Table S8. Commensal microbiota alterations at Phylum, Class, Order, and Family levels in animal models of depression.

Table S9. Commensal microbiota alterations at Genus level in animal models of depression.

Table S10. Commensal microbiota alterations at Species level in animal models of depression.

Table S11. Characteristics of studies investigating the efficiency of gut microbiotabased therapeutics in patients with depression.

Table S12. Characteristics of studies investigating the efficiency of gut microbiotabased therapeutics in animal models of depression.

Table S13. A summary of probiotics that alleviate depression symptoms.

Table S14. Search strategy for electronic databases.

Table S15. Search strategy for microbiota-based interventions of depression from PubMed.

Table S1. Characteristics of studies investigating gut microbiota composition in patients with depression.

Study	People	Study design	groups	Age (Years) (mean ±SD) Patients Control	Sex (M/F) Patients Control	BMI Patients Control	Comorbidity	Drug treatment in patients	Diagnostic criteria	Severity measure	Severity (mean ±SD) Patients Control	Sample	Storage	Gut microbiome estimation	Sequencing region	Microbial biomarkers from comparisons	Microbial biomarkers selection
Aizawa E et al. 2016[1]	Japanese	Case-control	MDD (N=43)<br>Control (N=57)	39.4(10.0)<br>42.8(12.7)	25/18<br>22/35	23.2(3.6)<br>22.3(3.7)	In MDD: 14 IBS;<br>In controls: 7 IBS	In MDD: 28 Imipramine	DSM-IV	HAMD-21	16.9(6.8)<br>NA	Fecal samples	4 ℃ and -80 ℃	RT-qPCR for Bifidobacterium and Lactobacillus	NA	MDD and Controls	MannWhitney U test: $p < 0.05$
Bai S et al. 2021[2]	Chinese	Case-control	MDD (N=60)<br>Control (N=60)	35.62(17.10)<br>35.13(15.79)	21/39<br>24/36	20.90(2.30)<br>21.19(4.29)	NA	NA	DSM-IV,<br>HAMD-17 > 17	HAMD-17	25.3(6.01)<br>0.67(0.93)	Fecal samples	−80°C	16S rRNA gene sequencing-Illumina MiSeq platform	V3-V4	MDD and Controls	LEfSe: $p < 0.05$ and LDA > 2.0
Bai S et al. 2022[3]	Chinese	Case-control	MDD (N=56)<br>Control (N=56)	35.11(16.79)<br>35.71(15.99)	18/38<br>20/36	20.94(2.37)<br>21.23(4.27)	NA	NA	DSM-IV-TR	HDRS	24.86(5.94)<br>0.71(0.95)	Fecal samples	−80°C	16S rRNA gene sequencing-Illumina MiSeq platform	V3-V4	MDD and Controls	LEfSe: $p < 0.05$ and LDA > 2.0
Caso JR et al. 2021[4]	Spainish	Case-control	Active MDD (N=46)<br>Remission MDD (N=22)<br>Control (N=45)	42.1<br>45.85<br>44.72	10/36<br>5/17<br>11/34	_	_	_	DSM-IV-TR,<br>HDRS≥14	HDRS	21.17<br>10.54<br>0.08	Fecal samples	−80°C	16S rRNA gene sequencing-Illumina MiSeq platform	V3-V4	Active MDD and Controls	Kruskal-Wallis test followed by Bonferroni-adjusted analysis: $p < 0.05$
Chahwan B et al. 2019[5]	Australian	Case-control	Depression (N=71)<br>(N=68 provided stool samples)<br>Controls (N=20)	36.05(11.99)<br>35.95(11.74)	22/49<br>5/15	_	_	In depression: 38 Antidepressant, 46 Psychological treatment	DSM-IV MINI;<br>ICD-10	BDI-II	28.43 (9.90)<br>2.80 (2.78)	Fecal samples	−80°C	16s rRNA gene sequencing-Illumina MiSeq platform	V3-V4	Clinical/Subclinical Depression and Controls	Kruskal Wallis test with the False Discovery Rate (FDR) correction: adjusted $p < 0.05$<br>Spearman's Correlations (with FDR correction for multiple testing): adjusted $p < 0.05$
Chen DL et al. 2021[6]	Chinese	Case-control	UC (N=31)<br>UC with depression (N=31)<br>Control (N=31)	44.16(11.49)<br>41.94(13.42)<br>43.13(10.98)	17/14<br>16/15<br>16/15	22.8(1.9)<br>22.1(2.1)<br>22.4(2.6)	ulcerative colitis	NA	SDS≥53	SDS	_	Fecal samples	−80°C	16S rRNA gene sequencing-pyrosequencing	V3-V4	UC with depression and Controls	ANOVA: $p < 0.05$
Chen JJ et al. 2018[7]	Chinese	Case-control	MDD-female (N=24)<br>control-female(N=24)<br><br>MDD-male (N=20)<br>control-male(N=20)	41.5(11.53)<br>43.95(12.11)<br><br>40.35(11.05)<br>42.80(15.13)	0/24<br>0/24<br><br>20/0<br>20/0	22.01(2.17)<br>22.63(2.43)<br><br>22.22(2.18)<br>22.50(2.25)	3 female patients had coexisting anxiety disorders.<br><br>2 male patients had coexisting anxiety disorders.	_	DSM-IV	HDRS-17	23.04(4.93)<br>_<br><br>23.9(3.68)<br>_	Fecal samples	−80°C	16s rRNA gene sequencing-Roche 454 sequencing	V3-V5	Female and male MDD and corresponding controls	Lefse: $p < 0.05$ and LDA > 2.0
Chen JJ et al. 2020[8]	Chinese	Case-control	MDD-young(N=25)<br>control-young(N=27)<br><br>MDD-middle-aged (N=45)<br>control-middle-aged(N=44)	24.0(3.74)<br>24.96(2.31)<br><br>44.96(7.76)<br>47.16(8.07)	7/18<br>8/19<br><br>14/31<br>10/34	22.13(2.24)<br>21.53(2.37)<br><br>22.64(2.64)<br>23.23(2.33)	NA	In young MDD: 7 medications<br><br>In middle-aged MDD: 14 medications	DSM-IV	HDRS-17	22.64(3.18)<br>0.29(0.61)<br><br>23.0(4.61)<br>0.34(0.74)	Fecal samples	−80°C	16s rRNA gene sequencing-Roche 454 sequencing	V3-V5	Young and middle-aged MDD and corresponding controls	Lefse: $p < 0.05$ and LDA > 2.0
Chen T et al. 2021[9]	Chinese	Case-control	UC with depression (N=16)<br>UC (n=19)	41.31(12.2)<br>43.68(12.73)	7/9<br>10/9	22.49(2.64)<br>21.08(3.44)	Ulcerative colitis (UC)	NA	PHQ-9≥10	PHQ-9	13.75(3.96)<br>2.26(1.35)	Fecal samples	_	qRT-PCR for Akkermansia muciniphila	NA	UC with depression and UC	One-way analysis of variance (ANOVA) with Tukey–Kramer test: $p < 0.05$

Chen Y et al. 2021[10]	English	Cross-sectional associations	Depression (N=113693)	56.23	51162/62531	_	_	_	PHQ-9	PHQ-9	2.71 (3.64)	Fecal samples	_	16S rRNA gene sequencing-Illumina HiSeq platform	V4	Cross-sectional associations with depression symptom measures	Linear regression model: $p < 0.05$
Chen YH et al. 2021[11]	Chinese	Case-control	MDD-female (N=62)<br>Control-female(N=46)<br><br>MDD-female (N=20)<br>Control-female(N=21)	39.58 (12.66)<br>36.93(8.58)<br><br>Subgroup	0/62<br>0/46<br><br>0/20<br>0/21	21.99(2.93)<br>22.24(2.92)<br><br>Subgroup	NA	In MDD: 26 Antidepressants for a short period of time<br><br>_	DSM-5<br>HAMD-17≥18	HAMD-17	27.97(4.63)<br>NA<br><br>Subgroup	Fecal samples	−80◦C	16S rRNA gene sequencing-Illumina MiSeq platform<br><br>Shotgun metagenomic sequencing<br>Illumina NovaSeq 6000	V3-V4<br><br><br>NA	MDD and Controls	LEfSe: $p < 0.05$ and LDA > 2.4<br>Wilcoxon test followed by FDR correction: $p < 0.05$
Chen Z et al. 2018[12]	Chinese	Case-control	MDD (N=10)<br>Control (N=10)	43.9(13.8)<br>39.6 (9)	5/5<br>5/5	23.5(2.0)<br>22.6(1.5)	NA	In MDD: 2 SSRIs or SNRIs treatment	DSM-IV<br>HAMD-17≥20	HAMD-17	25.6(4.7)<br>NA	Fecal samples	−80 °C	Metaproteomics: phylogenetic analysis of bacterial peptides	NA	MDD and Controls	Mann–Whitney test: $p < 0.05$ and fold change >1.5
Cheng S et al. 2020[13]	European	Cross-sectional associations	MDD (N=135458)<br>Control (N=344901)	_	_	_	_	_	DSM-III, DSM-IV, ICD-9, or ICD-10	_	_	Fecal samples	_	Microbiota-related gene set enrichment analysis (GSEA)-Illumina MiSeq/Illumina HiSeq2000/Illumina HiSeq	_	Cross-sectional associations between MDD and gut microbiota using published GWAS data	Unspecified
Chung YE et al. 2019[14]	Chinese	Case-control	MDD (N=36)<br>Control (N=37)	45.83 (14.08)<br>41.19 (12.73)	8/28<br>14/23	22.80 (4.21)<br>23.95((3.92)	NA	In MDD: 31 Antidepressant use	DSM-5	BDI	19.18 (12.47)<br>4.54 (4.85)	Fecal samples	−80 °C	16S rRNA gene sequencing-Illumina MiSeq/MiniSeq platforms	V3-V4/V4	MDD and Controls	Analysis of composition of microbiomes (ANCOM): $p < 0.05$
Ciocan D et al. 2021[15]	French	Case-control	MDD (N=56)<br>Control (N=56)	41.9(11.6)<br>41.9(12.7)	18/38<br>17/39	24(5.3)<br>_	In MDD: 3 Diabetes	Antidepressant-free for at least 1 year at baseline<br>Longitudinal intervention in MDD: 25 Venlafaxine; 19 Citalopram; 12 Escitalopram	DSM-IV-TR<br>MINI<br>HDRS-17≥18	HDRS-17	_	Plasma sample	−80◦C	16S rRNA gene sequencing-MiSeq technology (Vaiomer,Labège, France)	V3-V4	MDD and Controls (baseline)	LEfSe: $p < 0.05$ and LDA > 2.0
Dong Z et al. 2021[16]	Chinese	Case-control	MDD (N=23)<br>GAD (N=21)<br>Control (N=10)	30.04(5.90)<br>30.43(7.95)<br>30.22(6.50)	7/16<br>7/14<br>4/6	21.87(3.00)<br>21.19(2.89)<br>21.45(2.80)	NA	NA	DSM-5<br>HAMD-24 ≥20	HAMD-24	29.26(7.51)<br>12.10(5.25)<br>NA	Fecal samples	−80◦C	16S rRNA gene sequencing-Illumina MiSeq platform	V3-V4	MDD and Controls	ANOVA: $p < 0.05$
Fontana A et al. 2020[17]	Italian	Case-control	MDD (N=34)<br>Control (N=20)	55.58(43.3-61.6)<br>37.7(30.6–58.0)	10/24<br>13/7	23.96(21.0-28.8)<br>22.7 (21.2–23.8)	In MDD: 8 Cardiometabolic comorbidities;<br>In controls: 4 Cardiometabolic comorbidities	In MDD: 12 SSRI; 15 SNRI/TCA/Serotonin modulator; 7 Mood stabilizers;<br>7 Antipsychotics; 11 Any concomitant drugs	DSM IV-TR	_	_	Fecal samples	−80◦C	16S rRNA gene sequencing-Illumina MiSeq platform	V3-V4	MDD (treatment responsive, treatment-resistant and untreated at the moment of sampling) and controls	Two-sample t-test and Mann–Whitney U test: $p < 0.05$
Heym N et al. 2019[18]	English	Cross-sectional associations	Depression(N=40)	36.38(11.43)<br>male:36.77(13.24)<br>female: 36.19(10.72)	13/27	_	_	7 patients: contraceptives; 3 patients:blood pressure and statins; 1 patient:thyroid; 1 patient:stomach acid; 1 patient:acne; 1 patient:asthma	BDI-II	BDI-II	_	Fecal samples	− 20 ℃	16S rRNA–targeted oligonucleotide probes for Bif164 (bifidobacteria) and Lab158 (lactobacilli)	_	Psychological risk and resilience factors for depression	Pearson's zero-order correlations
Hoggard M et al. 2018[19]	New zealanders	Cross-sectional associations	Chronic rhinosinusitis (N=14)<br>Control (N=12)	59.5(29-74)<br>41.5(21-76)	8/6<br>2/10	_	In CRS: 14 chronic rhinosinusitis, 6 Asthma, 3 Diabetes, 3 GERD, 12 Allergy (medications), 7 Allergy	In CRS: 3 antibiotics, 3 systemic corticosteroids, 6 topical corticosteroids;	_	PHQ-9	6(0-12)<br>4.5(0-11)	Sinonasal swab and	_	16S rRNA gene sequencing-Illumina MiSeq platform	Unspecified	Cross-sectional associations with depression symptom measures	Spearman correlation and linear regression analysis

							(other), 4 Polyposis; Control: 2 Asthma, 1 Diabetes, 6 Allergy (medications), 6 Allergy (other)	Control: 1 antibiotics, 1 systemic corticosteroids, 4 topical corticosteroids				mucus samples					
Huang Y et al. 2018[20]	Chinese	Case-control	MDD (N=27)<br>Control (N=27)	48.7(12.8)<br>42.3(14.1)	7/20<br>7/20	23.8(2.8)<br>23.4(2.9)	NA	NA	ICD-10	_	_	Fecal samples	−80◦C	16S rRNA gene sequencing-Illumina Hiseq2500	V3–V4	First episode MDD and Controls	LEfSe: p < 0.01 and LDA > 2.0
Huang Y et al. 2021[21]	Chinese	Case-control	Stroke+affective disorder (N=20)<br>Stroke+cognitive impairment (N=29)<br>Stroke (N=27)<br>Control (N=19)	61.3(11.37)<br>62.76(9.01)<br>62.19(8.75)<br>NA	11/9<br>20/9<br>14/13<br>NA	_	In PSTD: 9 Hypertension, 5 Diabetes<br>In PSCI: 17 Hypertension, 9 Diabetes<br>In ST: 12 Hypertension, 4 Diabetes	_	_	Depression score	7.05(2.87)<br>1.52(1.27)<br>2.26(1.81)<br>NA	Fecal samples	−80◦C	16S rRNA gene sequencing-Illumina MiSeq platform	V3-V4	Stroke+affective disorder and stroke control	LEfSe: p < 0.05 and LDA > 2.0
Ishii W et al. 2019[22]	Japanese	Case-control	Depression-OI (N=15)<br>Nondepression-OI (N=41)<br>Control (N=9)	13.6(1.6)[a]<br>12.3(3.8)	21/35[a]<br>3/6	18.8(2.9)[a]<br>18.0(2.2)	56 Orthostatic Intolerance; 33 Anxiety	NA	CDI≥22	CDI	≥22	Fecal samples	−80◦C	16S rRNA gene-Terminal-restriction fragment length polymorphism (T-RFLP)	_	Orthostatic Intolerance with depression and controls	Mann-Whitney test and Kruskal-Wallis test: p<0.017
Jackson MA et al. 2018[23]	English	Cross-sectional associations	Depression(N=354) from all individuals(N=2737)	60(12)[b]	301/2436[b]	26 (5)[b]	38 diseases(e.g., hypercholesterolaemia, respiratory allergies, anxiety, osteoarthritis, and hypertension)	51 prescription medications(e.g., statins, proton pump inhibitors, cholecalciferol, and calcium)	_	_	_	Fecal samples	−80 ℃	16s rRNA gene sequencing-Illumina MiSeq platform	V4	Cross-sectional associations with depression symptom measures	Beta coefficients of associations: p < 0.05
Jiang H et al. 2015[24]	Chinese	Case-control	MDD-active (N=29)<br>MDD-responded (N=17)<br>Control (N=30)	25.3 (5.4)<br>27.1 (5.4)<br>26.8 (5.4)	18/11<br>9/8<br>15/15	20.3 (3.4)<br>21.8 (3.4)<br>19.6 (3.4)	_	In MDD: 38 SSRIs or SNRIs, 12 Atypical antipsychotic, 34 Benzodiazepines	DSM-IV HAMDS-24≥20	HAMDS -24	29.8 (7.6)<br>8.3 (4.6)<br>NA	Fecal samples	−80◦C	16S rRNA gene sequencing-Roche 454 sequencing	V1–V3	Active-MDD, Responding MDD and Controls	LEfSe: p < 0.05 and LDA > 2.0
Jiang HY et al. 2020[25]	Chinese	Case-control	CDE(N=24)<br>Control (N=16)	37.2(7.2)<br>35.8(6.8)	13/11<br>9/7	23.6(7.1)<br>22.3(6.5)	_	In CDE: 11 Antidepressant; 16 Benzodiazepines; 9 Atypical antipsychotic; 7 Mood stabilizer	DSM-IV	HAMDS -24	25.3(6.8)<br>2.3(1.1)	Fecal samples	−80◦C	16S rRNA gene sequencing-Illumina MiSeq platform	V3–V4	Patients with current depressive episode (CDE) and controls	LEfSe: p < 0.05 and LDA > 2.0
Kang Y et al. 2021[26]	Chinese	Case-control	Post-stroke depression (N=67)<br>Stroke (N=96)	55.91(8.61)	85/78	_	Stroke	NA	CCMD-3, HAMD-24≥ 8, SDS index ≥ 0.5	_	_	Fecal samples	_	Enterococcus faecalis, Escherichia coli and Bifidobacterium in feces were detected by ATB-expression semi-automatic microbial detection system	_	Post-stroke depression and stroke	Independent sample t-test: p < 0.05
Kelly JR et al. 2016[27]	Irish	Case-control	MDD (N=34)<br>Control (N=33)	45.8(11.5)<br>45.8(11.9)	21/13<br>19/14	26.2(4.5)<br>24.58(2.7)	7 patients: Dyslipidaemia, 3 patients: Hypertension, 5 patients: BPAD II , 4 patients:Anxiety disorder<br>4 patients: Dyslipidaemia, 3 patients: Hypertension	All patients:SSRIs	DSM-IV MINI	HAMD-17	19.5 (14)<br>NA	Fecal samples	−80◦C	16S rRNA gene sequencing Illumina Miseq platform	Unspecified	MDD and Controls	Mann-Whitney U test and Benjamini-Hochberg FDR-adjusted p-value ≤ 0.1

Kleiman SC et al. 2015[28]	American	Cross-sectional associations	Anorexia Nervosa (N=16)<br>Control (N=12)	28.0 (±11.7)<br>29.8 (11.6)	0/16<br>0/12	16.2 (1.5)<br>21.5 (1.9)	NA	NA	_	BDI	26.6 (13.4)<br>NA	Fecal samples	−80°C	16S rRNA gene sequencing-454 Life Sciences Genome Sequencer FLX machine	V1-V3	Cross-sectional associations with depression symptom measures	Wilcoxon matched pairs rank test ($-2 \leq$ skewness ≤ 2) or the sign test (skewness ≤ -2 or ≥ 2): $p < 0.05$
Kurokawa S et al. 2018[29]	Japanese	Case-control	IBS with depression (N=12)<br>IBS without depression (N=5)<br>Donors (N=17)	43.41(10.75)[c]<br>51.41(18.11)	8/9[c]<br>7/10	_	IBS	NA	HAMD≥8	HAMD-	_	Fecal samples	−80°C	16S rRNA gene sequencing-Illumina MiSeq platform	V1-V2	IBS with depression and donors	Paired-t-test: $p < 0.05$
Lai WT et al. 2021[30]	Chinese	Case-control	MDD (N=26)<br>Control (N=29)	43.73 (11.46)<br>39.41(10.96)	8/18<br>13/16	21.17 (2.17)<br>21.10(2.23)	NA	In MDD: 12 SSRIs, 7 SNRIs, 6 Other antidepressants	DSM-5	HAMD-17	19.81 (2.95)<br>NA	Fecal samples	−80°C	Shotgun metagenomic-Illumina Hiseq2500 sequencer	NA	MDD and Controls	LEfSe: adjusted $p < 0.05$ and LDA > 3.0
Lin P et al. 2017[31]	Chinese	Case-control	MDD (N=10)<br>Control (N=10)<br><br>MDD (N=60)<br>Control (N=60)	36.2(10.1)<br>38.1(2.9)<br><br>20–85	6/4<br>6/4<br><br>_	23.8(1.9)<br>24.2(2.0)<br><br>_	_	Longitudinal intervention (all patients received Escitalopram)	DSM-IV-TR<br>HAM-D-17≥23	HAM-D-17	>23<br>_	Fecal samples	−70°C	16S rRNA gene sequencing-Illumina Miseq platform<br><br>qRT-PCR for Streptococcus, Clostridium XI, Prevotella and Klebsiella	V3–V4<br><br>_	MDD and Controls	Double-sided Student's t-test and<br>Wilcoxon's Sign Rank Test: $p < 0.01$
Ling Y et al. 2020[32]	Chinese	Case-control	PSCCID (N=41)<br>non-PSCCID (N=25)	69.63(9.35)<br>68.92(8.46)	17/24<br>14/11	25.14(3.62)<br>26.62(3.58)	In PSCCID: 24 Hypertension, 12 Diabetes mellitus; 15 Hyperlipidemia<br>In non-PSCCID: 16 Hypertension, 6 Diabetes mellitus; 10 Hyperlipidemia	Unknown	HAMD≥8	HAMD	13.6(3.50)<br>5.56(2.58)	Fecal samples	−80°C	16S rRNA gene sequencing-Illumina MiSeq platform	V3–V4	PSCCID and non-PSCCID	LEfSe: $p < 0.05$ and LDA > 2.0
Liśkiewicz P et al. 2021[33]	Polish	Cross-sectional associations	MDD(N=16)	44.0 (34.3–56.3)	8/8	25.0 (22.4–26.7)	NA	Longitudinal intervention (all patients received Escitalopram)	ICD-10	HAMDS-24	23.0 (21.0–28.5)	Fecal samples	_	16S rRNA gene sequencing-Illumina NextSeq 500 platform	V4	Cross-sectional associations between with symptom severity (baseline)	Spearman's rank correlation
Liu P et al. 2021[34]	Chinese	Case-control	MDD (N=66)<br>Control (N=43)	24.20(9.60)<br>23.67(3.19)	27/39<br>20/23	21.46(4.61)<br>21.81(2.15)	NA	NA	DSM-IV, HAMD-17≥17	HAMD-17	20.07(4.20)<br>2.31(2.04)	Fecal samples	−80°C	16S rRNA gene sequencing-Illumina Novaseq PE250 platform	V3-V4	MDD and Controls	LEfSe: $p < 0.05$ and LDA > 2.0
Liu RT et al. 2020[35]	American	Case-control	MDD (N=43)<br>Control (N=47)	21.9 (2.1)<br>22.1 (1.8)	5/38<br>13/34	_	NA	In MDD: 28 prescribed psychotropic medications<br>In Control: 1 rescribed psychotropic medications	PROMIS Depression Score > 21, DSM-5	PROMIS Depression Score	25.0 (6.9)<br>9.3 (1.4)	Fecal samples	−80°C	16S rRNA gene sequencing-Illumina MiSeq platform	V4	MDD and Controls	LEfSe: $p < 0.05$ and LDA > 2.0
Liu T et al. 2020[36]	Chinese	Cross-sectional associations	IBS-D (N=84)<br>Control (N=46)	41.76 (11.57)<br>38.30 (13.13)	46/24<br>25/21	23.36 (3.49)<br>23.63 (3.76)	IBS-D	_	HAMD/SDS	HAMD	13.23 (5.15)/49.89 (9.59)<br>NA/NA	Fecal samples	−80°C	16S rRNA gene sequencing-Illumina MiSeq platform	V3-V4	Cross-sectional associations with depression symptom measures	Spearman’s correlation coefficient
Liu Y et al. 2016[37]	Chinese	Case-control	IBS-D (N=40)<br>Comorbid IBS-D and MDD (N=25)<br>MDD (N=15)<br>Control (N=20)	38.5(13.6)<br>39.0(13.9)<br>44.8(14.9)<br>43.9(11.2)	28/12<br>14/11<br>4/11<br>7/13	22.6(3.1)<br>22.0(3.8)<br>22.0(3.2)<br>24.6(2.2)	IBS-D	NA	DSM-IV MINI	SDS	Data dispalyed in histogram	Fecal samples	−80°C	16s rRNA gene sequencing-Roche 454 sequencing	V1-V3	Depression and Controls	Wilcoxon rank-sum test with q value was used for correction: $q < 0.05$

Madan A et al. 2020[38]	American	Cross-sectional associations	Depression(N=111)	35.7 (13.8)	51/60	_	_	_	SCID-I/II	PHQ-9	_	Fecal samples	−80°C	16S rRNA gene sequencing-Illumina MiSeq platform<br><br>Metagenomic shotgun sequencing-Illumina sequencers	V4	Cross-sectional associations with symptom severity	LEfSe: $p < 0.05$ and LDA > 2.0
Mason BL et al. 2020[39]	American	Case-control	Comorbid MDD and anxiety (N=38)<br>Anxiety (N=8)<br>MDD (N=14)<br>Control (N=10)	39.2(10.6)<br>40.0(13.7)<br>41.9(12.0)<br>33.0(8.4)	7/31<br>0/8<br>3/11<br>4/6	30.4(5.8)<br>33.3(9.2)<br>31.0(5.8)<br>25.6(3.5)	Anxiety	In patients: 59 any medication; 30 Antidepressants; 15 Anti-anxiety medications	DSM-IV Axis I Disorders (SCID)	QIDS-SR	15.2(3.7)<br>10.9(3.9)<br>15.6(3.2)<br>1.3(0.9)	Fecal samples	−80°C	16s rRNA gene sequencing-Roche 454 Titanium platform<br><br>16S rRNA gene qPCR for Eubacteria, Enterobacteriaceae, Eubacterium rectale/Clostridium group (Clostridial cluster XIVa), Lactobacillus/Enterococcus, Bacteroides, Clostridium leptum group (Clostridial cluster IV)	V4<br>_	MDD and Controls	Kruskal–Wallis one-way ANOVA: $p < 0.05$<br>Within these clusters, p-values were adjusted to control the false discovery rate using the Benjamini and Hochberg method
Medina-Rodriguez EM et al. 2020[40]	American	Case-control	MDD (N=10)<br>Control (N=10)	47.2(12.8)<br>39.6(13.4)	2/8<br>2/8	33.9(6.7)<br>28.7(7.1)	In MDD: majority of patients had marked or fully expressed generalized anxiety disorder or other anxiety disorders; 2 Mania or hypomania	_	QIDS≥13	QIDS	15.0(1.8)<br>1.7(1.5)	Fecal samples	−80°C	qPCR	_	MDD and Controls	Mann Whitney U test: $p < 0.05$
Minichino A et al. 2021[41]	British	Cross-sectional associations	Depression-anhedonia/amotivation (N=786)	65.2 (7.6)	52/734	_	218 Obesity	22 Antidepressants	_	_	_	Fecal samples	−80°C	16S rRNA gene sequencing-Illumina MiSeq platform	V4	Cross-sectional associations with depression symptom (anhedonia/amotivation)	Pearson's correlation analyses
Naseribafrouei A et al. 2014[42]	Norwegian	Case-control	Depression(N=37)<br>Control (N=18)	49.2(13.9)<br>46.1(13.9)	17/20<br>7/11	25.9(4.2)<br>24.7(3.3)	NA	Depression medication, Blood pressure medication	ICD-10	MADRS	26.3(7.6)<br>7.2(4.8)	Fecal samples	−70°C	16S rRNA gene sequencing-Illumina MiSeq platform	Unspecified	Depression and Controls	PLS-DA
Pérez-Santiago J et al. 2021[43]	American	Case-control	MDD (N=66)<br>Control (N=61)	54(48-62)<br>54(48-62)	_	_	In MDD: 56 HIV<br>In Controls: 36 HIV	_	DSM-IV	_	_	Fecal samples	_	16S rRNA gene sequencing-Illumina MiSeq platform	V3–V4	MDD and Controls	Mann-Whitney or t test: $p < 0.05$
Qin Q et al. 2021[44]	Chinese	Case-control	Depression (N=60)<br>Control (N=60)	19.30(0.05)<br>19.85(0.11)	30/30<br>30/30	_	Test anxiety	NA	_	HAMD-17	10.95(3.95)<br>1.92(1.49)	Fecal samples	−80°C	16S rRNA gene sequencing-Illumina HiSeq 2000 platform	V4	Depression with test anxiety and Controls	One-way ANOVA followed by Tukey's multiple comparison test: $p < 0.05$
Ramirez-Carrillo E et al. 2020[45]	Mexican	Cross-sectional associations	Depression (N=34 for adults, N=29 for children)	30.48(7.79) for adults<br>7.6(1.8) for children	16/18 for adults<br>13/16 for children	_	_	_	_	_	_	Fecal samples	−80°C	16S rRNA gene sequencing-Illumina MiSeq platform	V4	Cross-sectional associations with depression symptom	Phyloseq, vegan and ggplot2 packages in R
Rhee SJ et al. 2020[46]	Koreans	Case-control	BD (N=42)<br>MDD (N=30)<br>Control (N=36)	34.2(10.8)<br>46.2(9.7)<br>43.0(5.6)	15/27<br>5/25<br>9/27	24.3(4.0)<br>24.4(4.3)<br>23.9(3.9)	NA	In BD: 6 Antidepressant; 32 Anticonvulsant or Lithium; 33 Antipsychotics<br>In MDD: 26 Antidepressant; 1 Anticonvulsant or Lithium; 13 Antipsychotics	DSM-IV, DSM-5, MINI	HAMD-17	6.21(4.80)<br>6.07(5.43)<br>_	Serum samples	−80°C	16S rRNA gene sequencing-Illumina MiSeq System	V3–V4	MDD and Controls	ANCOM

Rhee SJ et al. 2021[47]	Koreans	Cross-sectional associations	Depression (N=69)	39.6(12.0)	18/51	24.2(4.1)	29 MDD patients, 40 BD patients	31 Antidepressant; 33 Anticonvulsant or lithium; 45 Antipsychotics	DSM-IV, DSM-5, MINI	HAMD-17	6.13(5.08)	Serum	−80◦C	16S rRNA gene sequencing-Illumina MiSeq platform	V3-V4	Cross-sectional associations with depression symptom measures	Multivariate association with linear models (MaAsLin2)
Rong H et al. 2019[48]	Chinese	Case-control	BD (N=30)<br>MDD (N=31)<br>Control (N=30)	38.40 (8.33)<br>41.58(10.40)<br>39.47(10.22)	15/15<br>9/22<br>14/16	21.92 (2.22)<br>21.46(2.3)<br>21.97(3.18)	NA	In MDD: 13 SSRIs; 8 SNRIs; 6 Other antidepressants<br>In BPD: 11 SSRIs;3 SNRIs;1 Other antidepressants; 7 Atypical antipsychotics	DSM-5	HAMD-17	20.37 (3.41)<br>20.23(3.11)<br>_	Fecal samples	−80◦C	Shotgun metagenomic-Illumina Hiseq2500 sequencer	_	MDD and Controls	Wilcoxon rank-sum test with FDR multiple testing correction: $p < 0.05$
Shen Y et al. 2021[49]	Chinese	Case-control	MDD (N=30)<br>Control (N=30)	44.83(11.00)<br>43.97(10.57)	13/17<br>15/15	23.99(2.05)<br>23.83(2.08)	NA	NA	MINI<br>HAMD-17 ≥24	HAMD-17	_	Fecal samples	−80◦C	16S rRNA gene sequencing-Illumina HiSeq 2500 platform	V3-V4	MDD and Controls	Metastats (T test): $p < 0.05$
Simpson CA et al. 2020[50]	Australian	Case-control	high-depression(N=33)<br>low-depression(N=33)	16.84(1.11)<br>17.02(0.89)	7/26<br>13/20	_	Anxiety	NA	CESD	CESD	20.14(9.78)<br>10.59(5.75)	Saliva sample	−30◦C	16S rRNA gene sequencing-Ion Torrent Personal Genome Machine	V4	High-depression and low-depression	Multivariate association with linear model analysis (MaAsLin2): p-value < 0.05 and FDR q-value < 0.25
Stevens BR et al. 2021[51]	American	Case-control	Hypertension (N=18)<br>MDD (N=7)<br>MDD with Hypertension (N=8)<br>Control (N=21)	59.9(17.6)<br>63.8(6.2)<br>67.0(10.7)<br>53.0(14.8)	8/10<br>3/4<br>3/5<br>8/13	37.5(13.4)<br>27.3(5.9)<br>34.2(10.5)<br>30.7(7.0)	In HTN: 8 Diabetes; 7 Chronic kidney disease; 2 Peripheral artery disease; 1 Stroke/Transient ischemia attack; 3 Atrial fibrillation; 1 Heart failure<br>In MDD: 2 Diabetes; 2 Stroke/Transient ischemia attack<br>In DEP plus HTN: 2 Diabetes; 3 Chronic kidney disease<br>In Controls: 9 Diabetes; 2 Chronic kidney disease; 2 Heart failure	Unknown	DSM-5	_	_	Fecal samples	−80◦C	whole metagenome shotgun sequencing (WMGS)-Illumina HiSeq4000	NA	Depression only and reference subjects	Pearson correlation heatmaps were generated based on relative Rho magnitudes
Stevens BR et al. 2020[52]	American	Case-control	MDD (N=20)<br>Control (N=20)	mean: 34	10/10<br>6/14	_	NA	In MDD: 15 Antidepressant	DSM-IV	_	_	Fecal samples	−80◦C	16S rRNA gene sequencing-Illumina MiSeq platform	V3–V4	MDD and Controls	ALDEx2 effect sizes for taxons assigned from ASVs. Displayed cutoffs are effect size >0.5 (NODEP) or <−0.5 (DEPR)
Strandwitz P et al. 2019[53]	American	Cross-sectional associations	MDD (N=23)	19-65	8/15	_	NA	NA	DSM-IV-TR	_	_	Fecal samples	−80◦C	16S rRNA gene sequencing-American Gut dataset	V4	Cross-sectional associations with depression symptom measures	Pearson
Szczesniak O et al. 2016[54]	Norwegian	Case-control	Depression(N=34)<br>Control (N=17)	49.2(13.9)[d]<br>46.1(13.9)[d]	17/20 [d]<br>7/11[d]	25.9(4.2)[d]<br>24.7(3.3)[d]	NA	Depression medication, Blood pressure medication	ICD-10	MADRS	26.3(7.6)<br>7.2(4.8)	Fecal samples	−70◦C	16S rRNA gene sequencing-Illumina MiSeq platform	Unspecified	As described in Naseribafrouei et al. (2014) Depression and Controls	PLS-DA
Taylor AM et al. 2020[55]	American	Cross-sectional associations	Depression (N=133)	33.4(5.7)	60/73	30.9(6.7)	NA	NA	DASS-42	DASS-42-Depression Scale	3.4(4.6)	Fecal samples	−80 ℃	16S rRNA gene sequencing-Illumina MiSeq2000 platform	V4	Cross-sectional associations with depression symptom measures	Linear mixed modeling
Taylor BC et al. 2020[56]	American	Case-control	MDD (N=34)<br>Control (N=14)	53.8(9.1)[e]	41/7 [e]	_	HIV and HCV infections	_	DSM-IV, BDI-II≥14	BDI-II	10.9(10.7)	Fecal samples	_	16S rRNA gene sequencing-Illumina MiSeq platform	V4	MDD and Controls (Coinfected)	Kruskal-Wallis test: $p < 0.05$

Valles-Colomer M et al. 2019[57]	Belgian<br><br><br><br>Netherlander	Case-control	FGFP cohort:<br>Depression (N=121)<br>Control (N=933)<br><br>Validation LLD cohort:<br>Depression(N=115)<br>Contros (N=948)	50.9<br><br><br><br>44.9	479/575<br><br><br><br>448/615	24.9<br><br><br><br>25.3	Unspecified	In FGFP cohort: 52 Antidepressant treatment<br><br><br>In LLD cohort: 29 Antidepressant treatment	Unspecified	_	_	Fecal samples	−80°C	16S rRNA gene sequencing Illumina HiSeq 2500 System/Illumina MiSeq platform Shotgun metagenomics-Illumina HiSeq2500	V4	Two independent studies (Flemish Gut Flora Project [FGFP] and Dutch LifeLines DEEP [LLD]): Depression and Controls	Generalized linear models (GLMs): FDR <0.1
Wingfield B et al. 2021[58]	English	Case-control	MDD (N=40)<br>Control (N=43)	21.8(5.1)<br>20.4(3.9)	11/29<br>13/30	_	NA	NA	DSM-IV	_	_	Saliva samples	Room temperature	16S rRNA gene sequencing-Illumina MiSeq platform	V3–V4	MDD and Controls	Wald test and Benjamini-Hochberg (FDR) correction: FDR < 0.05
Xu C et al. 2020[59]	Chinese	Case-control	IBS-D (N=22)<br>Comorbid IBS-D and MDD (N=13)<br>MDD (N=15)<br>Control (N=15)	40.7(3.1)<br>43.1(3.6)<br>42.3(3.0)<br>44.8(2.9)	12/10<br>6/7<br>6/9<br>5/10	22.9(0.7)<br>22.7(1.1)<br>21.3(0.7)<br>24.1(0.7)	IBS-D	NA	DSM-IV MINI	SDS	39.5(2.1)<br>64.5(3.6)<br>58.2(2.3)<br>28.7(1.4)	Fecal samples	−80°C	Metagenomic Sequencing-Illumina HiSeq™ platform	NA	MDD and Controls	One-way ANOVA: $p < 0.05$
Yang J et al. 2020[60]	Chinese	Case-control	MDD (N=118)<br>Control (N=118)	27.19(4.71)<br>26.86(5.24)	51/67<br>51/67	22.50(3.56)<br>22.09(3.33)	NA	NA	DSM-IV MINI	HAMD-17	22.03(5.08)<br>NA	Fecal samples	_	Metagenomic-Illumina NovaSeq	NA	MDD and Controls	Lefse: LDA > 2.5
Yang Y et al. 2021[61]	Chinese	Cross-sectional associations	METH abuser (N=16)<br>Control (N=14)	36(7.8)<br>36(9.4)	16/0<br>14/0	_	NA	Methamphetamine	DSM-5	PANSS	_	Fecal samples	−80°C	16S rRNA gene sequencing-Illumina NovaSeq platform	V4	Cross-sectional associations with depression symptom measures	Spearman's rank-correlation analysis: $p < 0.05$
Ye X et al. 2021[62]	Chinese	Case-control	MDD (N=26)<br>Control (N=28)	26.04(7.83)<br>26.04(7.83)	5/21<br>7/21	19.78(2.12)<br>21.59(3.48)	NA	NA	DSM-IV<br>HAMD-17≥24	HAMD-17	27.92(2.77)<br>1.29(1.3)	Fecal samples	−80°C	16S rRNA gene sequencing-Illumina HiSeq 2500 platform	V3-V4	MDD and Controls (baseline)	LEfSe: $p < 0.05$ and LDA > 3.5
Zhang Q et al. 2021[63]	Chinese	Case-control	MDD (N=36)<br>Control (N=45)	36.81(13.52)<br>39.29(11.44)	12/15<br>19/26	24.47(4.16)<br>23.94(3.05)	NA	NA	ICD-10<br>HAM-D >17	HAMD-17	5.36(6.24)<br>0.51(1.01)	Fecal samples	−80°C	16S rRNA gene sequencing-Illumina NovaSeq 6000 platform	V4–V5	MDD and Controls	LEfSe: $p < 0.05$ and LDA ≥ 2.0
Zhao H et al. 2021[64]	Chinese	Case-control	MDD (N=24)<br>Control (N=26)	29.96(8.554)<br>31.31(9.707)	7/17<br>8/18	_	NA	NA	DSM-5, HAMD-17≥17	HAMD-17	24.83(3.116)<br>2.12(2.776)	Fecal samples	−80°C	Metagenomics sequencing-Illumina Novaseq 6000 platform	_	MDD and Controls	LEfSe: $p < 0.05$ and LDA > 2.0 meta stat analysis
Zheng P et al. 2016[65]	Chinese	Case-control	MDD (N=58)<br>Control (N=63)	40.6(11.7)<br>41.8(12.3)	22/36<br>23/40	22.0(2.4)<br>22.6(2.5)	NA	In MDD: 19 Antidepressants (e.g., Citalopram, Venlafaxine)	DSM-IV-TR	HAMD-17	22.8(4.4)<br>0.3(0.7)	Fecal samples	−80 °C	16S rRNA gene sequencing-Roche 454 sequencing	V3-V5	MDD and Controls	Random Forests
Zheng P et al. 2020[66]	Chinese	Case-control	MDD (N=122)<br>BD (N=169)<br>Control (N=171)	26.54(4.07)<br>25.59(8.41)<br>26.85(5.48)	45/77<br>85/84<br>71/100	22.41(3.63)<br>21.77(3.68)<br>22.07(3.38)	NA	NA	DSM-IV	HAMD	22.65(5.50)<br>NA	Fecal samples	−80°C	16S rRNA gene sequencing-Illumina MiSeq platform	V3-V4	MDD and Controls	Kruskal-Wallis test: $p < 0.05$<br>Lefse: fold change >2.0 and LDA > 2.5
Zheng S et al. 2021[67]	Chinese	Case-control	Depression (N=30)<br>Control (N=30)	30.80 (10.85)<br>33.37 (7.02)	12/18<br>13/17	_	NA	NA	ICD-10	HAMD-24	20.17 (7.92)<br>0.30 (0.79)	Fecal samples	−80°C	16S rRNA gene sequencing-Illumina MiSeq platform	Unspecified	Depression and Controls	LEfSe: $p < 0.05$ and LDA > 2.0 and other methods unspecified
Zhou Y et al. 2020[68]	Chinese	Case-control	PPD (N=28)<br>Control (N=16)	33.64(4.27)^{f}<br>32.57(3.98)^{f}	0/28<br>0/16	21.50 (2.87)^{f}<br>20.90 (2.24)^{f}	NA	NA	DSM-IV<br>HAMD-17≥7	HAMD-17	13.46 (3.51)#<br>3.83(1.98)#	Fecal samples	−80°C	16S rRNA gene sequencing-Illumina MiSeq platform	V4	PPD and Controls	LEfSe: $p < 0.05$ and LDA > 2.0
Zhu J et al. 2021[69]	Chinese	Case-control	Depression (N=23)<br>Control (N=46)	55.30(8.20)<br>55.63(7.78)	13/10<br>21/25	24.43(3.21)<br>25.51(3.47)	Anxiety	_	PHQ-9≥5	PHQ-9	_	Fecal samples	−80°C	16S rRNA gene sequencing-Illumina MiniSeq platform	V4	Depression with anxiety and Controls	LEfSe: $p < 0.05$ and LDA > 2.032
Zhuang Z et al. 2020[70]	European	Cross-sectional associations	MDD (N=135458)<br>Control (N=344901)	_	_	_	_	_	DSM-IV,DSM-III, ICD-9 or ICD-10	_	_	Fecal samples	_	Microbiota-related gene set enrichment analysis (GSEA)-	_	Cross-sectional associations between MDD and gut	Unspecified

														Illumina MiSeq/Illumina HiSeq2000/Illumina HiSeq		microbiota using published GWAS data	

Note:

[a]Data from 56 OI patients with or without depression (Ref.22)

[b]Data from all 2737 individuals (Ref.23)

[c]Data from all 17 patients with irritable bowel syndrome (Ref.29)

[d]Data from 38 patients with depression and 18 controls (Ref.54)

[e]Data from total 48 patients (Ref.56)

[f]Data from 39 patients with postpartum depressive disorder and 18 controls (Ref.68)

References

1. E. Aizawa, H. Tsuji, T. Asahara, T. Takahashi, T. Teraishi, S. Yoshida, M. Ota, N. Koga, K. Hattori and H. Kunugi, *J Affect Disord* **2016**, *202*, 254-257.

2. S. Bai, J. Xie, H. Bai, T. Tian, T. Zou and J.-J. Chen, *J Inflamm Res* **2021**, *14*, 3755-3766.

3. S. Bai, H. Bai, D. Li, Q. Zhong, J. Xie and J.-J. Chen, *Front Cell Infect Microbiol* **2022**, *12*, 831186.

4. J. R. Caso, K. S. MacDowell, A. González-Pinto, S. García, J. de Diego-Adeliño, M. Carceller-Sindreu, F. Sarramea, J. Caballero-Villarraso, P. Gracia-García, C. De la Cámara, L. Agüera, M. L. Gómez-Lus, C. Alba, J. M. Rodríguez and J. C. Leza, *Transl Psychiatry* **2021**, *11*, 645.

5. B. Chahwan, S. Kwan, A. Isik, S. van Hemert, C. Burke and L. Roberts, *J Affect Disord* **2019**, *253*, 317-326.

6. D.-L. Chen, Y.-C. Dai, L. Zheng, Y.-L. Chen, Y.-L. Zhang and Z.-P. Tang, *Medicine (Baltimore)* **2021**, *100*, e24845.

7. J.-J. Chen, P. Zheng, Y.-Y. Liu, X.-G. Zhong, H.-Y. Wang, Y.-J. Guo and P. Xie, *Neuropsychiatr Dis Treat* **2018**, *14*, 647-655.

8. J.-J. Chen, S. He, L. Fang, B. Wang, S.-J. Bai, J. Xie, C.-J. Zhou, W. Wang and P. Xie, *Aging (Albany NY)* **2020**, *12*, 2764-2776.

9. T. Chen, R. Wang, Z. Duan, X. Yuan, Y. Ding, Z. Feng, F. Bu, L. Liu, Q. Wang, J. Zhou, L. Zhu, Q. Ni, G. Shi and Y. Chen, *Front Cell Infect Microbiol* **2021**, *11*, 723856.

10. Y. Chen, P. Meng, S. Cheng, Y. Jia, Y. Wen, X. Yang, Y. Yao, C. Pan, C. e. Li, H. Zhang, J. Zhang, Z. Zhang and F. Zhang, *Mol Brain* **2021**, *14*, 133.

11. Y.-H. Chen, F. Xue, S.-F. Yu, X.-S. Li, L. Liu, Y.-Y. Jia, W.-J. Yan, Q.-R. Tan, H.-N. Wang and Z.-W. Peng, *J Affect Disord* **2021**, *282*, 391-400.

12. Z. Chen, J. Li, S. Gui, C. Zhou, J. Chen, C. Yang, Z. Hu, H. Wang, X. Zhong, L. Zeng, K. Chen, P. Li and P. Xie, *Neuroreport* **2018**, *29*, 417-425.

13. S. Cheng, B. Han, M. Ding, Y. Wen, M. Ma, L. Zhang, X. Qi, B. Cheng, P. Li, O. P. Kafle, X. Liang, L. Liu, Y. Du, Y. Zhao and F. Zhang, *Brief Bioinform* **2020**, *21*, 1016-1022.

14. Y.-C. E. Chung, H.-C. Chen, H.-C. L. Chou, I. M. Chen, M.-S. Lee, L.-C. Chuang, Y.-W. Liu, M.-L. Lu, C.-H. Chen, C.-S. Wu, M.-C. Huang, S.-C. Liao, Y.-H. Ni, M.-S. Lai, W.-L. Shih and P.-H. Kuo, *J Psychiatr Res* **2019**, *111*, 74-82.

15. D. Ciocan, A.-M. Cassard, L. Becquemont, C. Verstuyft, C. S. Voican, K. El Asmar, R. Colle, D. David, S. Trabado, B. Feve, P. Chanson, G. Perlemuter and E. Corruble, *J Psychiatry Neurosci* **2021**, *46*, E358-E368.

16. Z. Dong, X. Shen, Y. Hao, J. Li, H. Li, H. Xu, L. Yin and W. Kuang, *Front Psychiatry* **2021**, *12*, 651536.

17. A. Fontana, M. Manchia, C. Panebianco, P. Paribello, C. Arzedi, E. Cossu, M. Garzilli, M. A. Montis, A. Mura, C. Pisanu, D. Congiu, M. Copetti, F. Pinna, B. Carpiniello, A. Squassina and V. Pazienza, *Biomedicines* **2020**, *8*,

18. N. Heym, B. C. Heasman, K. Hunter, S. R. Blanco, G. Y. Wang, R. Siegert, A. Cleare, G. R. Gibson, V. Kumari and A. L. Sumich, *Psychopharmacology (Berl)* **2019**, *236*, 1459-1470.

19. M. Hoggard, A. Nocera, K. Biswas, M. W. Taylor, R. G. Douglas and B. S. Bleier, *Int Forum Allergy Rhinol* **2018**, *8*, 394-405.

20. Y. Huang, X. Shi, Z. Li, Y. Shen, X. Shi, L. Wang, G. Li, Y. Yuan, J. Wang, Y. Zhang, L. Zhao, M. Zhang, Y. Kang and Y. Liang, *Neuropsychiatr Dis Treat* **2018**, *14*, 3329-3337.

21. Y. Huang, Z. Shen and W. He, *Front Aging Neurosci* **2021**, *13*, 706765.

22. W. Ishii, S. Komine-Aizawa, C. Takano, Y. Fujita, I. Morioka and S. Hayakawa, *Prim Care Companion CNS Disord* **2019**, *21*,

23. M. A. Jackson, S. Verdi, M.-E. Maxan, C. M. Shin, J. Zierer, R. C. E. Bowyer, T. Martin, F. M. K. Williams, C. Menni, J. T. Bell, T. D. Spector and C. J. Steves, *Nat Commun* **2018**, *9*, 2655.

24. H. Jiang, Z. Ling, Y. Zhang, H. Mao, Z. Ma, Y. Yin, W. Wang, W. Tang, Z. Tan, J. Shi, L. Li and B. Ruan, *Brain Behav Immun* **2015**, *48*, 186-194.

25. H.-Y. Jiang, L.-Y. Pan, X. Zhang, Z. Zhang, Y.-Y. Zhou and B. Ruan, *Brain Behav* **2020**, *10*, e01677.

26. Y. Kang, Y. Yang, J. Wang, Y. Ma, H. Cheng and D. Wan, *J Coll Physicians Surg Pak* **2021**, *31*, 1224-1227.

27. J. R. Kelly, Y. Borre, C. O' Brien, E. Patterson, S. El Aidy, J. Deane, P. J. Kennedy, S. Beers, K. Scott, G. Moloney, A. E. Hoban, L. Scott, P. Fitzgerald, P. Ross, C. Stanton, G. Clarke, J. F. Cryan and T. G. Dinan, *J Psychiatr Res* **2016**, *82*, 109-118.

28. S. C. Kleiman, H. J. Watson, E. C. Bulik-Sullivan, E. Y. Huh, L. M. Tarantino, C. M. Bulik and I. M. Carroll, *Psychosom Med* **2015**, *77*, 969-981.

29. S. Kurokawa, T. Kishimoto, S. Mizuno, T. Masaoka, M. Naganuma, K.-C. Liang, M. Kitazawa, M. Nakashima, C. Shindo, W. Suda, M. Hattori, T. Kanai and M. Mimura, *J Affect Disord* **2018**, *235*, 506-512.

30. W.-T. Lai, W.-F. Deng, S.-X. Xu, J. Zhao, D. Xu, Y.-H. Liu, Y.-Y. Guo, M.-B. Wang, F.-S. He, S.-W. Ye, Q.-F. Yang, T.-B. Liu, Y.-L. Zhang, S. Wang, M.-Z. Li, Y.-J. Yang, X.-H. Xie and H. Rong, *Psychol Med* **2021**, *51*,

31. P. Lin, B. Ding, C. Feng, S. Yin, T. Zhang, X. Qi, H. Lv, X. Guo, K. Dong, Y. Zhu and Q. Li, *J Affect Disord* **2017**, *207*, 300-304.

32. Y. Ling, Q. Gu, J. Zhang, T. Gong, X. Weng, J. Liu and J. Sun, *J Alzheimers Dis* **2020**, *77*, 1595-1608.

33. P. Liśkiewicz, M. Kaczmarczyk, B. Misiak, M. Wroński, A. Bąba-Kubiś, K. Skonieczna-Żydecka, W. Marlicz, P. Bieńkowski, A. Misera, J. Pełka-Wysiecka, J. Kucharska-Mazur, A. Konopka, I. Łoniewski and J. Samochowiec, *Prog Neuropsychopharmacol Biol Psychiatry* **2021**, *106*, 110076.

34. P. Liu, M. Gao, Z. Liu, Y. Zhang, H. Tu, L. Lei, P. Wu, A. Zhang, C. Yang, G. Li, N. Sun and K. Zhang, *Front Neurosci* **2021**, *15*, 800764.

35. R. T. Liu, A. D. Rowan-Nash, A. E. Sheehan, R. F. L. Walsh, C. M. Sanzari, B. J. Korry and P. Belenky, *Brain Behav Immun* **2020**, *88*, 308-324.

36. T. Liu, X. Gu, L.-X. Li, M. Li, B. Li, X. Cui and X.-L. Zuo, *BMC Microbiol* **2020**, *20*, 168.

37. Y. Liu, L. Zhang, X. Wang, Z. Wang, J. Zhang, R. Jiang, X. Wang, K. Wang, Z. Liu, Z. Xia, Z. Xu, Y. Nie, X. Lv, X. Wu, H. Zhu and L. Duan, *Clin Gastroenterol Hepatol* **2016**, *14*,

38. A. Madan, D. Thompson, J. C. Fowler, N. J. Ajami, R. Salas, B. C. Frueh, M. R. Bradshaw, B. L. Weinstein, J. M. Oldham and J. F. Petrosino, *J Affect Disord* **2020**, *264*,

39. B. L. Mason, Q. Li, A. Minhajuddin, A. H. Czysz, L. A. Coughlin, S. K. Hussain, A. Y. Koh and M. H. Trivedi, *J Affect Disord* **2020**, *266*, 394-401.

40. E. M. Medina-Rodriguez, D. Madorma, G. O'Connor, B. L. Mason, D. Han, S. K. Deo, M. Oppenheimer, C. B. Nemeroff, M. H. Trivedi, S. Daunert and E. Beurel, *Am J Psychiatry* **2020**, *177*, 974-990.

41. A. Minichino, M. A. Jackson, M. Francesconi, C. J. Steves, C. Menni, P. W. J. Burnet and B. R. Lennox, *Mol Psychiatry* **2021**, *26*, 6269-6276.

42. A. Naseribafrouei, K. Hestad, E. Avershina, M. Sekelja, A. Linløkken, R. Wilson and K. Rudi, *Neurogastroenterol Motil* **2014**, *26*, 1155-1162.

43. J. Pérez-Santiago, M. J. Marquine, D. Cookson, R. Giraud-Colón, R. K. Heaton, I. Grant, R. J. Ellis, S. L. Letendre and S. N. Peterson, *J Neurovirol* **2021**, *27*, 228-238.

44. Q. Qin, H. Liu, Y. Yang, Y. Wang, C. Xia, P. Tian, J. Wei, S. Li and T. Chen, *Dis Markers* **2021**, *2021*, 5597401.

45. E. Ramírez-Carrillo, O. Gaona, J. Nieto, A. Sánchez-Quinto, D. Cerqueda-García, L. I. Falcón, O. A. Rojas-Ramos and I. González-Santoyo, *Sci Rep* **2020**, *10*, 3680.

46. S. J. Rhee, H. Kim, Y. Lee, H. J. Lee, C. H. K. Park, J. Yang, Y.-K. Kim, S. Kym and Y. M. Ahn, *J Psychiatr Res* **2020**, *123*, 31-38.

47. S. J. Rhee, H. Kim, Y. Lee, H. J. Lee, C. H. K. Park, J. Yang, Y.-K. Kim and Y. M. Ahn, *Sci Rep* **2021**, *11*, 13987.

48. H. Rong, X.-H. Xie, J. Zhao, W.-T. Lai, M.-B. Wang, D. Xu, Y.-H. Liu, Y.-Y. Guo, S.-X. Xu, W.-F. Deng, Q.-F. Yang, L. Xiao, Y.-L. Zhang, F.-S. He, S. Wang and T.-B. Liu, *J Psychiatr Res* **2019**, *113*, 90-99.

49. Y. Shen, X. Yang, G. Li, J. Gao and Y. Liang, *Sci Rep* **2021**, *11*, 14918.

50. C. A. Simpson, C. Adler, M. R. du Plessis, E. R. Landau, S. G. Dashper, E. C. Reynolds, O. S. Schwartz and J. G. Simmons, *Physiol Behav* **2020**, *226*, 113126.

51. B. R. Stevens, C. J. Pepine, E. M. Richards, S. Kim and M. K. Raizada, *Am Heart J* **2021**, *239*, 27-37.

52. B. R. Stevens, L. Roesch, P. Thiago, J. T. Russell, C. J. Pepine, R. C. Holbert, M. K. Raizada and E. W. Triplett, *Mol Psychiatry* **2020**, *26*, 4277-4287.

53. P. Strandwitz, K. H. Kim, D. Terekhova, J. K. Liu, A. Sharma, J. Levering, D. McDonald, D. Dietrich, T. R. Ramadhar, A. Lekbua, N. Mroue, C. Liston, E. J. Stewart, M. J. Dubin, K. Zengler, R. Knight, J. A. Gilbert, J. Clardy and K. Lewis, *Nat Microbiol* **2019**, *4*, 396-403.

54. O. Szczesniak, K. A. Hestad, J. F. Hanssen and K. Rudi, *Nutr Neurosci* **2016**, *19*, 279-283.

55. A. M. Taylor, S. V. Thompson, C. G. Edwards, S. M. A. Musaad, N. A. Khan and H. D. Holscher, *Nutr Neurosci* **2020**, *23*, 983-992.

56. B. C. Taylor, K. C. Weldon, R. J. Ellis, D. Franklin, T. Groth, E. C. Gentry, A. Tripathi, D. McDonald, G. Humphrey, M. Bryant, J. Toronczak, T. Schwartz, M. F. Oliveira, R. Heaton, I. Grant, S. Gianella, S. Letendre, A. Swafford, P. C. Dorrestein and R. Knight, *mSystems* **2020**, *5*,

57. M. Valles-Colomer, G. Falony, Y. Darzi, E. F. Tigchelaar, J. Wang, R. Y. Tito, C. Schiweck, A. Kurilshikov, M. Joossens, C. Wijmenga, S. Claes, L. Van Oudenhove, A. Zhernakova, S. Vieira-Silva and J. Raes, *Nat Microbiol* **2019**, *4*, 623-632.

58. B. Wingfield, C. Lapsley, A. McDowell, G. Miliotis, M. McLafferty, S. M. O'Neill, S. Coleman, T. M. McGinnity, A. J. Bjourson and E. K. Murray, *Sci Rep* **2021**, *11*, 15009.

59. C. Xu, Q. Jia, L. Zhang, Z. Wang, S. Zhu, X. Wang, Y. Liu, M. Li, J. Zhang, X. Wang, J. Zhang, Q. Sun, K. Wang, H. Zhu and L. Duan, *Front Cell Infect Microbiol* **2020**, *10*, 580980.

60. J. Yang, P. Zheng, Y. Li, J. Wu, X. Tan, J. Zhou, Z. Sun, X. Chen, G. Zhang, H. Zhang, Y. Huang, T. Chai, J. Duan, W. Liang, B. Yin, J. Lai, T. Huang, Y. Du, P. Zhang, J. Jiang, C. Xi, L. Wu, J. Lu, T. Mou, Y. Xu, S. W. Perry, M.-L. Wong, J. Licinio, S. Hu, G. Wang and P. Xie, *Sci Adv* **2020**, *6*,

61. Y. Yang, X. Yu, X. Liu, G. Liu, K. Zeng and G. Wang, *Sci Rep* **2021**, *11*, 18178.

62. X. Ye, D. Wang, H. Zhu, D. Wang, J. Li, Y. Tang and J. Wu, *Front Psychiatry* **2021**, *12*, 641491.

63. Q. Zhang, Y. Yun, H. An, W. Zhao, T. Ma, Z. Wang and F. Yang, *Front Psychiatry* **2021**, *12*, 645045.

64. H. Zhao, K. Jin, C. Jiang, F. Pan, J. Wu, H. Luan, Z. Zhao, J. Chen, T. Mou, Z. Wang, J. Lu, S. Lu, S. Hu, Y. Xu and M. Huang, *Transl Psychiatry* **2022**, *12*, 8.

65. P. Zheng, B. Zeng, C. Zhou, M. Liu, Z. Fang, X. Xu, L. Zeng, J. Chen, S. Fan, X. Du, X. Zhang, D. Yang, Y. Yang, H. Meng, W. Li, N. D. Melgiri, J. Licinio, H. Wei and P. Xie, *Mol Psychiatry* **2016**, *21*, 786-796.

66. P. Zheng, J. Yang, Y. Li, J. Wu, W. Liang, B. Yin, X. Tan, Y. Huang, T. Chai, H. Zhang, J. Duan, J. Zhou, Z. Sun, X. Chen, S. Marwari, J. Lai, T. Huang, Y. Du, P. Zhang, S. W. Perry, M.-L. Wong, J. Licinio, S. Hu, P. Xie and G. Wang, *Adv Sci (Weinh)* **2020**, *7*, 1902862.

67. S. Zheng, Y. Zhu, W. Wu, Q. Zhang, Y. Wang, Z. Wang and F. Yang, *Brain Behav* **2021**, *11*, e02036.

68. Y. Zhou, C. Chen, H. Yu and Z. Yang, *Front Cell Infect Microbiol* **2020**, *10*, 567268.

69. J. Zhu, M. Li, D. Shao, S. Ma and W. Wei, *Front Psychiatry* **2021**, *12*, 757139.

70. Z. Zhuang, R. Yang, W. Wang, L. Qi and T. Huang, *J Neuroinflammation* **2020**, *17*, 288.

Table S2. Characteristics of studies investigating gut microbiota composition in animal models of depression.

Study	Object	Country	Study design	housed	Depression model	groups	Age	Sex	Definition of depression-like behaviors	Time point of sample collection	Sample	Storage	Gut microbiome estimation	Sequencing region	Microbial biomarkers from comparisons	Microbial biomarkers selection
Abildgaard A et al. 2021[1]	Flinders sensitive line rats<br>Flinders resistant line rats	Denmark	case-control	pair-housed	FSL-depression	FSL (N=12)<br>FSL-HFD (N=12)<br>FSL+Probiotics (N=11)<br>FSL-HFD+Probiotics (N=11)<br>FRL-Control (N=12)	5-week-old	male	FST: No data (immobility)<br>OFT: No data (locomotor activity)	Fresh faecal samples were collected at study initiation and study end	Fecal samples	−80 ℃	16S rRNA gene sequencing-Illumina MiSeq platform	V4	FSL-depression and FRL-control	Two-way ANOVA followed by Bonferroni correction: p < 0.05
Aitbali Y et al. 2018[2]	Swiss mice	Morocco	case-control	_	GBH-depression	Acute GBH low-dose (N=6)<br>Subchronic GBH low-dose (N=6)<br>Chronic GBH low-dose (N=6)<br>Acute GBH high-dose (N=6)<br>Subchronic GBH high-dose (N=6)<br>Chronic GBH high-dose (N=6)<br>Acute control (N=6)<br>Subchronic control (N=6)<br>Chronic control (N=6)	1-month-old	male	OFT: center time↓<br>EPM: anxiety index↑<br>TST: immobility time↑<br>Splash test: grooming time↓	After subchronic and chronic groups were treated daily for 6 and 12 weeks	Intestinal samples	_	Bacterial strains-dilution/spreading method	_	GBH-depression and control	Two-way ANOVA followed by Holm-Sidak post hoc test: p < 0.05
Amini-Khoei H et al. 2019[3]	NMRI mice	Iran	case-control	4 mice/cage	MS-depression	MS (N=9-12)<br>Adrenalectomized MS (N=9-12)<br>Control (N=9-12)<br>Adrenalectomized control (N=9-12)	pnd 50-52	male	FST: immobility time↑<br>Splash test: grooming activity time↓<br>OFT: horizontal activity and rearing↑<br>EPM: open arm time and entries↓	Fresh feces were collected from male mice at PND52 after having carried out valid behavioral tests	Colon contents	−80◦C	Real-time RT-PCR	_	MS-depression and control	Two-way ANOVA followed by Bonferroni post hoc test: p < 0.05
An Q et al. 2020[4]	C57BL/6J mice	China	case-control	5 mice/cage	CUMS-depression	CUMS (N=12)<br>CUMS+AGO (N=12)<br>CUMS+NMDEA low-dosage (N=12)<br>CUMS+NMDEA high-dosage (N=12)<br>Control (N=12)	8-week-old	male	Body weight:↓<br>SPT: sucrose preference↓<br>FST: immobility time↑<br>TST: immobility time↑	After 1 week of acclimatization and 8 weeks of CUMS (the last 4 weeks received drugs treatment)	Fecal samples	−80◦C	16S rRNA gene sequencing-Illumina MiSeq platform	V3-V4	CUMS-depression and control	Mann-Whitney test followed by multiple comparisons using Benjamini & Hochberg's false discovery rate: FDR < 0.05
Arslanova A et al. 2021[5]	mice	Russia	case-control	4-5 mice/cage	Antibiotic-depression	Antibiotic (N=25)<br>Antibiotic+lactobacilli (N=25)<br>Control (N=25)	25-day-old	male	Mortality rate↑<br>Body weight↓<br>OFT: crossing number, defecation, grooming↑; rearing number, head dips, latency to exit from central zone ↓<br>Rotarod test: Latency to falls↓<br>PaGE: time to spent on the grip↓<br>T maze: alternation↓<br>Novel Object Recognition: time index↓	Cecum content samples were collected on the 15th day of the experiment	Caecum contents	−80◦C	16S rRNA gene sequencing-Illumina MiSeq platform	V3-V4	Antibiotic-depression and control	Nonparametric ANOVA Kruskal–Wallis test: p < 0.05
Bharwani A et al. 2017[6]	C57BL/6 mice	Canada	case-control	Single	CSDS-depresstion	CSDS (N=24)<br>CSDS+L. rhamnosus JB-1 (N=17)<br>Control (N=18)<br>Control+L. rhamnosus JB-1 (N=13)	8-week-old	male	SIT: social interaction ratios↓<br>OFT: rearing number↓<br>LDT: light zone entries↓	Fecal pellets were collected before the first defeat session (at 18th day of L. rhamnosus JB-1 treatment), the final defeat session (at the final day of JB-1	Fecal samples	−80◦C	16S rRNA gene sequencing-Illumina MiSeq platform	V3	CSDS-depresstion and control	Kruskal-Wallis one-way ANOVA or the Mann Whitney U test, followed by the Benjamini-Hochberg correction

										treatment), and 3 weeks after the final defeat session						for multiple comparisons (False Discovery Rate < 0.05)
Bridgewater LC et al. 2017[7]	C57BL/6J mice	China	cross-over	4 mice/cage	HFD-depression<br><br>CUMS-depression	HFD (N=34)<br>NCD (N=33)<br><br>HFD+CUMS (N=29)<br>NCD+CUMS (N=29)	6-week-old	male/female	OFT: center distance↓<br>EPM: open arm time and entries↓	Fecal samples were collected for microbiota analysis at day 0 (2 weeks after acclimatization), at day 81 (afer the frst 12 weeks on HFD) and at day 136 (afer CUMS).	Fecal samples	_	16S rRNA gene sequencing-Illumina MiSeq platform	V3-V4	HFD-depression and NCD (only for male)<br><br>post-CUMS-NCD and pre-CUMS-NCD	Random Forest models
Burokas A et al. 2017[8]	C57BL/6J mice	Ireland	case-control	Single	Prebiotics-anti-depression<br><br>CSDS-depression	Study 1<br>Prebiotics FOS (N=10)<br>Prebiotics GOS (N=10)<br>Prebiotics FOS+GOS (N=10)<br>Control (N=10)<br><br>Study 2<br>CSDS (N=9–10)<br>CSDS+FOS+GOS (N=9–10)<br>Control (N=9–10)	7-week-old	male	Study 1<br>FST: immobility time↓<br>TST: immobility time↓<br>OFT: center time↑<br>EPM: open arms entries↑<br><br>Study 2<br>SIT: interaction ratio↓<br>OFT: center entries↓<br>EPM: open arms entries and time↓<br>FST: immobility time↑<br>TST: immobility time↑	Study 1<br>After 10 weeks of prebiotic administration<br><br>Study 2<br>After 6 weeks of prebiotic administration (the first 3 weeks received CSDS)	Cecal contents	−80◦C	16S rRNA gene sequencing-Illumina MiSeq platform	V3-V4	Study 1<br>Prebiotics-anti-depression and control<br><br>Study 2<br>CSDS-depression and control	Nonparametric Kruskal-Wallis and Mann-Whitney or Dunn's tests: p < 0 05
Cao C et al. 2020[9]	ICR mice	China	case-control	_	CUMS-depression	CUMS (N=8)<br>CUMS+D-652 (N=8)<br>CUMS+K-984 (N=8)<br>CUMS+K-1640 (N=8)<br>CUMS+Fluoxetine (N=8)<br>Control (N=8)	20-25 g	male	SPT: sucrose preference↓<br>FST: immobility time↑	After 1 week of acclimatization and 7 weeks of CUMS (the last 7 days received drugs treatment)	Fecal samples	−80 ℃	16S rRNA gene sequencing-Illumina HiSeq 2500 platform	V3-V4	CUMS-depression and control	LefSe (one-way ANOVA followed by a Bonferroni post hoc analysis): p < 0.05
Chakraborti A et al. 2021[10]	C57BL/6J mice	USA	case-control	3 rats/cage	HFCS-MFD-depression	HFCS-MFD (N=12)<br>Control (N=12)	4 weeks old	male	OFT: center time and entries↓<br>EPM: open arms time and entries↓<br>FST: immobility time↑	After 1 week of acclimatization and 16 weeks of a High Fructose Corn Syrup-Moderate Fat Diet (HFCS-MFD) treatment	Caecal content	−80◦C	16S rRNA gene sequencing-Illumina MiSeq platform	V4	HFCS-MFD-depression and control	LEfSe: p < 0.05 and LDA > 2.0
Chen L et al. 2021[11]	C57BL/6J mice	China	case-control	_	LPS-depresstion	LPS (N=20)<br>LPS+NU9056 (N=15)<br>Control (N=8)	12-week-old	Unspecified	Survival rate↓<br>NOR: test preference index↓<br>EPM: open arms time↓<br>TST: immobility duration↑	After 7 days of acclimatization, stool samples were collected 16 h, 1, 3, and 9 days after intraperitoneal injection of LPS	Colon contents	−80◦C	16S rRNA gene sequencing-Illumina NovaSeq platform	Unspecified	LPS-depression and control	One-way ANOVA: p < 0.05
Chen P et al. 2019[12]	BALB/c mice	China	case-control	_	UCMS-depresstion	UCMS (N=10)<br>UCMS+GPS (N=10)<br>UCMS+Paroxetine (N=10)<br>Control (N=10)<br><br>Abx-UCMS-FMT (N=8)<br>Abx-GPS-FMT (N=8)	3-4-week-old	male	Body weight↓<br>OFT: immobility time↑, total distance↓<br>FST: immobility time↑<br>TST: immobility time↑	After 30 days of UCMS and 30 days of drugs treatment	Fecal samples	_	16S rRNA gene sequencing-pyrosequencing-based analysis	V3-V4	UCMS-depression and control	One-way ANOVA with the Bonferroni test: p < 0.05

						UCMS (N=8)<br>UCMS+L. reuteri (N=8)<br>Control (N=8)										
Chen T et al. 2021[13]	C57BL/6N mice	China	case-control	3-4 mice/cage	CRS-depression<br><br><br><br><br><br>FMT CRS-depression	Experiment 1:<br>CRS (N=8)<br>DSS (N=8)<br>DSS+CRS (N=8)<br>Control (N=8)<br><br>Experiment 2:<br>donor Control (N=10)<br>donor CRS (N=20)<br>recipient Control (N=6)<br>recipient CRS (N=6)<br>recipient CRS+A. muciniphila (N=6)<br>recipient Control+DSS(N=8)<br>recipient CRS+DSS(N=8)<br>recipient CRS+A. muciniphila+DSS (N=8)	18–20 g	male	OFT: total distance↓<br>TST: immobility time↑<br>FST: immobility time↑	After 7 days of acclimatization and 30 days of CRS, followed by 7 days of DSS treatment	Cecal contents	_	16S rRNA gene sequencing-Illumina HiSeq platform	V3–V4	CRS-depression and control<br><br><br><br><br><br>FMT-CRS-depression and FMT-control	Two-way ANOVA with Bonferroni's post-hoc test for multiple comparisons: $p < 0.05$.
Chen X et al. 2021[14]	Sprague-Dawley rats	China	case-control	_	Lead exposure-depression	Lead exposure (N=15)<br>Control (N=15)	Adult 200 ±10 g	male	SPT: sucrose preference↓<br>FST: immobility time↑<br>OFT: total distance↑, center distance↑<br>EPM: open arms entries↑, open arms time↓	After 1 week of acclimatization and 24 weeks of lead exposure	Fecal samples	_	16S rRNA gene sequencing-	V4	Lead exposure-depression and control	Anosim: $p < 0.05$<br>LEfSe: $p < 0.05$ and LDA > 3.0
Chen X et al. 2022[15]	Sprague-Dawley rats	China	case-control	_	Lead exposure-depression	Lead exposure (N=15)<br>Lead exposure+Probiotics (N=15)<br>Control (N=15)	Adult 200 ±10 g	male	SPT: sucrose preference↓<br>FST: immobility time↑<br>TST: immobility time↑	After 1 week of acclimatization and 24 weeks of lead exposure	Fecal samples	_	16S rRNA gene sequencing-	V4	Lead exposure-depression and control	Anosim: $p < 0.05$<br>LEfSe: $p < 0.05$ and LDA > 3.0
Chen Y et al. 2021a[16]	C57BL/6 mice	China	case-control	_	CUMS-depression	Prevention trial:<br>CUMS (N=6)<br>CUMS+Fluoxetine (N=6)<br>CUMS+PHGG (N=6)<br>CUMS+PHGG+Fluoxetine (N=6)<br>Control (N=6)<br><br>Intervention trial:<br>CUMS (N=7)<br>CUMS+Fluoxetine (N=7)<br>CUMS+PHGG (N=7)<br>CUMS+PHGG+Fluoxetine (N=7)	5-week-old	male	Body weight↓<br>SPT: sucrose preference↓<br>FST: immobility time↑<br>OFT: center time↓, rearing number and crossing number↓	After 1 week of acclimatization, mice feces were collected every week during 4 weeks of CUMS and drugs treatment	Fecal samples	−80°C	16S rRNA gene sequencing-Illumina HiSeq platform	V3–V4	CUMS-depression and control	Student's t-test: $p < 0.05$
Chen Y et al. 2021b[17]	Sprague-Dawley rats	China	case-control	_	CUMS-depression	CUMS (N=10)<br>CUMS+ Semen Sojae Praeparatum (N=10)<br>Control (N=10)	Adult 180–220 g	male	SPT: sugar preference↓<br>FST: immobility time↑<br>LDT: dark time↑	After 15 days of acclimatization, 3 weeks of CUMS and 4 weeks of Semen Sojae Praeparatum treatment	Cecum contents	−80°C	16S rRNA gene sequencing-Illumina MiSeq platform	V3-V4	CUMS-depression and control	LEfSe: $p < 0.05$ and LDA > 3.0<br>one-way ANOVA and Welch's t-test: $p < 0.05$

Cheng D et al. 2018[18]	Sprague-Dawley rat	China	case-control	single	Hydrocortisone-depression	Hydrocortisone (N=10)<br>Hydrocortisone+Tiansi (N=10)<br>Control (N=10)	250–280 g	male	OFT: total distance↓<br>SPT: sucrose preference↓	After 1 week of acclimatization and 21 days of drugs	Feces from the colon samples and small intestinal mucosa	−80 ℃	16S rRNA gene sequencing-MiSeq (Illumina) platform	V3–V4	Hydrocortisone-depression and control	A Kruskal–Wallis test: $p < 0.05$<br>Metastats: FDR-adjust $p \leq 0.1$
Cheng R et al. 2021[19]	C57BL/6 mice	China	case-control	_	CUMS-depression	CUMS (N=7-8)<br>CUMS+Amuc_1100 (N=7-8)<br>CUMS+Fluoxetine (N=7-8)<br>Control (N=7-8)	5–6 weeks old	male	LDT: light time↓	After 6 weeks of CUMS and treatment	Fecal samples	_	16S rRNA gene sequencing-	V3-V4	CUMS-depression and control	One-way ANOVA and Fisher's least significant difference (LSD) tests for multiple comparisons: $p < 0.05$
Chevalier G et al. 2020[20]	C57BL/6J mice	France	case-control	_	UCMS-depression	UCMS (N=4)<br>Control (N=4)<br><br>FMT-UCMS (N=4)<br>FMT-Control (N=4)	8–10-week-old	male	NSFT: latency to eat↑<br>Splash test: grooming latency↑, self-grooming behavior↓<br>FST:immobility time↑<br>TST:immobility time↑	After 1 week of acclimatization and 8 weeks of UCMS<br><br>8 weeks post FMT	Fecal samples	_	16S rRNA metagenomic-Illumina MiSeq instrument	V3–V4	UCMS-depression and control<br><br>FMT-UCMS and FMT-control	Mann–Whitney test: $p < 0.05$
Chi L et al. 2020[21]	Sprague-Dawley rats	China	case-control	4-6 rats/cage for control, single for CUMS mice	CUMS-depression	CUMS (N=12)<br>CUMS+FOS (N=12)<br>CUMS+Flx (N=12)<br>CUMS+DP5 (N=10)<br>Control (N=8)<br>Control+FOS (N=8)	6-week-old	male	Body weight↓<br>SPT: sucrose preference↓<br>OFT：total distance↓, immobility time↑, travel velocity↓	After 1 week of acclimatization and 7 weeks of CUMS, the last 3 weeks received drugs treatment, fecal samples were collected at day 0, 15, and 22 during the drug treatment period	Fecal samples	−80 ℃	16S rRNA gene pyrosequencing-Illumina MiSeq system	V3–V4	CUMS-depression and control	LEfSe: $p < 0.05$ and LDA > 1.0
Choi J et al. 2022[22]	C57BL6/J mice	Korea	case-control	_	CRS-depression	CRS (N=12)<br>CRS+Lac-EV (N=8)<br>CRS+Bac-EV (N=12)<br>CRS+Akk-EV (N=11)<br>Control (N=12)<br>Control+Lac-EV (N=6)<br>Control+Bac-EV (N=12)<br>Control+Akk-EV (N=12)	7-week-old	male	FST: immobility time↑<br>TST: immobility time↑	After 5 days of acclimatization, stools were collected at day 1 (before stress), day 14 (after stress), and post-stress day 14	Fecal samples	−80◦C	16S rRNA gene sequencing-Roche 454 sequencing	V1-V2	CRS-depression and control (post-stress day 14)	Two-way ANOVA: $p < 0.05$
Daug éV et al. 2020[23]	Long Evans Rats	China	case-control	_	MD-depression	Experiment for Fischer rats:<br>Probiotics (N=12)<br>Control (N=12)<br>Experiment for Long Evans rats:<br>MD (N=12)<br>MD+Probiotics (N=12)<br>Control (N=12)<br>Control+Probiotics (N=12)	6-week-old	male	OFT: center visits unmber↓, rearings number↓	After receiving 0.5 mL of the probiotics for 5 weeks for Fischer rats and 9 weeks for Long Evans rats (until euthanasia)	Cecal content	−80 ℃	16S rRNA sequencing analysis-Illumina MiSeq platform	V3-V4	MD-depression and control	ANOVA: p < 0 05
Deng Y et al. 2021[24]	C57BL/6J mice	China	case-control	5 mice/cage	CRS-depression	CRS+PBS (N=8)<br>CRS+CITA (N=8)<br>Control+PBS (N=8)<br>Control+CITA (N=8)	6-week-old	male	FST: immobility time↑<br>SPT: sucrose preference↓<br>PFT: center time↓<br>EPM: open arms time↓	After 2 week of acclimatization and 5 weeks of CRS (the last 3 weeks received drugs)	Fecal samples	−80 ℃	16S rRNA gene sequencing-Illumina MiSeq platform	V3-V4	CRS-depression and control (PBS)	Two-way ANOVA followed by Fisher's LSD test or Dunn's correction: $p < 0.05$

Dhaliwal J et al. 2018[25]	Swiss albino LACA mice	India	case-control	_	CUMS-depression<br><br><br><br><br>SD-depression	CUMS (N=8)<br>CUMS+L. plantarum MTCC 9510 (N=8)<br>Control (N=8)<br>Control+L. plantarum MTCC 9510 (N=8)<br><br>SD (N=8)<br>SD+L. plantarum MTCC 9510 (N=8)<br>Control (N=6)<br>Control+L. plantarum MTCC 9510 (N=6)	25–30 g	male	Locomotor activity↓<br>EZM: open arm time↓<br>MCT: mirror chamber time↓<br>Stereotypic behaviour: stereotypy scores↑<br>MWM: transfer lantency↓, target quadrant time and frequency of appearance↓<br>PAR: number of mistakes↑<br>FST: immobility time↑<br>TST: immobility time↑	After 4 weeks of CUMS (and L. plantarum MTCC 9510)<br><br><br><br><br>After 21 days of L. plantarum MTCC 9510 (day 7-10 received sleep deprivation)	Caecal contents	_	qPCR	_	CUMS-depression and control<br><br><br><br><br><br><br>SD-depression and control	One-way ANOVA followed by Tukey's multiple comparison test:<br>p < 0 05
Ding Y et al. 2021[26]	C57BL/6 mice	China	case-control	_	CRS-depression	CRS (N=6)<br>CRS+A. muciniphila (N=6)<br>A. muciniphila (N=6)<br>CRS+ Lactobacillus low-dose (N=6)<br>CRS+ Lactobacillus high-dose (N=6)<br>Control (N=6)	6-8-week-old	male	OFT: total distance↓<br>FST: immobility time↑<br>TST: immobility time↑	After 1 week of acclimatization and 3 weeks of CRS and Akkermansia muciniphila treatment	Cecal contents and fecal samples	−80◦C	16S rRNA gene sequencing-Illumina platform	V3-V4	CRS-depression and control	Welch's t-test: p < 0.05
Diviccaro S et al. 2019[27]	Sprague-Dawley rats	Italy	case-control	_	Finasteride-depression	Finasteride after treatment (N=10)<br>Control after treatment (N=10)<br>Finasteride after withdrawal (N=10)<br>Control after withdrawal (N=10)	250-275g	male	FST: immobility time↑	After 1 week of acclimatization and 20 days of finasteride treatment and 1 month after withdrawal	Fecal samples	_	16S rRNA gene sequencing-Illumina MiSeq platform	V3-V4	Finasteride-depression and control (withdrawal)	Mann-Whitney test: p < 0.05
Donoso F et al. 2020[28]	Sprague-Dawley rats	Ireland	case-control	2-4 rats/cage	MS-depression	MS (N=12)<br>MS+Phlorotannins (N=10)<br>MS+Xanthohumol (N=10)<br>MS+Quercetin (N=10)<br>Control (N=12)	16-week-old	male	FST: immobility time↑, swimming time↓<br>OFT: center time and entries↓	After maternal separation (PND2-12) and dietary intervention of polyphenols (week 9-16)	Fecal samples	_	16S rRNA gene sequencing-Illumina MiSeq platform	V3-V4	MS-depression and control	Pairwise implementation of the aldex.test() function followed by Benjamini-Hochberg correction: q < 0.1
Du HX et al. 2020[29]	NOD/ShiLtJ mice	China	case-control	_	EAP-depression<br><br><br>ABX FMT-EAP-depression	EAP treatment (N=8)<br>Control (N=8)<br><br>ABX FMT-EAP (N=8)<br>ABX FMT-Control (N=8)<br>ABX FMT-PBS (N=8)<br>Na ïve (N=8)	4-week-old	male	OFT: center distance↓, center time↓, rearing number↓<br>SPT: sucrose preference↓<br>FST: immobility time↑<br>TST: immobility time↑	After 1 week of acclimatization and 24 days of immunization<br><br>After 1 week of acclimatization, 14 days of antibiotics treatment and 14 days fecal transplantation	Fecal samples	−80 ℃	16S rRNA gene sequencing-Illumina Miseq platform	V3-V4	EAP-depression and control<br><br>ABX FMT-EAP and ABX FMT-Control	Independent sample t test: p < 0.05<br>One-way ANOVA followed by post hoc Tukey tests: p < 0.05
Duan J et al. 2021[30]	C57BL/6 mice	China	case-control	Single	CUMS-depression	CUMS (N=7)<br>CUMS+escitalopram response (N=7)<br>CUMS+escitalopram non-response (N=9)<br>Control (N=8)	4–6 weeks of age	male	Body weight↓<br>SPF: sucrose preference↓<br>FST: immobility time↑	After 4 weeks of CUMS then fecal samples were collected prior to ESC administration (day 0) and at 4 weeks post administration (end of study)	Fecal samples	−80 ℃	16S rRNA gene sequencing-Illumina MiSeq platform	V3-V4	CUMS-depression and control	Wilcoxon rank-sum test: p < 0 05
Egerton S et al. 2020[31]	Sprague–Dawley rats	Ireland	case-control	2-4 mice/cage	MS-depression	MS (N=12)<br>MS+Fluoxetine (N=12)<br>MS+Fish oil (N=10)<br>MS+Fluoxetine+Fish oil (N=12)<br>Control (N=12)	16-week-old	male	OFT: center time and entries↓<br>FST: immobility time↑, swimming time↓	After maternally separated stress (PND2-12), and drugs intervention (week 8-16), fecal samples were collected at 16 weeks	Fecal samples	_	16S rRNA gene sequencing-Illumina MiSeq platform	V3–V4	MS-depression and control	Student's t-test: p < 0.05

El Aidy S et al. 2017[32]	mice	Netherlands	case-control	_	MS	MS-5-HTT−/− (N=8)<br>Control-5-HTT−/− (N=8)<br>MS-5-HTT+/− (N=8)<br>Control-5-HTT+/− (N=8)<br>MS-5-HTT+/+ (N=8)<br>Control-5-HTT+/+ (N=8)	pnd21	male/female	NA	Fecal samples were collected at PND 21.	Fecal samples	−80◦C	16S rRNA gene sequencing-Illumina MiSeq platform	Unspecified	MS and control	The rank test-Kruskal–Wallis test: $p < 0.05$
Fan L et al. 2021[33]	Sprague-Dawley rats	China	case-control	_	CUMS-depression	CUMS (N=9)<br>CUMS+TG (N=9)<br>CUMS+CTE (N=9)<br>CUMS+Fluoxetine (N=9)<br>CUMS+TG- PhG (N=9)<br>CUMS+TG-IrG (N=9)<br>CUMS+HT (N=9)<br>Control (N=9)	6-week-old	male	SPT: sucrose preference↓<br>OFT: total distance traveled↓, rearing number↓<br>FST: total immobility time↑	After 1 week of acclimatization and 4 weeks of CUMS	Cecum content samples	−80 ℃	16S rRNA gene sequencing-Illumina MiSeq PE300 system	V3-V4	CUMS-depression and control	LefSe: $p < 0.05$ and LDA > 2.5<br>Kruskal-Wallis H-test: $p < 0.05$
Farshim P et al. 2016[34]	Wistar albino rats	UK	case-control	6 rats/cage	Non-weaned-depression	S-Weaned (N=6)<br>S-Non-weaned (N=6)<br>NS-Weaned (N=6)<br>NS-Non-weaned (N=6)	PND25	male	FST: immobility time↑, limbing time↓, swimming time↓	Fecal samples were collected at PND25	Contents of duodenum, jejunum, ileum, cecum and colon	_	Fluorescence in situ Hybridization (FISH) analysis for Lactobacillus—Enterococcus, Bifidobacterium spp. and Clostridium histolyticum group	_	Non-weaned-depression and weaned	Two-way ANOVA followed by Bonferroni-adjusted: $p <0.05$
Feng Y et al. 2020[35]	Sprague-Dawley rats	China	case-control	_	CUMS-depression	CUMS (N=6)<br>CUMS+Bupleuri Radix (N=6)<br>CUMS+Venlafaxine (N=6)<br>Control (N=6)	200 ±20 g	male	Body weight↓<br>SPT: sucrose preference↓<br>OFT: crossing and rearing number↓	After 1 week of acclimatization and 4 weeks of CSDS	Cecum contents	−80 ℃	16S rRNA sequencing analysis-Illumina MiSeq platform	V3-V4	CUMS-depression and control	one-way ANOVA followed by Dunnett's test: $p < 0.05$<br>LEfSe: $p < 0.05$ and LDA > 3.0
Feng Z et al. 2020[36]	Sprague Dawley rats	China	case-control	single	CMS-depression	CMS (N=6)<br>CMS+low-dose WYJYD (N=6)<br>CMS+medium-dose WYJYD (N=6)<br>CMS+high-dose WYJYD (N=6)<br>CMS+Fluoxetine (N=6)<br>Control (N=6)	8-week-old	male	FST: immobility time↑	After 1 week of acclimatization and 4 weeks of CMS with drugs treatment	Colon contents	_	16S rRNA gene sequencing-	Unspecified	CMS-depression and control	One-way ANOVA: $p < 0.05$
Forouzan S et al. 2021[37]	Sprague-Dawley rats	USA	case-control	2 rats/cage	METH-depression	METH (N=8)<br>Control (N=8)	60–90 days old	male	OFT: total distance↓<br>FST: immobility time↑	Fecal samples were collected at the following time points: saline day 5, day 7, and day 14 of METH administration, and at 24, 48, and 96 h, and days 7, 14, and 30 of withdrawal or cessation	Fecal samples	−80 ℃	16S rRNA gene sequencing-Illumina MiSeq platform	V4	METH-depression and control	Non-parametric Mann-Whitney test or the Kruskal-Wallis test: $p < 0.05$
Gao K et al. 2022[38]	BALB/c mice	China	case-control	_	CUMS-depression	CUMS (N=10)<br>CUMS+Lc. lactis WHH2078 (N=10)<br>CUMS+Fluoxetine (N=10)<br>Control (N=10)	6–8 weeks old	male	SPT: sugar preference↓<br>FST: immobility time↑<br>TST: immobility time↑<br>OFT: total distance↓, center time↓	After 1 week of adaptation and 5 weeks of WHH2078 or fluoxetine treatment (4 weeks of CUMS after adaptation)	Fecal samples	−80◦C	16S rRNA gene sequencing-Illumina MiSeq platform	V3-V4	CUMS-depression and control	One-way ANOVA (or Kruskal–Wallis test): $p < 0.05$<br>LEfSe: $p < 0.05$ and LDA > 3.0

Gao X et al. 2020[39]	Sprague-Dawley rats	China	case-control	_	CUMS-depression	CUMS (N=6)<br>CUMS+TPC (N=6)<br>CUMS+Venlafaxine (N=6)<br>Control (N=6)	200 ±20 g	male	Body weight↓<br>SPT: sucrose preference↓<br>OFT:crossing number↓, rearing number↓	After 1 week of acclimatization and 4 weeks of CUMS	Cecal contents	−80 ℃	16S rRNA gene sequencing-Illumina MiSeq platform	V3-V4	CUMS-depression and control	One-way ANOVA followed by Dunnett's test: $p < 0.05$
Gong X et al. 2021[40]	C57BL/6J mice	China	case-control	5 mice/cage	CSDS-depresstion	CSDS (N=10)<br>Control (N=10)	6–8-week-old	male	SIT: SI ratio↓<br>OFT: total and central distance↓, central time and entries↓<br>FST: immobility time↑	After 1 week of acclimatization and 10 days of CSDS	Fecal samples	−80◦C	16S rRNA gene sequencing-Illumina MiSeq platform	V3–V4	CSDS-depresstion and control	LEfSe: $p < 0.05$ and LDA > 3.0
Gu F et al. 2020[41]	Sprague-Dawley rats	China	case-control	Single	CUMS-depression	CUMS (N=6)<br>CUMS+L. casei (N=6)<br>CUMS+Paroxetine (N=6)<br>Control (N=5)	5-week-old	male	Body weight↓<br>SPT: sucrose consumption↓<br>FST: immobility time↑<br>OFT: moving distance↓, velocity↓	After 1 week of acclimatization and 7 weeks of CUMS (the last 4 weeks received drugs treatment)	Fecal samples	_	16S rRNA gene sequencing-Illumina HiSeq 250 platform	V3-V4	CUMS-depression and control	LEfSe: Unspecified
Gu X et al. 2022[42]	Wistar rats	China	case-control	4 rats/cage	PSD-depression	PSD (N=8)<br>PSD+Conventional coffee (N=8)<br>PSD+Decaffeinated coffee (N=8)<br>Control (N=8)	240 ±10g	male	OFT: total behavioral score↓<br>FST: immobility time↑<br>TST: immobility time↑<br>SPT: sucrose preference↓	After 1 week of acclimatization and 7 days of paradoxical sleep deprivation	Fecal samples	−80 ℃	16S rRNA sequencing analysis-Illumina MiSeq platform/NovaSeq platform	V3-V4	PSD-depression and control	One-way ANOVA test and two tailed Student's t-test: $p < 0.05$
Guida F et al. 2018[43]	C57/bl6 mice	Italy	case-control	_	Antibiotic-depresstion	Antibiotics (N=10-12)<br>Antibiotics+L. casei (N=10-12)<br>Antibiotics+Saline (N=10-12)<br>Control (N=10-12)<br>Antibiotics+Recovery (N=10-12)	6-week-old	male	TST: immobility time↑<br>FST: immobility time↑	After 1 week of acclimatization, 14 days of antibiotics treatment, and 7 days of probiotics treatment	Fecal samples	_	16S rRNA gene sequencing-Illumina MiSeq platform	V3–V4	Antibiotic-depresstion and control	Kruskal-Wallis and pairwise Wilcoxon tests: $p < 0.05$
Guo Y et al. 2018[44]	ICR mice	China	case-control	5 mice/cage	CRS-depression	CRS (N=12)<br>CRS+Rosemary extracts (N=12)<br>Control (N=12)	Adult	male	OFT: center time↓<br>TST: immobility time↑<br>FST: immobility time↑	After 1 week of acclimatization and 3 weeks of CRS	Fecal samples	_	16S rRNA gene sequencing-Illumina MiSeq platform	V1-V3	CRS-depression and control	One-way ANOVA: $p < 0.05$
Guo Y et al. 2019[45]	ICR mice	China	case-control	4–5/cage	CRS-depression	Experiment 1:<br>Amitriptyline (N=10)<br>Low-dose B. adolescentis (N=10)<br>Middle-dose B. adolescentis (N=10)<br>High-dose B. adolescentis (N=10)<br>Control (N=10)<br><br>Experiment 2:<br>CRS (N=12)<br>CRS+B. adolescentis (N=12)<br>Control (N=12)	6-week-old	male	OFT: center time↓<br>EPM: open arms time and entries↓<br>FST: immobility time↑<br>TST: immobility time↑	After 1 week of acclimatization and 21 days of CRS	Cecal contents	_	16S rRNA gene sequencing-Illumina MiSeq platform	V1-V3	CRS-depression and control	One-way ANOVA followed by the Student-Newman-Keuls test: $p < 0.05$
Han SK et al. 2019[46]	C57BL/6 mice	Republic of Korea	case-control	_	IS-depression	IS (N=8)<br>IS+NK41 (N=8)<br>IS+NK46 (N=8)<br>IS+NK41+NK46 (N=8)<br>IS+fluoxetine (N=8)<br>Control (N=8)	6-week-old	male	EPM: open arms time and entries↓<br>FST: immobility time↑<br>TST: immobility time↑	After 2 days of IS	Fecal samples	_	qPCR	_	IS-depression and control	One-way ANOVA followed by a Duncan multiple range test: $p < 0.05$

Han SK et al. 2020a[47]	C57BL/6 mice	Korea	case-control	_	IS-depresstion<br><br><br><br>EC-depresstion	Experiment 1:<br>IS (N=6)<br>IS+Red ginseng low-dose (N=6)<br>IS+Red ginseng middle-dose (N=6)<br>IS+Red ginseng high-dose (N=6)<br>IS+Fermented red ginseng low-dose (N=6)<br>IS+Fermented red ginseng middle-dose (N=6)<br>IS+Fermented red ginseng high-dose (N=6)<br>Control (N=6)<br><br>Experiment 2:<br>IS (N=6)<br>IS+Fluoxetine (N=6)<br>IS+Red ginseng low-dose (N=6)<br>IS+Red ginseng middle-dose (N=6)<br>IS+Fermented red ginseng low-dose (N=6)<br>IS+Fermented red ginseng middle-dose (N=6)<br>Control (N=6)<br><br>Experiment 3:<br>EC (N=6)<br>EC+Buspirone (N=6)<br>IS+Red ginseng low-dose (N=6)<br>IS+Red ginseng middle-dose (N=6)<br>IS+Fermented red ginseng low-dose (N=6)<br>IS+Fermented red ginseng middle-dose (N=6)<br>Control (N=6)<br><br>Experiment 4:<br>EC (N=6)<br>EC+Ginsenoside Rd (N=6)<br>EC+Protopanaxatriol (N=6)<br>Control (N=6)	6-week-old	male	EPM: open arm time and entries↓<br>TST: immobility time↑<br>FST: immobility time↑<br>LDT: light box time↓	After 1 week of acclimatization, 5 days of Escherichia coli exposed and 5 days of drugs treatment	Colon contents	−80◦C	16S rRNA gene sequencing-Illumina iSeq 100 platform	V4	EC-depresstion and control	One-way ANOVA followed by Tukey’s multiple range test: $p < 0.05$<br>LEfSe: $p < 0.05$ and LDA > 3.5
Han SK et al. 2020b[48]	C57BL/6 mice	Korea	case-control	_	EC-depresstion	EC (N=5)<br>EC+Lactobacillus reuteri NK33 (N=5)<br>EC+Bifidobacterium adolescentis NK98	5-week-old	male	TST: immobility time↑<br>FST: immobility time↑	After 1 week of acclimatization, 5 days of Escherichia coli	Fecal samples	_	16S rRNA gene sequencing-Illumina iSeq 100 platform	V4	EC-depresstion and control	One-way ANOVA followed by Tukey’s multiple range test: p <

						(N=5)<br>EC+NK33/NK98(1:1) (N=5)<br>EC+NK33/NK98(4:1) (N=5)<br>EC+NK33/NK98(9:1) (N=5)<br>Control (N=5)				exposed and 5 days of drugs treatment						0.05<br>LEfSe: $p < 0.05$ and LDA > 3.5
Han SK et al. 2021[49]	C57BL/6 mice	Korea	case-control	3 mice/cage	RS-depresstion<br><br>FMT-RS-depression	Experiment 1:<br>RS (N=6)<br>RS+CSS low-dose (N=6)<br>RS+CSS middle-dose (N=6)<br>RS+CSS high-dose (N=6)<br>Control (N=6)<br><br>Experiment 2:<br>RS (N=6)<br>RS+CSS middle-dose (N=6)<br>RS+Buspirone (N=6)<br>Control (N=6)<br><br>Experiment 3:<br>Control (N=6)<br>FMT-RS (N=6)<br>FMT-Control (N=6)<br><br>Experiment 4:<br>FMT-RS (N=6)<br>Control (N=6)<br>FMT-RS+CSS to RS (N=6)<br>FMT-Control to RS (N=6)	6-week-old	male	EPM: open arm time and entries↓<br>TST: immobility time↑<br>FST: immobility time↑<br>LDT: light box time and entries↓	After 1 week of acclimatization, 2 days of RS exposed and 5 days of drugs treatment	Fecal samples	_	16S rRNA gene sequencing-Illumina iSeq platform	V4	RS-depresstion and control<br><br>FMT-RS-depression and control	Kruskal–Wallis test with Dunn's post-hoc test: $p<0.05$
Hao W et al. 2021[50]	C57BL/6 mice	China	case-control	_	Antibiotic-depression	Ampicillin (N=10)<br>Ampicillin+Xiaoyaosan (N=10)<br>Ampicillin+Probiotics (N=10)<br>Control (N=10)	8-week-old	male	Body weight↓<br>TST: immobility time↑<br>OFT: total distance↓<br>EPM: open arms time and distance↓	After 1 week of acclimatization and 2 weeks of ampicillin administration	Cecal contents	20 ℃	16S rRNA gene sequencing-<br>_	V3–V4	Antibiotic-depression and control	Kruskal–Wallis multi-sample rank sum test: $p < 0.05$
Hao WZ et al. 2021[51]	C57BL/6 mice	China	case-control	_	CUMS-depression	CUMS (N=10)<br>CUMS+Coniferyl ferulate (N=10)<br>Control (N=10)	8-week-old	male	Body weight↓<br>SPT: sugar preference↓<br>FST: immobility time↑<br>TST: immobility time↑<br>OFT: total distance↓, center frequency↓<br>EPM: open arms time and entries↓	After 1 week of acclimatization and 8 weeks of CUMS (teh last 4 weeks received coniferyl ferulate treatment)	Colon contents	_	16S rRNA gene sequencing-	V3-V4	CUMS-depression and control	LEfSe: $p < 0.05$ and LDA > 4.0<br>Repeated ANOVA: $p < 0.05$
Hassan AM et al. 2019[52]	C57BL/6J mice	Austria	case-control	2-3 mice/cage	HFD-depression	HFD (N=12)<br>Control (N=12)	8-week-old	male	SIT: mouse compartment preference↓, mouse near vicinity preference↓<br>Hair coat index:↑<br>SPF: sucrose preference↓	caecal contents were collected from each rat at the end of the HFD experiment (8 weeks)	Caecal contents	_	16S rDNA sequencing-<br>Ion PGM Sequencer and an Ion Sequencing 400 Kit	Unspecified	HFD-depression and control	LEfSe: $p < 0.05$ and LDA > 2.0

									LabMaster system: horizontal and vertical locomotor activity↓<br>MPT: morphine preference↓, saccharin solution intake↓							
Huang F et al. 2021[53]	C57BL/6 mice	China	case-control	pair-housed	Ovariectomy-depression	Ovariectomy (N=7-8)<br>Ovariectomy+P. histicola (N=7-8)<br>Ovariectomy+Cohousing (N=7-8)<br>Control (N=7-8)	10–12-week-old	female	OFT: center time and frequency↓<br>EPM: open arms time and frequency↓<br>FST: immobility↑<br>TST: immobility↑	14 weeks after ovariectomy	Fecal samples	−80 ℃	16S rRNA sequencing analysis-Illumina MiSeq platform	Unspecified	Ovariectomy-depression and control	Kruskall-Wallis H test: $p < 0$ 05<br>LEfSe: $p < 0.05$ and LDA > 3.0
Huang N et al. 2019[54]	C57BL/6 mice	China	case-control	_	LPS-depresstion	LPS (N=8)<br>LPS+ketamine (N=8)<br>Control (N=8)	2-month-old	male	FST: immobility time↑	After 1 week of acclimatization, fecal samples were collected after LPS and ketamine treatment	Fecal samples	−80◦C	16S rRNA gene sequencing-Illumina Miseq platform	V3-V5	LPS-depresstion and control	Fisher's exact test: $p < 0.05$<br>LEfSe: $p < 0.05$ and LDA > 2.0
Huang YJ et al. 2021[55]	C57BL/6J mice	China	case-control	single	sCSDS-depresstion	sCSDS (N=7)<br>sCSDS + WGE (N=7)<br>Control (N=7)	5-week-old	male	Water and food intake↑<br>SIT: social interaction ratio↓, total distance↓<br>OFT: total distance↓, crossings number↓, center bouts proportion↓<br>SPT: sucrose preference↓	After 1 week of acclimatization and 10 days of sCSDS	Fecal samples	_	16S rRNA gene sequencing-Illumina NextSeq platform	V4	sCSDS-depression and control	Kruskal-Wallis test: $p < 0.01$
Huang YY et al. 2022[56]	C57BL/6N mice	China	case-control	_	DSS-depression	DSS (N=8)<br>DSS+low-dose L. plantarum(N=8)<br>DSS+high-dose L. plantarum (N=8)<br>DSS+Fluoxetine(N=8)<br>Control (N=8)	4–5 weeks	male	OFT: total distance↓, center distance↓<br>LDT: light area distance↓<br>SPT: sugar preference↓<br>FST: immobility time↑	After 7 days of adaption and 7 days of DSS treatment	Fecal samples	−80◦C	16S rRNA gene sequencing-Illumina platform	V3-V4	DSS-depression and control	LEfSe: $p < 0.05$ and LDA > 2.0<br>One-way ANOVA: $p < 0.05$
Inserra A et al. 2019[57]	C57BL/6J mice	Australia	case-control	Single	(Casp1, Ifngr, Nos2) KO anti-depression<br><br>CUS-depression	(Casp1, Ifngr, Nos2) KO CUS (N=20)<br>(Casp1, Ifngr, Nos2) KO (N=20)<br>Wild-type CUS(N=16)<br>Wild-type (N=16)	60 days	male	(Casp1, Ifngr, Nos2) KO:<br>FST: foating time↓, swimming and climbing time↑<br>SPT: sucrose preference↑<br>EPM: open arms time↑<br>CUS:<br>FST: foating time↑, climbing time↑<br>EPM: open arms time↓<br>OFT: center time↓	After 1 week of acclimatization and 4 weeks of CUS	Fecal samples	−80 ℃	16S rRNA gene sequencing-Illumina Miseq	V4	(Casp1, Ifngr, Nos2) KO anti-depression and wild-type<br><br>CUS-depression and control	LEfSe: $p < 0.05$ and LDA > 3.0
Jang HM et al. 2019[58]	C57BL/6 mice	Korea	case-control	_	IS-depresstion	IS (N=7)<br>PC+Buspirone (N=7)<br>IS+NK33 (N=7)<br>IS+NK98 (N=7)<br>IS+NK33+NK98 (N=7)<br>Control (N=7)	5-week-old	male	EPM: open arm time and entries↓<br>TST: immobility time↑<br>FST: immobility time↑<br>LDT: light box time↓, transition number↓	After 1 week of acclimatization, 2 days of immobilization stress and 5 days of drugs treatment	Fecal samples	_	qPCR for Firmicutes, Bacteroidetes, Actinobacteria and δ/γ-Proteobacteria	_	IS-depresstion and control	One-way ANOVA followed by a Duncan multiple range test: $p < 0.05$
Ji S et al. 2022[59]	C57BL/6 mice	China	case-control	_	CRS-depression	CRS (N=15)<br>CRS+high-dose JWXY (N=10)<br>CRS+middle-dose JWXY (N=14)<br>CRS+low-dose JWXY (N=12)	8-week-old	_	SPT: sucrose preference↓<br>FST: floating time↑<br>OFT: total distance↓, center distance and time↓	After 1 week of acclimatization, the fecal samples were collected after 8 weeks of CRS	Cecal contents	−80 ℃	Full-length 16S rRNA sequencing-PacBio platform	_	CRS-depression and control	LEfSe: $p < 0.05$ and LDA > 4.0<br>Metastats: $p < 0.05$ or FDR < 0.05

						CRS+Sertraline (N=14)<br>Control (N=10)			Y-Maze: alternation↓<br>LDT: Time (count) and time (percent)↓							
Jiang W et al. 2021[60]	Sprague-Dawley rats	China	case-control	5 rat/cage	Post-stroke depression	Post-stroke depression (N=30)<br>Stroke (N=21)<br>Control (N=14)	180-200g	male	Body weight↓<br>SPT: sucrose preference↓<br>OFT: center time↓<br>FST: immobility time↑	After 1 week of acclimatization, 3 days after IR surgery and 4 weeks of CUMS	Fecal samples	−80 ℃	16S rRNA gene sequencing-Illumina MiSeq platform	V3-V4	Post-stroke depression and control	LEfSe: $p < 0.05$ and LDA ≥ 2.0
Jiang Y et al. 2020[61]	C57BL/6J mice	China	case-control	Single	Alcohol-depression	Alcohol (N=7)<br>Alcohol+NR (N=7)<br>Control (N=7)<br><br>ABX FMT-alcohol (N=6)<br>ABX FMT-alcohol+NR (N=6)<br>ABX FMT-control (N=6)	7-week-old	male	Body weight↓<br>SPT: sucrose preference↓<br>FST: immobility time↑<br>EMP: open arms time and entries↓<br>Y-maze test: novel arm time↓, start arm time↑	After 3 weeks of group hosed and 10 weeks of alcohol exposure	Colonic contents	−80 ℃	16S rRNA gene sequencing-Illumina HiSeq PE250 platform	V3-V4	Alcohol-depression and control	LEfSe: $p < 0.05$ and LDA > 2.0
Jianguo L et al. 2019[62]	Sprague–Dawley rats	China	case-control	single	CUMS-depression	CUMS (N=6)<br>Control (N=6)	8-week-old	male	SPT: sugar preference↓<br>OFT: immobility time↑, grooming time↓, crossing counts↓, rearing counts↓<br>FST: immobility time↑<br>TST: immobility time↑	After 1 week of acclimatization and 4 weeks of CUMS	Fecal samples	_	16S rRNA gene sequencing-Illumina MiSeq system	V4	CUMS-depression and control	LEfSe: adjusted $p < 0.05$ and LDA > 3.0
Kamimura Y et al. 2021[63]	C57BL/6J mice	Japan	case-control	single	CSDS-depression<br><br>Modified CSDS-depression	CSDS (N=5)<br>Control (N=7)<br><br>Modified CSDS (N=5)<br>Control (N=6)	6-week-old	male	Body weights↑<br>food consumption↑<br>SIT: social interaction ratio↓<br>FST: immobility time↑	After 1 week of acclimatization and 10 days of CSDS	Colon contents	_	16S rRNA gene sequencing-Illumina MiSeq platform<br><br>RT-PCR for Bifidobacterium spp.	V3-V4	CSDS-depression and control<br><br>Modified CSDS-depression and control	Student's t-test: $p < 0.05$
Karen C et al. 2021[64]	Wistar rats	India	case-control	_	MS-depression	Experiment 1:<br>MS (N=6)<br>M+P+S (N=6)<br>MS+S−M (N=6)<br>Control (N=6)<br><br>Experiment 2:<br>MS+Probiotics (N=6)<br>M+P+S+Probiotics (N=6)<br>MS+S−M+Probiotics (N=6)<br>Control+Probiotics (N=6)	PND34	_	OFT: cross squares number↓, center time and entries↓<br>FST: immobility time↑, swimming time↓	After maternal separation (PND5-10) and probiotics treatment (PND2-16), fecal samples were collected at PND27	Fecal samples	−80◦C	16S rRNA gene sequencing-Illumina MiSeq platform	V3–V4	MS-depresstion and control	One-way ANOVA and post hoc comparisons were performed with the Bonferroni test: $p < 0.05$
Kelly JR et al. 2016[65]	Sprague-Dawley rats	Ireland	case-control	_	ABX FMT-depression	ABX FMT-MDD (N=13)<br>ABX FMT-HC (N=15)	Adult 350 g	male	SPT: sucrose preference↓<br>EPM: open arms visits↓<br>OFT: center time↓	After 14 days of acclimatization, 28 days of antibiotic , 3 days of FMT and 7 dyas of recolonization	Fecal samples	−80 ℃	16S rRNA gene sequencing-Illumina Miseq platform	Unspecified	FMT-MDD and FMT-HC	Mann-Whitney U test and Benjamini-Hochberg FDR-adjusted p-value ≤ 0.1
Kemp KM et al. 2021[66]	C57BL/6J mice	USA	case-control	_	MS	MS+Early weaning (N=37)<br>Control (N=31)	PND28	male:female(1:1)	NA	Colon contents were collected at PND2, PND10 and PND28	Colon contents	−80◦C	16S rRNA gene sequencing-Illumina MiSeq platform	V4	MS and control	Analysis of composition of microbiomes (ANCOM)

Kim JK et al. 2020[67]	C57BL/6J mice	South Korea	case-control	3-4 mice/cage	EC-depression	Escherichia coli K1 (N=7)<br>EC K1+L. mucosae NK41 (N=7)<br>Control (N=7)	5-week-old	male	EPM: open arms time↓<br>FST: immobility time↑	After 1 week of acclimatization and 5 days of Escherichia coli K1 treatment and 5 days of Lactobacillus mucosae NK41 treatment	Fecal samples	−80◦C	16S rRNA gene sequencing-Illumina iSeq 100 platform	V4	EC-depression and control	LEfSe: $p < 0.05$ and LDA > 2.0<br>one-wayANOVA followed by a Duncan multiple range test: $p < 0.05$
Kim JK et al. 2021[68]	C57BL/6N mice	South Korean	case-control	3 mice/cage	IS-depression<br><br>EC-depression	IS (N=6)<br>IS+Buspirone orally gavaged (N=6)<br>IS+Buspirone intraperitoneally injected (N=6)<br>Control (N=6)<br><br>EC (N=6)<br>EC+Buspirone orally gavaged (N=6)<br>EC+Buspirone intraperitoneally injected (N=6)<br>Control (N=6)	6-week-old	male	EPM: open arms time and entries↓<br>LDT: light time and transition↓<br>FST: immobility time↑<br>TST: immobility time↑	After 2 days of immobilization stress (or Escherichia coli K1) and 5 days of buspirone treatment	Fecal samples	_	16S rRNA gene sequencing-Illumina iSeq 100 platform	V4	IS-depression and control<br><br>EC-depression and control	LEfSe: $p < 0.05$ and LDA > 3.5
Knudsen JK et al. 2021[69]	Flinders sensitive line rats<br>Flinders resistant line rats	Denmark	case-control	pair-housed	FMT-MDD-depression	FSL FMT-MDD (N=10)<br>FSL FMT-Healthy (N=10)<br>FSL CON-Auto (N=10)<br>FSL CON-H2O (N=10)<br>FRL FMT-MDD (N=10)<br>FRL FMT-MDD-Ser (N=10)<br>FRL FMT-Healthy (N=10)<br>FRL CON-Auto (N=10)<br>FRL CON-H2O (N=10)	6–8-week-old	male	FST: struggling↓, immobility↑	After 1 week of acclimatization, faecal samples were collected before transplantation (pre-FMT) and after transplantation (post-FMT)	Fecal samples	−80 ℃	16S rRNA sequencing analysis-Illumina MiSeq platform	V4	FRL FMT-MDD and FMT-Healthy	Kruskall-Wallis test followed by Dunn's post hoc test: p < 0 05
Kosuge A et al. 2021[70]	C57BL/6J mice	Japan	case-control	_	CSDS-depresstion	Experiment 1:<br>CSDS (N=11)<br>Control (N=22)<br><br>Experiment 2:<br>CSDS+M-16V (N=12)<br>CSDS (N=21)<br>Control+M-16V (N=12)<br>Control (N=28)	7-week-old	male	SIT: interaction zone time↓, corner areas time↑	The fecal samples were collected 1 day before exposure of CSDS and after 5 days of CSDS	Fecal samples	−80◦C	16S rRNA gene sequencing-Illumina MiSeq platform	V3-V4	CSDS-depresstion and control	LEfSe: $p < 0.05$ and LDA > 2.0
Kuti D et al. 2020[71]	C57BL/6J mice	Hungary	case-control	2-3 mice/cage	MS+CVS-depresstion	MS+CVS+Rifaximin (N=14)<br>MS+CVS (N=12)<br>Control+Rifaximin (N=10)<br>Control (N=9)	PND80	male	OFT: velocity and total distance↑, center time↓, first latency in border↓<br>EPM: Open arm preference↓<br>SPT: Sugar consumption↓	Colon contents were collected after 12 days of MS (PND1-12) and 4 weeks of CVS (PND50-78)	Colon contents	−70◦C	RT-qPCR	_	MS+CVS-depresstion and control	Two-way ANOVA followed by Sidak's multiple comparison test: $p <0.05$
Lai WD et al. 2022[72]	Wistar rats	China	case-control	_	SD-depression	SD (N=8)<br>SD+Fish oil-riched diet (N=8)<br>Control (N=8)	6-week-old	_	Body weight↓<br>OFT: total distance↓, center time and entries↓	After a 6-week dietary intervention and followed by 4-week chronic sleep deprivation	Colon contents	−80 ℃	16S rRNA gene sequencing-Illumina MiSeq platform	V3-V4	SD-depression and control	LEfSe: $p < 0.05$ and LDA > 3.0

									SPT: sugar preference↓<br>FST: immobility time↑							
Leclercq S et al. 2020[73]	C57BL/6J mice	Belgium	case-control	3 mice/cage	FMT-AD-depression	Experiment 1:<br>ABX FMT-AD (N=12)<br>ABX FMT-Control (N=12)<br><br>Experiment 2:<br>Ketogenic diet (N=21)<br>Control (N=21)	3-week-old	male	Three-chamber sociability test: chamber time↑, sociability index↓<br>FST: latency to immobility↓	After 10 days of antibiotic treatment, feces were collected at three time points (23, 35 and 45 days after FMT) and caecal content obtained at necropsy	Fecal samples<br>Caecal contents	−80 ℃	16S rRNA gene sequencing-Illumina MiSeq platform<br><br>qPCR for Faecalibacterium praustnizii	V5-V6	FMT-AD-depression and FMT-control	Wilcoxon test with FDR correction: q <0.05
Lee HC et al. 2020[74]	BALB/c mice	China	case-control	5-6 mice/cage	Lard diet-depression	Normal diet+Fluoxetine (N=6)<br>Fish oil-based diet (N=10)<br>Lard-based diet (N=10)<br>Normal diet control (N=6)	8-week-old	_	OFT: central zone visit number↓<br>FST: immobility time↑	After 4 weeks of acclimatization and 12 weeks of dietary intervention	Fecal samples	_	16S rRNA gene sequencing-Illumina HiSeq 2500 platform	V1–V3	Lard diet-depression and normal diet control	LEfSe: p < 0.05 and LDA > 3.0
Li H et al. 2019[75]	Wistar rats	China	case-control	_	CUMS-depression	CUMS (N=10)<br>CUMS+FOS/GOS (N=10)<br>CUMS+B. longum (N=10)<br>CUMS+L. rhamnosus (N=10)<br>Control (N=10)	200 ~ 220 g	male	Body weights↓<br>FST: immobility time↑<br>SPT: sucrose ingestion↓	After 7 days of acclimatization and 4 weeks of CUMS	Cecal contents	−80◦C	16S rRNA gene sequencing-Illumina HiSeq2500 platform	V4	CUMS-depression and control	Metastats analysis followed by Benjamini and Hochberg false discovery rate: q <0.05
Li H et al. 2021[76]	Sprague-Dawley rats	China	case-control	_	CUMS-depression	CUMS (N=14)<br>CUMS+Rifaximin (N=14)<br>Control (N=14)<br>Control+Rifaximin (N=14)	3-week-old	male	SPF: sucrose preference↓<br>OFT: crossing number↓, rearing number↓, center time↓	After 1 week of acclimatization and 28 days of CUMS and rifaximin treatment	Fecal samples	_	16S rRNA sequencing analysis-Shanghai Majorbio Bio-pharm Technology (Shanghai, China)	V3-V4	CUMS-depression and control	Two-way ANOVA followed by Benjamini and Hochberg correction post hoc test: q < 0 05
Li N et al. 2018[77]	C57BL/6 mice	China	case-control	3-4 mice/cage	CMS-depression	CMS (N=8)<br>CMS+Fluoxetine (N=8)<br>CMS+Probiotics (N=8)<br>Control (N=8)<br>Control+Fluoxetine (N=8)<br>Control+Probiotics (N=8)	6-8-week-old	male	SPT: sucrose preference↓<br>EPM: open arms time↓<br>FST: immobility time↑	After 2 weeks of acclimatization and 4 weeks of CMS	Cecal contents	−80◦C	16S rDNA gene sequencing-Illumina HiSeq PE250 platform	V3-V4	CMS-depression and control	Non-parametric Kruskal-Wallis test: p <0.05
Li N et al. 2019[78]	C57BL/6J mice	China	case-control	Single	CUMS-depression	CUMS (N=8)<br>Control (N=8)<br>CUMS-donor (N=8)<br>Control-donor (N=8)<br><br>ABX FMT-CUMS (N=8)<br>ABX FMT-Control (N=8)	7-week-old	male	Body weights↓<br>SPT: sucrose preference↓<br>OFT: center time↓<br>EPM: open arms time↓<br>FST: immobility time↑	After 1 week of acclimatization and 4 weeks of UCMS<br><br>2 weeks post FMT	Cecum content	−80 ℃	16S rRNA gene sequencing-Illumina HiSeq platform	V3–V4	CUMS-depression and control<br><br>FMT-CUMS and FMT-control	Wilcoxon rank sum tests: p < 0.05
Li P et al. 2021[79]	Sprague-Dawley rats	China	case-control	Single	CUMS-depression	CUMS (N=8)<br>CUMS+Acupuncture (N=8)<br>CUMS+Fluoxetine (N=8)<br>Control (N=8)	5-week-old	male	Body weight↓<br>OFT: total distance↓<br>EPM: open arms time↓<br>SPT: sucrose preference↓	After 1 week of acclimatization and 6 weeks of CUMS	Fecal samples	_	16S rDNA gene sequencing-Illumina platform	V3-V4	CUMS-depression and control	One-way ANOVA followed by Games-Howell test: p < 0.05
Li Q et al. 2021[80]	Sprague-Dawley rats	China	case-control	Single	CUMS-depression	CUMS (N=6)<br>CUMS+Paroxetin (N=6)	6-8-week-old	male	Body weights↓<br>FST: immovability time↑,	After 1 week of acclimatization and 4 weeks of CUMS	Cecal contents	_	RT-PCR for Lactobacillus, Bifidobacteria, Enterococcus faecalis and Escherichia coli	_	CUMS-depression and control	One-way ANOVA followed by the least significant difference test: p <0.05

						CUMS+Probiotic (N=6)<br>Control (N=6)			OFT: total distance↓, center time↓<br>SPT: sucrose preference↓							
Li Y et al. 2018[81]	Sprague-Dawley rats	China	case-control	in groups	CUS-depression	CUS+Saline (N=8)<br>CUS+high-dose CTE (N=8)<br>CUS+low-dose CTE (N=8)<br>CUS+Fluoxetine (N=8)<br>Control+Saline (N=8)	200 ±20 g	male	SPT: sucrose preference↓<br>OFT: total distance↓<br>NSF: latency to eat↑	After 1 week of acclimatization and 4 weeks of CUS	Fecal samples	−80 ℃	16S rRNA gene sequencing-Illumina MiSeq platform	V3-V4	CUS-depression and control	Metastats: $p < 0.05$
Lim EY et al. 2021[82]	Sprague Dawley rats	Korea	case-control	10-11 rats/cage	Ovariectomy-depression	Experiment 1:<br>Ovariectomy (N=11)<br>Control (N=11)<br><br>Experiment 2:<br>Ovariectomy (N=10)<br>Ovariectomy+17β-oestradiol (N=10)<br>Ovariectomy+VSL#3 (N=10)<br>Ovariectomy+L. intestinalis (N=11)<br>Ovariectomy+L. intestinalis YT2 (N=11)<br>Control (N=10)	10-week-old	female	Body weight↓<br>FST: immobility time↑	Faecal samples (0.5 g) were collected at 0, 1, 3, 6, 10, 14, 18 week after ovariectomy	Fecal samples	_	16S rRNA gene sequencing-high-sensitivity DNA chips and the Bioanalyzer 2100 (Agilent Technologies, Santa Clara, CA, USA)	V1-V2	Ovariectomy-depression and control	Wilcoxon rank-based test: $p < 0.05$<br>LEfSe: $p < 0.05$ and LDA > 3.0
Lin S et al. 2021[83]	ICR mice	China	case-control	5 mice/cage	CRS-depression	CRS (N=10)<br>CRS+low-dose crocetin (N=10)<br>CRS+middle-dose crocetin (N=10)<br>CRS+high-dose crocetin (N=10)<br>CRS+fluoxetine (N=10)<br>Control (N=10)	2-week-old	male	Body weights↓<br>TST: latency time↓, immobility time↑<br>OFT: crossing number↓, fecal number↑	After 28 days of CRS (before the behavioral tests)	Fecal samples	−80 ℃	16S rRNA gene sequencing-Illumina MiSeq 16S Metagenomic Sequencing Library Preparation protocol	V3–V4	CRS-depression and control	Student's test: $p < 0.05$
Liu QF et al. 2020[84]	ICR mice	Korea	case-control	_	IS-depresstion	IS (N=10)<br>IS+Fluoxetine (N=10)<br>IS+Probiotic (N=10)<br>Control (N=10)	6-week-old	male	FST: immobility time↑, swimming time↓<br>TST: immobility time↑<br>OFT: total distance↓, periphery and center diatance↓<br>EPM: closed arms time↑, open arms time↓	After 1 week of acclimatization, fecal samples were collected on day 60 after immobilization stress and fluoxetine or probiotic treatment	Fecal samples	−70 ℃	16S rRNA gene sequencing-Illumina MiSeq platform	V4-V5	IS-depresstion and control	Student's t test: $p <0.05$
Liu X et al. 2021a[85]	C57BL/6 mice	China	case-control	Single	CUMS-depression	CUMS+Inosine (N=30)<br>CUMS (N=30)<br>Control (N=15)	PND21	male	Body weights↓<br>SPT: sucrose preference↓<br>OFT: total distance↓<br>EPM: open arms time↓, closed arms time↑	After 1 week of acclimatization and 4 weeks of CUMS and inosine treatment	Fecal samples	−80◦C	16S rRNA gene sequencing-Illumina MiSeq platform	V3-V4	CUMS-depression and control	LEfSe: $p <0.05$ and LDA > 2.0
Liu XJ et al. 2021b[86]	Sprague-Dawley rats	China	case-control	_	CUMS-depression	CUMS (N=6)<br>CUMS+Venlafaxine (N=6)<br>CUMS+Xiaoyaosan (N=6)<br>CUMS+Shugan (N=6)<br>CUMS+Jianpi (N=6)<br>Control (N=6)	6-week-old	male	Body weights↓<br>SPT: sucrose preference↓<br>FST: immobility time↑	After 1 week of acclimatization and 4 weeks of CUMS and drugs treatment	Cecal contents	−80◦C	16S rRNA gene sequencing-Illumina MiSeq platform	V3-V4	CUMS-depression and control	One-way ANOVA followed by Tukey's post hoc test: $p <0.05$<br>LEfSe: $p < 0.05$ and LDA > 2.0
Liu Z et al. 2020[87]	C57BL/6J mice	China	case-control	4 mice/cage	HFD-postpartum depression	HFD (N=12)<br>HFD+Inulin (N=12)	2-month-old	female	MWM: escape time↑, target quadrant distance↓	Once the 21-day lactation was over	Fecal samples	−80 ℃	16S rRNA gene sequencing-Illumina MiSeq	V3-V4	HFD-postpartum depression and control	LDfSe: $p < 0.05$ and LDA > 3.0

						Control (N=12)<br>Control+Inulin (N=12)			TST: immobility time↑<br>OFT: crossing number↓							
Luo X et al. 2021[88]	C57BL/6J mice	China	case-control	_	EMF-induced-depression	Electromagnetic field exposure (EMF) (N=10)<br>EMF+Heat acclimation (N=10)<br>Heat acclimation (N=10)<br>Control (N=10)	8-week-old	male	FST: immobility time↑<br>TST: immobility time↑	After 28 days of ambient temperature, the fecal samples were collected after 5 weeks of EMF	Fecal samples	−80 ℃	16S rRNA sequencing analysis-Illumina MiSeq platform	V4	EMF-depression and control	LEfSe: $p < 0.05$ and LDA > 2.0
Lv M et al. 2021[89]	Sprague-Dawley rats	China	case-control	_	CUMS-depression	CUMS (N=6)<br>CUMS+Venlafaxine (N=6)<br>CUMS+Xiaoyaosan (N=6)<br>Control (N=6)	200 ±20 g	male	Body weights↓<br>SPT: sucrose preference↓<br>OFT: total distances and rearings number↓<br>FST: immobility time↑	After 1 week of acclimatization and 4 weeks of CUMS	Cecal contents	−80 ℃	16S rRNA gene sequencing-Illumina MiSeq platform	V3–V4	CUMS-depression and control	LEfSe: $p < 0.05$ and LDA > 2.0<br>Random Forests
Lv WJ et al. 2019[90]	Sprague-Dawley rats	China	case-control	Single	CUMS-depression	CUMS (N=6)<br>Control (N=6)<br><br>ABX FMT-CUMS (N=6)<br>ABX FMT-Control (N=6)	8-week-old	male	Body weight↓<br>SPT: sucrose preference↓<br>OFT: center time↓<br>TST: immobility time↑	After 1 week of acclimatization and 4 weeks of CUMS<br><br>_	Fecal samples	−80 ℃	16S rRNA gene sequencing-Unspecified<br><br>_	V3-V4	CUMS-depression and control	LEfSe: $p < 0.05$ and LDA > 3.0
Lv WJ et al. 2020[91]	Sprague-Dawley rats	China	case-control	_	DSS-depression	DSS (N=5)<br>DSS+Melatonin (N=6)<br>Control (N=7)	6-week-old	male	SPT: sucrose preference rates↓<br>OFT: center time↓<br>FST: immobility time↑	The fecal samples were collected after 7 weeks of DSS and 2 weeks of recovery	Fecal samples	−80◦C	16S rRNA gene sequencing-	V4–V5	DSS-depression and control	One-way ANOVA followed by Bonferroni's post hoc test: $p < 0.05$
Ma W et al. 2019[92]	Wistar rats	China	case-control	Single	PSD-depression	7d-PSD (N=10)<br>Control (N=10)	240 ±10 g	male	OFT: total behavioral score↓<br>FST: immobility time↑<br>TST: immobility time↑<br>SPT: sucrose preference↓	After 1 week of acclimatization and 7 days of paradoxical sleep deprivation	Fecal samples	−80 ℃	16S rRNA gene sequencing-Illumina MiSeq platform	V4	PSD-depression and control	Nonparametric Mann−Whitney U test: $p < 0.05$
Marin IA et al. 2017[93]	C57BL/6 mice	USA	case-control	2–3 mice/cage	UCMS-depression	UCMS (N=12)<br>control (N=11)	8-week-old	male	FST: escape behavior↓	After 1 week of acclimatization and 5 weeks of UCMS	Fecal samples	_	16S rRNA gene sequencing-Illumina MiSeq platform<br>qPCR	V3-V4	UCMS-depression and control	Two-tailed t-test with Welch's correction: $p < 0.05$
Martin-Hernandez D et al. 2016[94]	Wistar rats	Spain	case-control	Single	CMS-depression	CMS (N=9)<br>CMS+Antibiotics (N=10)<br>Control (N=8)	200-225g	male	Weight gain↓<br>FST: immobility time↑<br>SPT: sucrose consumption↓<br>Splash test: latency time↑<br>EPM: open arms time percentage↓	After 2 week of acclimatization and 3 weeks of CMS and antibiotics treatment	Blood, mesenteric lymph nodes (MLN), liver, spleen	_	Bacterial ecosystems analysis_	_	CMS-depression and control	_
Matsuda Y et al. 2020[95]	Sprague-Dawley rats	Japan	case-control	Single	CSDS-depression	CSDS (N=6)<br>Control (N=6)	8-week-old	male	SIT: social target↓	Fresh fecal samples were collected before the first SDS application (before), 1 day (stress 2d), 4 days (stress 5d), and 10 days (stress 11d) after the first SDS application,<br>and 1 day (after stress), 7 days (1 W), and 1 month (1 M) after the last SDS	Fecal samples	−80 ℃	16S rRNA gene sequencing-Illumina MiSeq platform	V3-V4	CSDS-depression and control (after stress)	Two-way ANOVA followed by Sidak's multiple comparisons test: $p < 0.05$

McGaughey KD et al. 2019[96]	C57BL/6J mice	USA	case-control	Single	CSDS-depression	CSDS (N=20)<br>Control (N=19)	6-week-old	male	OFT: total distance↓, vertical activity↓, center time↓, corner time↑<br>SPT: sucrose preference↓<br>FST: immobility time↑	24hr before the start of the social defeat trials and 24hr afer the social interaction testing (after 7 days of acclimatization and 7 days of CSDS)	Fecal samples	−80 ℃	16S rRNA gene sequencing-Illumina MiSeq platform	V3-V4	CSDS-depression and control	Wilcoxon Rank Sum and Kruskal-Wallis tests followed by a Dunn's post-test: $p < 0.05$
Medina-Rodriguez EM et al. 2020[97]	C57BL/6 mice	USA	case-control	_	LH-depression	LH (N=5)<br>Non-LH (N=5)<br>Control (N=3)	6-12-week-old	male	Escape failures↑<br>Learned helplessness:↑	Stools immediately after exposure to escapable foot shocks	Fecal samples	−80 ℃	16S rRNA gene sequencing-Illumina MiSeq instrument	V4	LH-depression and control	Kruskal-Wallis rank sum test with Bonferroni correction: $p < 0.05$
Meng C et al. 2022[98]	Sprague-Dawley rats	China	case-control	_	CUMS-depression	CUMS (N=20)<br>CUMS+Antibiotics (N=20)<br>Control (N=20)	6-week-old	male	FST: immobility time↑, swimming time↓	After 1 week of acclimatization and 4 weeks of CUMS, fresh feces samples were collected at end of short-term antibiotics exposure period (at week 5) and long-term antibiotics exposure period (at week 9)	Fecal samples	−80 ℃	16S rRNA gene sequencing-Illumina NovaSeq platform	V3-V4	CUMS-depression and control (short-term antibiotics exposure)	One-way ANOVA with a Duncan's test: $p < 0.05$
Moya-Pérez A et al. 2017[99]	C57BL/6J mice	Spain	case-control	_	MS-depression	MS (N=18)<br>MS+Bifidobacterium (N=18)<br>Control (N=18)<br>Control+Bifidobacterium (N=18)	PND21<br>PND40	male	EPM: open arms time↓	After maternal separation (PND2-21), stool samples were collected at PND21 and PND40	Fecal samples	−80◦C	16S rRNA gene sequencing-Illumina MiSeq platform	V4-V5	MS-depression and control (PND21)	Wilcoxon Ranks Sum test with post hoc Bonferroni test: $p < 0.05$
Murray E et al. 2019[100]	CD-1 mice	Canada	case-control	2 mice/cage	LPS-depression	Acute effect for both male and female:<br>LPS (N=10)<br>LPS+Probiotic (N=10)<br>Control (N=10)<br>Control+Probiotic (N=10)<br><br>Long term effect for both male and female:<br>LPS (N=10)<br>LPS+Probiotic (N=10)<br>Control (N=10)<br>Control+Probiotic (N=10)	3-week-old	male/female	FST: immobility time↑<br>OFT: center time↓<br>EPM: open arms time↓	Fecal samples were collected at five time points; 5 weeks of age (before probiotic treatment), 6 weeks of age (after 1 week of probiotics and just before LPS injection), 24 h after LPS injection, 7 weeks of age (at the end of two-week course of probiotic treatment), 10 weeks of age (adulthood)	Fecal samples	−80◦C	16S rRNA gene sequencing-Illumina MiSeq platform	V6-V8	LPS-depression and control	Three-way mixed ANOVA: $p < 0.05$
O'Mahony SM et al. 2020[101]	Sprague-Dawley rats	Ireland	case-control	3 rats/cage	MS-depression	MS (N=12)<br>MS+MFGM (N=12)<br>MS+Prebiotic (N=12)<br>MS+MFGM+Prebiotic (N=12)<br>Control (N=12)<br>Control+MFGM (N=12)<br>Control+Prebiotic (N=12)<br>Control+MFGM+Prebiotic (N=12)	14-week-old	male	NOR: discriminate ability↓	After maternal separation (PND2-12), stool samples were collected at the end of the study (weeks 14)	Fecal samples	−80◦C	16S rRNA gene sequencing-Illumina MiSeq platform	V3-V4	MS-depression and control	Rank Kruskal–Wallis test followed by Dunn's test with false discovery rate adjustment: $q < 0.05$

Osman A et al. 2021[102]	Wistar albino rats	USA	case-control	8 rats/cage	Casein-depression<br><br>A1/A2 β-casein-depression	Casein experiment：<br>Weaned+Casein-rich milk (N=8)<br>Weaned+Casein-free milk (N=8)<br>Weaned+Control (N=8)<br><br>A1/A2 milk experiment:<br>Weaned+A1/A2 β-casein milk (N=8)<br>Weaned+A2 β-casein milk (N=8)<br>Weaned+Control (N=8)	PND25	male	FST: immobility time↑	Fecal samples were collected at PND25	Contents of duodenum, jejunum, ileum, cecum and colon	_	Fluorescence in situ Hybridization (FISH) analysis for Bifidobacterium spp., Clostridium_histolyticum_group, Lactobacilli/Enterococci spp.	_	Casein-rich-depression and Casein-free control	Wilcoxon test followed by Benjamini-Hochberg: $p < 0.05$
Partrick KA et al. 2021[103]	Syrian hamsters (Mesocricetus auratus)	USA	case-control	Single	CSDS-depression	Acute CSDS+Placebo (N=20)<br>Acute CSDS+low probiotic (N=10)<br>Acute CSDS+high probiotic (N=20)<br>Repeat CSDS+Placebo (N=20)<br>Repeat CSDS+low probiotic (N=10)<br>Repeat CSDS+high probiotic (N=20)<br>Control+Placebo (N=20)<br>Control+low probiotic (N=10)<br>Control+high probiotic (N=20)	3-month-old	male	SBT: Avoidance behavior↑, Interacttion↓	Prior to the initial defeat (baseline samples), 24 h afer the acute defeat (acute defeat samples), and 24 h afer the fnal defeat (repeated defeat sample)	Fecal samples	−80 ℃	16S rRNA gene sequencing-Illumina MiSeq platform<br><br>qPCR	V3–V4	CSDS-depression and control	Nonparametric Kruskal–Wallis with Dunn's multiple comparisons test: $p < 0.05$<br>LEfSe: $p < 0.05$ and LDA > 2.0
Patterson E et al. 2019[104]	C57BL/6J mice	Ireland	case-control	3-4 mice/cage	HFD-depression	HFD (N=14)<br>HFD+ L. brevis DPC6108 (N=14)<br>HFD+L. brevis DSM32386 (N=14)<br>LFD (N=14)	3-week-old	male	Body weight↑<br>FST: immobility time↑	After 5 week of acclimatization, 24 weeks of high fat feeding (the last 12 weeks received L. brevis intervention)	Caecal contents	_	16S rRNA gene sequencing-Illumina MiSeq platform	V3-V4	HFD-depression and LFD-control	One-way ANOVA: $p < 0.05$
Pearson-Leary J et al. 2020[105]	Sprague-Dawley rats	USA	case-control	Single	CSDS-depression	CSDS-vulnerable (N=9)<br>CSDS-resilient (N=10)<br>Control (N=8)<br><br>FMT-CSDS-vulnerable (N=13)<br>FMT-CSDS-resilient (N=13)<br>FMT-Control (N=14)<br>Control (N=15)	275–300g	male	Rats exhibiting passive behavior and shortlatencies (SL/vulnerable) to defeat exhibit increased anxiety- and depressive-like behaviors<br>FST: latency to immobility↓, immobility time↑, swimming time↓	At 24 h before the first social defeat (day 0) and 24 h after the 7th episode of social defeat (30 min/day)(day 8)	Fecal samples	−80 ℃	Shotgun metagenome sequencing-Illumina HiSeq 2500<br><br>16S rRNA gene sequencing-Illumina MiSeq	_<br><br>V1-V2	CSDS-depression vulnerable and control	Wilcoxon rank sum test: adjust $p < 0.05$
Pu Y et al. 2021[106]	C57BL/6 mice	Japan	case-control	4-5/cage	Chrna7 KO-depression<br><br>FMT-Chrna7 KO-depression	Chrna7 KO (N=10)<br>Wild-type (N=10)<br><br>ABX FMT-Chrna7 KO (N=10)<br>ABX FMT-wild-type (N=10)	8-week-old	male	FST: immobility time↑<br>TST: immobility time↑<br>SPT: sucrose preference↓	At around 10:00 for KO mice<br><br>After 14 days of antibiotics treatment and 14 days of FMT	Fecal samples	−80 ℃	16S rRNA sequencing analysis-MyMetagenome Co., Ltd. (Tokyo, Japan)	Unspecified	Chrna7 KO-depression and wild-type<br><br>FMT-Chrna7 KO-depression and FMT-wild-type	LEfSe: $p < 0.05$ and LDA > 2.0
Pusceddu MM et al. 2015[107]	Sprague-Dawley rats	Ireland	case-control	5 rats/cage	MS	MS (N=10)<br>MS+EPA/DHA low-dose (N=10)<br>MS+EPA/DHA high-dose (N=10)<br>Control (N=10)	17-week-old	female	NA	After maternally separation stress (PND2-12) and EPA/DHA treatment (weeks 5-17), faecal pellets were collected at weeks 17 from female rats	Fecal samples	−80 ℃	16S rDNA gene sequencing-Illumina MiSeq platform	Unspecified	MS and control	Wilcoxon rank test: $p < 0.05$

						Control+EPA/DHA low-dose (N=10)<br>Control+EPA/DHA high-dose (N=10)										
Qiao Y et al. 2020[108]	Kunming mice	China	case-control	_	CRS-depression<br>CUMS-depression<br>CRS+CUMS-depression	CRS (N=10)<br>CUMS (N=10)<br>CRS+CUMS (N=10)<br>Control (N=10)	8-week-old	male	Body weight↓<br>Food consumption↓<br>SPT: sucrose preference↓<br>FST: immobility time↑<br>TST: immobility time↑<br>OFT: rest time↑, move time↓, center time and distance↓	After 1 week of acclimatization and 3 weeks of stress	Rectal contents	−80 ℃	16S rRNA gene sequencing-Illumina MiSeq platform	V3-V4	CRS-depression and control<br>CUMS-depression and control<br>CRS+CUMS-depression and control	Multiple t-test: p <0.05<br>LEfSe: p < 0.05 and LDA > 2.0
Qiu X et al. 2021[109]	C57BL/6J mice	China	case-control	Single	LPS-depression	LPS (N=8)<br>LPS+Lactobacillus (N=8)<br>Control (N=8)<br>Control+Lactobacillus (N=8)	8-week-old	_	SPF: sucrose preference↓<br>FST: immobility time↑	After 7 days of acclimatization, followd by LPS and 7 days of Lactobacillus treatment	Fecal samples	_	qPCR	_	LPS-depression and control	Two-way ANOVA followed by least significant difference (LSD) post-hoc test: p < 0.05
Qu W et al. 2019[110]	Sprague-Dawley rats	China	case-control	Single	CUMS-depression	CUMS-susceptible (N=6)<br>CUMS-susceptible+TCM (N=19)<br>Control (N=12)	adult 180-220 g	male	SPF: sucrose preference↓<br>OFT: rearing and crossing numbers↓<br>LDT: dark zone time↑	At least 2 fecal pellets were obtained from each rat in Week 9 and 15.	Fecal samples	−80◦C	16S rRNA gene sequencing-Illumina HiSeq 2500 platform<br><br>Shotgun metagenomic analysis-Illumina HiSeq 4000 platform	V4-V5	CUMS-depression and control	One-wa ANOVA followed by the LSD test: p < 0.05
Qu Y et al. 2017[111]	C57BL/6 mice	Japan	case-control	Single	CSDS-depression	CSDS susceptible+Saline (N=6)<br>CSDS susceptible+(R)-ketamine (N=6)<br>CSDS susceptible+Lanicemine (N=6)<br>Control (N=6)	8-week-old	male	FST: immobility time↑<br>TST: immobility time↑<br>SPT: sucrose preference↓	After 10 days of CSDS and 3 days after a single dose of drugs	Fecal samples	−80 ℃	16S rRNA gene sequencing-Illumina MiSeq 16S Metagenomic Sequencing	V4	CSDS-depression susceptible and control	One-way ANOVA followed by post-hoc Tukey test: p < 0.05
Qu Y et al. 2020[112]	C57BL/6 mice	Japan	case-control	_	CSDS-depression	CSDS (N=9)<br>CSDS+Betaine (N=9)<br>Control (N=11)<br>Control+Betaine (N=11)	8-week-old	male	SPT: sucrose preference↓	After 24 days of betaine treatment (day1-24) and 10 days of CSDS (day15-24), fecal samples were collected at day25	Fecal samples	−80◦C	16S rRNA gene sequencing-	V3-V4	CSDS-depression and control	Two-way ANOVA followed by post-hoc Fisher's Least Significant Difference (LSD) test: p < 0.05
Qu Y et al. 2022[113]	ICR mice	China	case-control	Single	CUMS-depression	CUMS (N=12)<br>Control (N=12)	7 weeks old	male	Body weight↓<br>SPT: sugar preference↓<br>FST: immobility time↑<br>TST: immobility time↑	After 1 week of acclimatization and 8 weeks of CUMS	Intestinal contents	−80 ℃	16S rDNA gene sequencing-Illumina MiSeq platform	V3-V4	CUMS-depression and control	Pearson's correlation analysis: p < 0.05
Rao J et al. 2021[114]	Sprague-Dawley rats	China	case-control	_	CUMS-depression	CUMS (N=6)<br>CUMS+FMT-control (N=6)<br>Control (N=6)	180 ±20 g	male	SPF: sucrose preference↓<br>FST: immobility time↑	After 1 week of acclimatization, 4 weeks of CUMS and 2 weeks of fecal microbiota transplantation treatment	Colonic contents	−80◦C	16S rRNA gene sequencing-Illumina MiSeq platform	V3-V4	CUMS-depression and control	Kruskal–Wallis test: p < 0.05
Ray P et al. 2021[115]	C57BL/6 mice<br>BALB/c mice	India	case-control	_	Vancomycin-depression	Vancomycin (N=6)<br>Control (N=6)	6-8-week-old	male	EPM: closed arms time↑<br>OFT: center time↓<br>FST: immobility time↑	Fresh cecal samples were collected everyday till 6th day (total of six time points) during pertuibation and four time points of restoration (on 15th, 30th, 45th, and 60th day)	Cecal contents	_	16S rRNA gene sequencing-Illumina MiSeq platform	V3-V4	Vancomycin-depression and control	ANOVA: p < 0.05

Robertson RC et al. 2017[116]	C57BL/6J mice	Ireland	case-control	3-4 mice/cage	n-3 PUFA deficiency-depression	n-3 PUFA supplement (N=10)<br>n-3 PUFA deficiency (N=10)<br>Control (N=10)	13-week-old	male	FST: immobility time↑<br>Three chamber test: time interaction with object↑, time interaction with mouse↓	Fecal samples were collected after 13 weeks of dietary treatment	Fecal samples	−20◦C	16S rRNA gene sequencing-Illumina MiSeq platform	V3-V4	n-3 PUFA deficiency-depression and control (adult only)	Kruskal-Wallis tests followed by Mann-Whitney tests: $p < 0.05$<br>LEfSe: $p < 0.05$ and LDA > 2.0
Rosa JM et al. 2020[117]	Swiss mice	Brazil	case-control	10 mice/cage	Aβ1-40-depression	Aβ1-40 (N=8)<br>Aβ1-40+Exercise (N=8)<br>Control (N=8)<br>Control+Exercise (N=8)	30-40 g, 45-55 days	male	FST: immobility time↑<br>FUST: sniffing time↓	After 1 week of acclimatization and 28 days of treadmill exercise (Aβ1-40 injection at day 18)	Colon contents	−80◦C	RT-qPCR for Firmicutes and Bacteroidetes	_	Aβ1-40-depression and control	Two-way ANOVA followed by Duncan's multiple range post hoc test: $p < 0.05$
Schmidtner AK et al. 2019[118]	NAB/HAB rats	Germany	case-control	3-4 rats/cage	HAB-depression	HAB (N= no data)<br>HAB+Minocycline (N= no data)<br>HAB+Escitalopram (N= no data)<br>HAB+Minocycline+Escitalopram (N= no data)<br>NAB (N= no data)<br>NAB+Minocycline (N= no data)<br>NAB+Escitalopram (N= no data)<br>NAB+Minocycline+Escitalopram (N= no data)	11-12-week-old	male/female	FST: struggling score↓, immobility score↑<br>LDT: light box time↓, distance traveled↓<br>EPM: open arms time↓	After 14 days of drugs injection	Cecum contents	−80◦C	16S rDNA gene sequencing-454 pyrosequencing	V3-V6	HAB-depression and NAB control	ANOVA with a subsequent Tukey's tes: $p < 0.05$
Shan B et al. 2021[119]	Sprague-Dawley rats	China	case-control	_	CUS-depression	Experiment 1:<br>CUS (N=15)<br>Control (N=10)<br><br>Experiment 2:<br>ABX (N=8-10)<br>ABX FMT-CUS (N=8-10)<br>ABX FMT-Control (N=8-10)<br>D-lactic acid (N=8-10)<br>L-lactic acid (N=8-10)<br>Control (N=8-10)	180–220 g	male	OFT: center time↓<br>EPM: open arms time↓, closed arms time↑<br>TST: immobility time↑<br>FST: immobility time↑	After 1 week of acclimatization and 5 weeks of CUS	Fecal samples	−80◦C	16S rRNA gene sequencing-Illumina MiSeq platform	V3-V4	CUS-depression and control	Wilcoxon rank-sum test: $p < 0.05$<br>LEfSe: $p < 0.05$ and LDA > 2.0
Shao S et al. 2021[120]	C57BL/6 J mice	China	case-control	Single	CRS-depression	CRS+CRC xenografts (N=5)<br>CRS+Fluoxetine+CRC xenografts (N=5)<br>CRS+XCHT-low+CRC xenografts (N=5)<br>CRS+XCHT-high+CRC xenografts (N=5)<br>Control+CRC xenografts (N=5)	6-7-week-old	male	SPF: sucrose preference↓<br>TST: immobility time↑	After 7 days of acclimatizationm, 14 days of CRS, 25 days of behavioural tests, tumour cell injection, and fluoxetine or XCHT treatment	Fecal samples	−80◦C	16S rRNA gene sequencing-Illumina MiSeq platform	V3-V4	CRS-depression and control	ANOVA test: $p < 0.05$
Sheng L et al. 2021[121]	Sprague-Dawley rats	China	case-control	_	CUMS-depression	CUMS (N=10)<br>CUMS+Fluoxetine (N=10)<br>CUMS+Exercise (N=10)<br>Control (N=10)	180 g	female	FST: swimming time↓, resting time↑<br>TST: resting time↑<br>OFT: total distance↓, peripheral distance↓<br>EPM: open arms time↓, closed arms time↑	Fecal samples were collected after 60 days of exercise	Fecal samples	−70◦C	16S rRNA gene sequencing-Illumina MiSeq platform	V4–V5	CUMS-depression and control	Mann–Whitney nonparametric test: $p < 0.05$
Siopi E et al. 2020[122]	C57BL/6J mice	France	case-control	5 mice/cage	UCMS-depresstion	Experiment 1:<br>UCMS (N=10)<br>Control (N=10)<br>ABX FMT-UCMS (N=10)	8-week-old	male	TST: immobility time↑<br>FST: immobility time↑	After 9 week of UCMS (the last week were recevied behavioral testing)	Fecal samples	−80◦C	16S rRNA gene sequencing-Illumina MiSeq platform	V3–V4	UCMS-depresstion and control<br><br>FMT-UCMS-	ANOVA test: $p < 0.05$

					FMT-UCMS-depression	ABX FMT-Control (N=10)<br><br>Experiment 2:<br>UCMS (N=10)<br>UCMS+Fluoxetine (N=12)<br>Control (N=10)<br><br>Experiment 3:<br>ABX FMT-UCMS (N=15)<br>ABX FMT-Control (N=13)<br>ABX FMT-UCMS+Fluoxetine (N=10)<br>ABX FMT-UCMS+5-HTP (N=10)<br>ABX FMT-UCMS+Fluoxetine+5-HTP (N=10)				After 1 week of ABX treatment, 4 days of microbiota transplantation, and 8 weeks of colonization (the last week were recevied behavioral testing)					depression and FMT-control	
Song J et al. 2019a[123]	Wistar rats	China	case-control	_	ACTH-depression	ACTH-treatment (N=10)<br>Control (N=10)	10-week-old	male	FST: immobility time↑<br>TST: immobility time↑	After 14 days of ACTH treatment	Fecal samples	−80 ℃	16S rRNA gene sequencing-Illumina MiSeq platform	V3-V4	ACTH-depression and control	LEfSe: $p < 0.05$ and LDA > 3.0
Song J et al. 2019b[124]	Wistar rats	China	case-control	_	ACTH-depression	ACTH (N=10)<br>ACTH+CGA (N=10)<br>control (N=10)	10-week-old	male	SPT: sugar preference↓<br>FST: immobility time↑<br>TST: immobility time↑<br>OFT: total distance↓, rearing number↓	After 7 days of acclimatization and 14 days of ACTH treatment	Fecal samples	−80 ℃	16S rRNA gene sequencing-Illumina MiSeq platform	V3-V4	ACTH-depression and control	LEfSe: $p < 0.05$ and LDA > 3.0
Song X et al. 2021[125]	ICR mice	China	case-control	Single	CUMS-depression	CUMS (N=8)<br>CUMS+Fluoxetine (N=8)<br>CUMS+Puerarin low-dose (N=8)<br>CUMS+Puerarin high-dose (N=8)<br>Control (N=8)<br>Control+Fluoxetine (N=8)<br>Control+Puerarin high-dos (N=8)	20.0 ±2.0 g	male	SPT: sucrose preference↓<br>FST: immobility time↑	After 1 week of acclimatization and 4 weeks of CUMS with drugs treatment	Fecal samples	−80◦C	16S rRNA gene sequencing-Illumina MiSeq platform	V3–V4	CUMS-depression and control	Kruskal-Wallis test with Bonferroni correction: $p < 0.05$<br>LEfSe: $p < 0.05$ and LDA > 2.0
Sovijit WN et al. 2019[126]	C57BL/6J mice	Japan	case-control	_	Ovariectomy-depression	Experiment 1:<br>Ovariectomy (N=15)<br>Ovariectomy+Progesterone (N=15)<br><br>Experiment 2:<br>Ovariectomy (N=5)<br>Ovariectomy+Progesterone (N=5)<br>Ovariectomy+L. reuteri (N=5)	6-week-old	female	OFT: center time and entries↓<br>FST: immobility time↑	After 2 weeks of recovery from ovariectomy (at 8-week-old of age) and 2 weeks of progesterone treatment	Fecal samples	_	16S rRNA gene sequencing-Illumina MiSeq platform	V3-V4	Ovariectomy-depression and ovariectomy+progesterone control	LEfSe: $p < 0.05$ and LDA > 2.0
Sun L et al. 2019a[127]	C57/6 mice	China	case-control	_	CUMS-depression	CUMS (N=10)<br>CUMS+Fluoxetine (N=10)<br>Control (N=10)	8-week-old	male	Body weight↓<br>SPT: sucrose preference↓<br>TST: immobility time↑<br>EPM: open arms time↓	After 1 week of acclimatization and 5 weeks of CUMS	Fecal samples	−80 ℃	16S rRNA gene sequencing-IlluminaMiseq platform	Unspecified	CUMS-depression and control	LEfSe: Unspecified
Sun L et al. 2019b[128]	C57BL/6 mice	China	case-control	_	Fto-KO anti-depression	Fto-KO (N=8)<br>Wild-type (N=8)	3-month-old	male	EPM: open arms time↑<br>OFT: central time↑<br>FST: floating time↓	Unspecified	Fecal samples	−80 ℃	16S rRNA gene sequencing-Illumina Miseq platform	V3–V4	Fto-KO anti-depression and wild-type	Wilcoxon rank sum test: $p < 0.05$

						Fto-KO (N=7)<br>wild-type (N=7)							Metagenomic-Illumina Hiseq2500 Microbiome Profiling setup	_		
Sun X et al. 2021[129]	C57BL/6 mice	China	case-control	3-4 mice/cage	CRS-depression	CRS (N=10)<br>CRS-WLPL04 (N=10)<br>Control (N=10)	4-week-old	male	OFT: center time↓<br>EPM: open arms time and entries↓<br>FST: immobility time↑	After 4 weeks of CRS	Fecal samples	_	16S rRNA gene sequencing-Illumina MiSeq platform	V3-V4	CRS-depression and control	One-way ANOVA: $p < 0.05$
Sun Y et al. 2019[130]	Kunming mice	China	case-control	_	CUMS-depression	CUMS (N=8)<br>CUMS+Fluoxetine (N=8)<br>CUMS+low-dose L.kefiranofaciens ZW3 (N=8)<br>CUMS+middle-dose L.kefiranofaciens ZW3 (N=8)<br>CUMS+high-dose L.kefiranofaciens ZW3 (N=8)<br>Control (N=8)	14-20 g	male	SPT: sucrose preference↓<br>FST: immobility time↑<br>OFT: vertical movements↓, crossings number↓	After 1 week of acclimatization and 6 weeks of CUMS (the last 2 weeks received drugs treatment)	Fecal samples	_	16S rRNA gene sequencing-Illumina HiSeq platform	V4	CUMS-depression and control	LEfSe: $p < 0.05$ and LDA > 2.0
Sun Y et al. 2020[131]	C57BL/6 mice	China	case-control	4 mice/cage	LPS-depression	LPS (N=12)<br>LPS+Schisandrin (N=12)<br>Control (N=12)	20 ±2 g	male	FST: immobility time↑<br>TST: immobility time↑	After 1 week of acclimatization and 2 weeks of vehicle or SCH treatment	Fecal samples	−80 ℃	16S rRNA gene sequencing-Illumina MiSeq platform	V3-V4	LPS-depression and control	One-way ANOVA followed by Tukey multiple comparison test : $p < 0.05$
Suzuki K et al. 2021[132]	C57BL/6 J (B6J) mice	Japan	case-control	_	CSDS-depression	CSDS (N=6)<br>CSDS+Crp4 (N=6)<br>Control (N=6)	7-week-old	male	SIT: social interaction time↓, distance traveled↓	We sampled at three time points on day 1, 9, and 14 in the first experiment and at two time points on day 0 and 32 in the second experiment	Fecal samples	−80◦C	16S rRNA gene sequencing-Illumina MiSeq platform	V3-V4	After CSDS-depression and pre-CSDS control	Pairs student's t test: $p < 0.05$
Szyszkowicz JK et al. 2017[133]	C57BL/6 mice	Canada	case-control	Single	CSDS-depression	CSDS-susceptible (N=10)<br>CSDS-resilient (N=8)<br>Control (N=6)	7–9-week-old	male	SIT: social interaction ratios↓, contact time↓, Corner zones time↑	After 1 week of acclimatization and 3 weeks of CSDS	Cecum contents	−80 ℃	16S rRNA gene sequencing-Illumina MiSeq Sequencing	V3-V4	CSDS-depression and control	One-way ANOVA followed by Bonferroni correction: $p < 0.05$
Takahashi E et al. 2021a[134]	C57BL/JJcl (B6) mice	Japan	case-control	group	AIN-93G-depression	AIN-93G young (N=10)<br>CRF-1 young (N=10)<br>AIN-93G old (N=10)<br>CRF-1 old (N=10)	3-week-old	male	TST: immobility time↑<br>FST: immobility time↑	After 5 weeks of AIN-93G diet for young and 85 weeks of AIN-93G diet for old mice	Small intestinal contents	−80◦C	Terminal restriction fragment length polymorphism (T-RFLP) method	_	AIN-93G-depression and CRF-1 control (in old mice)	ANOVA followed by the Bonferroni correction post hoc test: $p < 0.05$
Takahashi E et al. 2021b[135]	C57BL/JJcl (B6) mice	Japan	case-control	group	CRS-depression	Cohort 1<br>CRS+AIN-93G short (N=10)<br>CRS+CRF-1 short (N=10)<br>Control+AIN-93G short (N=10)<br>Control+CRF-1 short (N=10)<br><br>Cohort 2<br>CRS+AIN-93G long (N=10)<br>CRS+CRF-1 long (N=10)<br>Control+AIN-93G long (N=10)<br>Control+CRF-1 long (N=10)	3-week-old	male	Body weight↓<br>FST: struggling time↓, immobility time↑SPT: sucrose preference↓	After 3 weeks of CRS and 1 week of AIN-93G diet for cohort 1 or 5 weeks of AIN-93G diet for cohort 2	cecum contents	−80◦C	Terminal restriction fragment length polymorphism (T-RFLP) method	_	CRS+AIN-93G long-depression and Control+AIN-93G long control	Unpaired t-test with Welch's correction: $p < 0.05$

Teng T et al. 2021[136]	Macaca fascicularis monkeys	China	case-control	Single	CUMS-depression	CUMS-depression (N=5)<br>Control (N=5)	1-4 year-old	male	Frequency and duration of huddle posture↑<br>Frequency and duration of locomotion↓<br>AAT: attempts for the apple↓<br>HIT: anxiety-like behaviors↑	Fresh feces were collected from mice of each group over 55 days after the model was built	Lumen and mucosa of cecum, ascending colon, transverse colon, and descending colon	−80◦C	16S rRNA gene sequencing-Illumina MiSeq platform	V3-V4	CUMS-depression and control	LEfSe: p < 0.05 and LDA > 2.0
Tian P et al. 2019a[137]	C57BL/6J mice	China	case-control	_	CUMS-depression	CUMS (N=6)<br>CUMS+Fluoxetine (N=6)<br>CUMS+CCFM687 (N=6)<br>Control (N=6)	6-week-old	_	FST: immobile time↑<br>TST: immobile time↑<br>SDT: latent time↑<br>EMP: open arms time↓<br>LDT: light box time↓	After 1 week of acclimatization and 5 weeks of CUMS	Fecal samples	_	16S rRNA gene sequencing-Illumina MiSeq platform	V3-V4	CUMS-depression and control	One-way ANOVA followed by Fisher's LSD multiple comparison test: p < 0.05
Tian P et al. 2019b[138]	C57BL/6J mice	China	case-control	_	CUMS-depression	CUMS (N=6)<br>CUMS+Fluoxetine (N=6)<br>CUMS+Probiotics (N=6)<br>Control (N=6)	6-week-old	male	FST: swimming time↓<br>SPT: sucrose preference↓<br>EPM: open arms time↓<br>OFT: center time↓<br>LDT: light chamber entries↓<br>SDT: latency (s) of jumping↑	After 5 weeks of CUMS	Fecal samples	_	16S rRNA gene sequencing-Illumina MiSeq platform	V3-V4	CUMS-depression and control	LEfSe: p < 0.05 and LDA > 2.0
Tian P et al. 2020[139]	C57BL/6J mice	China	case-control	_	CUMS-depression	CUMS (N=10)<br>CUMS+Fluoxetine (N=10)<br>CUMS+CCFM1025 (N=10)<br>Control (N=10)	6-week-old	male	FST: immobility time↑<br>TST: immobility time↑<br>OFT: center time↓<br>SPT: sucrose preference↓<br>EPM: open arms time↓	After 1 week of acclimatization and 5 weeks of CUMS (and drugs)	Fecal samples	_	16S rRNA gene sequencing-Illumina MiSeq platform	V3-V4	CUMS-depression and control	LEfSe: p < 0.05 and LDA > 3.0
Tian P et al. 2021[140]	mice	China	case-control	_	CRS-depression	CRS (N=6)<br>CRS+Acetylated starch (N=8)<br>CRS+Propionylated starch (N=8)<br>CRS+Butylated starch (N=8)<br>CRS+Isobutyrylated starch (N=8)<br>Control (N=10)	_	_	OFT: active time↓, open field time↓<br>FST: immobility time↑	After 1 week of acclimatization and 2 weeks of CRS	Fecal samples	_	16S rRNA gene sequencing-	V3-V4	CRS-depression and control	LEfSe: p < 0.05 and LDA > 2.0
Tian XY et al. 2021[141]	BALB/c mice	China	case-control	Single	IS-postpartum depression	IS-PPD (N=10)<br>IS-PPD+919 syrup (N=10)<br>Control (N=10)	7-week-old	female	FST: immobility time↑<br>TST: immobility time↑	On PND2, PND9, PND16, and PND23, fecal samples were collected from each mouse at 12 a.m. and quickly frozen in liquid nitrogen.	Fecal samples	Liquid nitrogen	16S rRNA gene sequencing-Illumina HiSeq2500 platform<br><br>Metagenomic Sequencing-Illumina HiSeq platform	V3-V4<br><br>_	IS-postpartum depression and control	LEfSe: p < 0.05 and LDA > 3.0
Tillmann S et al. 2019[142]	Flinders sensitive line rats<br>Flinders resistant line rats	Danish	case-control	pair-housed	FSL-depression	FSL-saline (N=8)<br>FSL FMT-FSL (N=8)<br>FSL FMT-FRL (N=8)<br>FRL-saline (N=8)<br>FRL FMT-FSL (N=8)<br>FRL FMT-FRL (N=8)	10.6 ±1.1 weeks old	male	Body weight↓<br>OFT: No data<br>FST: No data	After 2 weeks of acclimatization and 16 days of fecal suspensions transplantation	Fecal samples	−80 ℃	16S rRNA gene sequencing-Illumina MiSeq platform	V4	FSL-depression and FRL-control	DESeq2: adjusted p (Benjamini-Hochberg) < 0.05

Tung TH et al. 2019[143]	Sprague-Dawley rats	China Taiwan	case-control	_	CMS-depression	CMS (N=5)<br>CMS+Imipramine (N=5)<br>CMS+Fish oil (N=5)<br>CMS+Olive oil (N=5)<br>Control (N=5)	6-week-old	male	Body weights↓<br>OFT: total distances↓<br>SPT: sucrose preference↓<br>FST: immobile time↑	After 2 weeks of acclimatization and 12 weeks of CMS	Fecal samples	−80 ℃	16S rRNA gene sequencing-Illumina MiSeq system	V3–V4	CMS-depression and control	LEfSe: $p < 0.05$ and LDA > 2.0
Wang L et al. 2020[144]	Kumming mice	China	case-control	Single	CUMS-depression	CUMS (N=8)<br>CUMS+low-dose TIV (N=8)<br>CUMS+medium-dose TIV (N=8)<br>CUMS+high-dose TIV (N=8)<br>CUMS+Fluoxentine (N=8)<br>Control (N=8)	6-week-old	male	Body weight↓<br>FST: immobility time↓<br>SPT: sucrose preference↓	After 1 week of acclimatization and 4 weeks CUMS	Fecal samples	−80 ℃	16S rRNA gene sequencing-Illumina HiSeq platform	V4	CUMS-depression and control	One-way ANOVA followed by LSD tests: $p < 0.05$
Wang L et al. 2021[145]	Sprague-Dawley rats	China	case-control	_	CUMS-depression	CUMS (N=10)<br>CUMS+Fluoxetine (N=10)<br>CUMS+low-dose SI (N=10)<br>CUMS+middle-dose SI (N=10)<br>CUMS+high-dose SI (N=10)<br>Control (N=10)	200-220g	female and male	Body weight↓<br>SPT: sucrose preference↓<br>OFT: horizontal and vertical movements score↓<br>LDT: dark area time↑	After 2 weeks of acclimatization, 10 weeks of CUMS and 8 weeks of drugs	Fecal samples	_	16S rRNA gene sequencing-Illumina Miseq platform	V3-V4	CUMS-depression and control	LEfSe: $p < 0.05$
Wang P et al. 2021[146]	C57BL/6J mice	China	case-control	_	Antibiotic-depression	Antibiotic (N=9)<br>Antibiotic+Saline (N=9)<br>Antibiotic+Probiotics (N=9)<br>Control (N=9)	6-week-old	male	EPM: total crossing↓, open arms time↓, velocity↓<br>OFT: total distance↓, center time and distance↓<br>TST: immobility time↑<br>Splash test: grooming latency↓	After 1 week of acclimatization, fecal samples were collected after 3 weeks of ASC antibiotics treatment, and 2 weeks of probiotics treatment	Fecal samples	_	qPCR	_	Antibiotic-depression and control	One-way ANOVA followed by Tukey`s multiple comparisons test: $p < 0.05$
Wang Q et al. 2019[147]	CD-1 mice	China	case-control	single	CMS-depression	CMS (N=8)<br>CMS+SE (N=8)<br>Control (N=8)	3-month-old	male	SPT: sucrose preference↓<br>FST: immobility time↑<br>MBT: number of marbles buried↑<br>LDT: light zone duration↓	After 2 weeks of acclimatization and 10 weeks of CMS	Fecal samples	_	16S rRNA gene sequencing-Illumina MiSeq platform	V3−V4	CMS-depression and control	LEfSe: $p < 0.05$ and LDA > 3.0
Wang R et al. 2021[148]	C57BL/6J mice	China	case-control	_	CRS-depression	CRS (N=5)<br>CRS+TFA low-dosage (N=5)<br>CRS+TFA high-dosage (N=5)<br>Control (N=5)	6-week-old	male	OFT: total distance↓<br>FST: immobility time↑<br>TST: immobility time↑	After 7 days of acclimatization and 30 days of CRS	Cecum contents	_	16S rRNA gene sequencing-Illumina MiSeq platform	V3-V4	CRS-depression and control	One-way ANOVA: $p < 0.05$
Wang S et al. 2020a[149]	C57BL/6 mice	Japan	case-control	Single	FMT CSDS-depression<br><br><br><br><br><br>Microbe-depression	ABX FMT CSDS-susceptible (N=7)<br>Water FMT CSDS-susceptible (N=7)<br>ABX FMT control (N=7)<br>Water FMT control (N=7)<br><br>ABX+Microbe (N=10)<br>ABX+Water (N=10)<br>Water (N=10)	8-week-old	male	SPT: sucrose preference↓<br>FST: immobile time↑<br>TST: immobile time↑	After 14 days of antibiotic cocktail treatment and 14 days of FMT from CSDS-susceptible mice or control	Fecal samples	_	16S rRNA gene sequencing-MiSeq according to the Illumina protocol	V1-V2	FMT CSDS-depression and FMT control	Two-way ANOVA followed by post hoc Fisher's LSD test: $p < 0.05$
Wang S et al. 2020b[150]	C57BL/6 mice	Japan	case-control	_	CSDS-depression	CSDS (N=10)<br>CSDS+Antibiotics (N=10)	8-week-old	male	SPT: sucrose preference↓	Fresh fecal samples were collected on day 15 before CSDS	Fecal samples	−80◦C	16S rRNA gene sequencing-Illumina MiSeq platform	V1–V2	CSDS-depression and control	Two-way ANOVA followed by post hoc Tukey test: $p < 0.05$

						Control (N=10)<br>Control+Antibiotics (N=10)				and 24 hours after CSDS (day15-24)						
Wang S et al. 2021[151]	C57BL/6 mice	Japan	case-control	Single	FMT CSDS-depression	ABX FMT CSDS-susceptible Ephx2 KO (N=10)<br>ABX FMT CSDS-susceptible WT (N=10)<br>ABX FMT control Ephx2 KO (N=10)<br>ABX FMT control WT (N=10)	8-week-old	male	SPT: sucrose preference↓	After 14 days for antibiotic cocktail treatment and 14 days for FMT procedure	Fecal samples	−80◦C	16S rRNA gene sequencing-Illumina MiSeq platform	V1-V2	FMT CSDS-depression and control (in both Ephx2 KO mice and WT mice)	Kruskal-Wallis test: $p < 0.05$
Wang Y et al. 2021[152]	C57BL/6 mice	China	case-control	4 mice/cage	LPS-depresstion	LPS (N=20)<br>LPS+(S)-norketamine (N=15)<br>LPS+(R)-norketamine (N=15)<br>Control (N=8)	2-month-old	male	FST: immobility time↑<br>TST: immobility time↑	After 1 week of acclimatization and a single injection of LPS	Fecal samples	−80◦C	16S rRNA gene sequencing-Illumina MiSeq platform	V3-V5	LPS-depression and control	Kruskal-Wallis test: $p < 0.05$
Warda AK et al. 2019[153]	C57BL/6 mice	Ireland	case-control	4 mice/cage	ADR-159-depression	ADR-159 (N=12)<br>Control (N=12)	8-week-old	male	OFT: center time↓, total distance↓<br>3CT: interaction time↑, chamber time↑<br>TST: immobility time↑	After 1 week of acclimatization, faecal samples were collected weekly during the 8 weeks of designated diet	Fecal samples	−80◦C	16S rRNA gene sequencing-MiSeq sequencing (Germany)	V3-V4	ADR-159-depression and control	DESeq2 with Wald test: $p < 0.05$
Wei CL et al. 2019[154]	C57BL/6J mice	China-Taiwan	case-control	4 mice/cage	Corticosterone-depression	Corticosterone (N=8)<br>Corticosterone+Fluoxetine (N=8)<br>Corticosterone+Live L. paracasei PS23 (N=8)<br>Corticosterone+Heat-killed L. paracasei PS23 (N=8)<br>Control (N=8)	6–8 weeks old	male	OFT: total distance↓, center time and entries↓<br>SPT: sugar preference↓<br>FST: immobility time↑	Three or four fecal pellets were collected on day 38	Fecal samples	−20 ℃	qPCR for Bifidobacterium, Clostridium coccoides group, Enterobacteriaceae, Enterococcus, and Lactobacillus	_	Corticosterone-depression and control	One-way ANOVA followed by Newman-Keuls multiple comparison test: $p < 0.05$
Wei LN et al. 2019[155]	Wistar rats	China	case-control	Single	CUMS-depression	CUMS (N=9)<br>Control (N=9)	180–210 g	male	SPT: sucrose preference↓<br>FST: swimming time↓, immobility time↓	After 1 week of acclimatization and 5 weeks of CUMS	Rectal contents	−80◦C	16S rRNA gene sequencing-Illumina HiSeq2500 platform	V4	CUMS-depression and control	LEfSe: $p < 0.05$ and $LDA > 4.0$
Westfall S et al. 2021[156]	C57BL/6J mice	USA	case-control	group	CUMS-depression<br><br>CUMS+US-depression	Stress (N=16)<br>Stress+BDPP (N=16)<br>Stress+Probiotics (N=16)<br>Stress+Synbiotics (N=16)<br>Control (N=16)<br>Control+BDPP (N=16)<br>Control+Probiotics (N=16)<br>Control+Synbiotics (N=16)	8-week-old	male	FST: freezing time↑<br>OFT: center time↓	After 2 week of acclimatization and 11 weeks of respective treatment (fecal pellets were collected after 4 weeks of CUMS and after 7 days of US)	Fecal samples	−80◦C	16S rRNA gene sequencing-Illumina MiSeq platform	V4	CUMS-depression and control<br><br>CUMS+US-depression and control	One-way ANOVA and Tukey's post-hoc analysis: $p < 0.05$
Wong ML et al. 2016[157]	C57BL/6J mice	Australia	case-control	group	CRS-depression	CRS (N=15)<br>CRS+Minocycline (N=15)<br>Minocycline (N=12)<br>Control (N=12)	60–90 days	male	Body weight↓<br>FST: floating time↑, swimming and climbing behaviors↓<br>EPM: open arms time↓, open/close arms time↓<br>OPT: defecations number↑	After 21 days of CRS	Fecal samples	−80 ℃	16S rRNA gene sequencing-Illumina MiSeq platform	V4	CRS-depression and control	Mann–Whitney U-tests: $p < 0.05$
Wu F et al. 2020[158]	C57BL/6 mice	China	case-control	_	HFD-depression	HFD (N=10)<br>HFD+A. muciniphila (N=10)<br>Control (N=10)<br>Control+A. muciniphila (N=10)	8-week-old	female	SPT: sugar preference↓<br>YMT: the error times↑	After 2 week of acclimatization and 10 months of high-fat diet plus A. muciniphilasub gavage	Fecal samples	_	16S rDNA gene sequencing-Illumina MiSeq platform	V4	HFD-depression and control	One-way ANOVA: $p < 0.05$

Wu J et al. 2021[159]	C57/BL6 mice	China	case-control	Single	CUMS-depression<br>Dexamethasone-depression	CUMS (N=15)<br>Dexamethasone (N=15)<br>Control (N=15)	4 to 6-week-old	female	OFT: total distance↓, speed↓, crossing and rearing number↓<br>EPM: open arms time↓<br>FST: immobility time↑<br>TST: immobility time↑<br>SPT: sucrose preference↓	After 1 week of acclimatization, the fecal samples were collected after 35 days of CUMS or dexamethasone treatment	Fecal samples	−80 ℃	16S rRNA sequencing analysis-Illumina MiSeq platform	V3-V4	CUMS-depression and control<br>Dexamethasone-depression and control	LEfSe: $p < 0.05$ and LDA > 3.0<br>Kruskal-Wallis tests with multiple comparison correction: $p < 0.05$
Wu J et al. 2022[160]	Macaca fascicularis monkeys	China	case-control	group	Naturally-occurring depression	Depression (N=6)<br>Control (N=6)	Adult	female	Duration of huddle and sit-alone behaviors↑<br>Duration of amicable and locomotion activities↓	After behavioral tests	Cecum contents	−80 ℃	Metagenomic-Illumina HiSeq X platform	_	Naturally-occurring depression and control	LEfSe: $p < 0.05$ and LDA > 2.0
Wu M et al. 2020[161]	C57BL/6 mice	China	case-control	Single	CRS-depression	CRS (N=20)<br>Control (N=20)	8-16 week-old	male	OFT: center time↓, center distance↓<br>FST: immobility time↑<br>SPT: sucrose preference↓<br>Body weight↓	After 4 weeks of CRS	Fecal samples	−80 ℃	16S rRNA gene sequencing-Roche 454 sequencing	V3-V5	CRS-depression and control	LEfSe: $p < 0.05$ and LDA > 3.0
Xia J et al. 2021[162]	C57BL/6J mice	China	case-control	Single	LPS-depression	LPS (N=6)<br>CAP (N=6)<br>LPS+CAP (N=6)<br>Control (N=6)	4-week-old	male	SPT: sucrose preference↓<br>OFT: rearing and crossing number↓<br>FST: immobility time↑<br>TST: immobility time↑	After 4 months of CAP treatment. During the last 5 days, mice in LPS and CAP+LPS groups received lipopolysaccharide	Fecal samples	−80 ℃	16S rRNA gene sequencing-Illlumina MiSeq platform	V3-V4	LPS-depression and control	LEfSe: $p < 0.05$ and LDA > 3.0
Xiao Q et al. 2020[163]	C57BL/6 mice	China	case-control	Single	CRS-depression	CRS (N=10)<br>CRS+Crocin-I (N=10)<br>Control (N=10)	6-week-old	male	SPT: sucrose preference↓<br>FST: immobility time↑<br>TST: immobility time↑	After 1 week of acclimatization and 10 weeks of CRS (the last 6 weeks received crocin-I and duration of CRS is halved)	Cecal contents	−80 ℃	16S rRNA gene sequencing-Illumina MiSeq platform	V3-V4	CRS-depression and control	LEfSe: $p < 0.05$ and LDA > 2.0
Xie R et al. 2020a[164]	C57BL/6 mice	China	case-control	Single	CSDS-depression	CSDS-susceptible (N=7)<br>CSDS-resilient (N=7)<br>Control (N=7)	7-week-old	male	SIT: interaction ratio↓, interaction time↓<br>SPT: sucrose preference↓<br>OFT: total distance↓, center time↓, center entries↓	After 1 week of acclimatization and 10 days of CSDS	Peyer's patches-gut-associated lymphoid tissue	_	16S rRNA gene sequencing-Illumina MiSeq platform	V3-V4	CSDS-depression susceptible and control	One-way ANOVA: $p < 0.05$
Xie R et al. 2020b[165]	C57BL/6 mice	China	case-control	4 mice/cage	CSDS-depression	CSDS-susceptible (N=10)<br>CSDS-susceptible+L. reuteri 3 (N=10)<br>Control (N=10)<br>Control+L. reuteri 3 (N=10)	8-week-old	male	SIT: interaction ratio↓<br>SPT: sucrose preference↓<br>TST: immobility time↑<br>OFT: center time and entries↓, total distance↓	After 1 week of acclimatization, 10 days of CSDS and 4 weeks of microbial treatment	Colonic contents	−80 ℃	16S rRNA gene sequencing-Illumina MiSeq sequencing	V3-V4	CSDS-depression and control	One-way ANOVA test followed by Tukey's test: $p < 0.05$
Xie Y et al. 2021[166]	C57BL/6 mice	China	case-control	Single	SIMT-anti-depression	Small intestinal microbiota transplantation (N=6)<br>Large intestinal microbiota transplantation (N=6)<br>Control (N=6)	PND56	male	SPT: sucrose preference↑<br>FST: immobility time↓<br>TST: immobility time↓	Before microbiota transplantation (PND77) and after 14 days of microbiota transplantation (PND92)	Fecal samples	−80 ℃	16S rRNA gene sequencing-Illumina MiSeq platform	V3-V4	SIMT-anti-depression and control	Kruskal-Wallis H test followed by the Bonferroni post hoc test: $p < 0.05$
Xu J et al. 2022[167]	C57BL/6 mice	China	case-control	Single	CUMS-depression	CUMS (N=12)<br>CUMS+high-dose L. rhamnosus zz-1 (N=12)<br>CUMS+middle-dose L. rhamnosus zz-1	4-week-old	male	Body weight↓<br>SPT: sugar preference↓<br>FST: immobility time↑<br>OFT: total distance↓, center time↓	After 1 week of acclimatization and 5 weeks of CUMS	Cecal contents	−80 ℃	16S rDNA gene sequencing-Illumina MiSeq platform	V3-V4	CUMS-depression and control	one-way analyses of variance (ANOVAs) followed by Tukey's post hoc test: $p < 0.05$<br>LEfSe

						(N=12)<br>CUMS+low-dose L. rhamnosus zz-1 (N=12)<br>Control (N=12)										
Xu M et al. 2022[168]	C57BL/6J mice	China	case-control	_	CUS-depression	CUS (N=9)<br>CUMS+Lactobacillus for each strain (N=7)<br>Control (N=8)	6 weeks	male	OFT: center time↓<br>MBT: number of buried beads↑<br>LDT: open chamber time↓<br>FST: immobility time↑<br>TST: immobility time↑	After 1 week of acclimatization and 6 weeks of CUS	Colon contents	_	16S rRNA gene sequencing-Illumina MiSeq platform	V3-V4	CUS-depression and control	_
Xu Z et al. 2019[169]	C57BL/6 mice	China	case-control	4 mice/cage	CAE-depression	CAE (N=6)<br>Control (N=6)	4-week-old	male	OFT: center time and distance↓<br>EPM: open arms time↓<br>FST: mobility time↓<br>TST: mobility time↓	After 1 week of acclimatization and fecal samples were collected at the twentieth day of the drinking session	Fecal samples	−80 ℃	16S rRNA metagenomic sequencing-<br>16S metagenomic sequencing library protocol (Illumina)	V3–V5	CAE-depression and control	Unpaired Student's t-test: $p < 0.05$
Xue M et al. 2021[170]	C57BL/6J mice	China	case-control	Single	Alcohol-depression	Alcohol (N=8)<br>Alcohol+Fucoidan (N=8)<br>Control (N=8)	8-week-old	male	Body weight↓<br>Food intake↓<br>SPT: sucrose preference↓<br>FST: immobility time↑<br>Y-maze test: novel arms time↓<br>OFT: total distance↓, center time and entries↓	After 3 weeks of acclimatization and 10 weeks of alcohol exposure (and fucoidan treatment)	Colon contents	_	16S rRNA gene sequencing-Illumina HiSeq platform	V3-V4	Alcohol-depression and control	One-way ANOVA followed by Tukey's test or Kruskal-Wallis test followed by Bonferroni test: $p < 0.05$
Yan T et al. 2020[171]	C57BL/6 mice	China	case-control	4 mice/cage	CUMS-depression	CUMS (N=10)<br>CUMS+Polysaccharide (N=10)<br>Control (N=10)<br>CUMS-donor (N=10)<br>CUMS+Polysaccharide-donor (N=10)<br>Control-donor (N=10)<br><br>ABX FMT-CUMS (N=10)<br>ABX FMT-CUMS+Polysaccharide (N=10)<br>ABX FMT-Control (N=10)	18-22 g	male	OFT: center distance↓<br>EPM: open arms time and entries↓<br>TST: immobility time↑<br>FST: immobility time↑	After 1 week of acclimatization and 4 weeks of CUMS	Cecum contents	−80 ℃	16S rRNA gene sequencing-Illumina MiSeq platform	V3-V4	CUMS-depression and control	One-way ANOVA followed by Tukey multiple comparison tests: $p < 0.05$
Yan T et al. 2021[172]	C57BL/6 mice	China	case-control	4 mice/cage	LPS-depression	LPS (N=10)<br>LPS+SCE (N=10)<br>LPS+SCL (N=10)<br>LPS+SCPS (N=10)<br>LPS+SCVO (N=10)<br>Control (N=10)	18–22 g	male	FST: immobility time↑<br>TST: immobility time↑	After 7 days of acclimatization and 14 days of drugs	Fecal samples	_	16S rRNA gene pyrosequencing-<br>Illumina MiSeq platform	V3–V4	LPS-depression and control	One-way ANOVA followed by Tukey multiple comparison tests: $p < 0.05$
Yang C et al. 2017a[173]	C57BL/6 mice	Janpan	case-control	Single	CSDS-depression	CSDS-susceptible (N=8)<br>CSDS-resilient (N=6)<br>Control (N=8)	8-week-old	male	SIT: interaction time↓	After 10 days of CSDS	Fecal samples	−80 ℃	16S rRNA analysis-Terminal restriction fragment length polymorphism (T-RFLP) analysis	_	CSDS-depression susceptible and control	Fisher's exact test: $p < 0.05$

Yang C et al. 2017b[174]	C57BL/6 mice	Japan	case-control	Single	CSDS-depression	CSDS (N=6)<br>CSDS+(R)-ketamine (N=6)<br>CSDS+(S)-ketamine (N=6)<br>Control (N=6)	8-week-old	male	FST: immobility↑<br>TST: immobility↑<br>SPF: sucrose preference↓	The fecal samples were collected 10 days of CSDS and 4 days (day 16) after a single dose of drugs	Fecal samples	−80 ℃	16S rRNA sequencing analysis-Illumina MiSeq platform	V4	CSDS-depression and control	One-way ANOVA: p < 0 05
Yang C et al. 2019[175]	Sprague-Dawley rats<br><br><br>C57BL/6 mice	China	case-control	_	SNI-depression<br><br><br>FMT SNI susceptible-depression	SNI anhedonia susceptible (N=8)<br>SNI anhedonia resilient (N=8)<br>Control (N=8)<br><br>ABX FMT-SNI susceptible (N=10)<br>ABX FMT-SNI resilient (N=10)<br>ABX PBS (N=10)<br>Control (N=10)	2-month-old	male	Body weight↓<br>MWT: withdraw threshold↓<br>SPT: sucrose preference↓<br>TFT: tall flick latency↓<br>FST: immobile time↑<br>TST: immobile time↑	After 7 days of acclimation, then 23 days after spared nerve injury (day 0)<br><br>After 7 days of acclimation, 14 days of antibiotics treatment and 14 dyas of feces transplantation	Fecal samples	−80 ℃	16S rRNA gene sequencing-Illumina Miseq system	V3-V4	SNI-depression susceptible and control<br><br>ABX FMT SNI susceptible-depression and ABX PBS-control	One-way ANOVA followed by post-hoc Tukey's test: p < 0.05
Yang HL et al. 2021[176]	C57BL/6J mice	China	case-control	_	CRS-depression	CRS (N=15)<br>CRS+Dexamethasone (N=15)<br>Control (N=15)<br>Control+Dexamethasone (N=15)	7-week-old	male	SPT: sucrose preference↓<br>FST: immobility time↑<br>OFT: center time↓<br>EPM: closed arms time↑	After 1 week of acclimatization and 5 weeks of CRS along with dexamethasone	Fecal samples	_	16S rRNA gene sequencing-Illumina MiSeq platform	V3-V4	CRS-depression and control	One-way ANOVA followed by Dunnett's multiple comparison test: p < 0.05
Yang J et al. 2021[177]	Sprague-Dawley rats	China	case-control	_	PSD-depression	PSD (N=15)<br>PSD+Exercise Training (N=15)<br>Control (n=15)	230–250 g	male	_	After 7 days of acclimatization and 30 consecutive days of UCS	_	_	_	_	PSD-depression and control	LEfSe: p < 0.05 and LDA > 3.0
Yang Q et al. 2020[178]	C57BL/6 mice	China	case-control	5 mice/cage	CUMS-depression	CUMS (N=8)<br>CUMS+Imipramine (N=8)<br>CUMS+chronic minocycline (N=8)<br>CUMS+acute minocycline (N=8)<br>Control (N=8)	6-8 week-old	male	OFT: total distance↓, center time↓<br>TST: immobility time↑<br>FST: immobility time↑	After 1 week of acclimatization and 6 weeks of CUMS (the last 4 weeks received drugs treatment)	Fecal samples	−80 ℃	16S rRNA gene sequencing-Illumina MiSeq platform	V4-V5	CUMS-depression and control	LEfSe: p < 0.05 and LDA > 2.0
Yang Y et al. 2022[179]	Sprague-Dawley rats	China	case-control	_	MS-postpartum depression	MS (N=8)<br>MS+Lactobacillus casei (N=8)<br>MS+Paroxetine (N=8)<br>Control (N=8)	Postpartum PND28	female	SPT: sugar preference↓<br>EPM: open arms time and entries↓<br>FST: immobility time↑<br>TST: immobility time↑	PND 28 (PND2-PND21 for MS)	Cecal contents	_	qPCR for Enterococcus faecalis, Bifidobacterium, Lactobacillus, Escherichia coli	_	MS-postpartum depression and control	ANOVA: p < 0.05
Yu JB et al. 2019[180]	Sprague-Dawley rats	China	case-control	_	CUS-depression	CUS (N=8)<br>CUS+Fluoxetine (N=8)<br>CUS+Paeoniflorin low-dosage (N=8)<br>CUS+Paeoniflorin high-dosage (N=8)<br>Control (N=8)	180–200 g	male	SPT: sucrose preference↓	After 8 weeks of CUMS (the last 2 weeks received drugs treatment)	Fecal samples	_	16S rRNA gene sequencing-Barcoded pyro sequencing	V3-V4	CUMS-depression and control	Student's t-test: p < 0.05
Yu M et al. 2017[181]	Wistar rats	China	case-control	_	CVS-depression	CVS (N=8)<br>Control (N=8)	8-week-old	male	Body weight↓<br>OFT: rearing and crossing numbers↓<br>SPT: sucrose preference↓	After 4 weeks of CVS	Fecal samples	−80 ℃	16S rRNA gene sequencing-Illumina HiSeq platform	V3-V4	CVS-depression and control	Student's t-test : p < 0.05
Yu M et al. 2020[182]	Wistar rats	China	case-control	_	CVS-depression	CVS (N=8)<br>CVS+Antibiotic (N=8)<br>CVS+CSGS (N=8)<br>CVS+Antibiotic+CSGS (N=8)<br>Control (N=8)	200 ±20 g	male	Body weight↓<br>SPT: sucrose preference↓<br>OFT: rearing number↓, crossing number↓	After 28 days of CVS experiment	Fecal samples	−80 ℃	16S rRNA gene sequencing-IlluminaMiseq platform	V3-V4	CVS-depression and control	One-way ANOVA: p < 0.05

Yu M et al. 2021[183]	Wistar rats	China	case-control	_	CVS-depression	CVS (N=8)<br>Antibiotic (N=8)<br>CVS+Antibiotic (N=8)<br>Control (N=8)	200 ±20 g	male	OFT: number of rearing and crossing↓<br>SPT: sucrose preference↓	After 4 weeks of CVS	Fecal samples	−80 ℃	16S rRNA gene sequencing-Illumina HiSeq platform	V3-V4	CVS-depression and control	One-way ANOVA: $p < 0.05$
Yun SW et al. 2020[184]	C57BL/6J mice	Korea	case-control	_	EC-depression	EC (N=6)<br>EC+L. gasseri NK109 (N=6)<br>Control (N=6)	5-week-old	male	TST: immobility time↑<br>FST: immobility time↑	After 1 week of acclimatization and 5 days of Escherichia coli K1 treatment and 5 days of Lactobacillus gasseri NK109 treatment	Fecal samples	−80◦C	16S rRNA gene sequencing-Illumina iSeq 100 platform	V4	EC-depression and control	Unpaired t–test: $p < 0.05$
Yun SW et al. 2021[185]	C57BL/6J mice	Korea	case-control	3 mice/cage	EC-depression	EC (N=6)<br>EC+L. paracasei NK112 (N=6)<br>Control (N=6)	5-week-old	male	EPM: open arms time and entries↓<br>TST: immobility time↑<br>FST: immobility time↑	After 1 week of acclimatization and 5 days of Escherichia coli K1 treatment and 5 days of Lacticaseibacillus paracasei NK112 treatment	Fecal samples	−80◦C	16S rRNA gene sequencing-Illumina iSeq 100 platform	V4	EC-depression and control	one-way ANOVA with Tukey's multiple comparison test: $p < 0.05$<br>LEfSe: $p < 0.05$ and LDA > 4.0
Zhang F et al. 2020[186]	Sprague-Dawley rats	China	case-control	5 rats/cage	5-Fu-depression	5-Fu treatment (N=10)<br>Control (N=10)	5-week-old	male	Body weight and food intake↓<br>TST: sedentary time↑<br>SPT: sugar preference rate↓	After 1 week of adaption and 4 days of 5-Fu treatment	Fecal samples	−80 ℃	16S rRNA gene sequencing-Illumina NovaSeq platform	V3-V4	5-Fu-depression and control	Wilcoxon's rank-sum test: $p < 0.05$
Zhang J et al. 2020[187]	C57BL/6 mice	Japan	case-control	_	LPS-depression	LPS (N=10)<br>LPS+SDV (N=10)<br>SDV (N=10)<br>Control (N=10)	8-week-old	male	FST: immobility time↑	After 14 days of recovery and LPS intraperitoneally (i.p.) injected	Fecal samples	−80 ℃	16S rRNA gene sequencing-MiSeq according to the Illumina protocol	V1-V2	LPS-depression and control	Two-way ANOVA followed by post-hoc Tukey's multiple comparison tests: $p < 0.05$
Zhang JC et al. 2017[188]	C57BL/6 mice	Janpan	case-control	Single	CSDS-depression	CSDS (N=5)<br>CSDS+MR16-1 (N=5)<br>Control (N=5)	8-week-old	male	SIT: No data<br>SPT: sucrose preference↓<br>FST: immobility time↑<br>TST: immobility time↑	After 10 days of CSDS	Fecal samples	−80 ℃	16S rRNA gene sequencing-Illumina MiSeq platform	V4	CSDS-depression and control	One-way ANOVA followed by the post hoc LSD test: $p < 0.05$
Zhang K et al. 2019[189]	Sprague-Dawley rats	Japan	case-control	_	LH-depression	LH (N=6)<br>non-LH (N=7)<br>Control (N=7)	7-week-old	male	Post-shock test: numbers of escape failures↑	Fresh fecal samples were collected in a blind manner before post-shock stress on day 4	Fecal samples	−80◦C	16S rRNA gene sequencing-	V3-V4	LH-depression and control	One-way analysis of variance, followed by post hoc Fisher's LSD test: $p < 0.05$
Zhang L et al. 2021[190]	Kunming mice	China	case-control	Single	CUMS-depression	CUMS (N=12)<br>CUMS+Fluoxetine (N=12)<br>CUMS+TIV low-dosage (N=12)<br>CUMS+TIV middle-dosage (N=12)<br>CUMS+TIV high-dosage (N=12)<br>Control (N=12)	Adult 16–20 g	male	Body weight:↓<br>TST: immobility time↑<br>SPT: sucrose preference↓	After 1 week of acclimatization and 4 weeks of CUMS (the last 2 weeks received drugs treatment)	Colon contents	−20◦C	16S rRNA gene sequencing-Rhonin's MiSeq platform	V4	CUMS-depression and control	One-way ANOVA: $p < 0.05$
Zhang M et al. 2021[191]	ICR mice	China	case-control	Single	CUMS-depression	CUMS (N=10)<br>CUMS+Imipramine (N=10)<br>CUMS+Alkaloids (N=10)<br>Control (N=10)	20-25 g	male	SPT: sucrose preference↓<br>FST: immobility time↑<br>OFT: crossings and rearings number↓, modifications number↓	After 1 week of acclimatization and 7 weeks of CUMS (the last 4 weeks received drugs treatment)	Cecum contents	−20◦C	16S rRNA gene sequencing-Illumina NovaSeq platform	V3–V4	Depression-like behaviors correlate with gut microbes	LEfSe: $p < 0.05$ and LDA > 2.0<br>Spearman correlation test: $p < 0.05$
Zhang W et al. 2021[192]	Sprague-Dawley rats	China	case-control	Single	CUMS-depression	At weeks 9:<br>CUMS (N=36)<br>Control (N=12)	180–220 g	male	Body weight↓<br>SPT: sucrose preference↓<br>OFT: rearing and crossing numbers↓<br>LDT: dark time↑	After 1 week of acclimatization, 8 weeks of CUMS and 6 weeks of treatment period. Faecal samples were collected at weeks 9 and 15.	Fecal samples	_	At weeks 9:<br>16S rRNA gene sequencing-Illumina HiSeq 2500 platform	V4–V5	CUMS-depression and control at weeks 9 and 15	Kruskal–Wallis H test with Tukey's posthoc tests: $p < 0.05$<br>LEfSe: $p < 0.05$ and LDA > 2.0

						At weeks 15:<br>CUMS (N=6)<br>CUMS+Amitriptyline (N=6)<br>CUMS+Fluoxetine (N=7)<br>Control (N=12)							At weeks 15:<br>Shotgun metagenomics-Illumina HiSeq 4000 platform	NA		
Zhang Y et al. 2021[193]	Sprague-Dawley rats	China	case-control	_	CUMS-depression	CUMS (N=9)<br>CUMS+Fluoxetine (N=9)<br>CUMS+Green tea (N=9)<br>CUMS+low-dose jasmine tea (N=9)<br>CUMS+middle-dose jasmine tea (N=9)<br>CUMS+high-dose jasmine tea ((N=9)<br>Control (N=9)	4 weeks	male	Body weight↓<br>SPT: sugar preference↓<br>FST: immobility time↑<br>OFT: inner/outer/stanging number↓	After 1 week of acclimatization,feces of rats in each group at day 29 were collected	Fecal samples	−80 ℃	16S rRNA gene sequencing-Illumina MiSeq platform	V3-V4	CUMS-depression and control	ANOVA followed by Bonferroni's test: $p < 0.05$
Zhang Y et al. 2019[194]	C57BL/6 mice	China	case-control	_	NLRP3-KO anti-depression<br><br>CUS-depression	NLRP3-KO (N=14)<br>Wild-type (N=14)<br><br>ABX FMT-NLRP3-KO CUS (N=16)<br>ABX FMT-NLRP3-KO Control (N=15)<br>ABX FMT-WT CUS (N=14)<br>ABX FMT-WT Control (N=15)	6–8-week-old	male	NLRP3-KO:<br>FST: immobility time↓<br>TST: immobility time↓<br>OFT：total distance↑, center time↑, center distance↑<br>CUS:<br>SPT: sucrose preference↓<br>FST: immobility time↑<br>TST: immobility time↑	Unspecified	Fecal samples	_	16S rRNA gene sequencing-_	V4-V5	NLRP3-KO anti-depression and wild-type<br><br>FMT-WT CUS and FMT-WT Control	Mann-Whitney test: $p < 0.05$
Zhang Z et al. 2021[195]	C57BL/6 mice	China	case-control	_	Offspring of prenatal IS-depression	Offspring of<br>Prenatal IS (N=12)<br>Control (N=12)	6-8 week-old	female	EPM: open arm time↓, closed arm time↑<br>LDT: dark area time↑<br>SPT: sucrose preference↓<br>FST: immobility time↑	Fecal samples were collected from F1 adult female mice at 6-8 weeks old	Fecal samples	−80◦C	16S rRNA gene sequencing-Illumina MiSeq platform	V3-V4	Prenatal IS-depression and control	LEfSe: $p < 0.05$ and LDA > 2.0
Zhang Z et al. 2020[196]	athymic nude mice (NCr-nu)	China	case-control	5 mice/cage	CRS-depression	CRS+CRC xenografts (N=15)<br>CRS+Fluoxetine+CRC xenografts (N=15)<br>CRS+Xiaoyaosan-low+CRC xenografts (N=15)<br>CRS+Xiaoyaosan-high+CRC xenografts (N=15)<br>Control+CRC xenografts (N=15)	6-7 week-old	male	SPF: sucrose preference↓<br>TST: immobility time↑	After 7 days of acclimatizationm, 14 days of CRS, 42 days of behavioural tests, tumour cell injection, and fluoxetine or xiaoyaosan treatment	Fecal samples	_	16S rRNA gene sequencing-Illumina MiSeq platform	V3-V4	CRS-depression and control	LEfSe: $p < 0.05$ and LDA ≥ 2.0
Zhang Z et al. 2022[197]	C57BL/6 mice	China	case-control	Single	CRS-depression	CRS (N=7)<br>CRS+Hyperforin (N=7)<br>Control (N=7)	7-9 weeks	male	SPT: sucrose preference↓	After 1 week of acclimatization and 10 days of CRS and hyperforin treatment	Fecal samples	Dry ice	Full-length 16S rRNA gene sequencing-PacBio platform	_	CRS-depression and control	Metastat analysis: $p < 0.05$
Zhao B et al. 2020[198]	C57BL/6 mice	China	case-control	_	DSS-depression	DSS (N=12)<br>DSS+Lycopene (N=12)<br>Control (N=12)	8-week-old	male	OFT: path length↓, dwell time↑<br>BMT: marbles buried↑<br>TST: immobility time↑<br>EPM: percentage of open arm entries↓	After 35 days of corresponding diet and 7 days of DSS treatment	Fecal samples	−80◦C	16S rRNA gene sequencing-Illumina MiSeq platform	V3-V4	DSS-depression and control	Kruskal−Wallis test or Wilcoxon rank-sum test: p < 0 05<br>LEfSe: $p < 0.05$ and LDA > 3.0
Zhao F et al. 2021[199]	Sprague-Dawley rats	China	case-control	single	CUMS-depression	Pregnant rat<br>CUMS (N=8)<br>CUMS+LBP (N=8)	200 ±20 g	female	Pregnant rat<br>SPF: sucrose preference↓<br>OFT: horizontal and vertical movements↓	Fresh feces were collected from female rats at 21 days after CUMS procedure, feces of the	Fecal samples	−80◦C	16S rRNA gene sequencing-Illumina MiSeq platform	V3-V4	Pregnant rat<br>CUMS-depression and control	One-way ANOVA followed by Bonferroni multiple comparison test: p < 0 05

				4 rats/cage	Offspring of prenatal CUMS-depression	Control (N=8)<br><br>Offspring of<br>Prenatal CUMS (N=16)<br>Prenatal CUMS+LBP (N=16)<br>Control (N=16)	PND 20	female : male (1:1)	Offspring<br>Body weight↓<br>SPF: sucrose preference↓<br>OFT: horizontal and vertical movements↓<br>TST: immobility time↑	offspring were collected at PND 20					Offspring of Prenatal CUMS-depression and control	
Zhao W et al. 2019[200]	C57BL/6J mice	China	case-control	_	ABX FMT-Alc depression	ABX FMT-Alc (N=14)<br>ABX FMT-Con (N=14)<br>Control (N=12)	6 week-old	male	OFT: center time↓, center distance↓<br>EPM: open arms time and entries↓<br>TST: immobility time↑	After 21 days of antibiotics administration and 13 days of FMT	Fecal samples	−80◦C	16S rRNA gene sequencing-	V3-V5	ABX FMT-Alc depression and ABX FMT-control	Student's unpaired t-test and Mann–Whitney test: p < 0 05
Zhao Z et al. 2020[201]	BALB/c mice	China	case-control	10 mice/cage	Antibiotic-depression	Ceftriaxone (N=20)<br>Control (N=20)	6–8 week-old	male	Body weight↓<br>OFT: center time↓, center distance↓, peripheral time↑, peripheral distance↑<br>TST: activity phase↓, quiescence phase↑	After 1 week of acclimatization and 11 weeks of ceftriaxone sodium solution intragastrically	Fecal samples	−80◦C	16S rRNA gene sequencing-Illumina MiSeq platform	V3-V4	Antibiotic-depression and control	One-way ANOVA or Wilcoxon rank sum test: p < 0 05
Zheng P et al. 2016[202]	Kumming mice	China	case-control	5 mice/cage	GF FMT-depression	GF FMT-MDD (N=63)<br>GF FMT-HC (N=69)	6–8-week-old	male	OFT: proportion of center motion distance↓<br>FST: duration of immobility↑<br>TST: duration of immobility↑	2 weeks post FMT	Fecal samples<br><br>Cecum samples	−80 ℃	16S rRNA gene sequencing-Roche 454 sequencing<br><br>Shotgun metagenomic-Illumina HiSeq2500	V3-V5<br><br>-	FMT-MDD and FMT-HC	Random Forests
Zheng P et al. 2020[203]	Macaca fascicularis monkeys	China	case-control	group	Naturally-occurring depression	Depression (N=6)<br>Control (N=6)	Adult	female	Duration of huddle and sit-alone behaviors↑<br>Duration of amicable and locomotion activities↓	After behavioral tests	Cecum contents	−80 ℃	16S rRNA gene sequencing-Illumina MiSeq platform<br>Metagenomic-Illumina HiSeq X platform	V3-V4<br><br>_	Naturally-occurring depression and control	LEfSe: p < 0.05 and LDA > 2.0
Zhou H et al. 2022[204]	C57BL/6 mice	China	case-control	_	Dcf1 KO-depression	Dcf1KO (N=5)<br>Dcf1KO+B. longum (N=5)<br>Dcf1KO+L. murinus (N=5)<br>Dcf1KO+L. reuteri (N=5)<br>WT (N=5)<br>WT+Fecal of Dcf1 KO mice (N=5)	Adult, 20-25 g	male	SPT: sucrose preference↓<br>FST: immobility time↑<br>TST: immobility time↑	_	Fecal samples	_	16S rRNA gene sequencing-	Unspecified	Dcf1 KO-depression and wild-type	_
Zhu HZ et al. 2019[205]	Sprague-Dawley rats	China	case-control	_	CRS-depression	CRS (N=13)<br>CRS+Xiaoyaosan (N=13)<br>CRS+Fluoxetine (N=13)<br>Control (N=13)	200 ±20 g	male	Body weight↓<br>SPT: sugar preference↓<br>OFT: residence time↓, total distance↓, number of entries↓	After 7 days of acclimatization and 21 days of CRS	Fecal samples	−20 ℃	16S rRNA gene sequencing-Illumina-Misq high-throughput sequencing platform	V3-V4	CRS-depression and control	Metastats: p < 0.05<br>LEfSe: p < 0.05 and LDA > 2.0
Zhu JP et al. 2021[206]	Sprague-Dawley rats	China	case-control	_	CUMS-depression	CUMS (N=12)<br>CUMS+Fluoxetine (N=12)<br>CUMS+low-dose BHT (N=12)<br>CUMS+middle-dose BHT (N=12)<br>CUMS+high-dose BHT (N=12)<br>Control (N=12)	230–250 g	male	SPF: sucrose preference↓<br>OFT: horizontal crossings↓, vertical erections↓	After 5 days of acclimatization and 30 consecutive days of CUMS	Rectum contents	Liquid nitrogen	16S rRNA sequencing analysis-Shanghai Biotree Biotechnology Co., Ltd.	Unspecified	CUMS-depression and control	One-way ANOVA or Kruskal–Wallis H test: p < 0 05

References

1. A. Abildgaard, T. Kern, O. Pedersen, T. Hansen, S. Lund and G. Wegener, *Eur Neuropsychopharmacol* **2021**, *43*, 10-21.

2. Y. Aitbali, S. Ba-M'hamed, N. Elhidar, A. Nafis, N. Soraa and M. Bennis, *Neurotoxicol Teratol* **2018**, *67*, 44-49.

3. H. Amini-Khoei, E. Haghani-Samani, M. Beigi, A. Soltani, G. R. Mobini, S. Balali-Dehkordi, A. Haj-Mirzaian, M. Rafieian-Kopaei, A. Alizadeh, M. R. Hojjati and M. Validi, *Int Immunopharmacol* **2019**, *66*, 242-250.

4. Q. An, C. Li, Y. Chen, Y. Yang, R. Song, L. Zhou, J. Li, A. Tong and Y. Luo, *Pharmacol Biochem Behav* **2020**, *192*, 172910.

5. A. Arslanova, A. Tarasova, A. Alexandrova, V. Novoselova, I. Shaidullov, D. Khusnutdinova, T. Grigoryeva, D. Yarullina, O. Yakovleva and G. Sitdikova, *Life (Basel)* **2021**, *11*,

6. A. Bharwani, M. F. Mian, M. G. Surette, J. Bienenstock and P. Forsythe, *BMC Med* **2017**, *15*, 7.

7. L. C. Bridgewater, C. Zhang, Y. Wu, W. Hu, Q. Zhang, J. Wang, S. Li and L. Zhao, *Sci Rep* **2017**, *7*, 10776.

8. A. Burokas, S. Arboleya, R. D. Moloney, V. L. Peterson, K. Murphy, G. Clarke, C. Stanton, T. G. Dinan and J. F. Cryan, *Biol Psychiatry* **2017**, *82*, 472-487.

9. C. Cao, M. Liu, S. Qu, R. Huang, M. Qi, Z. Zhu, J. Zheng, Z. Chen, Z. Wang, Z. Han, Y. Zhu, F. Huang and J.-A. Duan, *J Ethnopharmacol* **2020**, *261*, 113055.

10. A. Chakraborti, C. Graham, S. Chehade, B. Vashi, A. Umfress, P. Kurup, B. Vickers, H. A. Chen, R. Telange, T. Berryhill, W. Van Der Pol, M. Powell, S. Barnes, C. Morrow, D. L. Smith, M. S. Mukhtar, S. Watts, G. Kennedy and J. Bibb, *Front Neurosci* **2021**, *15*, 669410.

11. L. Chen, W. Qing, Z. Yi, G. Lin, Q. Peng and F. Zhou, *Front Nutr* **2021**, *8*, 701760.

12. P. Chen, M. Hei, L. Kong, Y. Liu, Y. Yang, H. Mu, X. Zhang, S. Zhao and J. Duan, *Food Funct* **2019**, *10*, 8161-8171.

13. T. Chen, R. Wang, Z. Duan, X. Yuan, Y. Ding, Z. Feng, F. Bu, L. Liu, Q. Wang, J. Zhou, L. Zhu, Q. Ni, G. Shi and Y. Chen, *Front Cell Infect Microbiol* **2021**, *11*, 723856.

14. X. Chen, S. Meng, S. Li, L. Zhang, L. Wu, H. Zhu and Y. Zhang, *Biomed Res Int* **2021**, *2021*, 5516604.

15. X. Chen, S. Meng, Y. Yu, S. Li, L. Wu and Y. Zhang, *Int J Occup Med Environ Health* **2022**, *35*,

16. Y. Chen, M. Wan, Y. Zhong, T. Gao, Y. Zhang, F. Yan, D. Huang, Y. Wu and Z. Weng, *Mol Nutr Food Res* **2021a**, *65*, e2100146.

17. Y. Chen, N. Xiao, Y. Chen, X. Chen, C. Zhong, Y. Cheng, B. Du and P. Li, *Food Res Int* **2021b**, *150*, 110808.

18. D. Cheng, H. Chang, S. Ma, J. Guo, G. She, F. Zhang, L. Li, X. Li and Y. Lu, *Molecules* **2018**, *23*,

19. R. Cheng, W. Xu, J. Wang, Z. Tang and M. Zhang, *Biochem Biophys Res Commun* **2021**, *566*, 170-176.

20. G. Chevalier, E. Siopi, L. Guenin-Macé, M. Pascal, T. Laval, A. Rifflet, I. G. Boneca, C. Demangel, B. Colsch, A. Pruvost, E. Chu-Van, A. Messager, F. Leulier, G. Lepousez, G. Eberl and P.-M. Lledo, *Nat Commun* **2020**, *11*, 6363.

21. L. Chi, I. Khan, Z. Lin, J. Zhang, M. Y. S. Lee, W. Leong, W. L. W. Hsiao and Y. Zheng, *Phytomedicine* **2020**, *67*, 153157.

22. J. Choi, H. Kwon, Y.-K. Kim and P.-L. Han, *Mol Neurobiol* **2022**, *59*, 2715-2728.

23. V. Daugé, C. Philippe, M. Mariadassou, O. Rué, J.-C. Martin, M.-N. Rossignol, N. Dourmap, L. Svilar, F. Tourniaire, M. Monnoye, D. Jardet, M. Bangratz, S. Holowacz, S. Rabot and L. Naudon, *Front Behav Neurosci* **2020**, *14*, 581296.

24. Y. Deng, M. Zhou, J. Wang, J. Yao, J. Yu, W. Liu, L. Wu, J. Wang and R. Gao, *Gut Microbes* **2021**, *13*,

25. J. Dhaliwal, D. P. Singh, S. Singh, A. K. Pinnaka, R. K. Boparai, M. Bishnoi, K. K. Kondepudi and K. Chopra, *J Appl Microbiol* **2018**, *125*, 257-269.

26. Y. Ding, F. Bu, T. Chen, G. Shi, X. Yuan, Z. Feng, Z. Duan, R. Wang, S. Zhang, Q. Wang, J. Zhou and Y. Chen, *Appl Microbiol Biotechnol* **2021**, *105*, 8411-8426.

27. S. Diviccaro, S. Giatti, F. Borgo, M. Barcella, E. Borghi, J. L. Trejo, L. M. Garcia-Segura and R. C. Melcangi, *Psychoneuroendocrinology* **2019**, *99*, 206-215.

28. F. Donoso, S. Egerton, T. F. S. Bastiaanssen, P. Fitzgerald, S. Gite, F. Fouhy, R. P. Ross, C. Stanton, T. G. Dinan and J. F. Cryan, *Psychoneuroendocrinology* **2020**, *116*, 104673.

29. H.-X. Du, Y. Liu, L.-G. Zhang, C.-S. Zhan, J. Chen, M. Zhang, X.-G. Chen, L. Zhang and C.-Z. Liang, *Prostate* **2020**, *80*, 663-673.

30. J. Duan, Y. Huang, X. Tan, T. Chai, J. Wu, H. Zhang, Y. Li, X. Hu, P. Zheng, P. Ji, L. Zhao, D. Yang, L. Fang, J. Song and P. Xie, *Transl Psychiatry* **2021**, *11*, 303.

31. S. Egerton, F. Donoso, P. Fitzgerald, S. Gite, F. Fouhy, J. Whooley, T. G. Dinan, J. F. Cryan, S. C. Culloty, R. P. Ross and C. Stanton, *Nutr Neurosci* **2020**, *25*, 356-378.

32. S. El Aidy, A. S. Ramsteijn, F. Dini-Andreote, R. van Eijk, D. J. Houwing, J. F. Salles and J. D. A. Olivier, *Front Cell Neurosci* **2017**, *11*, 222.

33. L. Fan, Y. Peng, J. Wang, P. Ma, L. Zhao and X. Li, *Phytomedicine* **2021**, *83*, 153471.

34. P. Farshim, G. Walton, B. Chakrabarti, I. Givens, D. Saddy, I. Kitchen, J. R Swann and A. Bailey, *Sci Rep* **2016**, *6*, 21958.

35. Y. Feng, X. Gao, M. Meng, H. Xue and X. Qin, *J Ethnopharmacol* **2020**, *256*, 112806.

36. Z. Feng, X. Ma, S. Meng, H. Wang, X. Zhou, M. Shi and J. Zhao, *Evid Based Complement Alternat Med* **2020**, *2020*, 3290450.

37. S. Forouzan, K. L. Hoffman and T. A. Kosten, *Psychopharmacology (Berl)* **2021**, *238*, 281-292.

38. K. Gao, A. Farzi, X. Ke, Y. Yu, C. Chen, S. Chen, T. Yu, H. Wang and Y. Li, *Food Funct* **2022**, *13*, 957-969.

39. X. Gao, Y. Feng, H. Xue, M. Meng and X. Qin, *Journal of Liquid Chromatography & Related Technologies* **2020**, *43*, 494-507.

40. X. Gong, C. Huang, X. Yang, J. Chen, J. Pu, Y. He and P. Xie, *Front Neurosci* **2021**, *15*, 701355.

41. F. Gu, Y. Wu, Y. Liu, M. Dou, Y. Jiang and H. Liang, *Food Funct* **2020**, *11*, 6148-6157.

42. X. Gu, S. Zhang, W. Ma, Q. Wang, Y. Li, C. Xia, Y. Xu, T. Zhang, L. Yang and M. Zhou, *Front Microbiol* **2022**, *13*, 778512.

43. F. Guida, F. Turco, M. Iannotta, D. De Gregorio, I. Palumbo, G. Sarnelli, A. Furiano, F. Napolitano, S. Boccella, L. Luongo, M. Mazzitelli, A. Usiello, F. De Filippis, F. A. Iannotti, F. Piscitelli, D. Ercolini, V. de Novellis, V. Di Marzo, R. Cuomo and S. Maione, *Brain Behav Immun* **2018**, *67*, 230-245.

44. Y. Guo, J. Xie, X. Li, Y. Yuan, L. Zhang, W. Hu, H. Luo, H. Yu and R. Zhang, *Front Pharmacol* **2018**, *9*, 1126.

45. Y. Guo, J.-P. Xie, K. Deng, X. Li, Y. Yuan, Q. Xuan, J. Xie, X.-M. He, Q. Wang, J.-J. Li and H.-R. Luo, *Front Behav Neurosci* **2019**, *13*, 126.

46. S.-K. Han and D. H. Kim, *J Microbiol Biotechnol* **2019**, *29*, 1369-1374.

47. S.-K. Han, M.-K. Joo, J.-K. Kim, W. Jeung, H. Kang and D.-H. Kim, *Nutrients* **2020a**, *12*,

48. S.-K. Han, J.-K. Kim, M.-K. Joo, K.-E. Lee, S.-W. Han and D. H. Kim, *J Microbiol Biotechnol* **2020b**, *30*, 1222-1226.

49. S.-K. Han, J.-K. Kim, H.-S. Park, Y.-J. Shin and D.-H. Kim, *Chin Med* **2021**, *16*, 77.

50. W. Hao, J. Wu, N. Yuan, L. Gong, J. Huang, Q. Ma, H. Zhu, H. Gan, X. Da, L. Deng, X. Li and J. Chen, *Front Pharmacol* **2021**, *12*, 619103.

51. W.-Z. Hao, Q.-Y. Ma, G. Tao, J.-Q. Huang and J.-X. Chen, *Food Funct* **2021**, *12*, 12550-12564.

52. A. M. Hassan, G. Mancano, K. Kashofer, E. E. Fröhlich, A. Matak, R. Mayerhofer, F. Reichmann, M. Olivares, A. M. Neyrinck, N. M. Delzenne, S. P. Claus and P. Holzer, *Nutr Neurosci* **2019**, *22*, 877-893.

53. F. Huang, X. Liu, S. Xu, S. Hu, S. Wang, D. Shi, K. Wang, Z. Wang, Q. Lin, S. Li, S. Zhao, K. Jin, C. Wang, L. Chen and F. Wang, *Front Nutr* **2021**, *8*, 805465.

54. N. Huang, D. Hua, G. Zhan, S. Li, B. Zhu, R. Jiang, L. Yang, J. Bi, H. Xu, K. Hashimoto, A. Luo and C. Yang, *Pharmacol Biochem Behav* **2019**, *176*,

55. Y.-J. Huang, L.-X. C. Choong, S. Panyod, Y.-E. Lin, H.-S. Huang, K.-H. Lu, W.-K. Wu and L.-Y. Sheen, *Phytother Res* **2021**, *35*, 5133-5142.

56. Y.-Y. Huang, Y.-P. Wu, X.-Z. Jia, J. Lin, L.-F. Xiao, D.-M. Liu and M.-H. Liang, *Food Funct* **2022**, *13*, 411-424.

57. A. Inserra, J. M. Choo, M. D. Lewis, G. B. Rogers, M.-L. Wong and J. Licinio, *Sci Rep* **2019**, *9*, 6456.

58. H.-M. Jang, K.-E. Lee and D.-H. Kim, *Nutrients* **2019**, *11*,

59. S. Ji, S. Han, L. Yu, L. Du, Y. You, J. Chen, M. Wang, S. Wu, S. Li, X. Sun, R. Luo and X. Zhao, *Phytomedicine* **2022**, *98*, 153940.

60. W. Jiang, L. Gong, F. Liu, Y. Ren and J. Mu, *Front Cell Infect Microbiol* **2021**, *11*, 663967.

61. Y. Jiang, Y. Liu, M. Gao, M. Xue, Z. Wang and H. Liang, *Food Funct* **2020**, *11*, 378-391.

62. L. Jianguo, J. Xueyang, W. Cui, W. Changxin and Q. Xuemei, *Transl Psychiatry* **2019**, *9*, 40.

63. Y. Kamimura, E. Kuwagaki, S. Hamano, M. Kobayashi, Y. Yamada, Y. Takahata, W. Yoshimoto, H. Morimoto, T. Yasukawa, Y. Uozumi and K. Nagasawa, *Life Sci* **2021**, *282*, 119821.

64. C. Karen, D. J. H. Shyu and K. E. Rajan, *Front Neurosci* **2021**, *15*, 719933.

65. J. R. Kelly, Y. Borre, C. O' Brien, E. Patterson, S. El Aidy, J. Deane, P. J. Kennedy, S. Beers, K. Scott, G. Moloney, A. E. Hoban, L. Scott, P. Fitzgerald, P. Ross, C. Stanton, G. Clarke, J. F. Cryan and T. G. Dinan, *J Psychiatr Res* **2016**, *82*, 109-118.

66. K. M. Kemp, J. Colson, R. G. Lorenz, C. L. Maynard and J. S. Pollock, *Am J Physiol Regul Integr Comp Physiol* **2021**, *320*, R663-R674.

67. J.-K. Kim, K.-E. Lee, S.-A. Lee, H.-M. Jang and D.-H. Kim, *Front Immunol* **2020**, *11*, 273.

68. J.-K. Kim, S.-K. Han, M.-K. Joo and D.-H. Kim, *Sci Rep* **2021**, *11*, 6094.

69. J. K. Knudsen, T. Y. Michaelsen, C. Bundgaard-Nielsen, R. E. Nielsen, S. Hjerrild, P. Leutscher, G. Wegener and S. Sørensen, *Sci Rep* **2021**, *11*, 21869.

70. A. Kosuge, K. Kunisawa, S. Arai, Y. Sugawara, K. Shinohara, T. Iida, B. Wulaer, T. Kawai, H. Fujigaki, Y. Yamamoto, K. Saito, T. Nabeshima and A. Mouri, *Brain Behav Immun* **2021**, *96*, 200-211.

71. D. Kuti, Z. Winkler, K. Horváth, B. Juhász, M. Paholcsek, A. Stágel, G. Gulyás, L. Czeglédi, S. Ferenczi and K. J. Kovács, *Brain Behav Immun* **2020**, *84*, 218-228.

72. W.-D. Lai, T.-H. Tung, C.-Y. Teng, C.-H. Chang, Y.-C. Chen, H.-Y. Huang, H.-C. Lee and S.-Y. Huang, *Food Funct* **2022**, *13*, 2662-2680.

73. S. Leclercq, T. Le Roy, S. Furgiuele, V. Coste, L. B. Bindels, Q. Leyrolle, A. M. Neyrinck, C. Quoilin, C. Amadieu, G. Petit, L. Dricot, V. Tagliatti, P. D. Cani, K. Verbeke, J.-M. Colet, P. Stärkel, P. de Timary and N. M. Delzenne, *Cell Rep* **2020**, *33*, 108238.

74. H.-C. Lee, Y.-C. Lo, S.-C. Yu, T.-H. Tung, I. H. Lin and S.-Y. Huang, *Int J Food Sci Nutr* **2020**, *71*, 440-452.

75. H. Li, P. Wang, L. Huang, P. Li and D. Zhang, *Neurogastroenterol Motil* **2019**, *31*, e13677.

76. H. Li, Y. Xiang, Z. Zhu, W. Wang, Z. Jiang, M. Zhao, S. Cheng, F. Pan, D. Liu, R. C. M. Ho and C. S. H. Ho, *J Neuroinflammation* **2021**, *18*, 254.

77. N. Li, Q. Wang, Y. Wang, A. Sun, Y. Lin, Y. Jin and X. Li, *Front Behav Neurosci* **2018**, *12*, 266.

78. N. Li, Q. Wang, Y. Wang, A. Sun, Y. Lin, Y. Jin and X. Li, *Stress* **2019**, *22*, 592-602.

79. P. Li, W. Huang, Y.-N. Yan, W. Cheng, S. Liu, Y. Huang, W. Chen, Y.-P. Chen, Y. Gao, W. Lu, Y. Xu and X. Meng, *Med Sci Monit* **2021**, *27*, e929027.

80. Q. Li, L. Li, X. Niu, C. Tang, H. Wang, J. Gao and J. Hu, *Neuroreport* **2021**, *32*, 686-693.

81. Y. Li, Y. Peng, P. Ma, H. Yang, H. Xiong, M. Wang, C. Peng, P. Tu and X. Li, *Front Pharmacol* **2018**, *9*, 967.

82. E. Y. Lim, E. J. Song, J. G. Kim, S. Y. Jung, S. Y. Lee, H. S. Shin, Y. D. Nam and Y. T. Kim, *Benef Microbes* **2021**, *12*, 503-516.

83. S. Lin, Q. Li, S. Jiang, Z. Xu, Y. Jiang, L. Liu, J. Jiang, Y. Tong and P. Wang, *J Ethnopharmacol* **2021**, *268*, 113608.

84. Q. F. Liu, H.-M. Kim, S. Lim, M.-J. Chung, C.-Y. Lim, B.-S. Koo and S.-S. Kang, *Daru* **2020**, *28*, 181-189.

85. X. Liu, T. Teng, X. Li, L. Fan, Y. Xiang, Y. Jiang, K. Du, Y. Zhang, X. Zhou and P. Xie, *Front Cell Infect Microbiol* **2021a**, *11*, 697640.

86. X. Liu, M. Lv, Y. Wang, P. Qu, S. Li, Z. Yu and X. Qin, *Phytomedicine* **2021b**, *87*, 153581.

87. Z. Liu, L. Li, S. Ma, J. Ye, H. Zhang, Y. Li, A. T. Sair, J. Pan, X. Liu, X. Li, S. Yan and X. Liu, *J Agric Food Chem* **2020**, *68*, 13697-13710.

88. X. Luo, X. Huang, Z. Luo, Z. Wang, G. He, Y. Tan, B. Zhang, H. Zhou, P. Li, T. Shen, X. Yu and X. Yang, *Ecotoxicol Environ Saf* **2021**, *228*, 112980.

89. M. Lv, Y. Wang, P. Qu, S. Li, Z. Yu, X. Qin and X. Liu, *J Ethnopharmacol* **2021**, *276*, 114167.

90. W.-J. Lv, X.-L. Wu, W.-Q. Chen, Y.-F. Li, G.-F. Zhang, L.-M. Chao, J.-H. Zhou, A. Guo, C. Liu and S.-N. Guo, *Oxid Med Cell Longev* **2019**, *2019*, 7902874.

91. W.-J. Lv, C. Liu, L.-Z. Yu, J.-H. Zhou, Y. Li, Y. Xiong, A. Guo, L.-M. Chao, Q. Qu, G.-W. Wei, X.-G. Tang, Y.-L. Yin and S.-N. Guo, *Oxid Med Cell Longev* **2020**, *2020*, 1241894.

92. W. Ma, J. Song, H. Wang, F. Shi, N. Zhou, J. Jiang, Y. Xu, L. Zhang, L. Yang and M. Zhou, *Life Sci* **2019**, *225*, 88-97.

93. I. A. Marin, J. E. Goertz, T. Ren, S. S. Rich, S. Onengut-Gumuscu, E. Farber, M. Wu, C. C. Overall, J. Kipnis and A. Gaultier, *Sci Rep* **2017**, *7*, 43859.

94. D. Martín-Hernández, J. R. Caso, Á. G. Bris, S. R. Maus, J. L. M. Madrigal, B. García-Bueno, K. S. MacDowell, L. Alou, M. L. Gómez-Lus and J. C. Leza, *Neuropharmacology* **2016**, *103*, 122-133.

95. Y. Matsuda, N. Ozawa, T. Shinozaki, K.-I. Wakabayashi, K. Suzuki, Y. Kawano, I. Ohtsu and Y. Tatebayashi, *Transl Psychiatry* **2020**, *10*, 170.

96. K. D. McGaughey, T. Yilmaz-Swenson, N. M. Elsayed, D. A. Cruz, R. M. Rodriguiz, M. D. Kritzer, A. V. Peterchev, J. Roach, W. C. Wetsel and D. E. Williamson, *Sci Rep* **2019**, *9*, 3281.

97. E. M. Medina-Rodriguez, D. Madorma, G. O'Connor, B. L. Mason, D. Han, S. K. Deo, M. Oppenheimer, C. B. Nemeroff, M. H. Trivedi, S. Daunert and E. Beurel, *Am J Psychiatry* **2020**, *177*, 974-990.

98. C. Meng, S. Feng, Z. Hao, C. Dong and H. Liu, *Psychoneuroendocrinology* **2022**, *136*, 105620.

99. A. Moya-Pérez, A. Perez-Villalba, A. Benítez-Páez, I. Campillo and Y. Sanz, *Brain Behav Immun* **2017**, *65*, 43-56.

100. E. Murray, R. Sharma, K. B. Smith, K. D. Mar, R. Barve, M. Lukasik, A. F. Pirwani, E. Malette-Guyon, S. Lamba, B. J. Thomas, H. Sadeghi-Emamchaie, J. Liang, J.-F. Mallet, C. Matar and N. Ismail, *Brain Behav Immun* **2019**, *81*, 198-212.

101. S. M. O'Mahony, K.-A. McVey Neufeld, R. V. Waworuntu, M. M. Pusceddu, S. Manurung, K. Murphy, C. Strain, M. C. Laguna, V. L. Peterson, C. Stanton, B. M. Berg, T. G. Dinan and J. F. Cryan, *Eur J Neurosci* **2020**, *51*, 1042-1058.

102. A. Osman, S. Zuffa, G. Walton, E. Fagbodun, P. Zanos, P. Georgiou, I. Kitchen, J. Swann and A. Bailey, *iScience* **2021**, *24*, 103048.

103. K. A. Partrick, A. M. Rosenhauer, J. Auger, A. R. Arnold, N. M. Ronczkowski, L. M. Jackson, M. N. Lord, S. M. Abdulla, B. Chassaing and K. L. Huhman, *Sci Rep* **2021**, *11*, 3763.

104. E. Patterson, P. M. Ryan, N. Wiley, I. Carafa, E. Sherwin, G. Moloney, E. Franciosi, R. Mandal, D. S. Wishart, K. Tuohy, R. P. Ross, J. F. Cryan, T. G. Dinan and C. Stanton, *Sci Rep* **2019**, *9*, 16323.

105. J. Pearson-Leary, C. Zhao, K. Bittinger, D. Eacret, S. Luz, A. S. Vigderman, G. Dayanim and S. Bhatnagar, *Mol Psychiatry* **2020**, *25*, 1068-1079.

106. Y. Pu, Y. Tan, Y. Qu, L. Chang, S. Wang, Y. Wei, X. Wang and K. Hashimoto, *Brain Behav Immun* **2021**, *94*, 318-326.

107. M. M. Pusceddu, S. El Aidy, F. Crispie, O. O'Sullivan, P. Cotter, C. Stanton, P. Kelly, J. F. Cryan and T. G. Dinan, *PLoS One* **2015**, *10*, e0139721.

108. Y. Qiao, J. Zhao, C. Li, M. Zhang, L. Wei, X. Zhang, O. Kurskaya, H. Bi and T. Gao, *Ann Transl Med* **2020**, *8*, 942.

109. X. Qiu, G. Wu, L. Wang, Y. Tan and Z. Song, *Ann Transl Med* **2021**, *9*, 366.

110. W. Qu, S. Liu, W. Zhang, H. Zhu, Q. Tao, H. Wang and H. Yan, *Food Funct* **2019**, *10*, 5886-5897.

111. Y. Qu, C. Yang, Q. Ren, M. Ma, C. Dong and K. Hashimoto, *Sci Rep* **2017**, *7*, 15725.

112. Y. Qu, K. Zhang, Y. Pu, L. Chang, S. Wang, Y. Tan, X. Wang, J. Zhang, T. Ohnishi, T. Yoshikawa and K. Hashimoto, *J Affect Disord* **2020**, *272*, 66-76.

113. Y. Qu, C. Su, Q. Zhao, A. Shi, F. Zhao, L. Tang, D. Xu, Z. Xiang, Y. Wang, Y. Wang, J. Pan and Y. Yu, *Front Pharmacol* **2022**, *13*, 837543.

114. J. Rao, R. Xie, L. Lin, J. Jiang, L. Du, X. Zeng, G. Li, C. Wang and Y. Qiao, *Eur J Neurosci* **2021**, *53*, 3598-3611.

115. P. Ray, U. Pandey, D. Das and P. Aich, *Dig Dis Sci* **2021**, *66*, 3776-3791.

116. R. C. Robertson, C. Seira Oriach, K. Murphy, G. M. Moloney, J. F. Cryan, T. G. Dinan, R. Paul Ross and C. Stanton, *Brain Behav Immun* **2017**, *59*, 21-37.

117. J. M. Rosa, F. L. Pazini, A. Camargo, I. A. V. Wolin, G. Olescowicz, L. B. Eslabão, O. B. Romero, E. C. Winkelmann-Duarte and A. L. S Rodrigues, *Behav Brain Res* **2020**, *393*, 112791.

118. A. K. Schmidtner, D. A. Slattery, J. Gläsner, A. Hiergeist, K. Gryksa, V. A. Malik, J. Hellmann-Regen, I. Heuser, T. C. Baghai, A. Gessner, R. Rupprecht, B. Di Benedetto and I. D. Neumann, *Transl Psychiatry* **2019**, *9*, 223.

119. B. Shan, Z. Ai, S. Zeng, Y. Song, J. Song, Q. Zeng, Z. Liao, T. Wang, C. Huang and D. Su, *Psychoneuroendocrinology* **2020**, *117*, 104699.

120. S. Shao, R. Jia, L. Zhao, Y. Zhang, Y. Guan, H. Wen, J. Liu, Y. Zhao, Y. Feng, Z. Zhang, Q. Ji, Q. Li and Y. Wang, *Phytomedicine* **2021**, *88*, 153606.

121. L. Sheng, Y. Wang, A. Jiang, Y. Zhou, H. Zhou and F. M. El-Demerdash, *Journal of Food Quality* **2021**, *2021*, 1-8.

122. E. Siopi, G. Chevalier, L. Katsimpardi, S. Saha, M. Bigot, C. Moigneu, G. Eberl and P.-M. Lledo, *Cell Rep* **2020**, *30*,

123. J. Song, W. Ma, X. Gu, L. Zhao, J. Jiang, Y. Xu, L. Zhang, M. Zhou and L. Yang, *J Transl Med* **2019a**, *17*, 224.

124. J. Song, N. Zhou, W. Ma, X. Gu, B. Chen, Y. Zeng, L. Yang and M. Zhou, *Food Funct* **2019b**, *10*, 2947-2957.

125. X. Song, W. Wang, S. Ding, X. Liu, Y. Wang and H. Ma, *J Affect Disord* **2021**, *290*, 353-363.

126. W. N. Sovijit, W. E. Sovijit, S. Pu, K. Usuda, R. Inoue, G. Watanabe, H. Yamaguchi and K. Nagaoka, *Neurosci Res* **2019**, *168*, 76-82.

127. L. Sun, H. Zhang, Y. Cao, C. Wang, C. Zhao, H. Wang, G. Cui, M. Wang, Y. Pan, Y. Shi and Y. Nie, *Int J Med Sci* **2019a**, *16*, 1260-1270.

128. L. Sun, L. Ma, H. Zhang, Y. Cao, C. Wang, N. Hou, N. Huang, K. M. von Deneen, C. Zhao, Y. Shi, Y. Pan, M. Wang, G. Ji and Y. Nie, *Theranostics* **2019b**, *9*, 721-733.

129. X. Sun, H.-F. Zhang, C.-L. Ma, H. Wei, B.-M. Li and J. Luo, *Can J Infect Dis Med Microbiol* **2021**, *2021*, 6613903.

130. Y. Sun, W. Geng, Y. Pan, J. Wang, P. Xiao and Y. Wang, *Food Funct* **2019**, *10*, 925-937.

131. Y. Sun, T. Yan, G. Gong, Y. Li, J. Zhang, B. Wu, K. Bi and Y. Jia, *Int Immunopharmacol* **2020**, *89*, 107029.

132. K. Suzuki, K. Nakamura, Y. Shimizu, Y. Yokoi, S. Ohira, M. Hagiwara, Y. Wang, Y. Song, T. Aizawa and T. Ayabe, *Sci Rep* **2021**, *11*, 9915.

133. J. K. Szyszkowicz, A. Wong, H. Anisman, Z. Merali and M.-C. Audet, *Brain Behav Immun* **2017**, *66*, 45-55.

134. E. Takahashi and E. Ono, *Biochem Biophys Rep* **2021a**, *28*, 101152.

135. E. Takahashi and E. Ono, *Physiol Behav* **2021b**, *243*, 113623.

136. T. Teng, G. Clarke, M. Maes, Y. Jiang, J. Wang, X. Li, B. Yin, Y. Xiang, L. Fan, X. Liu, J. Wang, S. Liu, Y. Huang, J. Licinio, X. Zhou and P. Xie, *Mol Psychiatry* **2021**,

137. P. Tian, R. Zou, L. Song, X. Zhang, B. Jiang, G. Wang, Y.-K. Lee, J. Zhao, H. Zhang and W. Chen, *Food Funct* **2019a**, *10*, 7588-7598.

138. P. Tian, G. Wang, J. Zhao, H. Zhang and W. Chen, *J Nutr Biochem* **2019b**, *66*, 43-51.

139. P. Tian, K. J. O'Riordan, Y.-K. Lee, G. Wang, J. Zhao, H. Zhang, J. F. Cryan and W. Chen, *Neurobiol Stress* **2020**, *12*, 100216.

140. P. Tian, H. Zhu, X. Qian, Y. Chen, Z. Wang, J. Zhao, H. Zhang, G. Wang and W. Chen, *Front Immunol* **2021**, *12*, 755481.

141. X.-Y. Tian, J.-W. Xing, Q.-Q. Zheng and P.-F. Gao, *Front Cell Infect Microbiol* **2021**, *11*, 694443.

142. S. Tillmann, A. Abildgaard, G. Winther and G. Wegener, *Psychopharmacology (Berl)* **2019**, *236*, 1445-1457.

143. T.-H. Tung, Y.-T. Tung, I. H. Lin, C.-K. Shih, N. T. K. Nguyen, A. Shabrina and S.-Y. Huang, *Biomolecules* **2019**, *9*,

144. L. Wang, Y. Sun, T. Zhao, Y. Li, X. Zhao, L. Zhang, L. Wu, L. Zhang, T. Zhang, G. Wei, A. Altamirano, Y. Tong and Z. Yan, *Planta Med* **2020**, *86*, 172-179.

145. L. Wang, X. Wu, Y. Ma, X. Li, J. Zhang and L. Zhao, *Food Funct* **2021**, *12*, 4995-5006.

146. P. Wang, K. Tu, P. Cao, Y. Yang, H. Zhang, X.-T. Qiu, M.-M. Zhang, X.-J. Wu, H. Yang and T. Chen, *Mol Brain* **2021**, *14*, 49.

147. Q. Wang, M. Jia, Y. Zhao, Y. Hui, J. Pan, H. Yu, S. Yan, X. Dai, X. Liu and Z. Liu, *J Agric Food Chem* **2019**, *67*, 12441-12451.

148. R. Wang, T. Chen, Q. Wang, X.-M. Yuan, Z.-L. Duan, Z.-Y. Feng, Y. Ding, F. Bu, G.-P. Shi and Y.-G. Chen, *Drug Des Devel Ther* **2021**, *15*, 2999-3016.

149. S. Wang, T. Ishima, J. Zhang, Y. Qu, L. Chang, Y. Pu, Y. Fujita, Y. Tan, X. Wang and K. Hashimoto, *J Neuroinflammation* **2020a**, *17*, 241.

150. S. Wang, Y. Qu, L. Chang, Y. Pu, K. Zhang and K. Hashimoto, *J Affect Disord* **2020b**, *260*, 448-457.

151. S. Wang, T. Ishima, Y. Qu, J. Shan, L. Chang, Y. Wei, J. Zhang, Y. Pu, Y. Fujita, Y. Tan, X. Wang, L. Ma, X. Wan, B. D. Hammock and K. Hashimoto, *J Affect Disord* **2021**, *292*, 565-573.

152. Y. Wang, R. Jiang, Z. Wu, L. Zhou, J. Xu, C. Huang, L. Yang, B. Zhu, E. Yan, C. Liu and C. Yang, *Pharmacol Biochem Behav* **2021**, *207*, 173226.

153. A. K. Warda, K. Rea, P. Fitzgerald, C. Hueston, E. Gonzalez-Tortuero, T. G. Dinan and C. Hill, *Behav Brain Res* **2019**, *362*, 213-223.

154. C.-L. Wei, S. Wang, J.-T. Yen, Y.-F. Cheng, C.-L. Liao, C.-C. Hsu, C.-C. Wu and Y.-C. Tsai, *Brain Res* **2019**, *1711*, 202-213.

155. L. Wei, Y. Li, W. Tang, Q. Sun, L. Chen, X. Wang, Q. Liu, S. Yu, S. Yu, C. Liu and X. Ma, *Front Physiol* **2019**, *10*, 1228.

156. S. Westfall, F. Caracci, M. Estill, T. Frolinger, L. Shen and G. M. Pasinetti, *Front Immunol* **2021**, *12*, 670500.

157. M. L. Wong, A. Inserra, M. D. Lewis, C. A. Mastronardi, L. Leong, J. Choo, S. Kentish, P. Xie, M. Morrison, S. L. Wesselingh, G. B. Rogers and J. Licinio, *Mol Psychiatry* **2016**, *21*, 797-805.

158. F. Wu, X. Guo, M. Zhang, Z. Ou, D. Wu, L. Deng, Z. Lu, J. Zhang, G. Deng, S. Chen, S. Li, J. Yi and Y. Peng, *Anaerobe* **2020**, *61*, 102138.

159. J. Wu, J. Li, C. Gaurav, U. Muhammad, Y. Chen, X. Li, J. Chen and Z. Wang, *Gen Psychiatr* **2021**, *34*, e100529.

160. J. Wu, T. Chai, H. Zhang, Y. Huang, S. W. Perry, Y. Li, J. Duan, X. Tan, X. Hu, Y. Liu, J. Pu, H. Wang, J. Song, X. Jin, P. Ji, P. Zheng and P. Xie, *Transl Psychiatry* **2022**, *12*, 74.

161. M. Wu, T. Tian, Q. Mao, T. Zou, C.-J. Zhou, J. Xie and J.-J. Chen, *Transl Psychiatry* **2020**, *10*, 350.

162. J. Xia, L. Gu, Y. Guo, H. Feng, S. Chen, J. Jurat, W. Fu and D. Zhang, *Front Cell Infect Microbiol* **2021**, *11*, 627608.

163. Q. Xiao, R. Shu, C. Wu, Y. Tong, Z. Xiong, J. Zhou, C. Yu, X. Xie and Z. Fu, *J Affect Disord* **2020**, *276*, 476-486.

164. R. Xie, P. Jiang, L. Lin, B. Yu, C. Wang, Y. Pan, J. Rao, W. Wei and Y. Qiao, *FASEB J* **2020a**, *34*, 8310-8325.

165. R. Xie, P. Jiang, L. Lin, J. Jiang, B. Yu, J. Rao, H. Liu, W. Wei and Y. Qiao, *J Psychiatr Res* **2020b**, *122*, 70-78.

166. Y. Xie, L. Song, J. Yang, T. Tao, J. Yu, J. Shi and X. Jin, *Life Sci Alliance* **2021**, *4*,

167. J. Xu, M. Tang, X. Wu, X. Kong, Y. Liu and X. Xu, *Food Funct* **2022**, *13*, 4331-4343.

168. M. Xu, P. Tian, H. Zhu, R. Zou, J. Zhao, H. Zhang, G. Wang and W. Chen, *Nutrients* **2022**, *14*,

169. Z. Xu, C. Wang, X. Dong, T. Hu, L. Wang, W. Zhao, S. Zhu, G. Li, Y. Hu, Q. Gao, J. Wan, Z. Liu and J. Sun, *Biofactors* **2019**, *45*, 187-199.

170. M. Xue, X. Teng, H. Liang, J. Zhao, Y. Jiang, X. Qiu, Z. Zhang, Z. Pei, N. Zhang and Y. Qin, *Journal of Functional Foods* **2021**, *86*,

171. T. Yan, T. Nian, Z. Liao, F. Xiao, B. Wu, K. Bi, B. He and Y. Jia, *Int J Biol Macromol* **2020**, *144*, 427-440.

172. T. Yan, N. Wang, B. Liu, B. Wu, F. Xiao, B. He and Y. Jia, *Phytother Res* **2021**, *35*, 289-296.

173. C. Yang, Y. Fujita, Q. Ren, M. Ma, C. Dong and K. Hashimoto, *Sci Rep* **2017a**, *7*, 45942.

174. C. Yang, Y. Qu, Y. Fujita, Q. Ren, M. Ma, C. Dong and K. Hashimoto, *Transl Psychiatry* **2017**, *7*, 1294.

175. C. Yang, X. Fang, G. Zhan, N. Huang, S. Li, J. Bi, R. Jiang, L. Yang, L. Miao, B. Zhu, A. Luo and K. Hashimoto, *Transl Psychiatry* **2019**, *9*, 57.

176. H.-L. Yang, M.-M. Li, M.-F. Zhou, H.-S. Xu, F. Huan, N. Liu, R. Gao, J. Wang, N. Zhang and L. Jiang, *Inflammation* **2021**, *44*, 2448-2462.

177. J. Yang, G. Zhou, Z. Ou, N. Jia and D. Wang, *Comput Math Methods Med* **2021**, *2021*, 3567447.

178. Q. Yang, L. Luo, T. Sun, L. Yang, L.-F. Cheng, Y. Wang, Q.-Q. Liu, A. Liu, H.-Y. Liu, M.-G. Zhao, S.-X. Wu and B. Feng, *Psychopharmacology (Berl)* **2020**, *237*, 3201-3213.

179. Y. Yang, S. Zhao, X. Yang, W. Li, J. Si and X. Yang, *Neurosci Lett* **2022**, *774*, 136474.

180. J.-B. Yu, Z.-X. Zhao, R. Peng, L.-B. Pan, J. Fu, S.-R. Ma, P. Han, L. Cong, Z.-W. Zhang, L.-X. Sun, J.-D. Jiang and Y. Wang, *Front Pharmacol* **2019**, *10*, 268.

181. M. Yu, H. Jia, C. Zhou, Y. Yang, Y. Zhao, M. Yang and Z. Zou, *J Pharm Biomed Anal* **2017**, *138*, 231-239.

182. M. Yu, H.-M. Jia, T. Zhang, H. Shang, H.-W. Zhang, L.-Y. Ma and Z.-M. Zou, *Metabolites* **2020**, *10*,

183. M. Yu, H.-M. Jia, L.-L. Qin and Z.-M. Zou, *J Affect Disord* **2021**, *297*,

184. S.-W. Yun, J.-K. Kim, K.-E. Lee, Y. J. Oh, H.-J. Choi, M. J. Han and D.-H. Kim, *Nutrients* **2020**, *12*,

185. S. W. Yun, J. K. Kim, M. J. Han and D. H. Kim, *Benef Microbes* **2021**, *12*, 541-551.

186. F. Zhang, H. Chen, R. Zhang, Y. Liu, N. Kong, Y. Guo and M. Xu, *Biochim Biophys Acta Mol Basis Dis* **2020**, *1866*, 165884.

187. J. Zhang, L. Ma, L. Chang, Y. Pu, Y. Qu and K. Hashimoto, *Transl Psychiatry* **2020**, *10*, 186.

188. J. C. Zhang, W. Yao, C. Dong, C. Yang, Q. Ren, M. Ma and K. Hashimoto, *Transl Psychiatry* **2017**, *7*, e1138.

189. K. Zhang, Y. Fujita, L. Chang, Y. Qu, Y. Pu, S. Wang, Y. Shirayama and K. Hashimoto, *Transl Psychiatry* **2019**, *9*, 231.

190. L. Zhang, L. Wang, L. Huang, Y. Zhao, H. Ding, B. Li, L. Wen, W. Xiong, Y. Liu, T. Zhang, L. Zhang, L. Wu, Q. Xu, Y. Fan, G. Wei, Q. Yin, Y. Chen, T. Zhang and Z. Yan, *Pharm Biol* **2021**, *59*, 912-921.

191. M. Zhang, A. Li, Q. Yang, J. Li, L. Wang, X. Liu, Y. Huang and L. Liu, *Front Cell Infect Microbiol* **2021**, *11*, 665159.

192. W. Zhang, W. Qu, H. Wang and H. Yan, *Transl Psychiatry* **2021**, *11*, 131.

193. Y. Zhang, J. Huang, Y. Xiong, X. Zhang, Y. Lin and Z. Liu, *Nutrients* **2021**, *14*,

194. Y. Zhang, R. Huang, M. Cheng, L. Wang, J. Chao, J. Li, P. Zheng, P. Xie, Z. Zhang and H. Yao, *Microbiome* **2019**, *7*, 116.

195. Z. Zhang, N. Li, R. Chen, T. Lee, Y. Gao, Z. Yuan, Y. Nie and T. Sun, *Neurobiol Stress* **2021**, *15*, 100333.

196. Z. Zhang, S. Shao, Y. Zhang, R. Jia, X. Hu, H. Liu, M. Sun, B. Zhang, Q. Li and Y. Wang, *Biomed Pharmacother* **2020**, *132*, 110916.

197. Z. Zhang, C. Yao, M. Li, L.-C. Wang, W. Huang and Q.-J. Chen, *Lett Appl Microbiol* **2022**,

198. B. Zhao, J. Wu, J. Li, Y. Bai, Y. Luo, B. Ji, B. Xia, Z. Liu, X. Tan, J. Lv and X. Liu, *J Agric Food Chem* **2020**, *68*, 3963-3975.

199. F. Zhao, S. Guan, Y. Fu, K. Wang, Z. Liu and T. B. Ng, *Biomed Pharmacother* **2021**, *143*, 112087.

200. W. Zhao, Y. Hu, C. Li, N. Li, S. Zhu, X. Tan, M. Li, Y. Zhang, Z. Xu, Z. Ding, L. Hu, Z. Liu and J. Sun, *Biofactors* **2019**, *46*, 38-54.

201. Z. Zhao, B. Wang, L. Mu, H. Wang, J. Luo, Y. Yang, H. Yang, M. Li, L. Zhou and C. Tao, *Front Cell Infect Microbiol* **2020**, *10*, 258.

202. P. Zheng, B. Zeng, C. Zhou, M. Liu, Z. Fang, X. Xu, L. Zeng, J. Chen, S. Fan, X. Du, X. Zhang, D. Yang, Y. Yang, H. Meng, W. Li, N. D. Melgiri, J. Licinio, H. Wei and P. Xie, *Mol Psychiatry* **2016**, *21*, 786-796.

203. P. Zheng, J. Wu, H. Zhang, S. W. Perry, B. Yin, X. Tan, T. Chai, W. Liang, Y. Huang, Y. Li, J. Duan, M.-L. Wong, J. Licinio and P. Xie, *Mol Psychiatry* **2020**, *26*, 2380-2392.

204. H. Zhou, S. Zhang, X. Zhang, H. Zhou, T. Wen and J. Wang, *Biochem Biophys Res Commun* **2022**, *593*, 137-143.

205. H.-Z. Zhu, Y.-D. Liang, Q.-Y. Ma, W.-Z. Hao, X.-J. Li, M.-S. Wu, L.-J. Deng, Y.-M. Li and J.-X. Chen, *Biomed Pharmacother* **2019**, *112*, 108621.

206. J.-P. Zhu, H.-Y. Wu, Y. Zi, X.-B. Xia, M.-Z. Xie and Z.-Y. Yuan, *Evid Based Complement Alternat Med* **2021**, *2021*, 5554363.

Table S3. Microbiota β-diversity in patients with depression.

**Study**	**Metric**	**Analysis**	**Significance**
Bai S et al. 2021	_	PCoA	Sig. difference
Bai S et al. 2022	_	OPLS-DA	Sig. difference
Caso JR et al. 2021	Bray–Curtis<br>Jaccard	PCoA, PERMANOVA	No sig. difference<br>No sig. difference
Chahwan B et al. 2019	Weighted Unifrac	PCoA, PERMANOVA	No sig. difference
Chen JJ et al. 2018	UniFrac	PCoA, PLS-DA	Sig. difference
Chen JJ et al. 2020	_	OPLS-DA	Sig. difference
Chen T et al. 2021	Unweighted UniFrac	PCoA, ANOSIM	Sig. difference
Chen YH et al. 2021	Weighted Unifrac<br>Unweighted UniFrac	PCoA	Sig. difference<br>Sig. difference
Chung YE et al. 2019	Weighted Unifrac<br>Unweighted UniFrac	PERMANOVA	Sig. difference<br>Sig. difference
Ciocan D et al. 2021	Weighted Unifrac<br>Unweighted UniFrac	ANOSIM	No sig. difference<br>Sig. difference
Dong Z et al. 2021	Bray–Curtis	PCA	No sig. difference
Huang Y et al. 2018	Weighted Unifrac<br>Unweighted UniFrac	PCoA	Sig. difference<br>No sig. difference
Huang Y et al. 2021	Bray-Curtis	PLS-DA, PCA, PCoA, NMDS	Sig. difference
Jiang H et al. 2015	Unweighted UniFrac	PCoA	No sig. difference
Jiang HY et al. 2020	Bray-Curtis<br>Weighted Unifrac<br>Unweighted UniFrac	PCoA	Sig. difference<br>No sig. difference<br>No sig. difference
Kelly JR et al. 2016	Bray-Curtis<br>Weighted Unifrac<br>Unweighted UniFrac	PCoA, Adonis PERMANOVA	Sig. difference<br>Sig. difference<br>Sig. difference
Kleiman SC et al. 2015	Weighted Unifrac<br>Unweighted UniFrac	_	No sig. difference<br>Sig. difference
Kurokawa S et al. 2018	Weighted UniFrac<br>Unweighted Unifrac	PCoA	No sig. difference<br>Sig. difference
Lai WT et al. 2021	Bray-Curtis	PCoA, PERMANOVA	Sig. difference
Lin P et al. 2017	Weighted Unifrac	PCoA	Sig. difference
Ling Y et al. 2020	Bray-Curtis	PCoA, ANOSIM	No sig. difference
Liśkiewicz P et al. 2021	Bray-Curtis<br>Unweighted UniFrac	PCoA, PERMANOVA	_<br>No sig. difference
Liu P et al. 2021	Jaccard	PCoA	Sig. difference
Liu RT et al. 2020	Bray-Curtis<br>Weighted Unifrac<br>Unweighted UniFrac	PCoA, Adonis	Sig. difference<br>Sig. difference<br>Sig. difference
Liu T et al. 2020	Bray-Curtis	PCoA, ANOSIM	No sig. difference
Liu Y et al. 2016	_	PCA	Sig. difference
Madan A et al. 2020	Jaccard similarity index	_	Sig. difference
Mason BL et al. 2020	Weighted UniFrac	PERMANOVA	No sig. difference
Minichino A et al. 2021	Weighted Unifrac<br>Unweighted UniFrac	PCoA	Sig. difference<br>Sig. difference
Naseribafrouei A et al. 2014	_	PLS-DA	Sig. difference
Pérez-Santiago J et al. 2021	Weighted Unifrac<br>Unweighted UniFrac	PCoA, PERMANOVA	Sig. difference<br>Sig. difference
Qin Q et al. 2021	_	PCA, NMDS	No sig. difference

Rhee SJ et al. 2020	Bray-Curtis<br>Weighted Unifrac<br>Unweighted UniFrac	PCoA, PERMANOVA	Sig. difference<br>No sig. difference<br>Sig. difference
Rhee SJ et al. 2021	Bray-Curtis<br>Weighted Unifrac<br>Unweighted UniFrac	PERMANOVA	No sig. difference<br>Sig. difference<br>No sig. difference
Shen Y et al. 2021	Binary jaccard algorithm	PCoA	Sig. difference
Simpson CA et al. 2020	Weighted Unifrac<br>Unweighted UniFrac	PCoA, PERMANOVA	No sig. difference<br>No sig. difference
Stevens BR et al. 2020	Bray-Curtis (unfiltered data)<br>Bray-Curtis (filtered data)	PERMANOVA	No sig. difference<br>Sig. difference
Stevens BR et al. 2021	Bray-Curtis	PCoA	Sig. difference
Taylor AM et al. 2020	Weighted UniFrac	_	_
Taylor BC et al. 2020	Unweighted Unifrac	PERMANOVA	Sig. difference
Wingfield B et al. 2021	Bray-Curtis	_	Sig. difference
Yang J et al. 2020	Bray-Curtis	PCoA, PERMANOVA	Sig. difference
Yang Y et al. 2021	Bray-Curtis<br>Weighted Unifrac<br>Unweighted UniFrac	PCoA, NMDS, ADONIS	Sig. difference<br>Sig. difference<br>Sig. difference
Ye X et al. 2021	Unweighted UniFrac	PCoA	Sig. difference
Zhang Q et al. 2021	Bray-Curtis<br>Jaccard<br>Weighted Unifrac<br>Unweighted UniFrac	PCoA, ANOSIM	Sig. difference<br>Sig. difference<br>Sig. difference<br>Sig. difference
Zhao H et al. 2021	_	PCA	Sig. difference
Zheng P et al. 2016	Bray-Curtis<br>Unweighted UniFrac	PCoA	Sig. difference
Zheng P et al. 2020	_	PLS-DA, PERMANOVA	Sig. difference
Zhou Y et al. 2020	Weighted UniFrac	PCoA, Wilcoxon rank-sum test	Sig. difference
Zhu J et al. 2021	Bary-Curtis<br>Jaccard<br>Weighted Unifrac<br>Unweighted Unifrac	PCoA, PERMANOVA	No sig. difference<br>Sig. difference<br>No sig. difference<br>No sig. difference

Table S4. Microbial β-diversity in animal models of depression.

Study	Metric	Analysis	Significance
Abildgaard A et al. 2021	Bray-Curtis<br>Jaccard	PCoA, PERMANOVA	Sig. difference<br>Sig. difference
An Q et al. 2020	Unweighted UniFrac	PCA, ANOSIM	Sig. difference
Arslanova A et al. 2021	Weighted Unifrac<br>Unweighted UniFrac	PCoA	_
Bharwani A et al. 2017	Bray-Curtis	PCoA	Sig. difference
Bridgewater LC et al. 2017	Bray-Curtis	PCoA	Sig. difference
Burokas A et al. 2017	Unweighted UniFrac	PCoA	Sig. difference
Chakraborti A et al. 2021	Weighted Unifrac<br>Unweighted UniFrac<br>Bray-Curtis	PCoA, PERMANOVA	Sig. difference<br>Sig. difference<br>Sig. difference
Chen L et al. 2021	_	PCA	Sig. difference
Chen P et al. 2019	Bray-Curtis<br>Jaccard	PCoA, NMDS	Sig. difference<br>Sig. difference
Chen T et al. 2021	Unweighted UniFrac	PCoA, ANOSIM	Sig. difference
Chen X et al. 2021	Weighted UniFrac	PCA	Sig. difference
Chen X et al. 2022	Weighted Unifrac	PCoA	Sig. difference
Chen Y et al. 2021b	Weighted Unifrac<br>Bray-Curtis	PCoA, PERMANOVA	Sig. difference<br>Sig. difference
Cheng D et al. 2018	Morisita-Horn dissimilarity	PCA	Sig. difference
Cheng R et al. 2021	UniFrac	PCoA	Sig. difference
Chevalier G et al. 2020	Bray-Curtis	PCoA	_
Chi L et al. 2020	_	PLS-DA	Sig. difference
Daugé V et al. 2020	Bray-Curtis	MDS	Sig. difference
Deng Y et al. 2021	_	PCoA	Sig. difference
Ding Y et al. 2021	_	PCA, ANOSIM	Sig. difference
Diviccaro S et al. 2019	Weighted Unifrac<br>Unweighted UniFrac<br>Bray-Curtis	PCoA, PEMANOVA	Sig. difference<br>Sig. difference<br>Not mention
Donoso F et al. 2020	Aitchison	PCA, PERMANOVA	Sig. difference
Du HX et al. 2020	Bray-Curtis	NMDS	Sig. difference
Duan J et al. 2021	Unweighted UniFrac	PCoA, PLS-DA, ANOSIM	Sig. difference
Egerton S et al. 2020	Bray-Curtis	PCoA, Adonis PERMANOVA	Sig. difference
El Aidy S et al. 2017	Weighted UniFrac	PCoA, PEMANOVA	Sig. difference
Fan L et al. 2021	_	PCoA	Sig. difference
Feng Y et al. 2020	_	PLS-DA	Sig. difference
Feng Z et al. 2020	UniFrac	PCA, NMDS	Sig. difference
Forouzan S et al. 2021	Weighted UniFrac	PCoA	Sig. difference
Gao K et al. 2022	Bray–Curtis	PCoA, PERMANOVA	Sig. difference
Gao X et al. 2020	_	PLS-DA	Sig. difference
Gong X et al. 2021	Weighted UniFrac	PCoA	Sig. difference
Gu F et al. 2020	Weighted UniFrac	PCoA	Sig. difference
Gu X et al. 2022	Weighted UniFrac	PLS-DA, ANOSIM	Sig. difference
Guida F et al. 2018	Bray-Curtis<br>Jaccard	PEMANOVA	Sig. difference<br>Sig. difference
Guo Y et al. 2018	_	NMDS	Sig. difference
Guo Y et al. 2019	_	PCA, Adonis	No sig. difference
Han SK et al. 2020a	Jensen-Shannon	PCoA	Sig. difference

Han SK et al. 2020b	Jensen-Shannon	PCoA	Sig. difference
Han SK et al. 2021	Weighted UniFrac	PCoA	Sig. difference
Hao W et al. 2021	_	PCA	Sig. difference
Hao WZ et al. 2021	Weighted Unifrac<br>Unweighted UniFrac<br>Bray-Curtis	PCoA	Sig. difference<br>Sig. difference<br>Sig. difference
Hassan AM et al. 2019	Weighted UniFrac	PCoA, Adonis	Sig. difference
Huang F et al. 2021	UniFrac	PCA	Sig. difference
Huang N et al. 2019	Euclidean<br>Bray-Curtis	PCoA	Sig. difference<br>Sig. difference
Huang YJ et al. 2021	Bray-Curtis	PCoA	Sig. difference
Huang YY et al. 2022	Bray–Curtis	PcoA	Sig. difference
Inserra A et al. 2019	Weighted UniFrac	PERMANOVA	Sig. difference
Ji S et al. 2022	Weighted Unifrac<br>Unweighted UniFrac<br>Bray-Curtis	PCoA, PERMANOVA	No sig. difference<br>Sig. difference<br>Sig. difference
Jiang W et al. 2021	_	PCoA, Adonis	Sig. difference
Jiang Y et al. 2020	Unweighted UniFrac	PCoA, ANOSIM	Sig. difference
Jianguo L et al. 2019	Bray-Curtis	PERMANOVA	Sig. difference
Kamimura Y et al. 2021	Weighted UniFrac	PCoA	Sig. difference
Kemp KM et al. 2021	Aitchison	PCA, Adonis PERMANOVA	Sig. difference
Kim JK et al. 2020	Weighted Unifrac	PcoA	Sig. difference
Kim JK et al. 2021	Generalized UniFrac	PCoA	Sig. difference
Knudsen JK et al. 2021	_	PCA	Sig. difference
Kosuge A et al. 2021	Bray-Curtis<br>Jaccard	PCoA, Adonis PERMANOVA	Sig. difference<br>Sig. difference
Lai WD et al. 2022	_	PCA, PcoA, NMDS	Sig. difference
Leclercq S et al. 2020	Bray-Curtis<br>Unweighted UniFrac	PCoA	Sig. difference<br>Sig. difference
Lee HC et al. 2020	Weighted Unifrac<br>Unweighted UniFrac	PCoA	Sig. difference<br>Sig. difference
Li H et al. 2021	Bray–Curtis	PCoA	Sig. difference
Li N et al. 2018	Weighted Unifrac<br>Unweighted UniFrac	PCoA, ANOSIM	Sig. difference<br>Sig. difference
Li N et al. 2019	Weighted Unifrac<br>Unweighted UniFrac	PCoA	Sig. difference<br>Sig. difference
Li P et al. 2021	Unweighted UniFrac	PCoA	Sig. difference
Li Y et al. 2018	Weighted Unifrac	PCoA	Sig. difference
Lim EY et al. 2021	Weighted Unifrac	PCoA	Sig. difference
Lin S et al. 2021	_	PCA, NMDS	Sig. difference
Liu QF et al. 2020	Unweighted UniFrac	PCoA	Sig. difference
Liu X et al. 2021a	Bray-Curtis	PCoA, PLS-DA	Sig. difference
Liu X et al. 2021b	Bray-Curtis	PCoA, NMDS, Adonis, Permdisp	Sig. difference
Liu Z et al. 2020	Unweighted UniFrac	PCoA	Sig. difference
Luo X et al. 2021	Bray-Curtis	NMDS, ANOSIM	Sig. difference
Lv M et al. 2021	Weighted Unifrac	PCoA, NMDS	Sig. difference
Lv WJ et al. 2019	Weighted Unifrac	PCA, PCoA, PLS-DA	Sig. difference
Lv WJ et al. 2020	Unweighted UniFrac	PCA, PCoA, NMDS,<br>PERMANOVA, ANOSIM	Sig. difference
Ma W et al. 2019	Bray-Curtis	PCA	Sig. difference

Matsuda Y et al. 2020	Bray-Curtis	PcoA	Sig. difference
McGaughey KD et al. 2019	Unweighted UniFrac	PCoA, ANOSIM	Sig. difference
Meng C et al. 2022	_	CPCoA, PcoA	Sig. difference
Moya-Pérez A et al. 2017	Bray-Curtis	PCoA, Permanova	Sig. difference
O'Mahony SM et al. 2020	Bray-Curtis	PCoA, Adonis	Sig. difference
Partrick KA et al. 2021	Unweighted UniFrac	PCoA, PERMANOVA	Sig. difference
Patterson E et al. 2019	Bray-Curtis	PCoA	Sig. difference
Pearson-Leary J et al. 2020	Bray-Curtis<br>Weighted UniFrac	PCoA	Sig. difference<br>Sig. difference
Pu Y et al. 2021	_	PCA, ANOSIM	No sig. difference
Pusceddu MM et al. 2015	_	RDA	Sig. difference
Qiao Y et al. 2020	Bray-Curtis<br>Weighted Unifrac<br>Unweighted UniFrac	PCoA	Sig. difference<br>Sig. difference<br>Sig. difference
Qu W et al. 2019	Bray-Curtis	PCoA	Sig. difference
Qu Y et al. 2017	Bray-Curtis	PCoA	Sig. difference
Qu Y et al. 2020	Weighted Unifrac	PCoA	Sig. difference
Rao J et al. 2021	Bray-Curtis<br>Jaccard	PCoA	Sig. difference
Robertson RC et al. 2017	Unweighted UniFrac	PCoA	Sig. difference
Schmidtner AK et al. 2019	Bray-Curtis	PCoA	Sig. difference
Shan B et al. 2021	Bray-Curtis	PCoA	Sig. difference
Shao S et al. 2021	_	PCoA	No sig. difference
Sheng L et al. 2021	Unweighted UniFrac	PCoA	Sig. difference
Siopi E et al. 2020	Bray-Curtis	PCoA	Sig. difference
Song J et al. 2019a	_	PLS-DA	Sig. difference
Song J et al. 2019b	_	PLS-DA	Sig. difference
Song X et al. 2021	Bray-Curtis<br>Unweighted UniFrac	PLS-DA, ANOSIM	Sig. difference<br>Sig. difference
Sovijit WN et al. 2019	Bray-Curtis	PCoA, PERMANOVA	Sig. difference
Sun L et al. 2019a	Bray-Curtis	PCoA	Sig. difference
Sun L et al. 2019b	Unweighted UniFrac	PCoA	Sig. difference
Sun Y et al. 2019	Unweighted UniFrac	PCA, PCoA	Sig. difference
Sun Y et al. 2020	Weighted Unifrac	PCoA	Sig. difference
Suzuki K et al. 2021	Unweighted UniFrac	PCoA, PERMANOVA	Sig. difference
Szyszkowicz JK et al. 2017	_	PCA	Sig. difference
Teng T et al. 2021	Bray-Curtis	PCoA, PLS-DA, sparse PLS-DA	Sig. difference
Tian P et al. 2019a	_	PCoA	Sig. difference
Tian P et al. 2019b	_	PCA	Sig. difference
Tian P et al. 2020	Unweighted UniFrac	PCA, PCoA, PERMANOVA	Sig. difference
Tian P et al. 2021	Aitchison	PCA, PERMANOVA	Sig. difference
Tian XY et al. 2021	_	PCA, PCoA, ANOSIM, Adonis	Sig. difference
Tillmann S et al. 2019	_	PCA, PLS-DA	Sig. difference
Tung TH et al. 2019	Bray-Curtis	NMDS	Sig. difference
Wang L et al. 2020	Unweighted UniFrac	PCoA	Sig. difference
Wang L et al. 2021	_	PCoA	Sig. difference
Wang Q et al. 2019	Unweighted UniFrac	PCoA, PERMANOVA	Sig. difference
Wang R et al. 2021	Bray-Curtis	PCoA	Sig. difference

Wang S et al. 2020a	Unweighted UniFrac	PCoA	Sig. difference
Wang S et al. 2020b	Unweighted UniFrac	PCoA	Sig. difference
Wang S et al. 2021	_	PCA, ANOSIM	Sig. difference
Wang Y et al. 2021	Weighted Unifrac<br>Jaccard	PCoA	Sig. difference<br>Sig. difference
Warda AK et al. 2019	Bray-Curtis	PCoA, PERMANOVA	Sig. difference
Wei LN et al. 2019	Bray-Curtis	PCoA, NMDS	Sig. difference
Westfall S et al. 2021	Weighted Unifrac	PCoA, PERMANOVA	Sig. difference
Wong ML et al. 2016	Bray-Curtis	PERMANOVA	Sig. difference
Wu F et al. 2020	Unweighted UniFrac	PcoA	Sig. difference
Wu J et al. 2021	Unweighted UniFrac	PCoA	Sig. difference
Wu M et al. 2020	_	PCoA	Sig. difference
Xia J et al. 2021	Weighted Unifrac<br>Unweighted UniFrac	PCoA, OPLS-DA	Sig. difference<br>Sig. difference
Xiao Q et al. 2020	Unweighted UniFrac	PCoA, NMDS	Sig. difference
Xie R et al. 2020a	Weighted Unifrac<br>Unweighted UniFrac	PCoA	Sig. difference<br>Sig. difference
Xie R et al. 2020b	_	PCA	Sig. difference
Xie Y et al. 2021	Bray-Curtis	PCoA	Sig. difference
Xu M et al. 2022	_	PCoA	Sig. difference
Xu Z et al. 2019	Bray-Curtis<br>Unweighted UniFrac	PCoA	Sig. difference<br>Sig. difference
Xue M et al. 2021	Unweighted UniFrac	PCoA, Adonis	Sig. difference
Yang C et al. 2017b	Bray–Curtis<br>Euclidean	PCoA	Not mention<br>Not mention
Yang C et al. 2019	_	PCoA	Sig. difference
Yang HL et al. 2021	Unweighted UniFrac	PCoA	No sig. difference
Yang Q et al. 2020	Bray-Curtis<br>Weighted Unifrac<br>Unweighted UniFrac	PCoA	Not mention
Yu M et al. 2017	Bray-Curtis	PCoA	Sig. difference
Yu M et al. 2020	_	PCoA	Sig. difference
Yu M et al. 2021	Bray-Curtis	PCoA	Sig. difference
Yun SW et al. 2020	Jansen–Shannon	PCoA	Sig. difference
Yun SW et al. 2021	Bray-Curtis	PCoA	Sig. difference
Zhang F et al. 2020	_	PCoA	Sig. difference
Zhang J et al. 2020	Bray-Curtis	PCoA	Sig. difference
Zhang K et al. 2019	_	PCoA	Sig. difference
Zhang L et al. 2021	Bray-Curtis	PCoA, NMDS	Sig. difference
Zhang M et al. 2021	Bray-Curtis	PCoA, PERMANOVA, ANOSIM	Sig. difference
Zhang W et al. 2021	Weighted Unifrac<br>Unweighted UniFrac	PCoA	Sig. difference<br>Sig. difference
Zhang Y et al. 2019	Unweighted UniFrac	PCoA	Sig. difference
Zhang Y et al. 2021	Bray-Curtis	PLS-DA	Sig. difference
Zhang Z et al. 2020	_	PLS-DA	No sig. difference
Zhang Z et al. 2021	Unweighted UniFrac	PCoA, NMDS, ANOSIM	Sig. difference
Zhang Z et al. 2022	Unweighted UniFrac	PCoA, PERMANOVA, ANOSIM	No sig. difference
Zhao B et al. 2020	Weighted Unifrac<br>Unweighted UniFrac	PCoA	Sig. difference<br>Sig. difference

Zhao F et al. 2021	Bray-Curtis	PCoA	Not mention
Zhao Z et al. 2020	Weighted Unifrac	PCA, PCoA, MDS	Sig. difference
Zheng P et al. 2016	Weighted Unifrac<br>Unweighted UniFrac	PCoA	Sig. difference<br>Sig. difference
Zheng P et al. 2020	_	PLS-DA	Sig. difference
Zhou H et al. 2022	_	PCoA	Sig. difference
Zhu HZ et al. 2019	Weighted Unifrac<br>Unweighted UniFrac	PCA, PCoA, NMDS	Sig. difference<br>Sig. difference

Table S5. Commensal microbiota alterations at Phylum, Class, Order, and Family levels in patients with depression.

Microorganisms				Commensal microbiota			Gut microbiota														
							Total			American			Chinese			MDD			Depression		
				Total no. of frequency	No. of increased	No. of decreased	Total no. of frequency	No. of increased	No. of decreased	Total no. of frequency	No. of increased	No. of decreased	Total no. of frequency	No. of increased	No. of decreased	Total no. of frequency	No. of increased	No. of decreased	Total no. of frequency	No. of increased	No. of decreased
			**Phylum**																		
			Actinobacteria	11	8	3	11	8	3				10	7	3	9	7	2	2	1	1
			Bacteroidetes	13	7	6	13	7	6				12	6	6	13	7	6			
			Firmicutes	13	5	8	12	4	8				10	4	6	10	4	6	2	0	2
			Fusobacteria	3	2	1	2	2	0				2	2	0	2	2	0			
			Proteobacteria	9	7	2	8	6	2				8	6	2	6	4	2	2	2	0
			Saccharibacteria	2	1	1															
			Verrucomicrobia	2	2	0	2	2	0				2	2	0						
		**Phylum**	**Class**																		
		Bacteroidetes	Bacteroidia	4	2	2	4	2	2				3	1	2	4	2	2			
		Firmicutes	Bacilli	2	2	0	2	2	0												
			Clostridia	8	0	8	8	0	8	2	0	2	5	0	5	5	0	5	3	0	3
		Proteobacteria	Alphaproteobacteria	2	1	1	2	1	1												
			Gammaproteobacteria	3	3	0	3	3	0				2	2	0				2	2	0
	**Phylum**	**Class**	**Order**																		
	Actinobacteria	Actinobacteria	Actinomycetales	2	2	0	2	2	0												
			Bifidobacteriales	2	2	0	2	2	0				2	2	0	2	2	0			
		Coriobacteriia	Coriobacteriales	2	2	0	2	2	0				2	2	0	2	2	0			
	Bacteroidetes	Bacteroidia	Bacteroidales	3	1	2	3	1	2				2	0	2	3	1	2			
	Firmicutes	Bacilli	Lactobacillales	3	3	0	3	3	0				2	2	0				2	2	0
		Clostridia	Clostridiales	8	1	7	8	1	7	3	0	3	3	0	3	5	1	4	3	0	3
		Negativicutes	Acidaminococcales	2	1	1	2	1	1				2	1	1	2	1	1			
	Proteobacteria	Alphaproteobacteria	Rhodospirillales	2	0	2	2	0	2							2	0	2			
		Gammaproteobacteria	Aeromonadales	2	0	2	2	0	2				2	0	2				2	0	2
			Alteromonadales	2	0	2	2	0	2				2	0	2	2	0	2			
			Enterobacteriales	2	2	0	2	2	0				2	2	0				2	2	0
			Pasteurellales	2	1	1	2	1	1				2	1	1	2	1	1			
**Phylum**	**Class**	**Order**	**Family**																		

Actinobacteria	Actinobacteria	Actinomycetales	Actinomycetaceae	7	6	1	5	5	0				4	4	0	4	4	0			
		Bifidobacteriales	Bifidobacteriaceae	8	6	2	8	6	2				7	6	1	7	6	1			
		Corynebacteriales	Corynebacteriaceae	2	1	1	2	1	1				2	1	1	2	1	1			
		Micrococcales	Micrococcaceae	3	1	2	2	1	1				2	1	1	2	1	1			
	Coriobacteriia	Coriobacteriales	Coriobacteriaceae	9	5	4	9	5	4	2	1	1	6	4	2	7	4	3	2	1	1
Bacteroidetes	Bacteroidia	Bacteroidales	Bacteroidaceae	10	4	6	9	4	5				9	4	5	9	4	5			
			Barnesiellaceae	3	1	2	3	1	2				2	1	1	3	1	2			
			Porphyromonadaceae	5	5	0	5	5	0				5	5	0	5	5	0			
			Prevotellaceae	9	0	9	9	0	9				8	0	8	8	0	8			
			Rikenellaceae	10	6	4	10	6	4	3	0	3	7	6	1	7	5	2	3	1	2
			Tannerellaceae	3	3	0	3	3	0				3	3	0	3	3	0			
		Marinilabiliales	Marinilabiliaceae	2	1	1	2	1	1							2	1	1			
	Chitinophagia	Chitinophagales	Chitinophagaceae	2	0	2	2	0	2				2	0	2	2	0	2			
	Flavobacteriia	Flavobacteriales	Flavobacteriaceae	3	1	2	2	1	1							2	1	1			
			Fusobacteriaceae	3	2	1	2	2	0				2	2	0	2	2	0			
Firmicutes	Bacilli	Lactobacillales	Carnobacteriaceae	2	1	1	2	1	1				2	1	1						
			Enterococcaceae	5	3	2	4	3	1				3	2	1	4	3	1			
			Erysipelotrichaceae	4	1	3	4	1	3				3	1	2	3	1	2			
			Lactobacillaceae	4	1	3	4	1	3				3	1	2	3	1	2			
			Leuconostocaceae	2	2	0	2	2	0				2	2	0						
			Streptococcaceae	4	3	1	4	3	1				3	2	1	3	2	1			
	Clostridia	Clostridiales	Clostridiaceae	9	3	6	9	3	6				9	3	6	8	2	6			
			Eubacteriaceae	5	4	1	5	4	1				4	4	0	4	3	1			
			Lachnospiraceae	20	8	12	19	7	12	3	0	3	14	5	9	13	6	7	6	1	5
			Oscillospiraceae	4	3	1	4	3	1				4	3	1	4	3	1			
			Peptostreptococcaceae	6	1	5	6	1	5				5	1	4	5	1	4			
			Ruminococcaceae	15	6	9	15	6	9	4	1	3	10	5	5	11	4	7	4	2	2
	Erysipelotrichia	Erysipelotrichales	Turicibacteraceae	2	0	2	2	0	2												
	Negativicutes	Acidaminococcales	Acidaminococcaceae	8	5	3	8	5	3				7	4	3	6	3	3	2	2	0
		Veillonellales	Veillonellaceae	6	4	2	5	3	2				5	3	2	5	3	2			
Patescibacteria	Saccharimonadia	Saccharimonadales	Saccharimonadaceae	2	2	0	2	2	0										2	2	0
Proteobacteria	Betaproteobacteria	Burkholderiales	Burkholderiaceae	3	2	1	3	2	1				3	2	1	3	2	1			
			Oxalobacteraceae	2	0	2															
			Sutterellaceae	2	0	2	2	0	2				2	0	2	2	0	2			
	Epsilonproteobacteria	Campylobacterales	Campylobacteraceae	2	2	0	2	2	0				2	2	0						

	Gammaproteobacteria	Enterobacterales	Enterobacteriaceae	13	10	3	12	9	3	2	2	0	9	6	3	8	5	3	4	4	0
		Pasteurellales	Pasteurellaceae	3	1	2	3	1	2				3	1	2	3	1	2			

Table S6. Commensal microbiota alterations at Genus level in patients with depression.

Microorganisms					Commensal microbiota			Gut microbiota															
								Total			American			Chinese			MDD			Depression		
					Total no. of frequency	No. of increased	No. of decreased	Total no. of frequency	No. of increased	No. of decreased	Total no. of frequency	No. of increased	No. of decreased	Total no. of frequency	No. of increased	No. of decreased	Total no. of frequency	No. of increased	No. of decreased	Total no. of frequency	No. of increased	No. of decreased
**Phylum**	**Class**	**Order**	**Family**	**Genus**																		
Actinobacteria	Actinobacteria	Actinomycetales	Actinomycetaceae	Actinomyces	6	5	1	4	4	0				4	4	0	4	4	0			
		Bifidobacteriales	Bifidobacteriaceae	Bifidobacterium	11	4	7	11	4	7				9	4	5	8	4	4	3	0	3
	Coriobacteriia	Coriobacteriales	Coriobacteriaceae	Collinsella	4	3	1	4	3	1				3	2	1	2	2	0	2	1	1
			Atopobiaceae	Atopobium	2	2	0	2	2	0				2	2	0	2	2	0			
				Olsenella	4	3	1	4	3	1				4	3	1	3	3	0			
		Eggerthellales	Eggerthellaceae	Adlercreutzia	2	2	0	2	2	0				2	2	0	2	2	0			
				Eggerthella	7	7	0	7	7	0				6	6	0	7	7	0			
				Granulicatella	2	2	0	2	2	0				2	2	0				2	2	0
Bacteroidetes	Bacteroidia	Bacteroidales	Bacteroidaceae	Bacteroides	18	8	10	17	8	9	2	1	1	11	5	6	12	7	5	5	1	4
				Barnesiella	6	1	5	6	1	5				3	1	2	2	1	1	4	0	4
			Odoribacteraceae	Butyricimonas	2	1	1	2	1	1				2	1	1						
				Odoribacter	3	1	2	3	1	2				3	1	2	2	1	1			
			Prevotellaceae	Alloprevotella	2	1	1	2	1	1				2	1	1						
				Paraprevotella	6	6	0	6	6	0				3	3	0	3	3	0	3	3	0
				Prevotella	10	5	5	9	4	5				7	3	4	7	3	4	3	2	1
				Prevotella-2	3	1	2	2	0	2				2	0	2	2	0	2			
				Prevotella-9	3	2	1	2	1	1				2	1	1	3	2	1			
				Alistipes	12	7	5	11	6	5				8	4	4	7	5	2	4	1	3
			Tannerellaceae	Parabacteroides	9	8	1	9	8	1				8	7	1	7	7	0	2	1	1
Firmicutes	Bacilli	Bacillales	Bacillales_incertae_sedis	Gemella	2	2	0															
		Lactobacillales	Enterococcaceae	Enterococcus	5	4	1	4	4	0				3	3	0	3	3	0			
			Lactobacillaceae	Lactobacillus	9	5	4	9	5	4				5	4	1	6	4	2	3	1	2
				Weissella	2	0	2	2	0	2				2	0	2						
			Streptococcaceae	Streptococcus	11	9	2	9	7	2				8	7	1	7	5	2	2	2	0

	Clostridia	Clostridiales	Christensenellaceae	Christensenellaceae R-7 group	5	0	5	5	0	5	2	0	2	3	0	3	4	0	4			
			Clostridiaceae	Butyricicoccus	3	0	3	3	0	3				2	0	2	2	0	2			
			Clostridiaceae	Clostridium_innocuum_group	2	1	1	2	1	1				2	1	1	2	1	1			
			Clostridiaceae	Hungatella	2	2	0	2	2	0				2	2	0	2	2	0			
			Eubacteriaceae	Eubacterium	6	3	3	6	3	3				5	3	2	4	3	1	2	0	2
				Eubacterium_ventriosum_group	3	1	2	3	1	2				2	1	1	3	1	2			
			Eubacteriales Family XIII. Incertae Sedis	Anaerovorax	3	0	3	3	0	3				3	0	3	2	0	2			
			Lachnospiraceae	Agathobacter	2	0	2	2	0	2												
				Anaerostipes	8	4	4	8	4	4				6	4	2	6	4	2	2	0	2
				Blautia	12	7	5	12	7	5	2	1	1	9	5	4	8	6	2	4	1	3
				CAG-56	3	0	3	3	0	3				2	0	2	2	0	2			
				Clostridium_XIVa	2	1	1	2	1	1												
				Coprococcus	9	0	9	9	0	9				6	0	6	5	0	5	4	0	4
				Dorea	5	2	3	5	2	3				3	1	2	3	1	2	2	1	1
				Eisenbergiella	2	2	0	2	2	0				2	2	0	2	2	0			
				Fusicatenibacter	6	0	6	6	0	6				4	0	4	3	0	3	3	0	3
				Lachnoclostridium	4	3	1	4	3	1				3	2	1	3	2	1			
				Lachnospira	6	1	5	6	1	5	3	0	3	3	1	2	3	1	2	3	0	3
				Lachnospiracea incertae sedis	3	1	2	3	1	2				3	1	2	2	1	1			
				Lachnospiraceae ND3007 group	4	0	4	4	0	4				2	0	2	2	0	2			
				Lachnospiraceae_NK4A136_group	3	1	2	3	1	2				2	1	1	2	1	1			
				Lachnospiraceae_UCG-001	2	0	2	2	0	2												
				Roseburia	13	5	8	13	5	8	3	1	2	9	3	6	8	3	5	5	2	3
				Shuttleworthia	2	2	0	2	2	0				2	2	0						
				Tyzzerella	3	1	2	3	1	2				2	1	1	3	1	2			
			Oscillospiraceae	Oscillibacter	9	4	5	9	4	5				6	3	3	5	3	2	4	1	3
			Peptostreptococcaceae	Clostridium_XI	4	2	2	4	2	2				4	2	2	3	2	1			
				Romboutsia	3	0	3	3	0	3				2	0	2				2	0	2
			Ruminococcaceae	Anaerofilum	2	1	1	2	1	1												

				Anaerotruncus	2	2	0	2	2	0				2	2	0	3	3	0			
				Clostridium_IV	2	1	1	2	1	1												
				Faecalibacterium	19	3	16	18	2	16	3	0	3	12	2	10	10	2	8	8	0	8
				Flavonifractor	5	5	0	5	5	0	2	2	0	3	3	0	4	4	0			
				Oscillospira	2	2	0	2	2	0							2	2	0			
				Ruminococcaceae_UCG-002	2	1	1															
				Ruminococcaceae_UCG-014	2	2	0	2	2	0												
				Ruminococcus	9	1	8	9	1	8	3	0	3	4	1	3	3	1	2	6	0	6
				Ruminococcus-1	3	1	2	3	1	2	2	1	1				2	0	2			
				Ruminococcus-2	2	0	2	2	0	2				2	0	2	2	0	2			
				Subdoligranulum	3	0	3	3	0	3				2	0	2	2	0	2			
	Erysipelotrichia	Erysipelotrichales	Erysipelotrichaceae	Clostridium_XVIII	2	1	1	2	1	1												
				Faecalitalea	3	2	1	3	2	1				3	2	1	3	2	1			
				Holdemanella	2	0	2	2	0	2										2	0	2
				Holdemania	5	5	0	5	5	0				3	3	0	4	4	0			
			Turicibacteraceae	Turicibacter	4	2	2	4	2	2				2	1	1	3	2	1			
	Negativicutes	Acidaminococcales	Acidaminococcaceae	Acidaminococcus	3	3	0	3	3	0				2	2	0	2	2	0			
				Phascolarctobacterium	7	2	5	7	2	5				6	1	5	6	1	5			
		Selenomonadales	Selenomonadaceae	Megamonas	6	1	5	6	1	5				6	1	5	5	1	4			
				Mitsuokella	3	1	2	2	0	2				2	0	2				2	0	2
		Veillonellales	Veillonellaceae	Dialister	12	5	7	11	4	7	2	2	0	5	2	3	5	2	3	6	2	4
				Megasphaera	4	1	3	4	1	3				4	1	3	4	1	3			
				Veillonella	8	7	1	7	6	1				6	5	1	2	2	0	5	4	1
	Tissierellia	Tissierellales	Peptoniphilaceae	Parvimonas	4	3	1	3	2	1				3	2	1	2	2	0			
Fusobacteria	Fusobacteriia	Fusobacterales	Fusobacteriaceae	Fusobacterium	5	2	3	3	1	2				3	1	2	2	1	1			
Proteobacteria	Alphaproteobacteria	Rhizobiales	Hyphomicrobiaceae	Gemmiger	3	0	3	3	0	3				2	0	2				3	0	3
	Betaproteobacteria	Burkholderiales	Sutterellaceae	Parasutterella	2	1	1	2	1	1				2	1	1	2	1	1			
			Sutterellaceae	Sutterella	8	1	7	8	1	7				7	0	7	6	1	5	2	0	2
	Deltaproteobacteria	Desulfovibrionales	Desulfovibrionaceae	Desulfovibrio	5	4	1	5	4	1				2	2	0	4	3	1			
	Epsilonproteobacteria	Campylobacterales	Campylobacteraceae	Campylobacter	2	1	1	2	1	1				2	1	1						
	Gammaproteobacteria	Enterobacterales	Enterobacteriaceae	Enterobacter	2	1	1	2	1	1				2	1	1	2	1	1			
				Escherichia	2	2	0	2	2	0				2	2	0	2	2	0			
				Escherichia-Shigella	7	5	2	6	4	2				5	3	2	3	2	1	3	2	1
				Klebsiella	2	1	1	2	1	1				2	1	1	2	1	1			

		Pasteurellales	Pasteurellaceae	Haemophilus	4	1	3	4	1	3				4	1	3	3	0	3			
		Pseudomonadales	Pseudomonadaceae	Pseudomonas	2	1	1															
Streptophyta	Magnoliopsida	Fabales	Fabaceae	Rothia	3	2	1	3	2	1				3	2	1	3	2	1			
Verrucomicrobia	Verrucomicrobiae	Verrucomicrobiales	Akkermansiaceae	Akkermansia	3	2	1	3	2	1				2	2	0				2	1	1

Table S7. Commensal microbiota alterations at Species level in patients with depression.

Microorganisms						Commensal microbiota			Gut microbiota															
									Total			American			Chines			MDD			Depression		
						Total no. of frequency	No. of increased	No. of decreased	Total no. of frequency	No. of increased	No. of decreased	Total no. of frequency	No. of increased	No. of decreased	Total no. of frequency	No. of increased	No. of decreased	Total no. of frequency	No. of increased	No. of decreased	Total no. of frequency	No. of increased	No. of decreased
**Phylum**	**Class**	**Order**	**Family**	**Genus**	**Species**																		
Actinobacteria	Actinomycetia	Bifidobacteriales	Bifidobacteriaceae	Bifidobacterium	Bifidobacterium_adolescentis	3	3	0	3	3	0				2	2	0	2	2	0			
					Bifidobacterium_bifidum	2	2	0	2	2	0				2	2	0	2	2	0			
					Bifidobacterium_breve	2	2	0	2	2	0				2	2	0	2	2	0			
					Bifidobacterium_dentium	2	2	0	2	2	0				2	2	0	2	2	0			
					Bifidobacterium_longum	6	4	2	6	4	2				4	3	1	4	3	1	2	1	1
		Micrococcales	Micrococcaceae	Rothia	Rothia_mucilaginosa	3	2	1	2	2	0				2	2	0	2	2	0			
	Coriobacteriia	Coriobacteriales	Atopobiaceae	Lancefieldella	Atopobium_parvulum	2	2	0	2	2	0				2	2	0	2	2	0			
				Olsenella	Olsenella_uli	2	2	0	2	2	0				2	2	0	2	2	0			
			Coriobacteriaceae	Coriobacterium	Coriobacterium_glomerans	2	2	0	2	2	0				2	2	0	2	2	0			
		Eggerthellales	Eggerthellaceae	Adlercreutzia	Adlercreutzia_equolifaciens	3	2	1	3	2	1				2	1	1	2	1	1			
				Eggerthella	Eggerthella_lenta	3	3	0	3	3	0				3	3	0	3	3	0			
Bacteroidetes	Bacteroidia	Bacteroidales	Bacteroidaceae	Bacteroides	Bacteroides_caccae	3	2	1	3	2	1	2	1	1				2	2	0			
					Bacteroides_dorei	2	2	0	2	2	0												
					Bacteroides_fragilis	3	3	0	3	3	0				2	2	0	2	2	0			
					Bacteroides_massiliensis	3	2	1	3	2	1				2	1	1	2	1	1			
					Bacteroides_nordii	2	2	0	2	2	0												
					Bacteroides_plebeius	2	1	1	2	1	1				2	1	1						
					Bacteroides_stercoris	3	2	1	3	2	1	2	1	1							2	1	1
					Bacteroides_thetaiotaomicron	4	4	0	4	4	0				3	3	0	2	2	0	2	2	0
					Bacteroides_uniformis	2	2	0	2	2	0												
					Bacteroides_vulgatus	2	1	1	2	1	1												
			Prevotellaceae	Paraprevotella	Paraprevotella_xylaniphila	2	2	0	2	2	0				2	2	0	2	2	0			
				Prevotella	Prevotella_copri	2	2	0	2	2	0										2	2	0
					Prevotella_melaninogenica	2	2	0	2	2	0				2	2	0	2	2	0			
			Rikenellaceae	Alistipes	Alistipes_finegoldii	2	2	0	2	2	0												
					Alistipes_onderdonkii	3	3	0	3	3	0	2	2	0				2	2	0			

					Alistipes_senegalensis	2	2	0	2	2	0	2	2	0							2	2	0
			Tannerellaceae	Parabacteroides	Parabacteroides_distasonis	3	3	0	3	3	0							3	3	0			
					Parabacteroides_merdae	2	2	0	2	2	0	2	2	0							2	2	0
			Enterococcaceae	Enterococcus	Enterococcus_faecium	3	2	1	3	2	1				3	2	1						
			Lactobacillaceae	Lactobacillus	Lactobacillus_crispatus	2	2	0	2	2	0				2	2	0	2	2	0			
	Bacilli	Lactobacillales			Streptococcus_parasanguinis	3	3	0	3	3	0				2	2	0	2	2	0			
			Streptococcaceae	Streptococcus	Streptococcus_pyogenes	3	3	0	3	3	0				3	3	0	3	3	0			
					Streptococcus_salivarius	3	1	2	3	1	2				2	0	2	2	0	2			
					Clostridium_asparagiforme	2	2	0	2	2	0												
					Clostridium_bolteae	2	2	0	2	2	0												
			Clostridiaceae	Clostridium	Clostridium_citroniae	3	3	0	3	3	0							2	2	0			
					Clostridium_hathewayi	2	2	0	2	2	0												
					Clostridium_saccharolyticum	2	2	0	2	2	0				2	2	0	2	2	0			
					Clostridium_symbiosum	3	3	0	3	3	0	3	3	0							2	2	0
			Lachnospiraceae	Coprococcus	Coprococcus_catus	3	2	1	3	2	1	2	1	1							2	1	1
					Coprococcus_eutactus	2	0	2	2	0	2				2	0	2	2	0	2			
			Ruminococcaceae	Clostridium IV	Clostridium_leptum	2	0	2	2	0	2	2	0	2									
			Eubacteriaceae	Eubacterium	Eubacterium rectale	4	1	3	4	1	3				2	1	1	2	1	1	2	0	2
Firmicutes			Heliobacteriaceae	Heliomicrobium	Heliobacterium modesticaldum	2	2	0	2	2	0				2	2	0	2	2	0			
	Clostridia	Chlorobiales		Anaerostipes	Anaerostipes_hadrus	2	0	2	2	0	2				2	0	2						
				Dorea	Dorea_longicatena	2	0	2	2	0	2												
			Lachnospiraceae	Roseburia	Roseburia_inulinivorans	2	1	1	2	1	1												
				unclassified Lachnospiraceae	Eubacterium_eligens	2	0	2	2	0	2												
			Oscillospiraceae	Oscillibacter	Oscillibacter_valericigenes	2	2	0	2	2	0				2	2	0	2	2	0			
				Faecalibacterium	Faecalibacterium_prausnitzii	4	0	4	4	0	4	2	0	2	2	0	2	3	0	3			
				Flavonifractor	Flavonifractor_plautii	4	4	0	4	4	0	3	3	0							3	3	0
			Ruminococcaceae		Ruminococcus_bromii	2	0	2	2	0	2												
				Ruminococcus	Ruminococcus_gnavus	4	4	0	4	4	0	2	2	0							4	4	0
					Ruminococcus_torques	2	1	1	2	1	1												
			unclassified_Clostridiales	unclassified_Clostridiales	Clostridiales_bacterium_1_7_47FAA	2	2	0	2	2	0												
	Erysipelotrichia	Erysipelotrichales	Erysipelotrichaceae	Holdemania	Holdemania_filiformis	2	2	0	2	2	0	2	2	0							2	2	0

	Negativicutes	Acidaminococcales	Acidaminococcaceae	Acidaminococcus	Acidaminococcus_fermentans	2	2	0	2	2	0				2	2	0	2	2	0			
					Acidaminococcus_intestini	2	2	0	2	2	0				2	2	0	2	2	0			
		Veillonellales	Veillonellaceae	Megasphaera	Megasphaera_elsdenii	2	2	0	2	2	0				2	2	0	2	2	0			
Proteobacteria	Deltaproteobacteria	Desulfovibrionales	Desulfovibrionaceae	Bilophila	Bilophila_wadsworthia	2	2	0	2	2	0												
				Desulfovibrio	Desulfovibrio_desulfuricans	2	2	0	2	2	0												
					Desulfovibrio_vulgaris	2	2	0	2	2	0				2	2	0	2	2	0			
	Gammaproteobacteria	Enterobacterales	Enterobacteriaceae	Enterobacter	Enterobacter_cloacae	2	2	0	2	2	0				2	2	0	2	2	0			
					Enterococcus_faecalis	2	1	1	2	1	1				2	1	1	2	1	1			
				Escherichia	Escherichia coli	4	4	0	4	4	0				4	4	0	3	3	0			
				Klebsiella	Klebsiella_pneumoniae	2	1	1	2	1	1							2	1	1			
		Pasteurellales	Pasteurellaceae	Haemophilus	Haemophilus_parainfluenzae	5	0	5	4	0	4				3	0	3	4	0	4			
Verrucomicrobia	Verrucomicrobiae	Verrucomicrobiales	Akkermansiaceae	Akkermansia	Akkermansia_muciniphila	5	3	2	5	3	2	2	1	1	3	2	1	3	2	1	2	1	1

Table S8. Commensal microbiota alterations at Phylum, Class, Order, and Family levels in animal models of depression.

Microorganisms		Commensal microbiota			Total																	
					Total			Mice			Rats			Fecal samples			Cecum contents			Colon contents		
		Total no. of frequency	No. of increased	No. of decreased	Total no. of frequency	No. of increased	No. of decreased	Total no. of frequency	No. of increased	No. of decreased	Total no. of frequency	No. of increased	No. of decreased	Total no. of frequency	No. of increased	No. of decreased	Total no. of frequency	No. of increased	No. of decreased	Total no. of frequency	No. of increased	No. of decreased
	**Phylum**																					
	Actinobacteria	35	14	21	35	14	21	27	9	18	8	5	3	30	11	19	5	3	2			
	Bacteroidetes	73	40	33	73	40	33	49	25	24	22	15	7	51	29	22	17	9	8	5	2	3
	Cyanobacteria	11	3	8	11	3	8	8	3	5	3	0	3	9	2	7	2	1	1			
	Deferribacteres	12	7	5	12	7	5	11	7	4				11	7	4						
	Firmicutes	78	28	50	78	28	50	55	18	37	23	10	13	58	20	38	15	6	9	5	2	3
	Proteobacteria	48	37	11	48	37	11	41	31	10	7	6	1	34	26	8	10	6	4	6	5	1
	Tenericutes	15	5	10	15	5	10	13	4	9	2	1	1	14	5	9						
	TM7	5	2	3	5	2	3	4	2	2				4	1	3						
	Verrucomicrobia	30	14	16	30	14	16	23	11	12	7	3	4	18	7	11	8	5	3	4	2	2
**Phylum**	**Class**																					
Actinobacteria	Actinobacteria	9	7	2	9	7	2	3	2	1	5	4	1	4	2	2	3	3	0			
	Coriobacteriia	4	1	3	4	1	3	3	1	2				4	1	3						
Bacteroidetes	Bacteroidia	15	7	8	15	7	8	12	6	6	3	1	2	11	6	5	3	1	2			
Cyanobacteria	4C0d_2	3	2	1	3	2	1	3	2	1							2	1	1			
Deferribacteres	Deferribacteres	5	4	1	5	4	1	4	3	1				5	4	1						
Firmicutes	Bacilli	15	7	8	15	7	8	9	4	5	6	3	3	8	3	5	5	3	2			
	Clostridia	17	11	6	17	11	6	12	7	5	5	4	1	11	7	4	3	1	2	2	2	0
	Erysipelotrichia	10	3	7	10	3	7	9	3	6				8	2	6						
	Negativicutes	2	1	1	2	1	1							2	1	1						
Proteobacteria	Alphaproteobacteria	6	0	6	6	0	6	5	0	5				4	0	4	2	0	2			
	Betaproteobacteria	7	5	2	7	5	2	2	1	1	5	4	1	3	2	1	3	3	0			
	Deltaproteobacteria	9	7	2	9	7	2	4	4	0	5	3	2	6	6	0						
	Epsilonproteobacteria	7	7	0	7	7	0	6	6	0				4	4	0	2	2	0			
	Gammaproteobacteria	8	8	0	8	8	0	7	7	0				6	6	0						
Spirochaetes	Spirochaetia	3	2	1	3	2	1	3	2	1				3	2	1						
Tenericutes	Mollicutes	7	4	3	7	4	3	6	4	2				6	4	2						

		Verrucomicrobia	Verrucomicrobiae	8	5	3	8	5	3	7	4	3				4	2	2	2	1	1	2	2	0
	**Phylum**	**Class**	**Order**																					
	Actinobacteria	Actinobacteria	Actinomycetales	4	2	2	4	2	2	2	1	1	2	1	1	3	1	2						
			Bifidobacteriales	10	6	4	10	6	4	6	2	4	4	4	0	3	2	1	6	3	3			
			Micrococcales	2	0	2	2	0	2							2	0	2						
		Coriobacteriia	Coriobacteriales	4	2	2	4	2	2	3	2	1				4	2	2						
	Bacteroidetes	Bacteroidia	Bacteroidales	24	14	10	24	14	10	19	11	8	5	3	2	19	12	7	4	2	2			
	Cyanobacteria	4C0d-2	YS2	3	2	1	3	2	1	3	2	1							2	1	1			
		Melainabacteria	Gastranaerophilales	3	0	3	3	0	3				3	0	3	2	0	2						
	Deferribacteres	Deferribacteres	Deferribacterales	6	4	2	6	4	2	5	4	1				5	4	1						
	Firmicutes	Bacilli	Bacillales	13	7	6	13	7	6	8	5	3	4	2	2	9	5	4	2	1	1			
			Lactobacillales	19	8	11	19	8	11	13	4	9	6	4	2	9	4	5	6	3	3			
		Clostridia	Clostridiales	26	14	12	26	14	12	18	9	9	8	5	3	17	8	9	5	2	3	2	2	0
		Erysipelotrichia	Erysipelotrichales	10	3	7	10	3	7	9	3	6				8	2	6						
		Negativicutes	Selenomonadales	2	1	1	2	1	1							2	1	1						
	Proteobacteria	Alphaproteobacteria	Rhizobiales	2	1	1	2	1	1				2	1	1									
			Rickettsiales	3	0	3	3	0	3	3	0	3				2	0	2						
			Sphingomonadales	2	1	1	2	1	1										2	1	1			
		Betaproteobacteria	Burkholderiales	8	5	3	8	5	3	2	2	0	6	3	3	4	1	3	4	4	0			
		Deltaproteobacteria	Desulfovibrionales	11	9	2	11	9	2	6	6	0	5	3	2	6	6	0	3	2	1			
		Epsilonproteobacteria	Campylobacterales	9	8	1	9	8	1	8	7	1				6	5	1	2	2	0			
		Gammaproteobacteria	Enterobacteriales	4	4	0	4	4	0	4	4	0				3	3	0						
			Pseudomonadales	3	2	1	3	2	1				2	2	0	2	1	1						
	Spirochaetes	Spirochaetia	Spirochaetales	3	2	1	3	2	1	3	2	1				3	2	1						
	Tenericutes	Mollicutes	Anaeroplasmatales	5	1	4	5	1	4	4	0	4				3	1	2	2	0	2			
			Mycoplasmatales	3	1	2	3	1	2	3	1	2												
			Mollicutes_RF39	6	3	3	6	3	3	6	3	3				6	3	3						
	Verrucomicrobia	Verrucomicrobiae	Verrucomicrobiales	8	4	4	8	4	4	7	3	4				4	2	2				2	2	0
**Phylum**	**Class**	**Order**	**Family**																					
Actinobacteria	Actinobacteria	Bifidobacteriales	Bifidobacteriaceae	15	8	7	15	8	7	9	3	6	6	5	1	7	4	3	7	3	4			
		Micrococcales	Micrococcaceae	4	1	3	4	1	3	2	1	1	2	0	2	3	0	3						
	Actinomycetia	Corynebacteriales	Corynebacteriaceae	3	2	1	3	2	1				2	1	1									
	Coriobacteriia	Coriobacteriales	Coriobacteriaceae	15	5	10	15	5	10	12	5	7	3	0	3	12	5	8						
			Atopobiaceae	2	1	1	2	1	1															

		Eggerthellales	Eggerthellaceae	6	2	4	6	2	4	5	2	3				5	2	3						
Bacteroidetes	Bacteroidia	Bacteroidales	Bacteroidaceae	30	19	11	30	19	11	21	16	5	8	3	5	21	13	8	6	5	1	3	1	2
			Bacteroidales_RF16_group	3	2	1	3	2	1				2	1	1	2	1	1						
			Bacteroidales_S24-7_group	21	10	11	21	10	11	13	5	8	8	5	3	18	7	11	2	2	0			
			Muribaculaceae	20	5	15	20	5	15	13	2	11	6	2	4	14	3	11				4	2	2
			Odoribacteraceae	6	2	4	6	2	4	6	2	4				5	2	3						
			Paraprevotellaceae	4	2	2	4	2	2	3	1	2				2	1	1						
			Porphyromonadaceae	14	9	5	14	9	5	11	7	4	3	2	1	11	8	3	3	1	2			
			Prevotellaceae	31	13	18	31	13	18	24	10	14	6	3	3	25	12	13	3	1	2	4	1	3
			Rikenellaceae	28	11	17	28	11	17	21	8	13	6	2	4	21	9	12	4	1	3	3	1	2
			Tannerellaceae	6	2	4	6	2	4	4	1	3	2	1	1	3	1	2	2	1	1			
		Marinilabiliales	Marinifilaceae	2	2	0	2	2	0	2	2	0												
	Cytophagia	Cytophagales	Cytophagaceae	2	2	0	2	2	0	2	2	0				2	2	0						
Deferribacteres	Deferribacteres	Deferribacterales	Deferribacteraceae	7	4	3	7	4	3	4	3	1	2	0	2	6	4	2						
Firmicutes	Bacilli	Bacillales	Bacillaceae	9	4	5	9	4	5	5	2	3	3	1	2	7	3	4						
			Planococcaceae	2	2	0	2	2	0															
			Staphylococcaceae	9	5	4	9	5	4	7	4	3	2	1	1	7	3	4						
		Lactobacillales	Aerococcaceae	5	4	1	5	4	1	3	3	0				2	2	0	2	1	1			
			Carnobacteriaceae	5	2	3	5	2	3	3	2	1				2	1	1	2	0	2			
			Enterococcaceae	7	4	3	7	4	3	6	4	2				3	2	1	4	2	2			
			Lactobacillaceae	36	12	24	36	12	24	21	8	13	14	4	10	24	8	16	9	3	6	2	0	2
			Leuconostocaceae	2	0	2	2	0	2	2	0	2				2	0	2						
			Streptococcaceae	9	2	7	9	2	7	6	2	4	2	0	2	5	0	5	3	2	1			
		Turicibacterales	Turicibacteraceae	3	1	2	3	1	2	2	1	1				3	1	2						
	Clostridia	Clostridiales	Caldicoprobacteraceae	2	0	2	2	0	2				2	0	2	2	0	2						
			Christensenellaceae	11	7	4	11	7	4	7	5	2	3	2	1	8	6	2	3	1	2			
			Clostridiales_vadinBB60_group	7	4	3	7	4	3	3	2	1	4	2	2	5	3	2						
			Clostridiaceae	20	15	5	20	15	5	14	11	3	6	4	2	14	12	2	4	3	1	2	0	2
			Defluviitaleaceae	2	0	2	2	0	2							2	0	2						
			Eubacteriaceae	4	1	3	4	1	3	4	1	3				2	0	2	2	1	1			
			Lachnospiraceae	45	19	26	45	19	26	31	14	17	13	5	8	30	12	18	11	4	7	4	3	1
			Mogibacteriaceae	6	2	4	6	2	4	5	1	4				5	2	3						
			Oscillospiraceae	3	2	1	3	2	1													2	2	0
			Peptococcaceae	10	4	6	10	4	6	7	4	3	2	0	2	8	2	6	2	2	0			

			Peptostreptococcaceae	12	7	5	12	7	5	7	6	1	5	1	4	7	3	4	2	2	0	2	1	1
			Ruminococcaceae	45	27	18	45	27	18	31	19	12	13	7	6	34	19	15	7	5	2	3	3	0
	Erysipelotrichia	Erysipelotrichales	Erysipelotrichaceae	19	7	12	19	7	12	16	5	11	2	1	1	13	4	9	3	2	1			
	Negativicutes	Acidaminococcales	Acidaminococcaceae	2	0	2	2	0	2															
	Negativicutes	Veillonellales	Veillonellaceae	3	2	1	3	2	1				2	1	1	3	2	1						
			Anaerovoracaceae	3	2	1	3	2	1							2	1	1						
			Monoglobaceae	2	2	0	2	2	0															
Patescibacteria	Saccharimonadia	Saccharimonadales	Saccharimonadaceae	3	3	0	3	3	0	3	3	0				2	2	0						
Proteobacteria	Alphaproteobacteria	Hyphomicrobiales	Devosiaceae	2	0	2	2	0	2	2	0	2												
		Rhizobiales	Brucellaceae	2	2	0	2	2	0							2	2	0						
		Rhodospirillales	Rhodospirillaceae	2	1	1	2	1	1	2	1	1				2	1	1						
		Sphingomonadales	Sphingomonadaceae	4	3	1	4	3	1	3	3	0				2	2	0	2	1	1			
	Betaproteobacteria	Burkholderiales	Alcaligenaceae	6	2	4	6	2	4	5	2	3				5	2	3						
			Burkholderiaceae	3	0	3	3	0	3															
			Comamonadaceae	3	3	0	3	3	0							2	2	0						
			Oxalobacteraceae	5	4	1	5	4	1	2	1	1	2	2	0				3	3	0			
			Sutterellaceae	6	1	5	6	1	5	5	1	4				4	0	4	2	1	1			
	Deltaproteobacteria	Desulfovibrionales	Desulfovibrionaceae	27	20	7	27	20	7	20	15	5	7	5	2	19	16	3	6	3	3			
	Epsilonproteobacteria	Campylobacterales	Campylobacteraceae	2	0	2	2	0	2															
			Helicobacteraceae	15	12	3	15	12	3	12	9	3	2	2	0	12	9	3						
	Gammaproteobacteria	Enterobacterales	Enterobacteriaceae	14	12	2	14	12	2	13	11	2				11	10	1	2	1	1			
		Pasteurellales	Pasteurellaceae	3	2	1	3	2	1							2	2	0						
		Pseudomonadales	Moraxellaceae	5	3	2	5	3	2	2	1	1	3	2	1	2	1	1	2	2	0			
			Pseudomonadaceae	2	0	2	2	0	2															
Spirochaetes	Spirochaetia	Spirochaetales	Spirochaetaceae	3	2	1	3	2	1	2	1	1				2	1	1						
		Brachyspirales	Brachyspiraceae	2	2	0	2	2	0															
Tenericutes	Mollicutes	Anaeroplasmatales	Anaeroplasmataceae	8	3	5	8	3	5	7	2	5				6	3	3	2	0	2			
		Mycoplasmatales	Mycoplasmataceae	3	1	2	3	1	2	3	1	2				2	1	1						
Verrucomicrobia	Verrucomicrobiae	Verrucomicrobiales	Akkermansiaceae	5	0	5	5	0	5	5	0	5				3	0	3						
			Verrucomicrobiaceae	10	9	1	10	9	1	8	8	0	2	1	1	4	3	1	4	4	0	2	2	0
			AC160630	3	1	2	3	1	2	3	1	2				3	1	2						

Table S9. Commensal microbiota alterations at Genus level in animal models of depression.

Microorganisms					Commensal microbiota			Gut microbiota																		
								Total			Mice			Rats			Fecal samples			Cecum contents			Colon contents			
					Total no. of frequency	No. of increased	No. of decreased	Total no. of frequency	No. of increased	No. of decreased	Total no. of frequency	No. of increased	No. of decreased	Total no. of frequency	No. of increased	No. of decreased	Total no. of frequency	No. of increased	No. of decreased	Total no. of frequency	No. of increased	No. of decreased	Total no. of frequency	No. of increased	No. of decreased	
**Phylum**	**Class**	**Order**	**Family**	**Genus**																						
Actinobacteria	Actinobacteria	Actinomycetales	Micrococcaceae	Rothia	9	4	5	9	4	5				9	4	5	5	3	2	2	0	2				
			Nocardiaceae	Rhodococcus	3	1	2	3	1	2	3	1	2				2	1	1							
		Bifidobacteriales	Bifidobacteriaceae	Bifdobacterium	38	13	25	38	13	25	23	6	17	15	7	8	27	9	18	9	3	6				
		Corynebacteriales	Corynebacteriaceae	Corynebacterium	11	6	5	11	6	5	4	3	1	7	3	4	5	3	2	2	2	0				
		Micrococcales	Brevibacteriaceae	Brevibacterium	3	2	1	3	2	1				2	2	0				2	2	0				
			Dermabacteraceae	Brachybacterium	3	2	1	3	2	1				2	2	0				2	2	0				
			Micrococcaceae	Arthrobacter	2	2	0	2	2	0				2	2	0				2	2	0				
				Yaniella	3	2	1	3	2	1				2	2	0				2	2	0				
	Coriobacteriia	Coriobacteriales	Atopobiaceae	Olsenella	5	3	2	5	3	2	4	2	2				4	3	1							
			Coriobacteriaceae	Coriobacteriaceae_UCG-002	3	2	1	3	2	1	3	2	1				2	1	1							
				Enterorhabdus	11	3	8	11	3	8	9	3	6	2	0	2	8	3	5	3	0	3				
		Eggerthellales	Eggerthellaceae	Adlercreutzia	10	7	3	10	7	3	9	7	1				8	5	3							
Bacteroidetes	Bacteroidia	Bacteroidales	Bacteroidaceae	Bacteroides	59	36	23	59	36	23	42	27	15	17	9	8	41	25	16	11	7	4	6	3	3	
			Barnesiellaceae	Barnesiella	11	4	7	11	4	7	11	4	7				6	3	3	3	1	2	2	0	2	
			Muribaculaceae	Muribaculum	9	2	7	9	2	7	8	2	6				7	1	6							
			Odoribacteraceae	Butyricimonas	13	5	8	13	5	8	10	4	6	3	1	2	10	4	6	2	1	1				
				Odoribacter	18	10	8	18	10	8	17	9	8				10	5	5	7	4	3				
			Porphyromonadaceae	Parabacteroides	39	22	17	39	22	17	27	16	11	12	6	8	27	16	11	8	3	5	4	3	1	
			Prevotellaceae	Alloprevotella	25	15	10	25	15	10	17	11	6	8	4	4	17	12	5	2	1	1	5	1	4	
				Paraprevotella	10	4	6	10	4	6	9	4	5				9	3	6							
				Prevotella	27	14	13	27	14	13	20	7	13	7	7	0	19	10	9	3	2	1	5	2	3	
				Prevotella-1	2	1	1	2	1	1							2	1	1							
				Prevotella-9	5	3	2	5	3	2				5	3	2	3	2	1							

				Prevotellaceae_NK3B31_group	4	2	2	4	2	2	2	0	2	2	2	0	4	2	2						
				Prevotellaceae_UCG-001	12	8	4	12	8	4	5	3	2	7	5	2	9	6	3	2	1	1			
				Prevotellaceae_UCG-003	2	2	0	2	2	0				2	2	0	2	2	0						
			Rikenellaceae	Alistipes	28	12	16	28	12	16	24	10	14	4	2	2	20	10	10	4	1	3	4	1	3
				Rikenella	6	5	1	6	5	1	4	4	0	2	1	1	4	3	1						
				Rikenellaceae_RC9_gut_group	5	2	3	5	2	3	2	1	1	3	1	2	3	1	2						
Candidatus_Saccharibacteria				Candidatus_Saccharimonas	3	2	1	3	2	1	2	2	0												
				Candidatus_Stoquefichus	4	1	3	4	1	3				3	0	3	3	0	3						
Cyanobacteria		Spirulinales	Spirulinaceae	Spirulina	2	1	1	2	1	1				2	1	1	2	1	1						
Deferribacteres	Deferribacteres	Deferribacterales	Deferribacteraceae	Mucispirillum	11	7	4	11	7	4	10	7	3				10	6	4	2	2	0			
Elusimicrobia	Elusimicrobia	Elusimicrobiales	Elusimicrobiaceae	Elusimicrobium	2	0	2	2	0	2				2	0	2	2	0	2						
Euryarchaeota	Methanobacteria	Methanobacteriales	Methanobacteriaceae	Methanosphaera	2	1	1	2	1	1				2	1	1	2	1	1						
Firmicutes	Bacilli	Bacillales	Bacillaceae	Bacillus	8	3	5	8	3	5	5	1	4	3	2	1	7	2	5						
			Planococcaceae	Sporosarcina	3	3	0	3	3	0				2	2	0				2	2	0			
			Staphylococcaceae	Jeotgalicoccus	4	3	1	4	3	1				3	2	1				2	2	0			
				Macrococcus	2	2	0	2	2	0				2	2	0				2	2	0			
				Staphylococcus	10	6	4	10	6	4	8	5	3	2	1	1	7	5	2				3	1	2
		Gemellales	Gemellaceae	Gemella	3	1	2	3	1	2	2	1	1				2	1	1						
		Lactobacillales	Aerococcaceae	Aerococcus	5	4	1	5	4	1	3	3	0	2	1	1	2	2	0						
				Facklamia	3	3	0	3	3	0				2	2	0				2	2	0			
			Carnobacteriaceae	Atopostipes	2	2	0	2	2	0															
			Enterococcaceae	Enterococcus	16	10	6	15	9	6	11	6	5	4	3	1	12	7	5	2	2	0			
			Lactobacillaceae	Lactobacillus	84	27	57	83	26	57	51	14	37	32	12	20	57	19	38	20	7	13	5	1	4
			Streptococcaceae	Lactococcus	10	4	6	10	4	6	7	3	4	2	0	2	4	2	2	4	2	2			
				Streptococcus	16	10	6	15	9	6	7	3	4	8	6	2	12	7	5	2	1	1	2	1	1
		Turicibacterales	Turicibacteraceae	Turicibacter	21	7	14	21	7	14	17	6	11	3	1	2	16	5	11	4	2	2			
	Clostridia	Clostridiales	Caldicoprobacteraceae	Caldicoprobacter	3	0	3	3	0	3				3	0	3	3	0	3						
			Christensenellaceae	Christensenella	3	1	2	3	1	2				2	0	2	3	1	2						
				Christensenellaceae_R-7_Group	3	1	2	3	1	2	2	1	1				2	1	1						
			Clostridiaceae	Butyricicoccus	4	2	2	4	2	2	4	2	2				4	2	2						
				Candidatus_Arthromitus	10	8	2	10	8	2	7	5	2	3	3	0	8	7	1						

				Clostridium	19	10	9	19	10	9	11	7	4	8	3	5	15	8	7	4	2	2			
				Clostridium_sensu_stricto	9	8	1	9	8	1	5	5	0	3	2	1	4	4	0				3	2	1
				SMB53	3	1	2	3	1	2	2	1	1				2	0	2						
			Clostridiales_Family_XIII_Incertae_Sedis	Anaerovorax	4	2	2	4	2	2	2	2	0	2	0	2	4	2	2						
			Dehalobacteriaceae	Dehalobacterium	7	3	4	7	3	4	4	2	2	3	1	2	7	3	4						
			Eubacteriaceae	[Eubacterium]_coprostanoligenes_group	2	0	2	2	0	2							2	0	2						
				Anaerofustis	7	4	3	7	4	3	6	3	3				6	4	2						
			Family_XIII	Family_XIII_AD3011_group	3	3	0	3	3	0	3	3	0				2	2	0						
				Family_XIII_UCG_001	2	0	2	2	0	2							2	0	2						
			Lachnospiraceae	Acetatifactor	4	0	4	4	0	4	2	0	2	2	0	2	3	0	3						
				Anaerostipes	4	1	3	4	1	3	4	1	3				2	0	2						
				Blautia	18	13	5	18	13	5	7	4	3	10	8	2	13	10	3	3	2	1			
				Butyrivibrio	3	2	1	3	2	1				2	1	1	2	2	0						
				Coprococcus	19	9	10	19	9	10	9	6	3	10	3	7	16	6	10	3	3	0			
				Dorea	13	7	6	13	7	6	9	5	4	4	2	2	10	4	6				2	2	0
				Fusicatenibacter	2	1	1	2	1	1				2	1	1	2	1	1						
				Lachnobacterium	6	2	4	6	2	4	3	2	1	3	0	3	4	2	2	2	0	2			
				Lachnoclostridium	8	7	1	8	7	1	4	3	1	4	4	0	3	2	1	4	4	0			
				Lachnospira	8	4	4	8	4	4	4	2	2	3	1	2	7	3	4						
				Lachnospiraceae_NC2004_group	2	0	2	2	0	2															
				Lachnospiraceae_ND3007_group	2	1	1	2	1	1				2	1	1	2	1	1						
				Lachnospiraceae_NK4A136_group	16	6	10	16	6	10	9	4	5	7	2	5	9	4	5	3	1	2	2	0	2
				Lachnospiraceae_UCG-001	6	2	4	6	2	4	2	0	2	4	2	2	5	2	3						
				Lachnospiraceae_UCG-004	2	1	1	2	1	1															
				Lachnospiraceae_UCG-006	3	2	1	3	2	1	2	2	0				3	2	1						
				Marvinbryantia	10	4	6	10	4	6	3	1	2	7	3	4	9	4	5						
				Robinsoniella	2	2	0	2	2	0	2	2	0												
				Roseburia	14	2	12	14	2	12	6	2	4	8	0	8	11	2	9	2	0	2			

					Tyzzerella	2	1	1	2	1	1	2	1	1												
					Tyzzerella-3	2	0	2	2	0	2															
				Oscillospiraceae	Intestinimonas	6	2	4	6	2	4	4	1	3	2	1	1	4	2	2						
					Oscillibacter	20	15	5	20	15	5	10	8	2	10	7	3	16	13	3	2	1	1	2	1	1
				Peptococcaceae	Peptococcus	3	2	1	3	2	1				2	1	1				2	1	1			
					rc4_4	3	2	1	3	2	1	2	2	0							2	1	1			
				Peptostreptococcaceae	Peptostreptococcaceae_incertae_sedis	2	0	2	2	0	2							2	0	2						
					Romboutsia	9	3	6	9	3	6	2	2	0	7	1	6	6	3	3				2	0	2
					Terrisporobacter	2	0	2	2	0	2				2	0	2									
				Ruminococcaceae	Anaerotruncus	12	7	5	12	7	5	9	6	3	3	1	2	6	4	2	6	3	3			
					Clostridium_IV	5	2	3	5	2	3	5	2	3				4	1	3						
					Eubacterium	12	3	9	12	3	9	7	2	5	4	1	3	9	1	8						
					Faecalibacterium	4	1	3	4	1	3	2	0	2	2	1	1	4	1	3						
					Flavonifractor	3	2	1	3	2	1	3	2	1				2	2	0						
					Gemmiger	2	0	2	2	0	2	2	0	2				2	0	2						
					Harryflintia	3	1	2	3	1	2	2	1	1												
					Oscillospira	24	13	11	24	13	11	15	8	7	8	4	4	20	10	10	3	2	1			
					Pseudoflavonifractor	5	4	1	5	4	1	5	4	1				5	4	1						
					Ruminiclostridium	6	1	5	6	1	5	3	1	2	3	0	3	4	1	3	2	0	2			
					Ruminiclostridium-5	7	4	3	7	4	3				7	4	3	5	3	2						
					Ruminiclostridium-6	8	4	4	8	4	4	2	1	1	6	3	3	7	4	3						
					Ruminiclostridium-9	7	4	3	7	4	3				6	3	3	5	4	1	2	0	2			
					Ruminococcaceae_NK4A214_group	5	2	3	5	2	3				4	2	2	3	1	2	2	1	1			
					Ruminococcaceae_UCG-004	3	0	3	3	0	3							2	0	2						
					Ruminococcaceae_UCG-005	7	3	4	7	3	4				5	1	4	5	1	4						
					Ruminococcaceae_UCG-010	4	2	2	4	2	2	2	2	0				2	1	1						
					Ruminococcaceae_UCG-013	9	4	5	9	4	5	3	2	1	6	2	4	5	2	3	2	1	1			
					Ruminococcaceae_UCG-014	17	6	11	17	6	11	8	3	5	9	3	6	13	5	8	2	0	2	2	1	1
					Ruminococcus	32	16	16	32	16	16	20	8	12	12	8	4	24	12	12	6	3	3			
					Ruminococcus-1	7	4	3	7	4	3	2	1	1	5	2	2	6	3	3						
					Ruminococcus-2	3	2	1	3	2	1				2	1	1	3	2	1						
				Veillonellaceae	Phascolarctobacterium	6	3	3	6	3	3	2	1	1	4	2	2	6	3	3						

				Veillonella	5	3	2	5	3	2	2	1	1	3	2	1	5	3	2						
				Dialister	4	0	4	4	0	4	2	0	2	2	0	2	4	0	4						
		Lachnospirales	Lachnospiraceae	A2	2	0	2	2	0	2															
	Erysipeloreichia	Erysipelotrichales	Coprobacillaceae	Coprobacillus	4	3	1	4	3	1	3	2	1				3	2	1						
			Erysipelotrichaceae	Dubosiella	7	4	3	7	4	3	5	3	2	2	1	1	4	2	2	2	2	0			
				Allobaculum	35	16	19	35	16	19	26	10	16	9	6	3	27	12	15	6	4	2	2	0	2
				Erysipelatoclostridium	5	2	3	5	2	3	3	2	1	2	0	2	4	1	3						
				Faecalibaculum	5	2	3	5	2	3	4	1	3				4	2	2						
				Faecalitalea	2	1	1	2	1	1															
	Negativicutes	Selenomonadales	Veillonellaceae	Anaerovibrio	3	3	0	3	3	0				2	2	0	2	2	0						
	nonrank_Firmicutes sensu stricto incertae sedis	nonrank_Firmicutes sensu stricto incertae sedis	nonrank_Firmicutes sensu stricto incertae sedis	Negativibacillus	5	3	2	5	3	2	3	2	1	2	1	1	2	1	1						
Fusobacteria	Fusobacteria	Fusobacteriales	Fusobacteriaceae	Fusobacterium	2	1	1	2	1	1	2	1	1				2	1	1						
Proteobacteria	Alphaproteobacteria	Hyphomicrobiales	Brucellaceae	Ochrobactrum	3	3	0	3	3	0				3	3	0				2	2	0			
		Rhizobiales	Hyphomicrobiaceae	Devosia	2	0	2	2	0	2	2	0	2												
		Sphingomonadales	Sphingomonadaceae	Sphingomonas	4	2	2	4	2	2	2	2	0	2	0	2				3	1	2			
	Betaproteobacteria	Burkholderiales	Burkholderiaceae	Burkholderia	3	1	2	3	1	2															
				Cupriavidus	3	3	0	3	3	0				3	3	0				2	2	0			
				Ralstonia	2	1	1	2	1	1							2	1	1						
			Oxalobacteraceae	Massilia	2	2	0	2	2	0															
			Sutterellaceae	Parasutterella	10	5	5	10	5	5	7	3	4	3	2	1	7	3	4	3	2	1			
				Sutterella	6	4	2	6	4	2	6	4	2				4	3	1						
		Neisseriales	Neisseriaceae	Neisseria	2	0	2	2	0	2							2	0	2						
	Deltaproteobacteria	Desulfovibrionales	Desulfovibrionaceae	Bilophila	8	6	2	8	6	2	6	4	2	2	2	0	6	5	1	2	1	1			
				Desulfovibrio	25	14	11	25	14	11	16	9	7	9	5	4	18	11	7	8	3	5			
	Epsilonproteobacteria	Campylobacterales	Helicobacteraceae	Flexispira	2	2	0	2	2	0	2	2	0				2	2	0						
				Heilcobacter	21	16	5	21	16	5	18	14	4	3	2	1	19	14	5	2	2	0			
	Gammaproteobacteria	Enterobacterales	Enterobacteriaceae	Escherichia	2	2	0	2	2	0	2	2	0				2	2	0						
				Escherichia-Shigella	8	7	1	8	7	1	7	6	1				6	6	0	2	1	1			
				Klebsiella	5	4	1	5	4	1				4	3	1	4	3	1						
			Morganellaceae	Proteus	2	1	1	2	1	1															
		Pasteurellales	Pasteurellaceae	Aggregatibacter	3	3	0	3	3	0				3	3	0	3	3	0						
				Haemophilus	2	0	2	2	0	2				2	0	2	2	0	2						

		Pseudomonadales	Moraxellaceae	Acinetobacter	4	2	2	3	1	2	2	1	1				2	0	2						
				Psychrobacter	4	2	2	4	2	2				3	2	1	2	0	2	2	2	0			
			Pseudomonadaceae	Pseudomonas	2	1	1	2	1	1	2	1	1												
		Xanthomonadales	Xanthomonadaceae	Lysobacter	2	1	1	2	1	1															
Spirochaetae	Spirochaetes	Spirochaetales	Spirochaetaceae	Treponema-2	3	3	0	3	3	0				3	3	0	3	3	0						
Tenericutes	Mollicutes	Anaeroplasmatales	Anaeroplasmataceae	Anaeroplasma	11	3	8	11	3	8	8	1	7	3	2	1	9	3	6	2	0	2			
		Mycoplasmatales	Mycoplasmataceae	Mycoplasma	4	2	2	4	2	2				2	2	0	2	2	0						
Verrucomicrobia	Verrucomicrobiae	Verrucomicrobiales	Akkermansiaceae	Akkermansia	30	16	14	30	16	14	23	13	10	7	3	4	18	9	9	8	5	3	3	2	1
				ASF356	3	2	1	3	2	1	3	2	1												
				LARJ	2	1	1	2	1	1	2	1	1				2	1	1						
				LLKB	2	2	0	2	2	0	2	2	0												

Table S10. Commensal microbiota alterations at Species level in animal models of depression.

Microorganisms						Commensal microbiota			Gut microbiota																				
									Total			Mice			Rats			Monkey			Fecal samples			Cecum contents			Colon contents		
						Total no. of frequency	No. of increased	No. of decreased	Total no. of frequency	No. of increased	No. of decreased	Total no. of frequency	No. of increased	No. of decreased	Total no. of frequency	No. of increased	No. of decreased	Total no. of frequency	No. of increased	No. of decreased	Total no. of frequency	No. of increased	No. of decreased	Total no. of frequency	No. of increased	No. of decreased	Total no. of frequency	No. of increased	No. of decreased
**Phylum**	**Class**	**Order**	**Family**	**Genus**	**Species**																								
Actinobacteria	Actinomycetia	Bifidobacteriales	Bifidobacteriaceae	Bifidobacterium	Bifidobacterium_pseudolongum	3	1	2	3	1	2	3	1	2							3	1	2						
	Coriobacteriia	Eggerthellales	Eggerthellaceae	Adlercreutzia	Adlercreutzia_equolifaciens	2	1	1	2	1	1										2	1	1						
Bacteroidetes	Bacteroidia	Bacteroidales	Bacteroidaceae	Bacteroides	Bacteroides_acidifaciens	4	3	1	4	3	1	4	3	1							4	3	1						
					Bacteroides_fragilis	2	1	1	2	1	1				2	1	1				2	1	1						
					Bacteroides_massiliensis	2	0	2	2	0	2										2	0	2						
					Bacteroides_stercoris	2	2	0	2	2	0										2	2	0						
					Bacteroides_uniformis	2	1	1	2	1	1										2	1	1						
				Barnesiella	Barnesiella_intestinihominis	2	1	1	2	1	1										2	1	1						
			Muribaculaceae	Muribaculum	Muribaculum_intestinale	3	1	2	3	1	2	3	1	2							3	1	2						
			Odoribacteraceae	Butyricimonas	Butyricimonas_virosa	2	0	2	2	0	2	2	0	2							2	0	2						
			Prevotellaceae	Prevotella	Prevotella_ruminicola	2	1	1	2	1	1				2	1	1				2	1	1						
			Rikenellaceae	Alistipes	Alistipes_finegoldii	2	2	0	2	2	0										2	2	0						
Deferribacteres	Deferribacteres	Deferribacterales	Deferribacteraceae	Mucispirillum	Mucispirillum_schaedleri	4	4	0	4	4	0	3	3	0							4	4	0						
Firmicutes	Bacilli	Lactobacillales	Enterococcaceae	Enterococcus	Enterococcus_faecalis	4	2	2	4	2	2	2	0	2	2	2	0				2	0	2	2	2	0			
			Lactobacillaceae	Lactobacillus	Lactobacillus_animalis	4	2	2	4	2	2	4	2	2							3	1	2						
					Lactobacillus_intestinalis	7	1	6	7	1	6	5	1	4	2	0	2				6	1	5						
					Lactobacillus_johnsonii	5	1	4	5	1	4	4	1	3							5	1	4						
					Lactobacillus_murinus	6	2	4	6	2	4	4	1	3							6	2	4						
					Lactobacillus_reuteri	11	4	7	11	4	7	7	2	5	2	1	1				11	4	7						
				Limosilactobacillus	Lactobacillus_equigenerosi	2	0	2	2	0	2										2	0	2						
			Streptococcaceae	Streptococcus	Streptococcus_hyointestinalis	2	1	1	2	1	1										2	1	1						
	Clostridia	Clostridiales	Clostridiaceae	CandidatusArthromitus	Segmented_filamentous_bacteria	6	5	1	6	5	1	4	4	0	2	1	1				6	5	1						
				Clostridium	Clostridium_fusiformis	2	1	1	2	1	1	2	1	1							2	1	1						
					Clostridium_histolyticum	2	1	1	2	1	1				2	1	1							2	1	1	2	1	1

			Eubacteriaceae	Eubacterium	Eubacterium_plexicaudatum	2	1	1	2	1	1	2	1	1							2	1	1						
					Eubacterium_ruminantium	2	1	1	2	1	1				2	1	1				2	1	1						
			Lachnospiraceae	Blautia	Blautia_glucerasea	2	0	2	2	0	2										2	0	2						
					Blautia_hydrogenotrophica	2	2	0	2	2	0										2	2	0						
			Oscillospiraceae	Oscillibacter	Oscillibacter_valericigenes	2	1	1	2	1	1				2	1	1				2	1	1						
			Ruminococcaceae	ClostridiumIV	Clostridium_leptum	2	2	0	2	2	0	2	2	0															
				Faecalibacterium	Faecalibacterium_prausnitzii	2	1	1	2	1	1																		
				Flavonifractor	Flavonifractor_plautii	3	2	1	3	2	1	2	2	0							3	2	1						
				Ruminococcus	Ruminococcus_bromii	2	2	0	2	2	0										2	2	0						
					Ruminococcus_lactaris	2	0	2	2	0	2	2	0	2							2	0	2						
Proteobacteria	Epsilonproteobacteria	Campylobacterales	Helicobacteraceae	Helicobacter	Helicobacter_ganmani	2	2	0	2	2	0	2	2	0							2	2	0						
					Helicobacter_japonicus	2	2	0	2	2	0	2	2	0							2	2	0						
					Helicobacter_macacae	2	0	2	2	0	2							2	0	2				2	0	2			
					Helicobacter_rodentium	4	4	0	4	4	0	3	3	0							4	4	0						
	Gammaproteobacteria	Enterobacterales	Enterobacteriaceae	Escherichia	Escherichia_coli	7	4	3	7	4	3	4	1	3	3	3	0				5	2	3						
		Pseudomonadales	Moraxellaceae	Acinetobacter	Acinetobacter_calcoaceticus	2	1	1	2	1	1				2	1	1												
Verrucomicrobia	Verrucomicrobiae	Verrucomicrobiales	Akkermansiaceae	Akkermansia	Akkermansia_muciniphila	3	3	0	3	3	0	2	2	0							3	3	0						
					FJ880724	2	1	1	2	1	1	2	1	1															

Table S11. Characteristics of studies investigating the efficiency of gut microbiota-based therapeutics in patients with depression.

**Study**	**Depression model**	**Microbiota-based therapies**	**Genus**	**Species**	**Intervention method**	**Depression alleviation**	**Gut-brain axis mechanism**	**OCEBM evidence level**
Kilinçarslan S et al. 2020[1]	IBD patients with depression symptoms	Fecal microbiota transplantation	_	_	The fresh stool from healthy donors was diluted with saline before transplantation, and the suspension was prepared by mixing with a spatula. The stool suspension was infused into the patient through colonoscopy	The severity of anxiety, depression and obsession in IBD patients decreased after FMT	_	3
Kurokawa S et al. 2018[2]	IBS patients with depression symptoms	Fecal microbiota transplantation	_	_	Approximately 100 g of feces were collected from the pack, dissolved in 200 mL of saline	Depression and anxiety symptoms were improved by FMT regardless of gastrointestinal symptom change in patients with IBS	FMT altered disordered fecal microbiota	3
Lin H et al. 2021[3]	IBS patients with depression symptoms	Fecal microbiota transplantation	_	_	The donor is a healthy 36-year-old male. Patients received FMT treatment (oral administration) from May 2019 to December 2019. The patients took the intestinal flora capsules 3 times in total, once every other day, 30 capsules each time	FMT treatment can effectively alleviate the anxiety and depression behaviors of IBS-D patients	FMT treatment regulated the gut microbiota	2
Guo Q et al. 2021[4]	IBS patients with depression symptoms	Fecal microbiota transplantation with enterobacteria capsules	_	_	The FMT treatment was intervened by oral enteric capsules for 3 times (every 2 days one time and 30 capsules each time)	FMT therapy alleviated anxiety/depression symptom in IBS patients	FMT therapy restored the intestinal micro-ecology	2
Chinna Meyyappan A et al. 2022[5]	Patients with MDD and GAD	Multispecies probiotics	_	Microbial Ecosystem Therapeutic-2 (MET-2), which contained 40 strains of bacteria	During the 8 weeks of treatment, all participants consumed three MET-2 capsules per day orally; each 0.5-g MET-2 capsule contains 3.2×10^{5} to 3.2×10^{11} CFU	Over the course of 10 weeks, MET-2 significantly decreased mean MADRS and GAD-7 scores	_	3
Dao VH et al. 2021[6]	Chronic gastrointestinal disorders patients wiht depression symptoms	Multispecies probiotics	Lactobacillus, Bifidobacterium, Lactococcus	B.bifidum W23, B. lactis W52, L. acidophilus W37, L. brevis W63, L. casei W56, L. salivarius W24, Lc. lactis W19, Lc. lactis W58	The product consists of over $2,5 \times 10^{9}$ CFU per gram, patients would mix one sachet (2g of product) in 100mL of water for 8 weeks	After 2 months using the probiotic product, the symptoms of anxiety and depression improved significantly	_	3
Venkataraman R et al. 2021[7]	Students facing examination stress	Multi-strain probiotics	Bacillus, Lactobacillus, Bifidobacterium	Bacillus coagulans Unique IS2, L. rhamnosus UBLR58, B. lactis UBBLa70, L. plantarum UBLP40, B. breve UBBr01, B. infantis UBBI01	Receiving the multi-strain probiotic (Bacillus coagulans Unique IS2, L. rhamnosus UBLR58, B. lactis UBBLa70, L. plantarum UBLP40 (each of 2 billion CFU); B. breve UBBr01, B. infantis UBBI01	Supplementation of multistrain probiotic significantly reduced the depressive symptoms in students facing examination		2

					(each of 1 billion CFU)) capsule with glutamine (250 mg) 2 times a day for 28 days			
Akkasheh G et al. 2016[8]	Patients with MDD	Probiotics	Lactobacillus, Bifidobacterium	L.acidophilus, L. casei, B. bifidum	Patients in the probiotic group received daily one probiotic capsule containing L. acidophilus (2×10^9 CFU/g), L. casei (2×10^9 CFU/g), and B. bifidum (2×10^9 CFU/g) for 8 weeks	After 8 wk of intervention, patients who received probiotic supplements had significantly decreased Beck Depression Inventory total scores	Probiotic administration in patients with MDD for 8 wk had beneficial effects insulin, homeostasis model assessment of insulin resistance, hs-CRP concentrations, and glutathione concentrations	2
Baião R et al. 2022[9]	Patients with moderate depression	Probiotics	Bacillus, Bifidobacterium, Lactobacillus, Streptococcus	14 species: B. subtilis PXN® 21, B. bifidum PXN® 23, B. breve PXN® 25, B. infantis PXN® 27, B. longum PXN® 30, L. acidophilus PXN® 35, L. delbrueckii ssp. bulgaricus PXN® 39, L. casei PXN® 37, L. plantarum PXN® 47, L. rhamnosus PXN® 54, L. helveticus PXN® 45, L. salivarius PXN® 57, L. lactis ssp. lactis PXN® 63, S. thermophilus PXN® 66	Participants were asked to take four capsules （2×10^9 CFU）in the morning each day with food within 4 week	Probiotic intake significantly reduced depression scores on the Patient Health Questionnaire 9	_	2
Bambling M et al. 2017[10]	Patients with SSRI treatment resistant depression	Probiotics	Lactobacillus, Bifidobacterium, Streptoccocus	L. acidophilus, B. bifidum, S. thermophiles	Capsules administered pre meals as a combination of lyophilized probiotics (L. acidophilus, B. bifidum, S. thermophiles total CFU of 2×10^{10}) and magnesium orotate 1600 mg divided in two daily doses for 8 weeks	At the end of an 8-week intervention mean changes for depression scores and quality of life in the group was clinically significantly improved	_	3
Chen HM et al. 2021[11]	Patients with MDD	Probiotics	Lactobacillus	L. plantarum PS128 (PS128)	One PS128 capsule twice a day was gaven to recruited patients, each PS128 capsule contains 300 mg of probiotics, equivalent to 3×10^{10} CFU of Lactobacillus plantarum PS128	After 8-week PS128 intervention, scores of Hamilton Depression Rating Scale-17 and Depression and Somatic symptoms Scale significantly decreased	_	3
Jamilian M et al. 2018[12]	Women with polycystic ovary syndrome	Probiotics	Lactobacillus, Bifidobacterium	L. acidophilus, L. reuteri, L. fermentum, B. bifidum	Intaking 8×10^9 CFU/day probiotic containing L. acidophilus, L. reuteri, L. fermentum, B. bifidum (2×10^9 CFU/g each) plus 200 μg/day selenium for 12 weeks	Probiotic and selenium co-supplementation resulted in a significant improvement in beck depression inventory and depression anxiety and stress scale scores compared with the placebo	Co-administration of probiotic and selenium for 12 weeks to women with PCOS had beneficial effects on mental health parameters, serum total testosterone, hirsutism, hs-CRP, TAC, GSH and MDA levels	2
Kazem YI et al. 2021[13]	Healthy female volunteers	Probiotics	Bifidobacterium	Yogurt enriched with Bifidobacterium spp.	All volunteers were given seven cups of yogurt every week (as one cup daily) fortified with the strain specific probiotic which was Bifidobacterium spp. for 8 weeks provided that each cup of yogurt was weighing 100 g	Bifidobacterium spp. supplementation combined with improvement in dietary intake resulted in improvement of depressive mood and well-being	Bifidobacterium spp. supplementation reduced kynurenine blood level	3

					and each gram contained not less than 10^8 cells of Bifidobacterium spp.			
Kim CS et al. 2019[14]	Nationwide individuals	Probiotics	_	_	The types of probiotic food included fermented vegetables (kimchi) and fermented milk products	Compared with the lowest tertile of probiotic food consumption, the highest tertile had significantly lower odds in PHQ-9 depression severity and self-reported clinical depression, particularly in men	_	4
Lee HJ et al. 2021[15]	Healthy adults	Probiotics	Lactobacillus, Bifidobacterium	Probiotic NVP-1704, a mixture of L. reuteri NK33 and B. adolescentis NK98	Each 500 mg capsule of NVP-1704 contained 2.5×10^9 CFU of microorganisms (2.0×10^9 CFU for L. reuteri NK33 and 0.5×10^9 CFU for B. adolescentis NK98), two capsules with water were taken once a day, daily, for eight weeks	NVP-1704 group had a more significant reduction in depressive symptoms at four and eight weeks of treatment, and anxiety symptoms at four weeks compared to the placebo group	NVP-1704 treatment decreased serum interleukin-6 levels, and regulated the gut microbiota composition	2
Majeed M et al. 2018[16]	MDD patients with IBS	Probiotics	Bacillus	Bacillus coagulans MTCC 5856	Receving B. coagulans MTCC 5856 at a daily dose of 2×10^9 CFU (2 billion spores) for 90 days	B. coagulans MTCC 5856 reduced the depression symptoms in MDD patients with IBS (HAM-D, MADRS)	B. coagulans MTCC 5856 reduced the level of serum myeloperoxidase	2
Messaoudi M et al. 2011[17]	Healthy human volunteers	Probiotics	Lactobacillus, Bifidobacterium	L. helveticus R0052, B. longum R0175	During or just after breakfast, all volunteers took one stick of 1 5 g/day of probiotics containing L. helveticus R0052 and B. longum R0175 (3×10^9 CFU/stick) for 30 days	Administration of probiotics significantly alleviated depression and anxiety in volunteers, as measured by the HSCL-90 scale	_	2
Mi GL et al. 2015[18]	Infantile colic and colicky induced maternal depression	Probiotics	Lactobacillus	L. reuteri (DSM 17938)	Participants received L. reuteri at a dose 10^8 CFU for 28 days and they were followed for 4 weeks	L. reuteri (DSM 17938) reduces daily crying time and maternal depression during infantile colic	_	2
Miyaoka T et al. 2018[19]	Patients with treatment-resistant MDD	Probiotics	Clostridium	C. butyricum MIYAIRI 588 (CBM588)	20 mg orally twice daily for the first week and 20 mg orally three times daily from weeks 2 to 8	CBM588 in combination with antidepressants is effective and well tolerated in the treatment of treatment-resistant MDD	_	2
Mohammadi AA et al. 2016[20]	Petrochemical workers	Probiotics	Lactobacillus, Bifidobacterium	L. lactis, L. acidophilus, Bifidobacterium	Probiotic and conventional yogurts or multispecies probiotic supplements were provided for participants every day for 6 weeks	After 6 weeks of intervention, a significant improvement of DASS scores was observed in the probiotic yogurt and in the probiotic capsule group	_	2
Moludi J et al. 2019[21]	Myocardial infarction patients with depression symptoms	Probiotics	Lactobacillus	L. rhamnosus	Patients in the probiotic group received one probiotic capsule daily containing 1.6×10^9 CFU of L. rhamnosus with their lunch for 12 weeks	The total BDI-II score decreased significantly in patients who received probiotic supplements compared with the placebo group	Markers of inflammatory and oxidative stress were influenced favorably by probiotic supplements	2

Okubo R et al. 2019[22]	Schizophrenia patients with depression symptoms	Probiotics	Bifidobacterium	B. breve A-1	All participants received B. breve strain A-1 (10^{11} CFU/day) for 4 weeks followed by 4 weeks of observation	B. breve A-1 in improved anxiety and depressive symptoms in patients with schizophrenia	B. breve A-1 administration changed the function of the gut epithelial barrier function related to TRANCE and IL-22	3
Otaka M et al. 2021[23]	Patients with MDD or BD	Probiotics	Lactobacillus	L. casei strain Shirota (LcS)	Daily intake of 8.0×10^{10} CFU for 12 weeks	Depression severity, evaluated by the Hamilton Depression Rating Scale, was significantly alleviated after LcS treatment	LcS was beneficial to alleviate depressive symptoms, partly through its association with abundance of Actinobacteria in the gut microbiota	3
Pinto-Sanchez MI et al. 2017[24]	IBS patients with depression symptoms	Probiotics	Bifidobacterium	B. longum NCC3001	Receiving 42 sachets of spray dried B. longum NCC3001 (1.0E+10 CFU/1gram powder with maltodextrin)	Probiotic B. longum NCC3001 reduced depression scores and increased quality of life in patients with IBS	The probiotic reduced limbic reactivity	2
Qin Q et al. 2021[25]	Patients with test anxiety	Probiotics	Lactobacillus, Bifidobacterium, Streptococcus	1.2×10^{10} CFU of B. longum subsp. Longum BAMA-B05/BauB1024, 1.9×10^{10} CFU of B. lactis BAMA-B06/Bau-B0111, 1.5×10^{10} CFU of B. adolescentis, 3.2×10^{9} CFU of S. thermophiles, 4.6×10^{9} CFU of L. acidophilus, 3.0×10^{9} CFU of L. delbrueckii subsp. bulgaricus	Taking probiotic supplement preparation for 15 consecutive days (twice per day, and approximately 12-hour set time between two intakes)	Administration of the PSP markedly alleviated students' depression	_	3
Raygan F et al. 2018[26]	Type 2 diabetic patients with depression symptoms	Probiotics	Lactobacillus, Bifidobacterium	L. acidophilus, B. bifidum, L. reuteri, L. fermentum	Intaking 50,000 IU vitamin D3 every 2 weeks plus 8×10^{9} CFU/g probiotic, containing L. acidophilus, B. bifidum, L. reuteri, and L. fermentum (each 2×10^{9}) for 12 weeks	Vitamin D and probiotic co-supplementation resulted in significant improvements in beck depression inventory total scores	Vitamin D and probiotic co-supplementation regulated serum hs-CRP, plasma NO, TAC, glycemic control and HDL-cholesterol levels	2
Raygan F et al. 2019[27]	Type 2 diabetic patients with depression symptoms	Probiotics	Lactobacillus, Bifidobacterium	L. acidophilus, B. bifidum, L. reuteri, L. fermentum	Receiving 200 mg/day selenium as selenium yeast plus 8×10^{9} CFU/ day probiotic containing L. acidophilus, L. reuteri, L. fermentum and B. bifidum (2×10^{9} CFU/g each) for 12 weeks	Probiotic and selenium co-supplementation significantly decreased Beck Depression Inventory index	Probiotic and selenium co-supplementation improved metabolic profiles	2
Sanchez M et al. 2017[28]	Obese individuals	Probiotics	Lactobacillus	L. rhamnosus CGMCC1.3724	Participants received two capsules per day , corresponding to an average of 3.24×10^{8} CFU/day for 24 weeks	The LPR female group displayed a more pronounced decrease in the Beck Depression Inventory score	_	2
Slykerman RF et al. 2017[29]	Postpartum depression	Probiotics	Lactobacillus	L. rhamnosus HN001	Women were randomised to receive either HN001 at a dose of 6×10^{9} CFU,to be taken daily from enrolment until birth and, from birth up till six months post-birth whilst breastfeeding.	Women who received HN001 had significantly lower depression and anxiety scores in the postpartum period	_	2

Tian P et al. 2022[30]	Patients with MDD	Probiotics	Bifidobacterium	B. breve CCFM1025	The freeze-dried CCFM1025 in a dose of viable bacteria of 10^{10} CFU was given to MDD patients daily for four weeks	B. breve CCFM1025 showed a better antidepressant-like effect than placebo, based on the HDRS-24 and MADRS evaluation	B. breve CCFM1025 changed in the gut microbiome and tryptophan metabolism	2
Wallace CJK et al. 2021[31]	Patients with MDD	Probiotics	Lactobacillus, Bifidobacterium	L. helveticus R0052 and B. longum R0175 (CEREBIOME®)	Participants consumed a probiotic supplement containing Lactobacillus helveticus R0052 and Bifidobacterium longum R0175 (CEREBIOME®) at a dose of 3×10^9 CFU once per day for 8 weeks	Significant improvements in depressive symptoms were observed at week 4 and were sustained at week 8	_	3
Wu SI et al. 2021[32]	Information technology specialists	Probiotics	Lactobacillus	Lactobacillus plantarum PS128TM (PS128TM)	Participants were asked to take two capsules containing PS128TM powder, equivalent to 20 billion CFU, daily for 8 weeks	After 8-week-intervention, participants showed significant decreases in the levels of depression (PHQ-9)	_	3
Yamamura R et al. 2021[33]	Schizophrenic patients with depression symptoms	Probiotics	Bifidobacterium	B. breve A-1 (synonym B. breve MCC1274)	For the first 4 weeks, the participants consumed two 2-g sachets of freeze-dried Bifidobacterium breve A-1 (synonym B. breve MCC1274) per day, each containing 5.0×10^{10} CFU	Probiotic treatment with B. breve A-1 alleviated anxiety and depressive symptoms in patients with schizophrenia	It is suggested that an elevated lipid and energy metabolism at baseline might be associated with the effects of probiotics on anxiety and depressive symptoms	3
Zhang X et al. 2021[34]	Patients with depression and constipation	Probiotics	Lactobacillus	Fermented Milk Containing L. paracasei Strain Shirota (LcS)	The subjects consumed 100 mL of a LcS beverage (10^8 CFU/mL) every day for 9 weeks.	Daily consumption of LcS for 9 weeks appeared to relieve constipation and improve the potentially depressive symptoms in patients	LcS supplementation ignificantly decreased the IL-6 levels, and appeared to regulate the intestinal microbiota related to mental illness	2
Kavyani M et al. 2021[35]	NAFLD patients with depression symptoms	Prebiotics	_	Resistant dextrin	The intervention group received 15% of the total daily intake of fat (~20 g) as Camelina sativa oil with 10 g/day of resistant dextrin, 5 g at breakfast and 5 g at dinner for 12 weeks	Supplementation of Camelina sativa oil + resistant dextrin for 12 weeks improved depression symptoms (DASS scores) in patients with NAFLD	Prebiotic and CSO co-supplementation improved glycemic status, metabolic endotoxemia, inflammation, oxidant/antioxidant biomarkers	2
Miki T et al. 2016[36]	Japanese employees	Prebiotics	_	Dietary fiber	Dietary intake for 58 food and beverage items, energy, and selected nutrients were estimated using an ad hoc computer algorithm for the BDHQ	Dietary fiber intake from vegetables and fruits was significantly inversely associated with depressive symptoms	_	4
Park M et al. 2020[37]	Patients with depression	Prebiotics	_	Flavonoids	Participants consumed flavonoid-rich orange juice (serving a daily 380 mL, 600 ± 5.4 mg flavonoids) for 8 weeks	Flavonoid-rich orange juice treatment significantly decreased the depression symptoms (CES-D)	Flavonoid-rich orange juice treatment regulated the gut microbiome	2
Heidarzadeh-Rad N et al. 2020[38]	Patients with MDD	Probiotics, prebiotics	Lactobacillus, Bifidobacterium	L. helveticus R0052, B. longum R0175, and galactalogosaccharide (GOS)	The probiotic sachet containing 10 billion (≥ 10 × 109) CFU of freeze-dried L. helveticus R0052 and B. longum R0175, the prebiotic sachets contained 80% GOS powder per sachet. Participants were instructed to consume 1 sachet at the same time daily for 8 weeks	Eight-week supplementation with B. longum and L. helveticus in depressive patients improved depression symptoms	Probiotics supplementation resulted in significantly higher serum BDNF levels	2

Karbownik MS et al. 2022[39]	Psychiatrically healthy medical students	Probiotics, prebiotics	_	Fermented food and food-derived prebiotics	An electronic open-ended form of the Food Record was constructed to gather information regarding consumption of 34 selected food items categorized in five classes	High intake of fermented food was associated with more severe depressive and anxiety symptoms under stress; however, no such link was observed for food-derived prebiotics	_	3
Kazemi A et al. 2019[40]	Patients with MDD	Probiotics, prebiotics	Lactobacillus, Bifidobacterium	L. helveticus R0052, B. longum R0175, and galactooligosaccharide	The probiotic product contains freeze-dried L. helveticus R0052 and B. longum R0175 bacteria at a dosage 10 × 109 CFU/per 5 g sachet for 8 weeks, and the prebiotic product was composed of galactooligosaccharide and 0.2% Plum flavor	8 weeks of probiotic supplements to subjects with MDD resulted in an improvement in BDI score compared with placebo whereas no significant effect of prebiotic supplementation was seen	The probiotic may exert at least part of its effects on depression through the kynurenine to tryptophan ratio	2
Moludi J et al. 2021[41]	Coronary artery disease (CAD) patients with depression symptoms	Probiotics, prebiotics	Lactobacillus	L. rhamnosus G	Reciving one capsule contained 1.9 × 10^9 CFU of L. rhamnosus per day, or one sachet containing 15 g inulin per day, or both for 8 weeks	Probiotic-Inulin Co-supplementation significantly decreased depression symptoms (BDI)	Probiotic plus prebiotic may exert their effects on depression through the inflammatory cytokines and LPS	2
Perez-Cornago A et al. 2016[42]	Spanish university graduates	Probiotics, prebiotics	_	Yogurt (total, wholefat, and low-fat) and prebiotic ((fructans and galacto-oligosaccharide))	Participants were allocated into 4 categories according to servings (1 serving = 125 g) of yogurt (total, whole-fat (3% fat), and low-fat (;0.1% fat)] consumed per week: <0.5 servings (<63 g), ≥0.5 to <3 servings (≥63 to <250 g), ≥3 to <7 servings (≥250 to <875 g), and ≥7 servings (≥875 g)	High consumption of whole-fat yogurt was related to a lower risk of depression in women of the SUN cohort	_	3
Hadi A et al. 2019[43]	Obese or overweight adults	Synbiotics	Lactobacillus, Bifidobacterium	Synbiotics containing L. acidophilus, L. casei, B. bifidum plus inulin	Synbiotic supplements in form of a 500 mg capsule contained L. acidophilus, L. casei and B. bifidum (2 × 10^9 CFU/g each) plus 0.8 g inulin for 8 weeks	A significant between-group decrease in depression was found in the synbiotic group compared to the placebo.	Synbiotic supplementation decreased the TG, TC, LDL-C levels	2
Haghighat N et al. 2021a[44]	Hemodialysis patients	Synbiotics, probiotics	Lactobacillus, Bifidobacterium	Synbiotic (15 g of prebiotics, 5 g of probiotic containing L. acidophilus T16, B. bifidum BIA-6, B. lactis BIA-7, B. longum BIA-8 (2.7 × 10^7 CFU/g each))<br>prebiotics (5 g fructo-oligosaccharides (FOS), 5 g galacto-oligosaccharides (GOS), 5 g of inulin)	Receiving the synbiotic (15 g of prebiotics, 5 g of probiotic containing L. acidophilus T16, B. bifidum BIA-6, B. lactis BIA-7, B. longum BIA-8 (2.7 × 107 CFU/g each)) or probiotics (5 g probiotics as in synbiotic group) for 12 weeks	Synbiotic supplementation resulted in greater improvement in depression symptoms compared to the probiotic supplementation in HD patients especially in the subgroup of patients with depression symptoms	Synbiotic supplementation increased the serum BDNF level	2
Haghighat N et al. 2021b[45]	Hemodialysis patients	Synbiotics, probiotics	Lactobacillus, Bifidobacterium	Synbiotic (15 g of prebiotics, 5 g of probiotic containing L. acidophilus T16, B. bifidum BIA-6, B. lactis BIA-7, B. longum BIA-8 (2.7 × 10^7 CFU/g each))<br>prebiotics (5 g fructo-oligosaccharides (FOS), 5 g galacto-oligosaccharides (GOS), 5 g of inulin)	Receiving the synbiotic (15 g of prebiotics, 5 g of probiotic containing L. acidophilus T16, B. bifidum BIA-6, B. lactis BIA-7, B. longum BIA-8 (2.7 × 107 CFU/g each)) or probiotics (5 g probiotics as in synbiotic group) for 12 weeks	From baseline to 12 weeks, synbiotic and probiotic supplementation resulted in a significant decrease in BDI and BAI score in comparison to the placebo	Synbiotic and probiotic supplementation increaded the serum Hb level	2

References

1. S. Kilinçarslan and A. Evrensel, *Actas Esp Psiquiatr* **2020**, *48*, 1-7.

2. S. Kurokawa, T. Kishimoto, S. Mizuno, T. Masaoka, M. Naganuma, K. C. Liang, M. Kitazawa, M. Nakashima, C. Shindo, W. Suda, M. Hattori, T. Kanai and M. Mimura, *J Affect Disord* **2018**, *235*, 506-512.

3. H. Lin, Q. Guo, Z. Wen, S. Tan, J. Chen, L. Lin, P. Chen, J. He, J. Wen and Y. Chen, *Microb Cell Fact* **2021**, *20*, 233.

4. Q. Guo, H. Lin, P. Chen, S. Tan, Z. Wen, L. Lin, J. He, J. Wen and S. Lu, *Bioengineered* **2021**, *12*, 11885-11897.

5. A. Chinna Meyyappan, E. Forth and R. Milev, *Interact J Med Res* **2022**, *11*, e32234.

6. V. H. Dao, L. B. Hoang, T. O. Trinh, T. T. T. Tran and V. L. Dao, *J Multidiscip Healthc* **2021**, *14*, 1395-1402.

7. R. Venkataraman, R. S. Madempudi, J. Neelamraju, J. J. Ahire, H. R. Vinay, A. Lal, G. Thomas and S. Stephen, *Probiotics Antimicrob Proteins* **2021**, *13*, 12-18.

8. G. Akkasheh, Z. Kashani-Poor, M. Tajabadi-Ebrahimi, P. Jafari, H. Akbari, M. Taghizadeh, M. R. Memarzadeh, Z. Asemi and A. Esmaillzadeh, *Nutrition* **2016**, *32*, 315-20.

9. R. Baião, L. P. Capitão, C. Higgins, M. Browning, C. J. Harmer and P. W. J. Burnet, *Psychol Med* **2022**, 1-11.

10. M. Bambling, S. C. Edwards, S. Hall and L. Vitetta, *Inflammopharmacology* **2017**, *25*, 271-274.

11. H. M. Chen, P. H. Kuo, C. Y. Hsu, Y. H. Chiu, Y. W. Liu, M. L. Lu and C. H. Chen, *Nutrients* **2021**, *13*,

12. M. Jamilian, S. Mansury, F. Bahmani, Z. Heidar, E. Amirani and Z. Asemi, *J Ovarian Res* **2018**, *11*, 80.

13. Y. I. Kazem, M. H. Mahmoud, H. A. Essa, O. Azmy, W. A. Kandeel, M. Al-Moghazy, I. El-Attar, A. Hasheesh and N. S. Mehanna, *J Complement Integr Med* **2021**,

14. C. S. Kim and D. M. Shin, *Nutrition* **2019**, *63-64*, 169-174.

15. H. J. Lee, J. K. Hong, J. K. Kim, D. H. Kim, S. W. Jang, S. W. Han and I. Y. Yoon, *Nutrients* **2021**, *13*,

16. M. Majeed, K. Nagabhushanam, S. Arumugam, S. Majeed and F. Ali, *Food Nutr Res* **2018**, *62*,

17. M. Messaoudi, R. Lalonde, N. Violle, H. Javelot, D. Desor, A. Nejdi, J. F. Bisson, C. Rougeot, M. Pichelin, M. Cazaubiel and J. M. Cazaubiel, *Br J Nutr* **2011**, *105*, 755-64.

18. G. L. Mi, L. Zhao, D. D. Qiao, W. Q. Kang, M. Q. Tang and J. K. Xu, *Antonie Van Leeuwenhoek* **2015**, *107*, 1547-53.

19. T. Miyaoka, M. Kanayama, R. Wake, S. Hashioka, M. Hayashida, M. Nagahama, S. Okazaki, S. Yamashita, S. Miura, H. Miki, H. Matsuda, M. Koike, M. Izuhara, T. Araki, K. Tsuchie, I. A. Azis, R. Arauchi, R. A. Abdullah, A. Oh-Nishi and J. Horiguchi, *Clin Neuropharmacol* **2018**, *41*, 151-155.

20. A. A. Mohammadi, S. Jazayeri, K. Khosravi-Darani, Z. Solati, N. Mohammadpour, Z. Asemi, Z. Adab, M. Djalali, M. Tehrani-Doost, M. Hosseini and S. Eghtesadi, *Nutr Neurosci* **2016**, *19*, 387-395.

21. J. Moludi, M. Alizadeh, M. H. S. Mohammadzad and M. Davari, *Psychosom Med* **2019**, *81*, 770-777.

22. R. Okubo, M. Koga, N. Katsumata, T. Odamaki, S. Matsuyama, M. Oka, H. Narita, N. Hashimoto, I. Kusumi, J. Xiao and Y. J. Matsuoka, *J Affect Disord* **2019**, *245*, 377-385.

23. M. Otaka, H. Kikuchi-Hayakawa, J. Ogura, H. Ishikawa, Y. Yomogida, M. Ota, S. Hidese, I. Ishida, M. Aida, K. Matsuda, M. Kawai, S. Yoshida and H. Kunugi, *Microorganisms* **2021**, *9*,

24. M. I. Pinto-Sanchez, G. B. Hall, K. Ghajar, A. Nardelli, C. Bolino, J. T. Lau, F. P. Martin, O. Cominetti, C. Welsh, A. Rieder, J. Traynor, C. Gregory, G. De Palma, M. Pigrau, A. C. Ford, J. Macri, B. Berger, G. Bergonzelli, M. G. Surette, S. M. Collins, P. Moayyedi and P. Bercik, *Gastroenterology* **2017**, *153*, 448-459.e8.

25. Q. Qin, H. Liu, Y. Yang, Y. Wang, C. Xia, P. Tian, J. Wei, S. Li and T. Chen, *Dis Markers* **2021**, *2021*, 5597401.

26. F. Raygan, V. Ostadmohammadi, F. Bahmani and Z. Asemi, *Prog Neuropsychopharmacol Biol Psychiatry* **2018**, *84*, 50-55.

27. F. Raygan, V. Ostadmohammadi and Z. Asemi, *Clin Nutr* **2019**, *38*, 1594-1598.

28. M. Sanchez, C. Darimont, S. Panahi, V. Drapeau, A. Marette, V. H. Taylor, J. Doré and A. Tremblay, *Nutrients* **2017**, *9*,

29. R. F. Slykerman, F. Hood, K. Wickens, J. M. D. Thompson, C. Barthow, R. Murphy, J. Kang, J. Rowden, P. Stone, J. Crane, T. Stanley, P. Abels, G. Purdie, R. Maude and E. A. Mitchell, *EBioMedicine* **2017**, *24*, 159-165.

30. P. Tian, Y. Chen, H. Zhu, L. Wang, X. Qian, R. Zou, J. Zhao, H. Zhang, L. Qian, Q. Wang, G. Wang and W. Chen, *Brain Behav Immun* **2022**, *100*, 233-241.

31. C. J. K. Wallace and R. V. Milev, *Front Psychiatry* **2021**, *12*, 618279.

32. S. I. Wu, C. C. Wu, P. J. Tsai, L. H. Cheng, C. C. Hsu, I. K. Shan, P. Y. Chan, T. W. Lin, C. J. Ko, W. L. Chen and Y. C. Tsai, *Front Nutr* **2021**, *8*, 614105.

33. R. Yamamura, R. Okubo, N. Katsumata, T. Odamaki, N. Hashimoto, I. Kusumi, J. Xiao and Y. J. Matsuoka, *J Pers Med* **2021**, *11*,

34. X. Zhang, S. Chen, M. Zhang, F. Ren, Y. Ren, Y. Li, N. Liu, Y. Zhang, Q. Zhang and R. Wang, *Nutrients* **2021**, *13*,

35. M. Kavyani, S. Saleh-Ghadimi, P. Dehghan, M. Abbasalizad Farhangi and M. Khoshbaten, *Food Funct* **2021**, *12*, 8594-8604.

36. T. Miki, M. Eguchi, K. Kurotani, T. Kochi, K. Kuwahara, R. Ito, Y. Kimura, H. Tsuruoka, S. Akter, I. Kashino, I. Kabe, N. Kawakami and T. Mizoue, *Nutrition* **2016**, *32*, 584-9.

37. M. Park, J. Choi and H. J. Lee, *Nutrients* **2020**, *12*,

38. N. Heidarzadeh-Rad, H. Gökmen-Özel, A. Kazemi, N. Almasi and K. Djafarian, *J Neurogastroenterol Motil* **2020**, *26*, 486-495.

39. M. S. Karbownik, Ł. Mokros, M. Dobielska, M. Kowalczyk and E. Kowalczyk, *Front Nutr* **2022**, *9*, 850249.

40. A. Kazemi, A. A. Noorbala, K. Azam, M. H. Eskandari and K. Djafarian, *Clin Nutr* **2019**, *38*, 522-528.

41. J. Moludi, H. Khedmatgozar, S. M. Nachvak, H. Abdollahzad, M. Moradinazar and A. Sadeghpour Tabaei, *Nutr Neurosci* **2021**, 1-10.

42. A. Perez-Cornago, A. Sanchez-Villegas, M. Bes-Rastrollo, A. Gea, P. Molero, F. Lahortiga-Ramos and M. A. Martínez-González, *J Nutr* **2016**, *146*, 1731-9.

43. A. Hadi, M. Sepandi, W. Marx, S. Moradi and K. Parastouei, *Complement Ther Med* **2019**, *47*, 102216.

44. N. Haghighat, S. Rajabi and M. Mohammadshahi, *Nutr Neurosci* **2021a**, *24*, 490-499.

45. N. Haghighat, M. Mohammadshahi, S. Shayanpour, M. H. Haghighizadeh, S. Rahmdel and M. Rajaei, *Indian J Nephrol* **2021b**, *31*, 149-156.

Table S12. Characteristics of studies investigating the efficiency of gut microbiota-based therapeutics in animal models of depression.

Study	Object	Depression model	Microbiota-based therapies	Genus	Species	Intervention method	Depression alleviation	Gut-brain axis mechanism
Rao J et al. 2021a[1]	Sprague-Dawley rats	CUMS-depression	Fecal microbiota transplantation	_	_	FMT group was administered a gavage of fecal supernatant with 2 $\times 10^9$ fecal microbiota for 14 consecutive days	Fecal microbiota transplantation improved the CUMS-induced depressive-like behavior	Fecal microbiota transplantation altered the gut microbiota imbalance, and alleviated the intestinal tract inflammation, intestinal mucosa disruption, and neuroinflammation
Rao J et al. 2021b[2]	Sprague-Dawley rats	CUMS-depression	Fecal microbiota transplantatio	_	_	For each rat, 1 ml of bacterial suspension (2 $\times 10^9$ CFU/ml) was transplanted to each of the recipient rat by gavage each day for consecutive 14 days	Treatment with fecal microbiota transplantation ameliorated depression-like behaviors	Treatment with fecal microbiota transplantation suppressed activation of glial cells and NLRP3 inflammasome in the brain
Han SK et al. 2021[3]	C57BL/6 mice	RS-depresstion FMT-RS-depression	Fecal microbiota transplantation	_	_	0.2 mL of the fecal microbiota suspension were orally gavaged in (the stomach of) mice once a day for 5 days	Fecal transplantation of vehicle-treated control or RS/CSS-treated mice into RS-exposed mice significantly mitigated RS-induced anxity- and depressive-like behaviors	Fecal transplantation treatment suppressed the NF-κB activation in the hippocampus and colon, reduced the IL-6 and corticosterone levels in the blood, and regulated gut microbiota composition
Marcondes Ávila PR et al. 2020[4]	Wistar rats	CMS-depression	Fecal microbiota transplantation	_	_	An equivalent of 3 $\times 10^8$ cells in a 100-μL solution was given to each rat for five consecutive days by gavage	FMT treatment improved depressive-related (open-field) behavior	Manipulation of the microbiota reversed the behavioral and biochemical changes induced by the CMS protocol, and the vagus nerve influenced the gut–brain axis response
Xu Z et al. 2018[5]	C57BL/6 mice	Alcohol-induced depression	Fecal microbiota transplantation	_	_	Mice in FMT group received 200 μL suspensions with a minimum dose of approximately 10^{10} bacteria at each oral gavage (from 3 male healthy volunteers)	FMT significantly decreased anxiety- and depressive-like behaviors	_
Zhang Y et al. 2019[6]	C57BL/6 mice	CUS-depression	Fecal microbiota transplantation	_	_	Antibiotic-treated mice were orally challenged with 300 μl fecal transplants (approximately 2 × 10^8 viable probiotic bacteria dissolvedin sterile PBS) by gavaging on 3 consecutive days	Transplantation of the NLRP3 KO microbiota alleviated the CUS-induced depressive-like behaviors	FMT significantly ameliorated astrocyte dysfunction in recipient mice treated with CUS via inhibition of circHIPK2 expression
Zhou H et al. 2022[7]	C57BL/6 mice	Dcf1 KO-depression	Fecal microbiota transplantation and probiotics treatment	Lactobacillus, Bifidobacterium	L. reuteri, L. murinus, B. longum	Microbial transplantation was performed at 9:00 a.m. each day for 14 days. L. murinus, L. reuteri, and B. longum were diluted using 0.9% NaCl to a density of 10^9 CFU/mL.	Depression-like behavior of KO group was relieved following transplantation with L. reuteri, L. murinus, B. longum	Lactobacillus rescued depressive symptoms by restoring GABA levels
Wu Z et al. 2021[8]	C57/B6	CSDS-depression	Fermentate of bacteria	Lactobacillus, Streptococcus	Adzuki bean sprout fermented milk generated by L. bulgaricus, S. thermophilus, L. plantarum 15953, and L. brevis J1	Administrationg of full dose of adzuki bean sprout fermented milk (high, 0.4 mL per day), half dose of adzuki bean sprout fermented milk (medium, 0.2 mL of per day), and a low dose of adzuki bean sprout fermented milk (low, 0.1 mL per day) for 10 days	GABA-enriched adzuki bean sprout fermented milk alleviated the depression-like	GABA-enriched adzuki bean sprout fermented milk treatment regulated the GABAB-cAMP-PKA-CREB) signaling pathway and increased the monoamine transmitters (5-hydroxytryptamine, norepinephrine, and dopamine) in the hippocampus of mice

Han SK et al. 2020a[9]	C57BL/6 mice	IS-depresstion EC-depresstion	Fermentate of Bifidobacteria	Bifidobacteria	Bifidobacteria-Fermented Red Ginseng and Its Constituents Ginsenoside Rd and Protopanaxatriol	10 mg/kg/day of RG; 25 mg/kg/day of RG; 50 mg/kg/day of RG; 10 mg/kg/day of fRG; 25 mg/kg/day of fRG; and 50 mg.kg/day of fRG dissolved in 1% maltose were orally gavaged once a day for 5 days	Treatment with RG and fRG significantly mitigated the stress-induced anxiety/depression-like behaviors	fRG and its constituents Rd and protopanaxatriol mitigated anxiety/depression and colitis by regulating NF-κB-mediated BDNF expression and gut dysbiosis
Ko CY et al. 2013[10]	Sprague-Dawley rats	FST-depression	Fermente of probiotics	Lactobacillus	Fermented black soybean milk by L. brevis FPA 3709	Feeding with 48-h fermented black soybean milk at a dosage of 35 mg/kg b.w. including 2.5 mg GABA/kg b.w., and a double-dosage sample group (70 mg/kg b.w. including 5.0 mg GABA/kg b.w.) for 28 days	Oral feeding of 48-h fermented product significantly reduced the duration of immobility in a dose dependent manner	The underlying mechanism for the antidepressant effect of this fermented product merits further research into the changes in the profile of monoamines, such as serotonin, dopamine, and norepinephrine, in rat brains
Warda AK et al. 2019[11]	C57BL/6 mice	ADR-159-depression	Heat-killed fermentate of bacteria	Lactobacillus	ADR-159, a heat-killed fermentate generated by L. fermentum and L. delbrueckii	ADR-159 was incorporated into standard mice chow to a final concentration of 5%, equivalent to approximately 3×10^9 cell bodies per gram of chow	ADR-159 fed animals exhibited depressive- and anxiety like behaviors	ADR-159 fed animals had significantly lower base line corticosterone levels and diturbed microbial community
Xu N et al. 2018[12]	ICR mice	Constipation-depression	Multispecies probiotics	bifidobacteria, lactobacillus, laccoccus and yeast	No data	The probiotic group was given probiotic (10 mg/kg daily by gavage), for 14 consecutive days	Administration of a probiotic ameliorated depressive behaviors	Probiotics alleviated depression through protecting neuronal health via activation of the AKT signaling pathway
Abildgaard A et al. 2021[13]	Flinders sensitive line rats Flinders resistant line rats	FSL-depression	Multi-species probiotics	Bifidobacterium, Lactococcus and Lactobacillus	9 strains: B. bifidum W23, B. lactis W51, B. lactis W52, L. acidophilus W37, L. brevis W63, L. casei W56, L. salivarius W24, Lc. lactis W19 and Lc. lactis W58	Receiving a bottle of 4.5 g (2.5×10^9 CFU/g) freeze-dried probiotics dissolved in 30 mL of tap water for 12 weeks. The treatments were administered daily between 4 and 6 pm and completely emptied by the animals during the night.	Probiotics has effects on the gut microbiota composition associated with depressive-like behaviour	_
Li N et al. 2018[14]	C57BL/6 mice	CMS-depression	Multistrain probiotics	Bifidobacterium, Lactobacillus	L. helveticus R0052, L. plantarum R1012, B. longum R0175	The bacterial solution (200 µl or 2×10^9 CFU) was administered by oral gavage daily for 4 weeks during the experimental procedure	Probiotics attenuated CMS-induced anxiety- and depressive-like behaviors	Probiotics treatment modulated the gut microbiota-inflammation-brain axis, characterized by regulated gut micrbiota, decreased hippocampal levels of proinflammatory cytokines (IFN-g and TNF-a), and direct or inflammatory-mediated inhibition of IDO1 activity
Liu QF et al. 2020[15]	ICR mice	IS-depresstion	Multi-strains probiotics	Bifidobacterium, Lactobacillus, Pediococcus	L. plantarum LP3, L. rhamnosus LR5, B. lactis BL3, B. breve BR3, P. pentosaceus PP1	Probiotic formulation (500 µL; 2×10^8 CFU/mL) was subsequently administered to mice subjected to stress conditions over a 4-week period	Probiotic administration alleviated depressive-like behaviors	Ingested probiotics altered the composition of gut microbiota and decreased corticosterone level in serum

Ding Y et al. 2021[16]	C57BL/6 mice	CRS-depression	Next-generation probiotics	Akkermansia, Lactobacillus	A. muciniphila ATCC® BAA-835™, L. plantarum CICC® 23,133	200 µl (5 × 10^8 CFU/mL) of A. muciniphila was administered via gavage for 3 weeks 200 µl (5 × 10^8 CFU/mL or 5 × 10^9 CFU/mL) of L. plantarum was administered via gavage for 3 weeks	A. muciniphila and high-dose Lactobacillus plantarum treatments ameliorated CRS-induced depressive-likebehaviors in mice	A. muciniphila treatment regulated abnormal variations in hormone (corticosterone), neurotransmitter (dopamine and serotonin), and BDNF expression levels in CRS-induced mice, and regulated gut microbiota
Abildgaard A et al 2017a[17]	Sprague-Dawley rats	Fed with a control or high-fat diet	Probiotics	Lactobacillus, Bifidobacterium	8 bacterial strains: B. bifidum W23, B. lactis W52, L. acidophilus W37, L. brevis W63, L. casei W56, L. salivarius W24, Lc. Lactis W19, Lc. Lactis W58	Each cage received a bottle containing 4.5 g(2.5 × 10^9 CFU/g) of freeze-dried powder dissolved in 30 mL of tap water,the bottles were administered daily between four and six pm for 5 weeks	Probiotic treatment markedly reduced depressive-like behaviour in the forced swim test	Probiotic treatment regulated the HPA axis, immune system and microbial tryptophan metabolism
Abildgaard A et al. 2017b[18]	Flinders Sensitive Line rats	HFD-depression	Probiotics	Lactobacillus, Bifidobacterium	8 bacterial strains: B. bifidum W23, B. lactis W52, L. acidophilus W37, L. brevis W63, L. casei W56, L. salivarius W24, Lc. Lactis W19, Lc. Lactis W58	Each cage received a bottle containing 4.5 g(2.5 × 10^9 CFU/g) of freeze-dried powder dissolved in 30 mL of tap water,the bottles were administered daily between four and six pm for 5 weeks	Probiotic treatment protects against the pro-depressant-like effect of high-fat diet in Flinders Sensitive Line rats	Probiotic treatment regualted cerebral T cell populations demonstrating that lymphocyte-brain interactions as a promising future research area in the field of psychoneuroimmunology
Abildgaard A et al. 2019[19]	Sprague-Dawley rats	_	Probiotics	Bifidobacterium, Lactococcus, Lactobacillus	B. bifidum W23, B. lactis W51, B. lactis W52, L. acidophilus W37, L. brevis W63, L. casei W56, L.salivarius W24, Lc. Lactis W19 and Lc. lactis W58	Each probiotics cage of two rats was administered a bottle of 4.5 g (2.5 × 10^9 CFU/g) freeze-dried PRO in a carrier matrix (maize starch, maltodextrins and vegetable protein) dissolved in 30 mL of tap water for 8 weeks	Probiotics had antidepressant-like effect	The cohabiting microbiota and the faecal abundance of probiotics may modulate the antidepressant-like effect of probiotics in rats
Agusti A et al. 2018[20]	C57BL/6 mice	HFD-depression	Probiotics	Bifidobacterium	B. pseudocatenulatum CECT 7765	Receiving a daily dose of 1 × 10^9 CFU B. pseudocatenulatum CECT 7765 by gavage for 14 weeks	B. pseudocatenulatum CECT 7765 ameliorated depressive-like behaviors	B. pseudocatenulatum CECT 7765 regulated the endocrine and immune mediators of the gut–brain axis.
Arseneault-Breárd J et al. 2012[21]	Sprague–Dawley rats	Post-myocardial infarction depression	Probiotics	Lactobacillus, Bifidobacterium	L. helveticus R0052, B. longum R0175	For 7 d before MI and between the 7th post-MI day and euthanasia, half the MI and sham rats were given one billion live bacterial cells of L. helveticus R0052 and B. longum R0175 per d dissolved in water	L. helveticus R0052 and B. longum R0175 combination interfered with the development of post-MI depressive behaviour	The beneficial impact of probiotics combination also includes the maintenance of intestinal barrier integrity, which may contribute to the inflammatory state observed after MI
Arslanova A et al. 2021[22]	mice	Antibiotic-depression	Probiotics	Lactobacillus	Two strains: L. rhamnosus B-8238, L. plantarum 8PA3	Receiving 1 mL drinking water contained 2 × 10^5 CFU/mL of a lactobacilli mixture (1:1) once a day for 14 days	Lactobacillus treatments decrease the anxiety level , increase the muscle endurance and motor coordination, and improve cognitive functions of mice	Lactobacillus treatments decreased the inflammation and oxidative stress, and improved microbiota content

Aygun H et al. 2022[23]	WAG/Rij rats	Depressive-like behavior in WAG/Rij rat	Probiotics	Lactobacillus, Bifidobacterium, Streptococcus	VSL#3 contains 4 strains of Lactobacillus (L. plantarum, L. acidophilus, L. delbrueckii subsp. bulgaricus and L. casei), 3 strains of Bifidobacterium (B. longum, B. breve, B. infantis) and Streptococcus salivarius subsp. Thermophilus	VSL#3 at the doses of 12.86 bn living bacteria/kg/day in 0.5 ml for 30 day by gavage	VSL#3 supplementation exhibited anxiolytic and anti-depressive effect	VSL#3 supplement also increased the NGF immunoreactivity while decreasing IL-6, TNF-a and NO levels in WAG/Rij rat brain
Barros-Santos T et al. 2020[24]	Swiss mice		Probiotics	Lactobacillus	L. plantarum 286 (Lp 286), L. plantarum 81 (Lp 81)	Mice were treated daily for 30 days with single doses of 0.1 ml vehicle solution supplemented with Lp 286 (10^9/0.1 ml CFU) or Lp 81 (10^9/0.1 ml CFU)	The L.plantarum 286 strain exerted anti-depressant- effects under our experimental conditions	_
Bharwani A et al. 2017[25]	C57BL/6 mice	CSDS-depresstion	Probiotics	Lactobacillus	L. rhamnosus JB-1	Gavageing with 200 μl (1.67×10^9 CFU) of L. rhamnosus (JB-1) ™ for 28 days	JB-1 treatment partially corrected the adverse effects of stress on social preference, exploration, and anxiety-like behaviours	JB-1 treatment promoted systemic changes in the immunoregulatory phenotype and influenced the effects of chronic stress on host immunity
Birmann PT et al. 2021[26]	Swiss mice	LPS-depression	Probiotics	Komagataella	K. pastoris KM71H	Mice were treated with K. pastoris KM71H in the concentration of 8 log UFC/g/per animal by intragastric route (i.g.) for a period of 14 consecutive days for CRS stress and 7 days for LPS stress	K. pastoris KM71H prevented depression-like behavior induced by stress	K. pastoris KM71H modulated of the permeability of the blood-brain barrier, prevented an inflammatory response and oxidative stress, and decreased the plasma corticosterone levels
Bravo JA et al. 2011[27]	BALB/c mice	Healthy status	Probiotics	Lactobacillus	L. rhamnosus (JB-1)	Animals were orally gavaged with broth with L. rhamnosus (JB-1) 10^9 CFU daily between 8:00 and 9:00 for a period of 28 continuous days	L. rhamnosus (JB-1) reduced stress-induced anxiety- and depression-related behavior	L. rhamnosus (JB-1) modulated the GABAergic system in mice and therefore may have beneficial effects in the treatment of depression and anxiety
Chen P et al. 2019[28]	BALB/c mice	UCMS-depresstion	Probiotics	Lactobacillus	Three strains: L. reuteri, L. murinus, L. johnsonii	Gavaging with 200 μl bacterial suspension of L. reuteri (3×10^9 CFU/mL in pre-reduced PBS) for another 30 days	Lactobacillus reuteri has a significant therapeutic effect on depression	Oral administration of Lactobacillus reuteri increased brain serotonin levels and serotonin-positive cells in the dorsal raphe nucleus, and modulated microbiota
Chen T et al. 2021[29]	C57BL/6N mice	CRS-depression FMT CRS-depression	Probiotics	Akkermansia	A. muciniphila	Orally administratiion of another 100 μl of A. muciniphila containing 1×10^8 bacteria for 14 days	A. muciniphila supplementation alleviated depression-like behaviors	A. muciniphila supplementation prevented mucosal barrier defects and aggravation of colitis, and modified the gut microbiota
Chen X et al. 2022[30]	Sprague-Dawley rats	Lead exposure-depression	Probiotics	Lactobacillus, Bifidobacterium	_	Probiotics (6 billion live bacteria/2 g) were administered to the rats by gavage 5 times a week, at least 1.2×10^9 CFU combined strains were given to each rat, and doses as high as 4.8×101^0 CFU were administered	Probiotic intervention relieved the depression-like behavior of lead-exposed rats	Probiotic intervention altered the gut microbiome, while the fecal SCFAs could be a possible adjuvant therapy of depression

Chen Y et al. 2021b[31]	Sprague-Dawley rats	CUMS-depression	Probiotics	Rhizopus, Bacillus	Semen Sojae Praeparatum, a fermented food by R. chinensis 12 and Bacillus sp. DU-106	The rats were given a daily dose of 0.97 g/kg Semen Sojae Praeparatum (dissolved in 10 mL of normal saline) for 4 weeks	Semen Sojae Praeparatum fermented by R. chinensis 12 and Bacillus sp. DU-106 could ameliorate depressive behaviors.	Semen Sojae Praeparatum regulated the metabolite levels in the serum and hippocampus tissue, reversed the cell morphology and mitochondrial function of hippocampal neurons through improving the imbalance in gut microbiota and inhibiting the excessive SCFAs accumulation
Chevalier G et al. 2020[32]	C57BL/6J mice	UCMS-depression	Probiotics	Lactobacillus	L. plantarum^{WJL}	Supplementing with oral feeding 5 days a week with 2×10^{8} CFU of L. plantarum^{WJL} diluted in 200 μl of PBS	Complementation with L. plantarum^{WJL} normalized depression-like behaviors	Complementation with L. plantarum^{WJL} improved lipid metabolism and the generation of eCBs, leading to increased signaling in the eCB system and adult neurogenesis in the hippocampus
Choi J et al. 2019[33]	C57BL6/J mice	CRS-depression	Probiotics	Lactobacillus	Extracellular vesicles (EV) from L. plantarum	L-EVs at a dose of 0.1 μg/kg were intraperitoneally injected into a mouse at a volume of 100 μl 30 min prior to restraint treatment for 14 days； for the post-stress period, L-EVs were intraperitoneally injected at a volume of 100 μl containing increasingly higher doses; 0.1 μg/kg for the first 5 days, 0.18 μg/kg for the following 2 days, and 0.27 μg/kg for the final 7 days	L-EV treatment in CRST-treated mice rescued stress-induced depressive-like behaviors	L-EV treatment in CRST-treated mice rescued the reduced expression of Bdnf
Choi J et al. 2022[34]	C57BL6/J mice	CRS-depression	Probiotics	Lactobacillus, Bacillus, Akkermansia	Extracellular vesicles (EV) from L. plantarum, Bacillus subtilis, and A. muciniphila	Lac-EV, Bac-EV, or Akk-EV were administered, each with 6 μg in 100 μl of injection volume, for 14 days	Injection of EV isolated from culture media of L. plantarum, Bacillus subtilis and A. muciniphila are sufcient to ameliorate stress-induced depressive-like behavior	Injection of EV from the three selected probiotics restored the expression of MeCP2, Sirt1, and/or neurotrophic factors in the hippocampus
Daugé V et al. 2020[35]	Fischer/Long Evans Rats	MD-depression	Probiotics	Lactobacillus, Bifidobacterium, Lactococcus, Streptococcus	L. helveticus LA 102, B. longum LA 101, Lc. lactis LA 103, and S. thermophilus LA 104	Rats received 0.5 mL of the probiotics (1 ×109 CFU) by gavage with probes 5 days a week for 5 weeks for Fischer rats and 9 weeks for Long Evans rats (until euthanasia)	A probiotic mixture induces anxiolytic- and antidepressive-Like effects	Probiotic mixture treatment changed the levels of certain metabolites, such as 21-deoxycortisol, and changed brain monoamines
Desbonnet L et al. 2010[36]	Sprague-Dawley rats	MS-depression	Probiotics	Bifidobacterium	B. infantis 35624	B. infantis 35624 was administered by dissolving a powdered preparation, containing a dose of 1×10^{10} live bacterial cells, in 100 ml of the rats drinking water every morning from P50 to the day of sacrifice	B. infantis 35624 treatment alleviated depressive-like behaviors	Probiotic treatment resulted in normalization of the immune response and restoration of basal NA concentrations in the brainstem.
Dhaliwal J et al. 2018[37]	Swiss albino LACA mice	CUMS-depression SD-depression	Probiotics	Lactobacillus	L. plantarum MTCC 9510	L. plantarum MTCC 9510 (2×10^{10} CFU CFU per mice) was supplemented to male Swiss albino mice either subjected to chronic unpredictable mild stress (28 days) or sleep deprivation stress (21 days)	L. plantarum MTCC 9510 supplementation prevented stress-induced behavioural despair (depression, anxiety, learning and memory, stereotypic behaviour)	L. plantarum MTCC 9510 supplementation prevented the oxidative stress and inflammatory response in brain and serum, and prevented intestinal permeability and selected gut microbial aberrations

Gao K et al. 2022[38]	BALB/c mice	CUMS-depression	Probiotics	Lactococcus	Lc. lactis strain WHH2078	Orally administrated with 200 μL pre-warmed WHH2078 preparation (1×10^9 CFU/mL) for 5 consecutive weeks	Lc. lactisstrain WHH2078 alleviated depressive and anxiety-like behaviors	Lc. lactisstrain WHH2078 improved the 5-HT metabolism along the GBA and modulation of the gut microbiome composition
Gu F et al. 2020[39]	Sprague-Dawley rats	CUMS-depression	Probiotics	Lactobacillus	L. casei	Administration with to L. casei (8×10^8 CFU/kg/day) for 4 weeks from 4th to 7th week of CUMS	L. casei treatment relieved the depressive-like behaviors of rats induced by CUMS	L. casei treatment up-regulated the expression levels of monoamines 5-HT, DA and NE, activated the BDNF-TrkB signaling, inhibited the phosphorylation of EKR1/2 and P38 in frontal cortex, and regulated the gut microbiota composition
Guida F et al. 2018[40]	C57/bl6 mice	Antibiotic-depresstion	Probiotics	Lactobacillus	L. casei DG	Oral gavage with the probiotic (L. casei DG, 10^9 cells in saline, 100 μl) up to 7 days.	L. casei treatment relieved the depressive-like behaviors induced by antibiotics	Administration of L. casei adjusted gut inflammation, nomalized the hippocampal BDNF-TrkB signaling, improved hippocampal and cortical electrophysiological neuronal activity, and reduced microglia and astrocyte activation
Guo Y et al. 2019[41]	ICR mice	CRS-depression	Probiotics	Bifidobacterium	B. adolescentis	Receiving 0.25×10^9 CFU/kg B. adolescentis by gavage for 21 days	B. adolescentis treatment prevented the development of anxiety- and depressive-like behaviors caused by CRS	B. adolescentis treatment are related to reducing inflammatory cytokines and rebalancing the gut microbiota
Han SK et al. 2019[42]	C57BL/6 mice	IS-depression	Probiotics	Lactobacillus, Bifidobacterium	L. mucosae NK41, B. longum NK46	1×10^9 CFU of NK41/mouse/day; 1×10^9 CFU of NK46/mouse/day; 1×10^9 CFU of NK41 and NK46 mixure (1:1) mix/mouse/day	Oral administration of NK41, NK46, or their mixure synergistically alleviated immobilization stress-induced anxiety- and depressive-like behaviors in mice	Oral administration of NK41, NK46, or their mixure inhibited gut inflammation through the inhibition of gut bacterial LPS production, and prevented gut dysbiosis
Han SK et al. 2020b[43]	C57BL/6 mice	EC-depresstion	Probiotics	Lactobacillu, Bifidobacterium	L. reuteri NK33, B. adolescentis NK98	1×10^9 CFU/mouse/day of NK33; 1×10^9 CFU/mouse/day of NK98; EN1:1, 1×10^9 CFU/mouse/day of the NK33 and NK98 (1:1)/(4:1)/(9:1) mixture were orally gavaged once a day for 5 days from 24 h after the final K1 treatment	Oral gavage of NK33 and/or NK98 alleviated Escherichia coli K1-induced depression-like behaviors in mice	Oral gavage of NK33 and/or NK98 shifted Escherichia coli K1-induced gut microbiota alteration in mice
Hao W et al. 2021[44]	C57BL/6 mice	Antibiotic-depression	Probiotics	Bifidobacterium, Lactococcus, Lactobacillus and Streptococcus	Sixteen strains: B. longum, L. acidophilus, B. bifidum, B. breve, B. lactis, L. brevis, L. bulgaricus, L. casei, L. helveticus, L. plantarum, L. reuteri, L. rhamnosus, L. salivarius, Lc. lactis, S. thermophilus, and B. infantis	Receiving probiotics solution (0.15 ml/d) for 14 consecutive days	Probiotics treatment mitigated antibiotic-Induced anxity- and depressive-like behaviors	Probiotics treatment modulated the gut microbiota,corrected excessive LPS release, and inhibited the immoderate activation of the NLRP3 inflammasome in the colon

Hao Z et al. 2019[45]	Sprague-Dawley rats	CUMS-depression	Probiotics	Faecalibacterium	F. prausnitzii (ATCC 27766)	Rats were fed at the same time each day by oral gavaged with 200 μL of resuspended F. prausnitzii, 1 $\times 10^9$ CFU (from the eighth week to the eleventh week) daily.	Administration of F. prausnitzii had preventive and therapeutic effects on CUMS-induced depression-like and anxiety-like behavior	F. prausnitzii administration led to higher levels of SCFAs in the cecum and higher levels of cytokines interleukin-10 (IL-10) in the plasma, prevented the effects on corticosterone, C-reaction protein and cytokines interleukin-6 (IL-6) release induced by CUMS
Huang F et al. 2021[46]	C57BL/6 mice	Ovariectomy-depression	Probiotics	Prevotella	P. histicola DSM19854	Receiving P. histicola (10 ml/kg) per second day for 12 weeks	P. histicola alleviated depressive behaviors caused by estrogen deficiency	P. histicola regulated disorder microbiota to attenuate central inflammation, which might be involved in TLR4/Myd88/JNK MAPK pathway (Figure 9) and further upregulate BDNF expression for hippocampal neurogenesis
Huang YY et al. 2022[47]	C57BL/6N mice	DSS-depression	Probiotics	Lactobacillus	L. plantarum DMDL 9010 (LP9010)	Mice administered orally with 0.2 mL/10 g weight per day LP9010 at a dose of 10^7 CFU/mL and 10^9 CFU/mL for 7 days	LP9010 intake lightened depression-like behavior	LP9010 promoted anti-inflammatory cytokines, reduced proinflammatory cytokines, enhanced SCFAs production, and reorganized the gut microbiome
Jang HM et al. 2019[48]	C57BL/6 mice	IS-depresstion	Probiotics	Lactobacillus, Bifidobacterium	L. reuteri NK33, B. adolescentis NK98	Orally gavaged NK3 or NK49 at a dose of 1×10^9 CFU/mouse/day for 5 days in IS-treated mice	Treatment with NK33 and/or NK98, which were orally gavaged in mice before or after IS treatment, significantly suppressed the occurrence and development of anxiety/depression	NK33 and NK98 synergistically regulated of gut immune responses (inhibited NF-κB activation, attenuated colitis and hippocampal inflammation) and microbiota composition
Kambe J et al. 2020[49]	C57BL/6 J mice	Healthy status	Probiotics	Enterococcus	Heat-killed E. fecalis strain EC-12 (EC-12)	The EC-12 group was fed on AIN-93 M diet with heat-killed EC-12 at a concentration of 0.125 % for 4 weeks	EC-12 supplementation reduced anxiety- and depressive-like behaviors	EC-12 supplementation regulated the gut microbiota
Karen C et al. 2021[50]	Wistar rats	MS-depression	Probiotics	Lactobacillus	L. paracasei HT6	Supplementing with L. paracasei HT6 (per orally, p.o. by oral gavage; from PND-2 to 16)	L. paracasei supplementation prevented early life stress-induced anxiety and depressive-Like behavior	L. supplementation potentially mediated stress hormones, neurotransmitters, and expression of miRNAs, glutamate receptors, and the microbiota-gut-brain axis
Kim JK et al. 2020[51]	C57BL/6J mice	Escherichia coli K1-depression	Probiotics	Lactobacillus	L. mucosae NK41	Mice were orally gavaged with the NK41 (1×10^9 CFU/mouse/day) once a day for 5 days from 24 h after treatment with K1 suspension	NK41 treatment reduced K1-induced cognitive decline and anxiety/depression	The superiority of anti-inflammatory bacteria such as L. mucosae can alleviate psychiatric disorders with the attenuation of altered microbiota
Kochalska K et al. 2020[52]	Wistar rats	CUMS-depression	Probiotics	Lactobacillus	L. rhamnosus JB-1	The JB-1 group was fed a microbiotic diet with LR-JB1™ daily for 4 weeks	Dietary supplement of LR-JB1™ resulted in a reduction of stress-induced behavior in a rat model of depressive-like disorder	A microbiotic diet with LR-JB1™ brought improvements in neurochemical balance in the course of depressive-like disorder
Kosuge A et al. 2021[53]	C57BL/6J mice	CSDS-depresstion	Probiotics	Bifidobacterium	B. breve M-16V	M-16V-treated groups were fed the AIN-93G diet which containing 5.0×10^9 nonviable cells/0.5 g	Heat-sterilized B. breve M-16V supplementation significantly prevented depressive-like behavior (social interaction impairment)	Heat-sterilized B. breve M-16V supplementation suppressed CSDS-induced neuroinflammation and modulated the gut microbiota composition

Li Q et al. 2021[54]	Sprague-Dawley rats	CUMS-depression	Probiotics	Bifidobacterium, Lactobacillus	L. helveticus, L. rhamnosus, L. casei, B. longum	Giving 2 ml of probiotics at a concentration of 4 × 10^8 CFU/kg once daily for 4 weeks	Probiotics alleviated CUMS-induced depressive-like behaviors	Probiotics treatment remodeled intestinal flora, increased the monoamine neurotransmitters (norepinephrine and 5-hydroxytryptamine), and inhibited hypothalamic–pituitary–adrenal neuroendocrine system (ACTH and corticosterone)
Liang S et al. 2015[55]	Sprague–Dawley rats	CRS-depression	Probiotics	Lactobacillus	L. helveticus NS8	The strain was resuspended in drinking water at a concentration of 10^9 CFU/ml, and was administrated for 26 days until the termination of the experiment	Treatment with probiotic L. helveticus NS8 had anxiolytic and antidepressant effects, promoted cognition	Treatment with probiotic L. helveticus NS8 decreased plasma CORT and ACTH levels, modulated pro-inflammatory and anti-inflammatory balance, and restored 5-HT, NE, and BDNF content in the hippocampus
Liao JF et al. 2019[56]	C57BL/6J mice	MS-depression	Probiotics	Lactobacillus	L. paracasei PS23 (PS23)	The live (live cells of PS23 at 1×10^9 CFU/mouse/day) and heat-killed cells (heat-killed cells of PS23 at 1×10^9 cells/mouse/day) were administered by oral gavage for 4 weeks starting from PD 29	L. paracasei PS23 alleviated stress, anxiety, and depressive traits in maternal separated mice	PS23 modulated stress responses via immunomodulatory effects along the dopaminergic systems of the brain, illustrating the potential of PS23 as a psychobiotic
Lim EY et al. 2021[57]	Sprague Dawley rats	Ovariectomy-depression	Probiotics	Lactobacillus	L. intestinalis YT2	Treatment (10^9 CFU/ml) started one week after surgery and lasted for 18 weeks	Treatment with L. intestinalis YT2 significantly alleviated menopausal symptoms lincluding depression-like behaviour	Administration of L. intestinalis YT2 restored the intestinal microbial composition, promoted gut barrier integrity by increasing the mRNA levels of tight junction-related markers
Liu Y et al. 2020[58]	BALB/c mice	Healthy status	Probiotics	Lactobacillus	L. rhamnosus JB-1	The bacteria were delivered in drinking water at 1 × 10^9 CFU per day for 28 days prior to behavioural testing and continued throughout the testing period for a total of 39 days treatment	L. rhamnosus JB-1 treatment reduced depressive- and anxiety-like behavior	CD4+CD25+ cells, most likely regulatory T cells, are both necessary and sufficient for L. rhamnosus JB-1 induced antidepressant and anxiolytic-like effects
Liu YW et al. 2015[59]	C57BL/6J mice	MS-depression	Probiotics	Lactobacillus	L. plantarum PS128	Giving saline re-suspended PS128 daily (10^9 CFU/ mouse/day) by gavage for 4 weeks from PND 29 to 8 weeks old	Chronic ingestion of PS128 could ameliorate anxiety- and depression-like behaviors	PS128 reduced inflammatory cytokines and increased anti-inflammatory cytokines in the serum, and increased the dopamine level in the prefrontal cortex
Maehata H et al. 2019[60]	C57BL/6J mice	sCSDS-depression	Probiotics	Lactobacillus	Heat-killed L. helveticus MCC1848	Heat-killed MCC1848 was added to AIN93G at 3.3 × 10^8 organisms/g, resulting in the consumption of approximately 1.0 × 10^9 organisms/day for 24 days	MCC1848 improved anxiety- or depressive-like behaviors in sCSDS mice	MCC1848 ameliorated sCSDS-induced gene expression alterations in signal transduction or nervous system development
McVey Neufeld KA et al. 2018[61]	BALB/c mice	Healthy status	Probiotics	Lactobacillus	L. rhamnosus JB-1	JB-1 was delivered via the drinking water at a dose of 10^9 CFU/day for 28 days	Feeding L. rhamnosus JB-1 to BALB/c mice reduced depressive-like behavior	Feeding L. rhamnosus JB-1 to BALB/c mice attenuated plasma corticosterone and hastens recovery
Moya-Pérez A et al. 2017[62]	C57BL/6J mice	MS-depression	Probiotics	Bifidobacterium	B. pseudocatenulatum CECT 7765	Receiving a daily oral dose of 1 × 10^8 CFU B. pseudocatenulatum CECT 7765 from P2 until P21	B. pseudocatenulatum CECT 7765 treatment reduced MS-induced anxiety- and depressive-like behaviors in adulthood	B. pseudocatenulatum CECT 7765 treatment modulated the consequences of chronic stress on the HPA response via modulation of the intestinal neurotransmitter, cytokine network, and gut microbiota composition

Murray E et al. 2019[63]	CD-1 mice	LPS-depression	Probiotics	Lactobacillus	L. lactis, L. cremoris, L. diacetylactis, L. acidophilus	5 g of dry kefir probiotic culture (3.0 $\times 10^9$ CFU/g) into 1L of skim milk , mice had *ad libitum* access to kefir	Probiotic consumption during puberty protected against LPS-induced depression- and anxiety-like behaviors in adulthood	Probiotic consumption during puberty mitigated inflammation, and prevented LPS-induced changes to the gut microbiome
Natale NR et al. 2021[64]	Long-Evans rats	CUS-depression	Probiotics	Lactobacillus	L. helveticus R0052, L. rhamnosus R0011	The PB-infused water was prepared by rehydrating 2.87 g of powder (mixed with maltodextrin and milk powder) in 75 mL of distilled water for a final concentration of 10^9 CFU/ml for 2 weeks	Probiotics prevented the anxiety and depressive behavior	Probiotics reduced microglia immunoreactivity in the basolateral amygdala, possibly indicating a neuroprotective effect of PB supplements in this rodent model
Partrick KA et al. 2021[65]	Syrian hamsters (Mesocricetus auratus)	CSDS-depression	Probiotics	Lactobacillus, Bifidobacterium	L. helveticus R0052, B. longum R0175	Probiotic at a low dose of 10^9 CUF per day, or probiotic high dose of 10^{10} CUF per day for 14 days	Surprisingly, probiotic administration at the low dose significantly increased social avoidance and decreased social interaction	Probiotic administration altered gut microbial composition and promoted an anti-inflammatory profile
Patterson E et al. 2019[66]	C57BL/6J mice	HFD-depression	Probiotics	Lactobacillus	L. brevis DPC6108, L. brevis DSM32386	Water containing either L. brevis DPC6108 (1 $\times 10^{10}$ CFU/day) or L. brevis DSM32386 (1 $\times 10^{10}$ CFU/day) was supplied to the mice in these groups for the 12 week intervention period and bottles were replaced daily	Probiotics treatment improved depression-like behaviour	Probiotics treatment improved metabolic syndrome in mice (reduced the accumulation of mesenteric adipose tissue, increased insulin secretion, improved plasma cholesterol clearance and reduced basal corticosterone)
Qiu X et al. 2021[67]	C57BL/6J mice	LPS-depression	Probiotics	Lactobacillus	L. delbrueckii	Giving 10^9 CFU Lac intragastrically daily for 7 days	L. delbrueckii treatment effectively inhibited the occurrence of depressive-like behavior	L. delbrueckii treatment inhibited intestinal inflammation and subsequent neuroinflammation (through inbibiting toll-like receptor 4 (TLR4) signaling), and microbiota dysbiosis
Ramalho JB et al. 2019[68]	C57BL/6 mice	Healthy status	Probiotics	Lactococcus	Lc. lactis subsp. cremoris LL95	Female C57BL/6 mice received LL95 orally at a dose of 10^9 UFC/day for 28 days	Oral supplementation of L. lactis LL95 improved depressive- and anxiety-like behaviour	Oral supplementation of L. lactis LL95 improved the antioxidant parameters in the hippocampus, and increased the feacel contents of lactic acid bacteria
Ramalho JB et al. 2022[69]	C57BL/6 mice	LPS-depression	Probiotics	Lactococcus	Lc. lactis subsp. cremoris LL95	Oral administration of LL95 for one week with a daily oral dose of 1 $\times 10^9$ CFU/100 μL	LL95 intervention improved LPS-induced depression-like behaviors in mice	LL95 intervention modulated the oxidative status and pro-inflammatory cytokine expression in the hippocampus and alteration in the LAB content of the gut microbiota
Sandes S et al. 2020[70]	C57BL/6J mice	CRS-depression	Probiotics	Weissella	W. paramesenteroides WpK4	Mice were pretreated, receiving 10^8 CFU of W. paramesenteroides WpK4 during 10 days, followed by 21 days of challenge, being restrained daily	Bacterial consumption was associated with a reduced anxiety-like and depressive-like behaviors	W. paramesenteroides WpK4 exerted their beneficial roles in gut-brain axis through their immunomodulatory effects with consequences in several metabolic pathways related to intestinal permeability and hippocampal physiology
Savignac HM et al. 2014[71]	BALB/c mice	_	Probiotics	Bifidobacterium	B. longum 1714, B. breve 1205	Bacteria were daily reconstituted in sterile phosphate buffered saline (PBS) to a final concentration of 1 $\times 10^9$ CFU/mL ingested by mice for 6 weeks	Both Bifidobacteria reduced anxiety and B. longum 1714 induced antidepressant-like behavior	_
Silva LC et al. 2021[72]	Swiss Webster mice	Light-dark box and tail suspension test	Probiotics	Saccharomyces, Lactobacillus	S.cerevisiae var boulardii 17, L. paracasei DTA 81, S. cerevisiae S-04	Daily treated with probiotic-containing functional wheat beer (PWB) or probiotic-containing functional sour beer (PSB) (100 μL) for 7 days	S. boulardii 17 and L. paracasei DTA 81 withstood at sufficient doses to promote antidepressant effects in the mice group	_

Soltanmoradi H et al. 2021[73]	BALB/c mice		Probiotics	Lactobacillus	L. rhamnosus GG, and kefir, a probiotic supplement	_	Kefir, L. rhamnosus GG, and the investigated probiotic supplement have antidepressant-like properties	_
Sovijit WN et al. 2019[74]	C57BL/6J mice	Ovariectomy-depression	Probiotics	Lactobacillus	L. reuteri	Feeding with food pellets that were pulverized in a blender and kneaded with L. reuteri (2 billion CFU/mouse/day) at 10 weeks of age, and lasting for 2 weeks	L. reuteri supplementation improves depressive behaviors in OVX mice	Supplementation of L. reuteri upregulated hippocampal brain-derived neurotrophic factor (BDNF) gene expression
Stenmana LK et al. 2020[75]	Swiss mice	CRS-depression	Probiotics	Bifidobacterium, Lactobacillus	12 candidate probiotics: B. longum BG0014, B. longum ssp. infantis Bl11471, B. animalis BL0005, B. animalis ssp. lactis 420, L. paracasei Lpc-37, L. salivarius Ls-33, L. plantarum LP12418, L. plantarum LP12151, L. plantarum LP12407, L. acidophilus LA11873, L. rhamnosus LX11881, L. helveticus LH0138	Mice were administered a daily oral gavage containing 1×10^9 CFU of selected candidate probiotic solution for one week prior to and for three weeks during daily chronic restraint stress.	Of the twelve candidate probiotics, L. paracasei Lpc-37, L. plantarum LP12407, L. plantarum LP12418 and L. plantarum LP12151 prevented stress-associated anxiety and depression-related behaviours	Each of these strains had a unique profile in terms of mechanistic biomarkers related to the HPA axis and prefrontal cortex GABA receptor expression
Sun J et al. 2018[76]	C57BL/6 mice	CUMS-depression	Probiotics	Clostridium	C. butyricum WZMC1018	The bacterial solution was prepared every day in sterile milk and treated orally at a concentration of 5×10^8 CFU/0.5 mL/day/mice for 28 consecutive days.	C. butyricum WZMC1018 treatment effectively improved depressive-like behavior	C. butyricum WZMC1018 treatment stimulated the GLP-1 secretion and increased the 5-HT and BDNF through the gut-brain axis
Sun X et al. 2021[77]	C57BL/6 mice	CRS-depression	Probiotics	Lactobacillus	L. plantarum WLPL04	The final concentration of the L. plantarum WLPL04 in drinking water was 10^9 CFU/mL for 28 days	L. plantarum WLPL04 treatment alleviated CRS-induced anxiety/depressive-like behaviors and cognitive deficits	L. plantarum WLPL04 treatment reversed the abnormal change in intestinal microbiota, and alleviated the reduced levels of 5-HT, BDNF, and TrkB induced by CRS in mice
Sun Y et al. 2019[78]	Kunming mice	CUMS-depression	Probiotics	Lactobacillus	L. kefiranofaciens ZW3	Treated with L. kefiranofaciens ZW3 at different doses (10^7 CFU, 10^8 CFU, 10^9 CFU)//mouse/day for 6 weeks	Supplementation with Lactobacillus kefiranofaciens ZW3 improved depressive-like behavior	L. kefiranofaciens ZW3 regulated disorder of tryptophan metabolism, protected the HPA axis, inhibited inflammation, and reshaped the structure of the gut microbiota caused by CUMS
Takahashi K et al. 2019[79]	ddY mice	DSS-depression	Probiotics	Enterococcus	E. faecalis 2001 (EF-2001)	EF-2001 was administered orally (250mg/kg per os [p.o.]) from 14 days before the beginning of DSS	EF-2001 attenuated IBD-like symptoms and depressive-like behavior in DSS-treated mice	EF-2001 decreased rectal and hippocampal inflammatory cytokines and facilitated the NFκB p65/XIAP pathway in the hippocampus

						administration until the day prior to the last DSS treatment for 20 days		
Takahashi K et al. 2022[80]	ddY mice	Olfactory bulbectomized-depression	Probiotics	Enterococcus	E. faecalis 2001 (EF-2001)	EF-2001 (250 mg/kg) was dissolved in drinking water and administered orally (per os [p.o.]) once a day in a volume of 0.1 mL/10 g mouse body weight using a 1 mL syringe with an oral probe, from 6 days before the OBX operation for 28 days.	EF-2001 administration prevented depressive-like behaviors	EF-2001 administration regulated prefrontal cortical myelination via the enhancement of CREB/BDNF and NFκB p65/LIF/STAT3 pathways
Tian P et al. 2019a[81]	C57BL/6J mice	CUMS-depression	Probiotics	Bifidobacterium	B. longum subspecies infantis strain CCFM687	Lyophilized bacteria powder was re-suspended in 10% skimmed milk solution, and administered at a dose of 10^9 CFU/mL viable bacteria for 6 weeks	B. longum subsp. infantis strain CCFM687 showed a good anti-depressive effect	B. longum subsp. infantis strain CCFM687 increased the 5-hydroxytryptamine, serotonin and BDNF, alleviated the hyperactivity of the hypothalamic–pituitary–adrenal (HPA) axis response and accordingly reversed the peripheral inflammation status, and reshaped the gut microbiome
Tian P et al. 2019b[82]	C57BL/6J mice	CUMS-depression	Probiotics	Bifidobacterium	B. longum subsp. infantis E41, B. breve M2CF22M7	Lactic acid bacteria treatment group was gavaged at a dose of 10^9 CFU/mL body weight daily for 5 weeks	Administration of several Lactic acid bacteria strains alleviated depressive behaviors of mice	Lactic acid bacteria strains alleviated depression possibly via a 5-HTP-dependent mechanism, gut microbiota structure modulation
Tian P et al. 2020[83]	C57BL/6J mice	CUMS-depression	Probiotics	Bifidobacterium	B. breve CCFM1025	The CCFM1025 treatment group was gavaged at a volume of 0.1 ml/10g (10^9 CFU/mL) body weight daily for 6 weeks	CCFM1025 treatment significantly reduced depression- and anxiety-like behaviors	CCFM1025 treatment significantly alleviated hyperactive hypothalamic-pituitary-adrenal response, as well as inflammation, down-regulated the pCREB-c-Fos pathway, increased BDNF, SCFA and 5-HTP, and restored gut microbial abnormalities
Tian P et al. 2021a[84]	C57BL/6J mice	UCMS-depression	Probiotics	Lactobacillus, Bifidobacterium, Pediococcus	30 strains: B. adolescentis, 3 strains of B. breve, 4 strains of B. bifidum, 2 strains of B. longum subsp. Infantis, 5 strains of B. longum subsp. Longum, 4 strains of B. longum, 3 strains of L. fermentum, 2 strains of L. helveticus, 3 strains of L. plantarum, 2 strains of L. rhamnosus, Pediococcus acidilactici	Giving viable bacteria (10^9 CFU/day) by oral gavage via 10% skim milk for 6 weeks	16 strains show anti-depression and anti-anxiety like effect in at least three behavioral tests	Intestinal 5-HTP supplementary on the biosynthesis of brain serotonin is the possible mechanism of the candidate probiotics

Tian P et al. 2021b[85]	C57BL/6 mice	CUMS-depression	Probiotics	Bifidobacterium	B. breve CCFM1025 and FHLJDQ3M5	Probiotic treatment persisted for 6 weeks by daily oral gavage. Lyophilized bacteria powder were re-suspended in 10% skim cow milk and administered at a dose of 10^9 CFU/mL viable bacteria.	CCFM1025 significantly decreases the chronically stressed mice's depressive-like behaviors	CCFM1025 altered genomic and metabolic features involving glycoside hydrolases and neuromodulatory metabolites
Tian T et al. 2019[86]	C57BL/6 mice	CSDS-depression	Probiotics	Clostridium	C. butyricum Miyairi 588 (CBM588)	SPF mice were provided sterile water containing Miyairi 588 (>5 ×106/CFU) for 4 weeks ad libitum	CBM588 pre-feeding attenuates social avoidance and depressive-like behaviours in CSDS mice	CBM588 may be involved in the regulation of microglia-mediated immune responses in the brain, and regulated gut microbiota composition
Tillmann S et al. 2019[87]	Flinders Sensitive Line rats<br>Flinders resistant line rats	FSL-depression	Probiotics	Bifidobacterium, Lactobacillus, Lactococcus	Ecologic® Barrier + 4:<br>B. breve W25, B. longum W108, L. helveticus W74, and L. rhamnosus W71<br>Ecologic® Barrier:<br>B. bifidum W23, B. lactis W51, B. lactis W52, L. acidophilus W37, L. brevis W63, L. casei W56, L. salivarius W24, Lc. lactis W19, and Lc. lactis W58	Rats received a daily dose of 5 × 10^9 CFU/2 g for Ecologic® Barrier and a daily dose of 5 × 10^9 CFU/1.25 g for Ecologic® Barrier + 4, administered in water bottles at a volume of 60 mL	Probiotics exhibited risk-reducing properties (depressive-related behavior)	_
Trudeau F et al. 2019[88]	Sprague-Dawley rats	Post-myocardial infarction-depression	Probiotics	Lactobacillus, Bifidobacterium	L. helveticus R0052; B. longum R0175; L. salivarius HA-118	Each rat in the probiotic groups received a daily dose of 10^9 CFU for 14 days	B. longum mitigated the depressive-like symptoms	B. longum reduced the Caspase-3 activity and plasma C-reactive protein concentrations in the lateral and medial amygdala
Wang P et al. 2021[89]	C57BL/6J mice	Antibiotic-depression	Probiotics	Bifidobacterium, Lactobacillus	Unspecific	Probiotics (Live Combined Bifidobacterium and Lactobacillus Tablets, Inner Mongolia Shuang Qi Pharmaceutical Co., Ltd. PRC, Inner Mongolia, China) for another 2 weeks	Probiotics treatment alleviated anxiety behaviors, depressive-like behaviors and recognitive performance	Probiotics treatment improved neuronal activation in different brain regions, characterized by increased expression of Fos protein
Wang S et al. 2020a[90]	C57BL/6 mice	FMT CSDS-depression<br>Microbe-depression	Probiotics	Lactobacillus	L. intestinalis, L. reuteri	Mice were orally administered water containing the microbes (approximately 1 ×108 CFU/day) for 14 days (days 15–28) using gastric gavage	Ingestion of L. intestinalis and L. reuteri causes depression- and anhedonia-like phenotypes	Ingestion of L. intestinalis and L. reuteri caused biochemical abnormalities in antibiotictreated mice via the subdiaphragmatic vagus nerve
Wei CL et al. 2019[91]	C57BL/6J mice	Corticosterone-depression	Probiotics	Lactobacillus	Live and heat-killed L. paracasei PS23	The L-PS23 group received a daily oral gavage of L-PS23 (108 cells/0.2 ml/day) from days 1 to 41, and the H-PS23 group received a daily gavage of H-PS23 at the same dose and duration.	PS23 reverses corticosterone treatment–induced anxiety-like and depression-like behaviors	The effects of chronic PS23 treatment are due to (1) increases in the hippocampal GR, MR, and BDNF proteins; (2) increases in serotonergic and dopaminergic activities in the hippocampus, prefrontal cortex, and striatum;and (3) improvement of the gut microbiota

Xie R et al. 2020[92]	C57BL/6 mice	CSDS-depression	Probiotics	Lactobacillus	L. reuteri 3	Treating for 4 weeks with L. reuteri 3 (10^{10} CFU/ml of per mouse in 0.1 ml phosphate-buffered saline [PBS]) after 10 days of CSDS	Treatment with L. reuteri 3 ameliorated depressive-like behaviors	Treatment with L. reuteri 3 regulated the gut microbiota, SCFAs, and serotonin metabolism
Xu J et al. 2022[93]	C57BL/6 mice	CUMS-depression	Probiotics	Lactobacillus	L. rhamnosus zz-1	Mice received L. rhamnosus zz-1 at a dose of 2×10^9 CFU per kg bw ,2×10^8 CFU per kg bw or 2×10^7 CFU per kg bw for 6 weeks.The volume of the daily gavage liquid was adjusted to 0.1 mL.	L. rhamnosus zz-1 intervention ameliorated CUMS-induced depression-like behaviors	L. rhamnosus zz-1 improved stress-induced physiological problems in model mice, including HPA axis hyperactivity, neurotransmitter deficiency, and impairments in the BDNF-TrkB signaling, and regulated gut microbiota
Xu M et al. 2022[94]	C57BL/6J mice	CUS-depression	Probiotics	Lactobacillus	L. paracasei 126L6, CCFM1229, 29L1, 4L3, L. helveticus 132M1, 8G3, Q7M66, 10M6, L. rhamnosus CCFM1131, CCFM1130, CCFM1228, L. reuteri CCFM1132, 11M59	The freeze-dried bacterial powder was suspended in sterile skimmed milk. The concentration of surviving bacteria was 5×10^9 CFU/mL. The gavage volume of each mouse is 200 μL for 6 weeks	L. paracasei CCFM1229 and L. rhamnosus CCFM1228 significantly reduced anxiety- and depression-related behaviour	The strains CCFM1229 and CCFM1228 regulated the gut micrbiota and xanthine oxidase activity in the brain
Yang Y et al. 2022[95]	Sprague-Dawley rats	MS-postpartum depression	Probiotics	Lactobacillus	L. casei	From postnatal day 2 to day 28, rats were gavage-fed with Lactobacillus casei (8 ×108 CFU/kg/day)	Administration of L. casei improved depressive-like behaviors	Administration of L. casei altered gut microbiota composition, brain monoamines and oxidative stress, which may be associated with the regulation of the BDNF-ERK1/2 pathway
Yun SW et al. 2020[96]	C57BL/6J mice	EC-depression	Probiotics	Lactobacillus	L. gasseri NK109	NK109 at a dosage of 1×10^9 CFUs/mouse/day was orally gavaged once a day for 5 days in the mice with Escherichia coli K1 (1×10^9 CFUs/mouse/day)-induced depression	NK109 significantly alleviated Escherichia coli K1-induced cognitive impairment- and depression-like behaviors	NK109 regulated the immune response through NF-κB-involved BDNF expression, IL-1β expression, and vagus nerve-mediated gut–brain signaling, and mitigated Escherichia coli-induced colitis and gut dysbiosis
Yun SW et al. 2021[97]	C57BL/6J mice	EC-depression	Probiotics	Lactobacillus	L. paracasei NK112	Receiving NK112 (1×10^9 CFU/mouse/day) daily for 5 days	Oral gavage of NK112 significantly alleviated K1-induced anxious, depressive, and memory-impaired behaviours	NK112 treatment suppressed IL-6, TNF-α, and BDNF expression through the regulation of gut microbiota and NF-κB activation
Yunes RA et al. 2020[98]	BALB/c mice	Healthy status	Probiotics	Lactobacillus, Bifidobacterium	L. plantarum 90sk, B. adolescentis 150	One dose (0.5 ml) of the mixture of strains contained 10^8 CFU L. plantarum 90sk and 10^7 CFU B. adolescentis 150 for 14 days	Administration of the probiotic composition decreased the duration of immobility of mice	_
Zhao Y et al. 2020[99]	Sprague-Dawley rats	Corticosterone-depression	Probiotics	Lactobacillus	Lactobacillus plantarum DP189	Administration of DP189 (1.0×10^9 CFU/d) suspension by gavage for 21 days	L. plantarum DP189 treatment prevented and/or alleviated depression-like behaviors	L. plantarum DP189 treatment increased neurotransmitters in brain tissue, reduced serum levels of inflammatory factors, and regulated hippocampal neural apoptosis
Burokas A et al. 2017[100]	C57BL/6J mice	Prebiotics-anti-depression CSDS-depression	Prebiotics	_	Fructo-oligosaccharides (FOS), Galacto-oligosaccharides (GOS)	Administing the prebiotics FOS, GOS, a combination of FOS and GOS (dissolved in drinking water for 0.3–0.4 g/mouse/day) for 3 weeks	FOS+GOS administration significantly improved the depressive- and anxiety-like behaviors	Prebiotic administration significantly decreased the hypothalamic-pituitary-adrenal axis (corticosterone levels), influenced hippocampal and hypothalamic gene expression,

								improved the tryptophan and monoamines metabolism, and normalized the effects of stress on the microbiota
Chen Y et al. 2021a[101]	C57BL/6 mice	CUMS-depression	Prebiotics	_	Partially hydrolyzed guar gum (PHGG)	After 28 days of CUMS, mice received 600 mg/kg PHGG	PHGG significantly inhibited the loss of body weight, and prevented CUMS-induced depressive-like behavior in mice	PHGG modulated the gut microbiota structure and then increased the levels of short-chain fatty acids in mice feces and the levels of 5-hydroxytryptamine and dopamine in serum, striatum, and hippocampus
Cheng D et al. 2018[102]	Sprague-Dawley rats	Hydrocortisone-depression	Prebiotics	_	Tiansi Liquid	The dose of Tiansi Liquid was 0.45 g/kg once a day for 21 days	Tiansi Liquid ameliorated depressive symptoms in rats	Tiansi Liquid modulated the gut microbiota composition and metabolites in the tryptophan-kynurenine pathway
Chi L et al. 2020[103]	Sprague-Dawley rats	CUMS-depression	Prebiotics	_	Fructo-oligosaccharides (FOS)	Administration with FOS (50 mg/kg) via oral gavage for 3 weeks from the fifth week onward	FOS administration alleviated depressive-like behaviors	FOS administration repaired intestinal epithelia damages, decreased the hypothalamic-pituitary-adrenal axis (corticosterone levels), and modified the gut microbiota
Davis DJ et al. 2017[104]	C57BL/6J mice	Social isolation-depression	Prebiotics	_	N-3 polyunsaturated fatty acid docosahexaenoic acid (DHA)	The mice were then treated with either 0.1% by weight or 1.0% by weight DHA	A DHA diet, regardless of dose, exhibited reduced anxiety and depressive-like behaviors only in male mice	DHA altered the commensal community composition
Donoso F et al. 2020[105]	Sprague-Dawley rats	MS-depression	Prebiotics	_	Polyphenols: phlorotannins, xanthohumol, quercetin	Dietary intervention of polyphenols (Phlorotannins 0.03%; Xanthohumol 0.015%; Quercetin 0.03%), delivered ad libitum in food, began once the animals were eight weeks old and continued for eight weeks.	Polyphenols reversed MS-induced depressive- and anxiety-like behaviours	Polyphenols treatment prevented exacerbated production of corticosterone after acute stress in MS animals, reversed MS-induced plasma BDNF depletion and changes in diversity
Egerton S et al. 2020[106]	Sprague–Dawley rats	MS-depression	Prebiotics	_	Fish oil (containing polyunsaturated fatty acid)	Fish oil (composition fatty acid profile and vitamins & minerals) was added to the diets in the place of soybean oil in the standard chow, at 7% of total feed from 9 to 16 weeks of age	Fish oil dietary supplementation partly prevented the depressive-like behaviours	Fish oil dietary supplementation altered brain fatty acids, significantly decreased plasma corticosterone levels and reduced brain stem serotonin turnover, and regulated the gut microbial composition
Fan L et al. 2021[107]	Sprague-Dawley rats	CUMS-depression	Prebiotics	_	Cistanche tubulosa extracts: total glycosides, Cistanche tubulosa aqueous extract, phenylethanoid and iridoid glycosides	Reciving different dose of extracts for 4 weeks	Cistanche tubulosa extracts prevented the depressive-like behaviours	Cistanche tubulosa extracts regulated the hyperactivation of the HPA axis, severe peripheral and neural inflammation, and deficiencies in 5-HT and BDNF in the hippocampus
Gao X et al. 2020[108]	Sprague-Dawley rats	CUMS-depression	Prebiotics	_	Triterpenoids extracts from Poria cocos (TPC)	Reciving TPC at 15 g herb/kg 30 min before stressing exposure lasting 28 days	TPC significantly ameliorated depression-like behaviors in CUMS rats	TPC treatment restored the level of the neurotrophic factor system and regulated the gut microbiota composition, and regulated metabolic system, including primary bile acid biosynthesis, taurine and hypotaurine metabolism, arginine and proline metabolism
Gong MJ et al. 2016[109]	Sprague-Dawley rats	Corticosterone-depression	Prebiotics	_	Icariin	The treatment group was treated with icariin (60 mg/kg, suspended in saline) by gastric instillation 1 h prior to CORT injection once a day for 21 days	Icariin produced an antidepressant-like effect in CORT-induced depressive rats	Icariin increased the BDNF expression in the hippocampus, regulated the energy metabolism, lipid metabolism, amino acid metabolism and gut microbe metabolism

Guo Y et al. 2018[110]	ICR mice	CRS-depression	Prebiotics	_	Rosemary extracts	Reciving rosemary extracts (100 mg/kg) for 21 days during CRS stress	Pretreatment with rosemary extracts prevented the depressive- and anxiety-like behaviors	Rosemary extracts improved antiinflammatory effects in hippocampus, serum and BV-2 microglia as well as rebalanced the gut microbiota
Hao WZ et al. 2021[111]	C57BL/6 mice	CUMS-depression	Prebiotics	_	Coniferyl ferulate	The mice received coniferyl ferulate at a dose of 50 mg/kg once daily via gavage for 4 weeks	Oral administration of coniferyl ferulate attenuated weight loss and depression-like and anxiety-like behaviors induced by CUMS in mice	Coniferyl ferulate administration significantly ameliorated colonic inflammation, lowered the levels of IL-6, IL-1β, and TNF-α, and restructured the gut microbiome, and microbial metabolism
Huang YJ et al. 2021[112]	C57BL/6J mice	sCSDS-depresstion	Prebiotics	_	Water extract of Gastrodia elata (WGE)	WGE was administered at an optimal dose of 500 mg/kg bw via gavage once a day for 30 successive days	Oral treatment with WGE resulted in reversal of depression-like behavior	WGE exerts antidepressant-like effects mediated by the serotonergic and KYN pathways in the prefrontal cortex and colon, and altered the gut microbiota composition
Lai WD et al. 2022[113]	Wistar rats	SD-depression	Prebiotics	_	Fish oil	Feeding with a fish oil-rich diet for 10 weeks	A fish oil-based diet reduced anxiety- and depressive-like behaviors, and improved cognitive function under chronic SD.	A fish oil-based diet increased the probiotics production, increased the SCFA content, improved the intestinal barrier, increased SCFA receptor expression, and decreased blood circulation proinflammatory status
Lax NC et al. 2018[114]	C57Bl/6J mice	_	Prebiotics	_	Cyanobacterial extract DUQ0002I	For all injections, fraction DUQ0002I and subfractions DUQ0002I-1A-C, DUQ0002-2-4 were administered at a dose of 40 μg per cannula	DUQ0002I induced robust antidepressant and anxiolytic-effects	This extract blocked the 5-HT7R
Lee HC et al. 2020[115]	BALB/c mice	Lard diet-depression	Prebiotics	_	Fish oil (containing polyunsaturated fatty acid)-based diet	Treatment with fish oil concentrated with 50% EPA and 20% DHA triacylglycerol form for 12 weeks	Treatment with fish oil prevented depressive-like behavior	Treatment with fish oil regulated gut microbiota composition, and the prefrontal cortex fatty acid profile
Li Y et al. 2018[116]	Sprague-Dawley rats	CUS-depression	Prebiotics	_	Cistanche tubulosa extract (CTE)	CTE at high dose (CTEH) (400 mg/kg) and low dose (CTEL) (200 mg/kg) were intragastrically administered 1 h before the CUS procedure (8:00 a.m. to 9:00 a.m.) over the course of 4 weeks	CTE significantly improved depression-like behaviors in rats under CUS	CTE restored the level of neurotransmitters and neurotrophic factors in CUS rats, regulated the gut microbial composition, and modulated SCFAs concentrations
Lin S et al. 2021[117]	ICR mice	CRS-depression	Prebiotics	_	Crocetin	Reciving crocetin-L (20 mg/kg), crocetin-M (40 mg/kg), crocetin-H (80 mg/kg) for 28 days	Crocetin ameliorated CRS-induced depression-like behaviors in mice	Crocetin regulated MKP-1/ERK1/2/CREB pathway and gut microbiota
Liu Z et al. 2020[118]	C57BL/6J mice	HFD-postpartum depression	Prebiotics	_	Inulin	Taking standard diet with 37 g inulin/1000 kcal for 8−10 weeks	Inulin intake significantly attenuated cognitive deficits and depressive-like behaviors	Inulin intake upregulated the monoamine neurotransmitters (5-hydroxytryptamine and norepinephrine) and suppressed neuroinflammation
Mika A et al. 2017[119]	F344 rats	LH-depression	Prebiotics	_	Galactooligosaccharide (GOS), polydextrose (PDX)	Rats began diets on postnatal day 24 (PND 24) with GOS and PDX (7.0 g/kg each) for 4 weeks	Prebiotics differentially attenuated stress-induced learned helplessness	Prebiotics diets reduced stress-evoked cfos mRNA in the dorsal raphe nucleus (DRN), attenuated stress-evoked decreases in mRNA for the 5-HT1A autoreceptor in the DRN, GOS and PDX diet increased basal BDNF mRNA within the prefrontal cortex

O'Mahony SM et al. 2020[120]	Sprague-Dawley rats	MS-depression	Prebiotics	_	Polydextrose/galacto-oligosaccharide prebiotic blend	Test diets differed from control diet by the inclusion of (a) GOS 20.86 g/kg and PDX 6.44 g/kg (Prebiotic)	Dietary interventions altered stress-induced spatial learning and memory	Dietary interventions regulated the hypothalamic-pituitary-adrenal axis, reduced the long-term impact of MS on myelination, and influenced the gut microbiota composition
Pusceddu MM et al. 2015[121]	Sprague-Dawley rats	MS	Prebiotics	_	N-3 Polyunsaturated Fatty Acids (PUFAs)	Oral administration of an eicosapentaenoic acid (EPA)/docosaexaenoic acid (DHA) (80% EPA, 20% DHA) (0.4 g/kg/day or 1 g/kg/day) n-3 PUFAs mixture was administered by gavage when animals reached 5 weeks of age	No data	Long-term supplementation of EPA/DHA restored the microbiota composition
Qu Y et al. 2020[122]	C57BL/6 mice	CSDS-depression	Prebiotics	_	Betaine	Betaine (2.5% as monohydrate in drinking water) was given to mice for 24 days from day 1 to day 24	Betaine supplementation contributed to resilience to anhedonia in mice subjected to CSDS	Betaine had prophylactic effects for abnormal composition of gut microbiota in mice after CSDS and anti-inflammation action
Robertson RC et al. 2017[123]	C57BL/6J mice	n-3 PUFA deficiency-depression	Prebiotics	_	N-3 Polyunsaturated Fatty Acids (PUFAs)	Feding with n-3 PUFAs supplemented diet (1 g Eicosapentaenoic acid (EPA) + Docosahexaenoic acid (DHA)/100 g diet) or n-3 PUFAs deficient diet from gestational day 0	N-3 PUFAs supplementation prevented depressive-like behaviors and memory defect	N-3 PUFAs supplementation comprehensively alterated the gut microbiota composition, HPA-axis activity and inflammation
Song J et al. 2019b[124]	Wistar rats	ACTH-depression	Prebiotics	_	Polyphenols: chlorogenic acid (CGA)	CGA pretreatment (500 mg/kg) by intragastric administration 1 h prior to ACTH injection once a day for 14 days	CGA pretreatment ameliorated depressive-like behavior	CGA pretreatment increased the level of monoamine neurotransmitters and reduced the levels of inflammatory cytokines, and modified gut microbial community structure
Song X et al. 2021[125]	ICR mice	CUMS-depression	Prebiotics	_	Puerarin	Low and high doses of Puerarin were 30 and 100 mg/kg, respectively, and these were administered intragastrically in the model	Puerarin (100 mg/kg) treatment was found to alleviate the CUMS-induced depression-like behaviors	Puerarin treatment reversed the gut microbial changes induced by CUMS
Sun Y et al. 2020[126]	C57BL/6 mice	LPS-depression	Prebiotics	_	Schisandrin	Mice were treated with schisandrin (30 mg/kg, i.p.) for 14 days	Schisandrin pre-treatment attenuated LPS-induced depressive-like behaviors in mice	Schisandrin might recover the gut microbial disorder of depressive mice through suppressing the expression of TLR4/NF-κB signaling pathway
Tian P et al. 2021[127]	mice	CRS-depression	Prebiotics	_	SCFA-Acylated Starches	Feeding with 15% acylated starch for 2 weeks during chronic restraint stress	Consumption of SCFA-acylated starches alleviated the depressive symptoms of stressed mice	SCFA-Acylated Starches significantly reduced the colonic permeability via increasing the tight junction proteins (including ZO-1, Claudin, and Occludin) gene expression and reduced the level of the inflammatory cytokines, and modified gut microbiome
Tung TH et al. 2019[128]	Sprague-Dawley rats	CMS-depression	Prebiotics	_	Fish oil (containing polyunsaturated fatty acid) and olive oil	Male rats were fed fish oil-rich (contained 20.5% (w/w) EPA and 11.2% DHA) or olive oil-rich diets for 14 weeks	Fish oil intervention reversed the stress-induced abnormal depressive-like behavior	Fish oil and olive oil exerted part of a prebiotic-like effect to ameliorate dysbiosis induced by CMS
Valdés-Sustaita B et al. 2021[129]	Wistar rats	Ovariectomized-depression	Prebiotics	_	Aqueous extract of pomegranate (AE-PG)	The pomegranate extract (AE-PG) was dissolved in saline solution 0.9% and given by intraperitoneal route.	AE-PG administered by intraperitoneal route induced antidepressant-like effects	AE-PG given intraperitoneally activated the ERβ and the serotonergic system

Wang L et al. 2020[130]	Kumming mice	CUMS-depression	Prebiotics	_	Total iridoids of Valeriana jatamansi (TIV)	Treated with lowdose, medium-dose, or high-dose TIV (5.7, 11.4, and 22.9 mg/kg/d, respectively) at a volume of 10 mL/kg by the intragastric route once per day for 2 successive weeks	Administration of TIV increased body weight, sucrose solution consumption, and ameliorated depression-like behaviors	It is concluded that the antidepressant effects of TIV may be related to gut flora structures and regulation of 5-HT, NE, SP, and CRF in the brain and intestine
Wang L et al. 2021[131]	Sprague-Dawley rats	CUMS-depression	Prebiotics	_	Soy isoflavones	The SI low dose, SI middle dose, and SI high dose groups were given SI at a dose of 40 mg/kg, 80 mg/kg, and 160 mg/kg per group per rat per day by oral gavage for 8 weeks	Soy isoflavones supplements significantly improved the CUMS-induced depression-like behaviour	Soy isoflavones supplements increased monoamine neurotransmitters of CUMS rats by reshaping the structure of the gut microbiota
Wang P et al. 2022[132]	C57BL/6J mice	Alcohol-depression	Prebiotics	_	Propolis	Propolis group was given 120 mg/kg of propolis by gavage daily for 10 weeks	Propolis exerted an improving effect on alcohol-induced depressive symptoms	Propolis dietary supplementation repaired the intestinal mucosal barrier and hippocampal injury, and further improved brain gut dysfunction.
Wang Q et al. 2019[133]	CD-1 mice	CMS-depression	Prebiotics	_	Sesamin	Orally administrated with sesamin (50 mg/kg/day, dissolved in olive oil) for 10 weeks	Oral sesamin administration (50 mg/kg bodyweight/day) significantly attenuated depressive, aversive, repetitive, and anxiety-like behaviors	Sesamin inhibited stress-induced gut barrier integrity damage, reduced circulating lipopolysaccharide levels, suppressed neuroinflammatory responses, and restructured the gut microbiome
Wang R et al. 2021[134]	C57BL/6J mice	CRS-depression	Prebiotics	_	Total Flavone of Abelmoschus manihot (TFA)	TFA was suspended in 1% carboxymethyl cellulose solution at different concentrations for oral administration (62.5 and 125 mg/kg) for 37 days	TFA treatment improved the depressive-like phenotype	TFA treatment improved the disturbed gut microbiota, and the intestinal barrier function
Xia J et al. 2021[135]	C57BL/6J mice	LPS-depression	Prebiotics	_	Capsaicin	The mice were fed standard laboratory chow plus 0.005% capsaicin for 4 months	Dietary capsaicin improved depressive-like behavior	Dietary CAP regulated the structure of gut microbiota, increased the levels of the monoamine neurotransmitter 5-HT, and reduced the levels of inflammatory cytokine TNF-a in LPS-induced mice
Xiao Q et al. 2020[136]	C57BL/6 mice	CRS-depression	Prebiotics	_	Crocin-I	Mice were daily supplied with crocin-I at a dose of 40 mg/kg by gavage for six weeks	Administration of crocin-I mitigated depression-like behaviors	Oral administration of crocin-I improved the structure of gut microbiota to restore SCFAs levels and intestinal barrier function, thereby decreasing the neuroinflammation and increasing BDNF protein to effectively alleviated depression-like behavior in depressed mice
Xue M et al. 2021[137]	C57BL/6J mice	Alcohol-depression	Prebiotics	_	Fucoidan extracted from Fucus vesiculosus	Receiving 300 mg/kg.body weight of fucoidan at 12:00 a.m. during the 10 weeks' experiment.	Oral administration of fucoidan alleviated alcohol withdrawal-induced depressionlike behaviors of mice	Oral administration of fucoidan regulated the gut flora of mice and reduce endotoxemia, down-regulated the TLR4/MyD88/NF-κB p65 pathway, inhibited alcohol-induced microglia cell activation and inflammation
Yan T et al. 2020[138]	C57BL/6 mice	CUMS-depression	Prebiotics	_	Polysaccharide from okra (Abelmoschus esculentus (L) Moench)	Mice were treated with polysaccharide (400 mg/kg, i.g.) for 14 days	Polysaccharide treatment alleviated depressive-like behaviors in CUMS-induced mice	Polysaccharide treatment inhibitated the inactivation of inflammatory reactions in the colon, serum, hippocampus as well as BV2 cells, down-regulated the TLR4/ NF-κB

								pathway and MAPKs signaling, and rebalanced the intestinal flora
Yan T et al. 2021[139]	C57BL/6 mice	LPS-depression	Prebiotics	_	Schisandra chinensis fractions: total extracts (SCE), lignans (SCL), polysaccharides (SCPS), and essential oil (SCVO)	Mice were treated with fractions (SCE treatment [1.2 g/kg], SCL treatment [500 mg/kg], SCPS treatment [300 mg/kg] , SCVO treatment [150 mg/kg]) (i.g.) for 14 days	Fractions treatment alleviated depressive-like behaviors in LPS-induced mice	Fractions treatment regulated the neuroinflammation via the TLR4/NF-κB/IKKα signaling pathway, and recovered the gut microbiota
Yu JB et al. 2019[140]	Sprague-Dawley rats	CUS-depression	Prebiotics	_	Paeoniflorin	Mice were treated with paeoniflorin at a dose of 10 mg/kg or 20 mg/kg for 8 weeks	Paeoniflorin treatment alleviated depressive-like behaviors in CUS-induced mice	Paeoniflorin regulated the composition of the gut microbiota by increasing the abundance of probiotics. And benzoic acid, the gut characteristic metabolite of paeoniflorin, was absorbed into blood and penetrated the BBB and entered the central nervous system relieving depressive behaviors
Zhang L et al. 2021[141]	Kumming mice	CUMS-depression	Prebiotics	_	Total iridoids of Valeriana jatamansi (TIV)	Treated with lowdose, medium-dose, or high-dose TIV (5.7, 11.4, and 22.9 mg/kg/d, respectively) at a volume of 10 mL/kg by the intragastric route once per day for 2 successive weeks	Administration of TIV increased body weight, sucrose solution consumption, and ameliorated depression-like behaviors	TIV may modulate the intestinal flora, thereby inducing the expression of ZO-1 and occludin, protecting the blood–brain barrier
Zhang M et al. 2021[142]	ICR mice	CUMS-depression	Prebiotics	_	Sophora alopecuroides L.-derived alkaloids	Alkaloids were dissolved in 0.9% saline at a stock concentration of 3 mg/ml, and the mice recived 30 mg/kg alkaloids for 4 weeks	Sophora alopecuroides L.-derived alkaloids improved depression-like behaviors and depression-related indicators in mice.	Alkaloids improved depression in mice through modulating gut microbiota
Zhang Z et al. 2022[143]	C57BL/6 mice	CRS-depression	Prebiotics	_	Hyperforin	Hyperforin is dissolved in DMSO and the dosage of hyperforin is 0.38mg/kg.Mice in hyperforin treated group were intraperitoneally injected with hyperforin solution before daily restraint	Hyperforin prevented anhedonia induced by CRS in mice	Hyperforin prevented altered the richness and evenness of bacteria populations
Zhao B et al. 2020[144]	C57BL/6 mice	DSS-depression	Prebiotics	_	Lycopene	Treating with distilled water and lycopene (50 mg/kg body weight/day) mixed in standard diet (AIN-93M) for 35 days	Lycopene improved DSS-induced depression and anxiety-like behavioral disorders	Lycopene suppressed neuroinflammation and prevented synaptic ultrastructure damages by upregulating the expressions of neurotrophic factor and postsynaptic-density protein, and reshaped the gut microbiome and improved the gut barrier integrity
Zhao F et al. 2021[145]	Sprague-Dawley rats	Prenatal CUMS-depression in offsprings	Prebiotics	_	Lycium barbarum polysaccharide (LBP)	Gavaging with LBP at 14:00 daily, the concentration of gavage is 40 mg/kg, for 14 days	LBP treatment improved the body weight, changed the emotional function	LBP treatment reduced offspring's plasma corticosterone level and increased the diversity of gut microbiota

Zhao ZX et al. 2018[146]	Sprague-Dawley rats	CUS-depression	Prebiotics	_	Albiflorin	Treating with benzoic acid (14 mg/kg)(albiflorin metabolites),albiflorin (7 mg/kg),albiflorin (14 mg/kg)for 2 weeks	Benzoic acid, a therapeutic mediator of albiflorin generated by the gut microbiota, after crossing the blood-brain barrier, entered the central nervous system to exert antidepressant effects	Albiflorin treatment regulated the gut microbiota and inhibited D-amino acid oxidase in the brain
Li H et al. 2019[147]	Wistar rats	CUMS-depression	Probiotics, prebiotics	Bifidobacterium, Lactobacillus	Fructo-oligosaccharides (FOS), Galacto-oligosaccharides (GOS), B. longum, L. rhamnosus	Orally gavaged with with FOS and GOS (8%, 1 mL per 100g weight; FOS/GOS) or with B. longum (1×10^9 CFU per 100 g weight) or with L. rhamnosus (1×10^9 CFU per 100 g weight) during the CUMS molding for 4 weeks	Prebiotics (FOS/GOS) and probiotics (B. longum and L rhamnosus) alleviated CUMS-induced depressive-like behaviors	Prebiotics and probiotics treatment has a considerable impact on the modulation of tryptophan metabolism and the composition of gut microbiota
Gilbert K et al. 2012[148]	Sprague-Dawley rats	Post-myocardial infarction depression	Probiotics, prebiotics	Lactobacillus, Bifidobacterium	L. helveticus R0052, B. longum R0175, and PUFA n-3 diets	Each rat in the probiotics group received a daily dose of 10^9 CFU for 2 weeks	Depressive-like behaviour was attenuated with the high-PUFA n-3 diet or/and probiotics	A high-PUFA n-3 diet or the administration of probiotics were beneficial to attenuate apoptosis in the limbic system in the rat
Zhu X et al. 2017[149]	Sprague-Dawley rats	CUS-depression	Probiotics, prebiotics	Bifidobacterium	Bifidobacterium, Berberine	Prior to modeling with each chronic unpredictable stress method, the rats were treated with either 2 ml of a low concentration of berberine (40 mg/kg/day), a high concentration of berberine (200 mg/kg/day), bifidobacterium (140 mg/kg/day)	Berberine and bifidobacterium treatment alleviated depressive behaviors caused by CUS	Berberine and bifidobacterium appeared to reverse the physical damage brought about by stress within the gastric mucosa and intestinal microvilli of the stomach, ileum, cecum and colon
Westfall S et al. 2021a[150]	C57BL/6J mice	CUMS-depression<br>CUMS+US-depression	Probiotics, prebiotics, and synbiotics		L. plantarum ATCC 793, B. longum ATCC 15707, Bioactive Dietary Polyphenol Preparation (BDPP)	The bacteria were incorporated into the animals' drinking water at a final dosage of 1.0×10^9 CFU/day per bacterium; BDPP was comprised of 1% w/v grape seed polyphenol extract, 1% w/v resveratrol and a 5% w/v concord grape extract made in sterile water during the experiment	The probiotic and synbiotic attenuated depressive-like behavior following CUS, while the synbiotic rescued the phenotype following CUS and CUS+US; only BDPP and the synbiotic improved anxiety-like behavior	Microbiota-targeted treatment normalized gut microbiota populations and promoted regulatory T cell (Treg) expansion through modulation of ileal innate lymphoid cell (ILC)3 activity, an impact reflecting behavioral responses better than limbic brain region neuroinflammation
Westfall S et al. 2021b[151]	C57BL/6J mice	CUS-depression	Probiotics, prebiotics, and synbiotics	Lactobacillus, Bifidobacterium	L. plantarum ATCC 793, B. longum ATCC 15707, Bioactive Dietary Polyphenol Preparation (BDPP)	The bacteria were incorporated into the animals' drinking water at a final dosage of 1.0×10^9 CFU/day per bacterium; BDPP was comprised of 1% w/v grape seed polyphenol extract, 1% w/v resveratrol and a 5% w/v concord grape extract made in sterile water during the experiment	The probiotic and synbiotic attenuated stress-induced depressive- and anxiety-like behaviors	A synbiotic rescued stress-induced reduction in serotonin through inflammatory and kynurenine pathway regulation, attenuated peripheral and neuroinflammatory responses, and ameliorated the stress-Induced increase of the T helper 17 to regulatory T cell ratio
Mesripour A et al. 2021[152]	albino mice	Dexamethasone or water avoidance stress induced depression	Synbiotics	Lactobacillus, Bifidobacterium, Streptococcus	Syn cocktail containing L. casei, L. acidophilus, L. rhamnosus, L. bulgaricus, B. breve, B. infantis, S.	The synbiotic regimen (12.5×10^6 CFU) was supplemented in drinking water for 7 days	Synbiotic mixture prevented the effects of WAS, acute or sub-acute Dex-induced depression in mice	_

					thermophiles, and Fructooligosaccharides			
Leo A et al. 2021[153]	C57Bl/6J mice	CUMS-depression	Prebiotics, Postbiotics	_	α-lactalbumin (ALAC) Sodium butyrate (NaB)	ALAC (125, 250 and 500 mg/kg), NaB (30, 100, 300 mg/kg) and the co-administration of ALAC (125, 250 and 500 mg/kg) with a fixed NaB dose (100 mg/kg) were administered in drinking water for 15 days	ALAC, NaB and their combination reduced depressive- and anxiety-like behaviour in CUMS mice	_
Cheng R et al. 2021[154]	C57BL/6 mice	CUMS-depression	Postbiotics	Akkermansia	Outer membrane protein Amuc_1100 of A. muciniphila	Mice were gavaged daily with 80 mg of Amuc_1100 in sterile PBS with a volume of 200 ml	Amuc_1100 intervention ameliorated CUMS-induced depression-like behavior	Amuc_1100 intervention improved the gut microbiota, up-regulated the BDNF level, and inhibited the neuroinflammatory response
Kochanowska AJ et al. 2008[155]	Swiss Webster mice	Healthy status	Postbiotics	_	Secondary metabolites from sponges (brominated compounds, sesquiterpene quinones, hydroquinones)	Each group was injected ip with the compound at a dose of 1–20 mg/ kg	5,6-Dibromo-N,N-dimethyltryptamin possessed significant antidepressant-like activity	_
Li J et al. 2018[156]	Sprague-Dawley rats	CUMS-depression	Postbiotics	_	Sodium propionate (NaP, the salt form of propionic acid)	1 mL of NaP (200 mmol/L) was administrated intrarectally every day for 1 week from the beginning of the 5th week	Administration of NaP induced antidepressant-like effects	Administration of NaP rebalanced the plasma metabolome, and rescued the neurotransmitters in the prefrontal cortex, which may be achieved through the reduction of catabolism of noradrenaline, tryptophan and dopamine, rather than serotonin
Matsuda Y et al. 2020[157]	Sprague-Dawley rats	CSDS-depression	Postbiotics	_	Ergothioneine, a metabolite of Lactobacillus reuteri	Oral administration of L-ergothioneine (0.25 mg/ml) aqueous solution was conducted from 1 week prior to SDS initiation (day −7) to the end SDS application (day 14)	Oral administration of ergothioneine prior to and during the SDS paradigm had a preventative effect on SDS-induced depressive behaviors	_
Yu M et al. 2021[158]	Wistar rats	CVS-depression	A broad-spectrum β-lactam antibiotics	_	Tienam (imipenem/cilastatin sodium with equal quantities)	Imipenem/cilastatin sodium was administered at a daily dose of 75 mg/kg of body weight from day 25 to day 28 during 28 days' CVS	Antibiotic treatment reversed the depression-like behaviors	Antibiotic treatment regulated the purine metabolism and fatty acid metabolism that are impacted by gut bacteria
Suzuki K et al. 2021[159]	C57BL/6 J (B6J) mice	CSDS-depression	Antibacterial active peptides	_	α-defensin (Cryptdin-4)	Recombinant Cryptdin-4 (mouse α-defensin) were dissolved in ultrapure water and administered orally at 250 μg/mouse once daily from day 1 to day 32	No data	Administration of α-defensin recovered dysbiosis and significant microbial composition changes in the intestinal metabolites
Martin-Hernandez D et al. 2016[160]	Wistar rats	CMS-depression	Antibiotics	_	Streptomycin, Penicillin G	Giving drinking water ad libitum containing streptomycin sulfate (2 mg/mL) and penicillin G (1500 U/mL) for 21 days	Antibiotics treatment reversed the CMS-induced a depressive-like phenotype	Antibiotics treatment inhibited bacteria translocation that paly a role in the pathophysiology of depression through the p38 MAPK pathway which could aggravate the neuroinflammation and the oxidative/nitrosative damage

Meng C et al. 2022[161]	Sprague-Dawley rats	CUMS-depression	Antibiotics	_	Metronidazole, ciprofloxacin	Treating with metronidazole (1 g/L) and ciprofloxacin (0.2 g/) in drinking water for 5 weeks	Antibiotics exposure reduced anxiety-like and depression-like behavior of rat	Antibiotics regulated neurotransmitter and inflammatory response through gut microbiota
Schmidtner AK et al. 2019[162]	NAB/HAB rats	HAB-depression	Antibiotics	_	Minocycline	In the first set of experiments, 40 mg/kg/day minocycline, while in the second set, 80 mg/kg/day minocycline alone, all dissolved in tap water, was applied for 22 days	Three weeks of minocycline treatment alleviated the depressive-like phenotype	Minocycline treatment promoted an anti-inflammatory state by control of microglial activation and/or modulation of the gut microbiome and metabolome
Wang S et al. 2020b[163]	C57BL/6 mice	CSDS-depression	Antibiotics	_	Ampicillin, Neomycin sulfate, Metronidazole	Broad-spectrum antibiotics (ampicillin 1 g/L, neomycin sulfate 1 g/L, and metronidazole 1 g/L) dissolved in drinking water were provided ad libitum to male C57BL/6 mice for 14 consecutive days	CSDS did not produce an anhedonia-like phenotype in the antibiotic-treated mice	Antibiotic-treated reduced the diversity of gut microbiota, and changed their composition
Wong ML et al. 2016[164]	C57BL/6J mice	CRS-depression	Antibiotics	_	Minocycline	Treating with minocycline (LKT Laboratories, St Paul, MN, USA; 5 mg/kg per day in 10 ml/kg saline, intraperitoneally) for 21 days	Minocycline treatment decreased depressive- and anxiety-like behaviors	Minocycline treatment regulated the gut microbiota–inflammasome–brain axis, characterized by attenuated inflammation and rebalanced the gut microbiota
Yang Q et al. 2020[165]	C57BL/6 mice	CUMS-depression	Antibiotics	_	Minocycline	Minocycline (40 mg/kg, Cayman Chemical) was administered intraperitoneally daily for 4 weeks starting 2 weeks after CUMS	Minocycline treatment for 4 weeks, not acute treatment, exerted antidepressant effect in mice exposed to CUMS	Chronic minocycline treatment inhibited neuroinflammation of hippocampus and altered species abundance and metabolites of gut microbiota, and ameliorated intestinal barrier disruption and reduced the bacteriological indexes

References

1. J. Rao, R. Xie, L. Lin, J. Jiang, L. Du, X. Zeng, G. Li, C. Wang and Y. Qiao, *Eur J Neurosci* **2021**, *53*, 3598-3611.

2. J. Rao, Y. Qiao, R. Xie, L. Lin, J. Jiang, C. Wang and G. Li, *J Psychiatr Res* **2021b**, *137*, 147-157.

3. S.-K. Han, J.-K. Kim, H.-S. Park, Y.-J. Shin and D.-H. Kim, *Chin Med* **2021**, *16*, 77.

4. P. R. Marcondes Ávila, M. Fiorot, M. Michels, D. Dominguini, M. Abatti, A. Vieira, A. B. de Moura, J. P. Behenck, L. A. Borba, M. E. M. Botelho, G. Z. Réus, F. Dal-Pizzol and C. Ritter, *J Affect Disord* **2020**, *277*, 410-416.

5. Z. Xu, Z. Liu, X. Dong, T. Hu, L. Wang, J. Li, X. Liu and J. Sun, *Chin J Physiol* **2018**, *61*, 360-371.

6. Y. Zhang, R. Huang, M. Cheng, L. Wang, J. Chao, J. Li, P. Zheng, P. Xie, Z. Zhang and H. Yao, *Microbiome* **2019**, *7*, 116.

7. H. Zhou, S. Zhang, X. Zhang, H. Zhou, T. Wen and J. Wang, *Biochem Biophys Res Commun* **2022**, *593*, 137-143.

8. Z. Wu, P. Wang, D. Pan, X. Zeng, Y. Guo and G. Zhao, *J Dairy Sci* **2021**, *104*, 78-91.

9. S.-K. Han, M.-K. Joo, J.-K. Kim, W. Jeung, H. Kang and D.-H. Kim, *Nutrients* **2020a**, *12*,

10. C. Y. Ko, H.-T. V. Lin and G. J. Tsai, *Process Biochemistry* **2013**, *48*, 559-568.

11. A. K. Warda, K. Rea, P. Fitzgerald, C. Hueston, E. Gonzalez-Tortuero, T. G. Dinan and C. Hill, *Behav Brain Res* **2019**, *362*, 213-223.

12. N. Xu, W. Fan, X. Zhou, Y. Liu, P. Ma, S. Qi and B. Gu, *Metab Brain Dis* **2018**, *33*, 1625-1633.

13. A. Abildgaard, T. Kern, O. Pedersen, T. Hansen, S. Lund and G. Wegener, *Eur Neuropsychopharmacol* **2021**, *43*, 10-21.

14. N. Li, Q. Wang, Y. Wang, A. Sun, Y. Lin, Y. Jin and X. Li, *Front Behav Neurosci* **2018**, *12*, 266.

15. Q. F. Liu, H.-M. Kim, S. Lim, M.-J. Chung, C.-Y. Lim, B.-S. Koo and S.-S. Kang, *Daru* **2020**, *28*, 181-189.

16. Y. Ding, F. Bu, T. Chen, G. Shi, X. Yuan, Z. Feng, Z. Duan, R. Wang, S. Zhang, Q. Wang, J. Zhou and Y. Chen, *Appl Microbiol Biotechnol* **2021**, *105*, 8411-8426.

17. A. Abildgaard, B. Elfving, M. Hokland, G. Wegener and S. Lund, *Psychoneuroendocrinology* **2017a**, *79*, 40-48.

18. A. Abildgaard, B. Elfving, M. Hokland, S. Lund and G. Wegener, *Brain Behav Immun* **2017b**, *65*, 33-42.

19. A. Abildgaard, T. Kern, O. Pedersen, T. Hansen, G. Wegener and S. Lund, *Eur Neuropsychopharmacol* **2019**, *29*,

20. A. Agusti, A. Moya-Pérez, I. Campillo, S. Montserrat-de la Paz, V. Cerrudo, A. Perez-Villalba and Y. Sanz, *Mol Neurobiol* **2018**, *55*, 5337-5352.

21. J. Arseneault-Bréard, I. Rondeau, K. Gilbert, S.-A. Girard, T. A. Tompkins, R. Godbout and G. Rousseau, *Br J Nutr* **2012**, *107*, 1793-1799.

22. A. Arslanova, A. Tarasova, A. Alexandrova, V. Novoselova, I. Shaidullov, D. Khusnutdinova, T. Grigoryeva, D. Yarullina, O. Yakovleva and G. Sitdikova, *Life (Basel)* **2021**, *11*,

23. H. Aygun, A. T. Akin, N. Kızılaslan, O. Sumbul and D. Karabulut, *Epilepsy Behav* **2022**, *128*, 108588.

24. T. Barros-Santos, K. S. O. Silva, M. Libarino-Santos, C.-P. Elisangela Gouveia, H. S. Reis, E. K. Tamura, A. J. de Oliveira-Lima, L. F. Berro, A. P. T. Uetanabaro and E. A. V. Marinho, *PLoS One* **2020**, *15*, e0234037.

25. A. Bharwani, M. F. Mian, M. G. Surette, J. Bienenstock and P. Forsythe, *BMC Med* **2017**, *15*, 7.

26. P. T. Birmann, A. M. Casaril, A. P. Pesarico, P. S. Caballero, T. Â. Smaniotto, R. R. Rodrigues, Â. N. Moreira, F. R. Conceição, F. S. S. Sousa, T. Collares, F. K. Seixas, R. T. França, C. D. Corcini and L. Savegnago, *Pharmacol Res* **2021**, *171*, 105740.

27. J. A. Bravo, P. Forsythe, M. V. Chew, E. Escaravage, H. M. Savignac, T. G. Dinan, J. Bienenstock and J. F. Cryan, *Proc Natl Acad Sci U S A* **2011**, *108*, 16050-16055.

28. P. Chen, M. Hei, L. Kong, Y. Liu, Y. Yang, H. Mu, X. Zhang, S. Zhao and J. Duan, *Food Funct* **2019**, *10*, 8161-8171.

29. T. Chen, R. Wang, Z. Duan, X. Yuan, Y. Ding, Z. Feng, F. Bu, L. Liu, Q. Wang, J. Zhou, L. Zhu, Q. Ni, G. Shi and Y. Chen, *Front Cell Infect Microbiol* **2021**, *11*, 723856.

30. X. Chen, S. Meng, Y. Yu, S. Li, L. Wu and Y. Zhang, *Int J Occup Med Environ Health* **2022**, *35*,

31. Y. Chen, N. Xiao, Y. Chen, X. Chen, C. Zhong, Y. Cheng, B. Du and P. Li, *Food Res Int* **2021b**, *150*, 110808.

32. G. Chevalier, E. Siopi, L. Guenin-Macé, M. Pascal, T. Laval, A. Rifflet, I. G. Boneca, C. Demangel, B. Colsch, A. Pruvost, E. Chu-Van, A. Messager, F. Leulier, G. Lepousez, G. Eberl and P.-M. Lledo, *Nat Commun* **2020**, *11*, 6363.

33. J. Choi, Y.-K. Kim and P.-L. Han, *Exp Neurobiol* **2019**, *28*, 158-171.

34. J. Choi, H. Kwon, Y.-K. Kim and P.-L. Han, *Mol Neurobiol* **2022**, *59*, 2715-2728.

35. V. Daugé, C. Philippe, M. Mariadassou, O. Rué, J.-C. Martin, M.-N. Rossignol, N. Dourmap, L. Svilar, F. Tourniaire, M. Monnoye, D. Jardet, M. Bangratz, S. Holowacz, S. Rabot and L. Naudon, *Front Behav Neurosci* **2020**, *14*, 581296.

36. L. Desbonnet, L. Garrett, G. Clarke, B. Kiely, J. F. Cryan and T. G. Dinan, *Neuroscience* **2010**, *170*, 1179-1188.

37. J. Dhaliwal, D. P. Singh, S. Singh, A. K. Pinnaka, R. K. Boparai, M. Bishnoi, K. K. Kondepudi and K. Chopra, *J Appl Microbiol* **2018**, *125*, 257-269.

38. K. Gao, A. Farzi, X. Ke, Y. Yu, C. Chen, S. Chen, T. Yu, H. Wang and Y. Li, *Food Funct* **2022**, *13*, 957-969.

39. F. Gu, Y. Wu, Y. Liu, M. Dou, Y. Jiang and H. Liang, *Food Funct* **2020**, *11*, 6148-6157.

40. F. Guida, F. Turco, M. Iannotta, D. De Gregorio, I. Palumbo, G. Sarnelli, A. Furiano, F. Napolitano, S. Boccella, L. Luongo, M. Mazzitelli, A. Usiello, F. De Filippis, F. A. Iannotti, F. Piscitelli, D. Ercolini, V. de Novellis, V. Di Marzo, R. Cuomo and S. Maione, *Brain Behav Immun* **2018**, *67*, 230-245.

41. Y. Guo, J.-P. Xie, K. Deng, X. Li, Y. Yuan, Q. Xuan, J. Xie, X.-M. He, Q. Wang, J.-J. Li and H.-R. Luo, *Front Behav Neurosci* **2019**, *13*, 126.

42. S.-K. Han and D. H. Kim, *J Microbiol Biotechnol* **2019**, *29*, 1369-1374.

43. S.-K. Han, J.-K. Kim, M.-K. Joo, K.-E. Lee, S.-W. Han and D. H. Kim, *J Microbiol Biotechnol* **2020b**, *30*, 1222-1226.

44. W. Hao, J. Wu, N. Yuan, L. Gong, J. Huang, Q. Ma, H. Zhu, H. Gan, X. Da, L. Deng, X. Li and J. Chen, *Front Pharmacol* **2021**, *12*, 619103.

45. Z. Hao, W. Wang, R. Guo and H. Liu, *Psychoneuroendocrinology* **2019**, *104*, 132-142.

46. F. Huang, X. Liu, S. Xu, S. Hu, S. Wang, D. Shi, K. Wang, Z. Wang, Q. Lin, S. Li, S. Zhao, K. Jin, C. Wang, L. Chen and F. Wang, *Front Nutr* **2021**, *8*, 805465.

47. Y.-Y. Huang, Y.-P. Wu, X.-Z. Jia, J. Lin, L.-F. Xiao, D.-M. Liu and M.-H. Liang, *Food Funct* **2022**, *13*, 411-424.

48. H.-M. Jang, K.-E. Lee and D.-H. Kim, *Nutrients* **2019**, *11*,

49. J. Kambe, S. Watcharin, Y. Makioka-Itaya, R. Inoue, G. Watanabe, H. Yamaguchi and K. Nagaoka, *Neurosci Lett* **2020**, *720*, 134753.

50. C. Karen, D. J. H. Shyu and K. E. Rajan, *Front Neurosci* **2021**, *15*, 719933.

51. J.-K. Kim, K.-E. Lee, S.-A. Lee, H.-M. Jang and D.-H. Kim, *Front Immunol* **2020**, *11*, 273.

52. K. Kochalska, W. Oakden, T. Słowik, A. Chudzik, A. Pankowska, A. Łazorczyk, P. Kozioł, M. Andres-Mach, R. Pietura, R. Rola, G. J. Stanisz and A. Orzylowska, *Nutr Res* **2020**, *82*, 44-57.

53. A. Kosuge, K. Kunisawa, S. Arai, Y. Sugawara, K. Shinohara, T. Iida, B. Wulaer, T. Kawai, H. Fujigaki, Y. Yamamoto, K. Saito, T. Nabeshima and A. Mouri, *Brain Behav Immun* **2021**, *96*, 200-211.

54. Q. Li, L. Li, X. Niu, C. Tang, H. Wang, J. Gao and J. Hu, *Neuroreport* **2021**, *32*, 686-693.

55. S. Liang, T. Wang, X. Hu, J. Luo, W. Li, X. Wu, Y. Duan and F. Jin, *Neuroscience* **2015**, *310*, 561-577.

56. J. F. Liao, C. C. Hsu, G. T. Chou, J. S. Hsu, M. T. Liong and Y. C. Tsai, *Benef Microbes* **2019**, *10*, 425-436.

57. E. Y. Lim, E. J. Song, J. G. Kim, S. Y. Jung, S. Y. Lee, H. S. Shin, Y. D. Nam and Y. T. Kim, *Benef Microbes* **2021**, *12*, 503-516.

58. Y. Liu, M. F. Mian, K.-A. McVey Neufeld and P. Forsythe, *Brain Behav Immun* **2020**, *88*, 451-460.

59. Y.-W. Liu, W.-H. Liu, C.-C. Wu, Y.-C. Juan, Y.-C. Wu, H.-P. Tsai, S. Wang and Y.-C. Tsai, *Brain Res* **2016**, *1631*,

60. H. Maehata, Y. Kobayashi, E. Mitsuyama, T. Kawase, T. Kuhara, J.-Z. Xiao, T. Tsukahara and A. Toyoda, *Biosci Biotechnol Biochem* **2019**, *83*, 1239-1247.

61. K.-A. McVey Neufeld, S. Kay and J. Bienenstock, *Front Neurosci* **2018**, *12*, 294.

62. A. Moya-Pérez, A. Perez-Villalba, A. Benítez-Páez, I. Campillo and Y. Sanz, *Brain Behav Immun* **2017**, *65*, 43-56.

63. E. Murray, R. Sharma, K. B. Smith, K. D. Mar, R. Barve, M. Lukasik, A. F. Pirwani, E. Malette-Guyon, S. Lamba, B. J. Thomas, H. Sadeghi-Emamchaie, J. Liang, J.-F. Mallet, C. Matar and N. Ismail, *Brain Behav Immun* **2019**, *81*, 198-212.

64. N. R. Natale, M. Kent, N. Fox, D. Vavra and K. Lambert, *IBRO Neurosci Rep* **2021**, *11*, 207-215.

65. K. A. Partrick, A. M. Rosenhauer, J. Auger, A. R. Arnold, N. M. Ronczkowski, L. M. Jackson, M. N. Lord, S. M. Abdulla, B. Chassaing and K. L. Huhman, *Sci Rep* **2021**, *11*, 3763.

66. E. Patterson, P. M. Ryan, N. Wiley, I. Carafa, E. Sherwin, G. Moloney, E. Franciosi, R. Mandal, D. S. Wishart, K. Tuohy, R. P. Ross, J. F. Cryan, T. G. Dinan and C. Stanton, *Sci Rep* **2019**, *9*, 16323.

67. X. Qiu, G. Wu, L. Wang, Y. Tan and Z. Song, *Ann Transl Med* **2021**, *9*, 366.

68. J. B. Ramalho, M. B. Soares, C. C. Spiazzi, D. F. Bicca, V. M. Soares, J. G. Pereira, W. P. da Silva, C. P. Sehn and F. W. S. Cibin, *Nutrients* **2019**, *11*,

69. J. B. Ramalho, C. C. Spiazzi, D. F. Bicca, J. F. Rodrigues, C. P. Sehn, W. P. da Silva and F. W. S. Cibin, *Behav Brain Res* **2022**, *426*, 113847.

70. S. Sandes, N. Figueiredo, S. Pedroso, F. Sant'Anna, L. Acurcio, M. Abatemarco Junior, P. Barros, F. Oliveira, V. Cardoso, S. Generoso, M. Caliari, J. Nicoli, E. Neumann and Á. Nunes, *Food Res Int* **2020**, *137*, 109741.

71. H. M. Savignac, B. Kiely, T. G. Dinan and J. F. Cryan, *Neurogastroenterol Motil* **2014**, *26*, 1615-1627.

72. L. C. Silva, H. de Souza Lago, M. O. T. Rocha, V. S. de Oliveira, R. Laureano-Melo, E. T. G. Stutz, B. P. de Paula, J. F. P. Martins, R. H. Luchese, A. F. Guerra and P. Rodrigues, *Probiotics Antimicrob Proteins*

2021, *13*, 698-708.

73. H. Soltanmoradi, M. Maniati, A. Davoodabadi, A. Mosapour, S. Samavarchi Tehrani, M. Pazhoohan, F. Daemi and H. Khaleghzadeh-Ahangar, *Acta Alimentaria* **2021**, *50*, 393-403.

74. W. N. Sovijit, W. E. Sovijit, S. Pu, K. Usuda, R. Inoue, G. Watanabe, H. Yamaguchi and K. Nagaoka, *Neurosci Res* **2019**, *168*, 76-82.

75. L. K. Stenman, E. Patterson, J. Meunier, F. J. Roman and M. J. Lehtinen, *Behav Brain Res* **2020**, *379*, 112376.

76. J. Sun, F. Wang, X. Hu, C. Yang, H. Xu, Y. Yao and J. Liu, *J Agric Food Chem* **2018**, *66*, 8415-8421.

77. X. Sun, H.-F. Zhang, C.-L. Ma, H. Wei, B.-M. Li and J. Luo, *Can J Infect Dis Med Microbiol* **2021**, *2021*, 6613903.

78. Y. Sun, W. Geng, Y. Pan, J. Wang, P. Xiao and Y. Wang, *Food Funct* **2019**, *10*, 925-937.

79. K. Takahashi, O. Nakagawasai, W. Nemoto, T. Odaira, W. Sakuma, H. Onogi, H. Nishijima, R. Furihata, Y. Nemoto, H. Iwasa, K. Tan-No and T. Tadano, *J Neuroinflammation* **2019**, *16*, 201.

80. K. Takahashi, K. Kurokawa, L. Hong, K. Miyagawa, A. Mochida-Saito, M. Iwasa, H. Iwasa, O. Nakagawasai, T. Tadano, H. Takeda and M. Tsuji, *J Psychiatr Res* **2022**, *148*, 137-148.

81. P. Tian, R. Zou, L. Song, X. Zhang, B. Jiang, G. Wang, Y.-K. Lee, J. Zhao, H. Zhang and W. Chen, *Food Funct* **2019a**, *10*, 7588-7598.

82. P. Tian, G. Wang, J. Zhao, H. Zhang and W. Chen, *J Nutr Biochem* **2019b**, *66*, 43-51.

83. P. Tian, K. J. O'Riordan, Y.-K. Lee, G. Wang, J. Zhao, H. Zhang, J. F. Cryan and W. Chen, *Neurobiol Stress* **2020**, *12*, 100216.

84. P. Tian, H. Zhu, R. Zou, Q. Kong, M. Xu, J. Zhao, H. Zhang, W. Chen and G. Wang, *Food Funct* **2021a**, *12*, 646-655.

85. P. Tian, T. F. S. Bastiaanssen, L. Song, B. Jiang, X. Zhang, J. Zhao, H. Zhang, W. Chen, J. F. Cryan and G. Wang, *Mol Nutr Food Res* **2021b**, *65*, e2000704.

86. T. Tian, B. Xu, Y. Qin, L. Fan, J. Chen, P. Zheng, X. Gong, H. Wang, M. Bai, J. Pu, J. Lu, W. Zhou, L. Zhao, D. Yang and P. Xie, *Biochem Biophys Res Commun* **2019**, *516*, 430-436.

87. S. Tillmann and G. Wegener, *Behav Brain Res* **2019**, *359*, 755-762.

88. F. Trudeau, K. Gilbert, A. Tremblay, T. A. Tompkins, R. Godbout and G. Rousseau, *PLoS One* **2019**, *14*, e0215101.

89. P. Wang, K. Tu, P. Cao, Y. Yang, H. Zhang, X.-T. Qiu, M.-M. Zhang, X.-J. Wu, H. Yang and T. Chen, *Mol Brain* **2021**, *14*, 49.

90. S. Wang, T. Ishima, J. Zhang, Y. Qu, L. Chang, Y. Pu, Y. Fujita, Y. Tan, X. Wang and K. Hashimoto, *J Neuroinflammation* **2020a**, *17*, 241.

91. C.-L. Wei, S. Wang, J.-T. Yen, Y.-F. Cheng, C.-L. Liao, C.-C. Hsu, C.-C. Wu and Y.-C. Tsai, *Brain Res* **2019**, *1711*, 202-213.

92. R. Xie, P. Jiang, L. Lin, J. Jiang, B. Yu, J. Rao, H. Liu, W. Wei and Y. Qiao, *J Psychiatr Res* **2020b**, *122*, 70-78.

93. J. Xu, M. Tang, X. Wu, X. Kong, Y. Liu and X. Xu, *Food Funct* **2022**, *13*, 4331-4343.

94. M. Xu, P. Tian, H. Zhu, R. Zou, J. Zhao, H. Zhang, G. Wang and W. Chen, *Nutrients* **2022**, *14*,

95. Y. Yang, S. Zhao, X. Yang, W. Li, J. Si and X. Yang, *Neurosci Lett* **2022**, *774*, 136474.

96. S.-W. Yun, J.-K. Kim, K.-E. Lee, Y. J. Oh, H.-J. Choi, M. J. Han and D.-H. Kim, *Nutrients* **2020**, *12*,

97. S. W. Yun, J. K. Kim, M. J. Han and D. H. Kim, *Benef Microbes* **2021**, *12*, 541-551.

98. R. A. Yunes, E. U. Poluektova, E. V. Vasileva, M. V. Odorskaya, M. V. Marsova, G. I. Kovalev and V. N. Danilenko, *Probiotics Antimicrob Proteins* **2020**, *12*, 973-979.

99. Y. Zhao, G. Yang, Z. Zhao, C. Wang, C. Duan, L. Gao and S. Li, *Behav Brain Res* **2020**, *395*, 112853.

100. A. Burokas, S. Arboleya, R. D. Moloney, V. L. Peterson, K. Murphy, G. Clarke, C. Stanton, T. G. Dinan and J. F. Cryan, *Biol Psychiatry* **2017**, *82*, 472-487.

101. Y. Chen, M. Wan, Y. Zhong, T. Gao, Y. Zhang, F. Yan, D. Huang, Y. Wu and Z. Weng, *Mol Nutr Food Res* **2021a**, *65*, e2100146.

102. D. Cheng, H. Chang, S. Ma, J. Guo, G. She, F. Zhang, L. Li, X. Li and Y. Lu, *Molecules* **2018**, *23*,

103. L. Chi, I. Khan, Z. Lin, J. Zhang, M. Y. S. Lee, W. Leong, W. L. W. Hsiao and Y. Zheng, *Phytomedicine* **2020**, *67*, 153157.

104. D. J. Davis, P. M. Hecht, E. Jasarevic, D. Q. Beversdorf, M. J. Will, K. Fritsche and C. H. Gillespie, *Brain Behav Immun* **2017**, *59*, 38-48.

105. F. Donoso, S. Egerton, T. F. S. Bastiaanssen, P. Fitzgerald, S. Gite, F. Fouhy, R. P. Ross, C. Stanton, T. G. Dinan and J. F. Cryan, *Psychoneuroendocrinology* **2020**, *116*, 104673.

106. S. Egerton, F. Donoso, P. Fitzgerald, S. Gite, F. Fouhy, J. Whooley, T. G. Dinan, J. F. Cryan, S. C. Culloty, R. P. Ross and C. Stanton, *Nutr Neurosci* **2020**, *25*, 356-378.

107. L. Fan, Y. Peng, J. Wang, P. Ma, L. Zhao and X. Li, *Phytomedicine* **2021**, *83*, 153471.

108. X. Gao, Y. Feng, H. Xue, M. Meng and X. Qin, *Journal of Liquid Chromatography & Related Technologies* **2020**, *43*, 494-507.

109. M.-j. Gong, B. Han, S.-m. Wang, S.-w. Liang and Z.-j. Zou, *J Pharm Biomed Anal* **2016**, *123*, 63-73.

110. Y. Guo, J. Xie, X. Li, Y. Yuan, L. Zhang, W. Hu, H. Luo, H. Yu and R. Zhang, *Front Pharmacol* **2018**, *9*, 1126.

111. W. Z. Hao, Q. Y. Ma, G. Tao, J. Q. Huang and J. X. Chen, *Food Funct* **2021**, *12*, 12550-12564.

112. Y.-J. Huang, L.-X. C. Choong, S. Panyod, Y.-E. Lin, H.-S. Huang, K.-H. Lu, W.-K. Wu and L.-Y. Sheen, *Phytother Res* **2021**, *35*, 5133-5142.

113. W. D. Lai, T. H. Tung, C. Y. Teng, C. H. Chang, Y. C. Chen, H. Y. Huang, H. C. Lee and S. Y. Huang, *Food Funct* **2022**, *13*, 2662-2680.

114. N. C. Lax, S.-A. J. Parker, E. J. Hilton, Y. Seliman, K. J. Tidgewell and B. J. Kolber, *Synapse* **2018**, *72*, e22059.

115. H.-C. Lee, Y.-C. Lo, S.-C. Yu, T.-H. Tung, I. H. Lin and S.-Y. Huang, *Int J Food Sci Nutr* **2020**, *71*, 440-452.

116. Y. Li, Y. Peng, P. Ma, H. Yang, H. Xiong, M. Wang, C. Peng, P. Tu and X. Li, *Front Pharmacol* **2018**, *9*, 967.

117. S. Lin, Q. Li, S. Jiang, Z. Xu, Y. Jiang, L. Liu, J. Jiang, Y. Tong and P. Wang, *J Ethnopharmacol* **2021**, *268*, 113608.

118. Z. Liu, L. Li, S. Ma, J. Ye, H. Zhang, Y. Li, A. T. Sair, J. Pan, X. Liu, X. Li, S. Yan and X. Liu, *J Agric Food Chem* **2020**, *68*, 13697-13710.

119. A. Mika, H. E. W. Day, A. Martinez, N. L. Rumian, B. N. Greenwood, M. Chichlowski, B. M. Berg and M. Fleshner, *Eur J Neurosci* **2017**, *45*, 342-357.

120. S. M. O'Mahony, K.-A. McVey Neufeld, R. V. Waworuntu, M. M. Pusceddu, S. Manurung, K. Murphy, C. Strain, M. C. Laguna, V. L. Peterson, C. Stanton, B. M. Berg, T. G. Dinan and J. F. Cryan, *Eur J Neurosci* **2020**, *51*, 1042-1058.

121. M. M. Pusceddu, S. El Aidy, F. Crispie, O. O'Sullivan, P. Cotter, C. Stanton, P. Kelly, J. F. Cryan and T. G. Dinan, *PLoS One* **2015**, *10*, e0139721.

122. Y. Qu, K. Zhang, Y. Pu, L. Chang, S. Wang, Y. Tan, X. Wang, J. Zhang, T. Ohnishi, T. Yoshikawa and K. Hashimoto, *J Affect Disord* **2020**, *272*, 66-76.

123. R. C. Robertson, C. Seira Oriach, K. Murphy, G. M. Moloney, J. F. Cryan, T. G. Dinan, R. Paul Ross and C. Stanton, *Brain Behav Immun* **2017**, *59*, 21-37.

124. J. Song, N. Zhou, W. Ma, X. Gu, B. Chen, Y. Zeng, L. Yang and M. Zhou, *Food Funct* **2019b**, *10*, 2947-2957.

125. X. Song, W. Wang, S. Ding, X. Liu, Y. Wang and H. Ma, *J Affect Disord* **2021**, *290*, 353-363.

126. Y. Sun, T. Yan, G. Gong, Y. Li, J. Zhang, B. Wu, K. Bi and Y. Jia, *Int Immunopharmacol* **2020**, *89*, 107029.

127. P. Tian, H. Zhu, X. Qian, Y. Chen, Z. Wang, J. Zhao, H. Zhang, G. Wang and W. Chen, *Front Immunol* **2021**, *12*, 755481.

128. T.-H. Tung, Y.-T. Tung, I. H. Lin, C.-K. Shih, N. T. K. Nguyen, A. Shabrina and S.-Y. Huang, *Biomolecules* **2019**, *9*,

129. B. Valdes-Sustaita, E. Estrada-Camarena, M. E. Gonzalez-Trujano and C. Lopez-Rubalcava, *Neurochem Int* **2021**, *142*, 104904.

130. L. Wang, Y. Sun, T. Zhao, Y. Li, X. Zhao, L. Zhang, L. Wu, L. Zhang, T. Zhang, G. Wei, A. Altamirano, Y. Tong and Z. Yan, *Planta Med* **2020**, *86*, 172-179.

131. L. Wang, X. Wu, Y. Ma, X. Li, J. Zhang and L. Zhao, *Food Funct* **2021**, *12*, 4995-5006.

132. P. Wang, P. Guo, Y. Wang, X. Teng, H. Zhang, L. Sun, M. Xue and H. Liang, *Nutrients* **2022**, *14*,

133. Q. Wang, M. Jia, Y. Zhao, Y. Hui, J. Pan, H. Yu, S. Yan, X. Dai, X. Liu and Z. Liu, *J Agric Food Chem* **2019**, *67*, 12441-12451.

134. R. Wang, T. Chen, Q. Wang, X.-M. Yuan, Z.-L. Duan, Z.-Y. Feng, Y. Ding, F. Bu, G.-P. Shi and Y.-G. Chen, *Drug Des Devel Ther* **2021**, *15*, 2999-3016.

135. J. Xia, L. Gu, Y. Guo, H. Feng, S. Chen, J. Jurat, W. Fu and D. Zhang, *Front Cell Infect Microbiol* **2021**, *11*, 627608.

136. Q. Xiao, R. Shu, C. Wu, Y. Tong, Z. Xiong, J. Zhou, C. Yu, X. Xie and Z. Fu, *J Affect Disord* **2020**, *276*, 476-486.

137. M. Xue, X. Teng, H. Liang, J. Zhao, Y. Jiang, X. Qiu, Z. Zhang, Z. Pei, N. Zhang and Y. Qin, *Journal of Functional Foods* **2021**, *86*,

138. T. Yan, T. Nian, Z. Liao, F. Xiao, B. Wu, K. Bi, B. He and Y. Jia, *Int J Biol Macromol* **2020**, *144*, 427-440.

139. T. Yan, N. Wang, B. Liu, B. Wu, F. Xiao, B. He and Y. Jia, *Phytother Res* **2021**, *35*, 289-296.

140. J.-B. Yu, Z.-X. Zhao, R. Peng, L.-B. Pan, J. Fu, S.-R. Ma, P. Han, L. Cong, Z.-W. Zhang, L.-X. Sun, J.-D. Jiang and Y. Wang, *Front Pharmacol* **2019**, *10*, 268.

141. L. Zhang, L. Wang, L. Huang, Y. Zhao, H. Ding, B. Li, L. Wen, W. Xiong, Y. Liu, T. Zhang, L. Zhang, L. Wu, Q. Xu, Y. Fan, G. Wei, Q. Yin, Y. Chen, T. Zhang and Z. Yan, *Pharm Biol* **2021**, *59*, 912-921.

142. M. Zhang, A. Li, Q. Yang, J. Li, L. Wang, X. Liu, Y. Huang and L. Liu, *Front Cell Infect Microbiol* **2021**, *11*, 665159.

143. Z. Zhang, C. Yao, M. Li, L. C. Wang, W. Huang and Q. J. Chen, *Lett Appl Microbiol* **2022**,

144. B. Zhao, J. Wu, J. Li, Y. Bai, Y. Luo, B. Ji, B. Xia, Z. Liu, X. Tan, J. Lv and X. Liu, *J Agric Food Chem* **2020**, *68*, 3963-3975.

145. F. Zhao, S. Guan, Y. Fu, K. Wang, Z. Liu and T. B. Ng, *Biomed Pharmacother* **2021**, *143*, 112087.

146. Z. X. Zhao, J. Fu, S. R. Ma, R. Peng, J. B. Yu, L. Cong, L. B. Pan, Z. G. Zhang, H. Tian, C. T. Che, Y. Wang and J. D. Jiang, *Theranostics* **2018**, *8*, 5945-5959.

147. H. Li, P. Wang, L. Huang, P. Li and D. Zhang, *Neurogastroenterol Motil* **2019**, *31*, e13677.

148. K. Gilbert, J. Arseneault-Breard, F. Flores Monaco, A. Beaudoin, T. M. Bah, T. A. Tompkins, R. Godbout and G. Rousseau, *Br J Nutr* **2012**, *109*, 50-6.

149. X. Zhu, Y. Sun, C. Zhang and H. Liu, *Mol Med Rep* **2017**, *15*, 3161-3171.

150. S. Westfall, F. Caracci, M. Estill, T. Frolinger, L. Shen and G. M. Pasinetti, *Front Immunol* **2021**, *12*, 670500.

151. S. Westfall, F. Caracci, D. Zhao, Q.-L. Wu, T. Frolinger, J. Simon and G. M. Pasinetti, *Brain Behav Immun* **2021b**, *91*, 350-368.

152. A. Mesripour and P. Rakhshankhah, *Turk J Pharm Sci* **2021**, *18*, 21-27.

153. A. Leo, C. De Caro, P. Mainardi, M. Tallarico, V. Nesci, N. Marascio, P. Striano, E. Russo, A. Constanti, G. De Sarro and R. Citraro, *Neuropharmacology* **2021**, *198*, 108782.

154. R. Cheng, W. Xu, J. Wang, Z. Tang and M. Zhang, *Biochem Biophys Res Commun* **2021**, *566*, 170-176.

155. A. J. Kochanowska, K. V. Rao, S. Childress, A. El-Alfy, R. R. Matsumoto, M. Kelly, G. S. Stewart, K. J. Sufka and M. T. Hamann, *J Nat Prod* **2008**, *71*, 186-9.

156. J. Li, L. Hou, C. Wang, X. Jia, X. Qin and C. Wu, *Front Psychiatry* **2018**, *9*, 454.

157. Y. Matsuda, N. Ozawa, T. Shinozaki, K. I. Wakabayashi, K. Suzuki, Y. Kawano, I. Ohtsu and Y. Tatebayashi, *Transl Psychiatry* **2020**, *10*, 170.

158. M. Yu, H.-M. Jia, L.-L. Qin and Z.-M. Zou, *J Affect Disord* **2021**, *297*,

159. K. Suzuki, K. Nakamura, Y. Shimizu, Y. Yokoi, S. Ohira, M. Hagiwara, Y. Wang, Y. Song, T. Aizawa and T. Ayabe, *Sci Rep* **2021**, *11*, 9915.

160. D. Martín-Hernández, J. R. Caso, Á. G. Bris, S. R. Maus, J. L. M. Madrigal, B. García-Bueno, K. S. MacDowell, L. Alou, M. L. Gómez-Lus and J. C. Leza, *Neuropharmacology* **2016**, *103*, 122-133.

161. C. Meng, S. Feng, Z. Hao, C. Dong and H. Liu, *Psychoneuroendocrinology* **2022**, *136*, 105620.

162. A. K. Schmidtner, D. A. Slattery, J. Gläsner, A. Hiergeist, K. Gryksa, V. A. Malik, J. Hellmann-Regen, I. Heuser, T. C. Baghai, A. Gessner, R. Rupprecht, B. Di Benedetto and I. D. Neumann, *Transl Psychiatry* **2019**, *9*, 223.

163. S. Wang, Y. Qu, L. Chang, Y. Pu, K. Zhang and K. Hashimoto, *J Affect Disord* **2020b**, *260*, 448-457.

164. M. L. Wong, A. Inserra, M. D. Lewis, C. A. Mastronardi, L. Leong, J. Choo, S. Kentish, P. Xie, M. Morrison, S. L. Wesselingh, G. B. Rogers and J. Licinio, *Mol Psychiatry* **2016**, *21*, 797-805.

165. Q. Yang, L. Luo, T. Sun, L. Yang, L.-F. Cheng, Y. Wang, Q.-Q. Liu, A. Liu, H.-Y. Liu, M.-G. Zhao, S.-X. Wu and B. Feng, *Psychopharmacology (Berl)* **2020**, *237*, 3201-3213.

Table S13. A summary of probiotics that alleviate depression symptoms.

**Probiotics**	**objects**		**Probiotics**	**objects**
Akkermansia_muciniphila	Animal models		Lactobacillus_diacetylactis	Animal models
Akkermansia_muciniphila ATCC® BAA-835™	Animal models		Lactobacillus_fermentum	Human beings,Animal models
Bacillus_coagulans MTCC 5856	Human beings		Lactobacillus_gasseri NK109	Animal models
Bacillus_coagulans Unique IS2	Human beings		Lactobacillus_helveticus	Animal models
Bacillus_sp.DU-106	Animal models		Lactobacillus_helveticus 132M1	Animal models
Bacillus_subtilis	Animal models		Lactobacillus_helveticus LA 102	Animal models
Bifidobacteria	Animal models		Lactobacillus_helveticus LH0138	Animal models
Bifidobacterium	Human beings,Animal models		Lactobacillus_helveticus MCC1848	Animal models
Bifidobacterium spp.	Human beings		Lactobacillus_helveticus NS8	Animal models
Bifidobacterium_adolescentis	Human beings,Animal models		Lactobacillus_helveticus PXN® 45	Human beings
Bifidobacterium_adolescentis 150	Animal models		Lactobacillus_helveticus R0052	Human beings,Animal models
Bifidobacterium_adolescentis NK98	Human beings,Animal models		Lactobacillus_helveticus W74	Animal models
Bifidobacterium_animalis BL0005	Animal models		Lactobacillus_intestinalis	Animal models
Bifidobacterium_animalis ssp.lactis 420	Animal models		Lactobacillus_intestinalis YT2	Animal models
Bifidobacterium_bifidum	Human beings,Animal models		Lactobacillus_johnsonii	Animal models
Bifidobacterium_bifidum BIA-6	Human beings		Lactobacillus_kefiranofaciens ZW3	Animal models
Bifidobacterium_bifidum plus inulin	Human beings		Lactobacillus_lactis	Human beings,Animal models
Bifidobacterium_bifidum PXN® 23	Human beings		Lactobacillus_lactis ssp. lactis PXN® 63	Human beings
Bifidobacterium_bifidum W23	Human beings,Animal models		Lactobacillus_mucosae NK41	Animal models
Bifidobacterium_breve	Animal models		Lactobacillus_murinus	Animal models
Bifidobacterium_breve 1205	Animal models		Lactobacillus_paracasei 126L6	Animal models
Bifidobacterium_breve A-1 (synonym Bifidobacterium_breve MCC1274)	Human beings		Lactobacillus_paracasei DTA 81	Animal models
Bifidobacterium_breve CCFM1025	Human beings,Animal models		Lactobacillus_paracasei HT6	Animal models
Bifidobacterium_breve FHLJDQ3M5	Animal models		Lactobacillus_paracasei Lpc-37	Animal models
Bifidobacterium_breve M-16V	Animal models		Lactobacillus_paracasei NK112	Animal models
Bifidobacterium_breve M2CF22M7	Animal models		Lactobacillus_paracasei PS23	Animal models
Bifidobacterium_breve PXN® 25	Human beings		Lactobacillus_paracasei PS23 (PS23)	Animal models
Bifidobacterium_breve UBBr01	Human beings		Lactobacillus_plantarum	Animal models
Bifidobacterium_breve W25	Animal models		Lactobacillus_plantarum 15953 (strain CGMCC15953)	Animal models
Bifidobacterium_infantis	Animal models		Lactobacillus_plantarum 286 (Lp 286)	Animal models
Bifidobacterium_infantis 35624	Animal models		Lactobacillus_plantarum 81 (Lp 81)	Animal models
Bifidobacterium_infantis PXN® 27	Human beings		Lactobacillus_plantarum 8PA3	Animal models
Bifidobacterium_infantis UBBI01	Human beings		Lactobacillus_plantarum 90sk	Animal models
Bifidobacterium_lactis	Animal models		Lactobacillus_plantarum ATCC 793	Animal models
Bifidobacterium_lactis BAMA-B06/Bau-B0111	Human beings		Lactobacillus_plantarum DMDL 9010 (LP9010)	Animal models
Bifidobacterium_lactis BIA-7	Human beings		Lactobacillus_plantarum LP12151	Animal models
Bifidobacterium_lactis UBBLa70	Human beings		Lactobacillus_plantarum LP12407	Animal models
Bifidobacterium_lactis W51	Animal models		Lactobacillus_plantarum LP12418	Animal models
Bifidobacterium_lactis W52	Human beings,Animal models		Lactobacillus_plantarum LP3	Animal models
Bifidobacterium_longum	Animal models		Lactobacillus_plantarum MTCC 9510	Animal models
Bifidobacterium_longum NK46	Animal models		Lactobacillus_plantarum PS128	Animal models
Bifidobacterium_longum R0175	Human beings		Lactobacillus_plantarum PS128 (PS128)	Human beings
Bifidobacterium_longum 1714	Animal models		Lactobacillus_plantarum PXN® 47	Human beings
Bifidobacterium_longum ATCC 15707	Animal models		Lactobacillus_plantarum R1012	Animal models
Bifidobacterium_longum BG0014	Animal models		Lactobacillus_plantarum UBLP40	Human beings
Bifidobacterium_longum BIA-8	Human beings		Lactobacillus_plantarum WLPL04	Animal models
Bifidobacterium_longum LA 101	Animal models		Lactobacillus_plantarumWJL	Animal models
Bifidobacterium_longum NCC3001	Human beings		Lactobacillus_reuteri	Human beings,Animal models
Bifidobacterium_longum PXN® 30	Human beings		Lactobacillus_reuteri (DSM 17938)	Human beings
Bifidobacterium_longum R0175	Human beings,Animal models		Lactobacillus_reuteri 3	Animal models
Bifidobacterium_longum ssp. infantis Bl11471	Animal models		Lactobacillus_reuteri CCFM1132	Animal models
Bifidobacterium_longum subsp. Infantis	Animal models		Lactobacillus_reuteri NK33	Human beings,Animal models
Bifidobacterium_longum subsp. infantis E41	Animal models		Lactobacillus_rhamnosus	Human beings,Animal models
Bifidobacterium_longum subsp. Longum	Animal models		Lactobacillus_rhamnosus (JB-1)	Animal models

Bifidobacterium_longum subsp. Longum BAMA-B05/BauB1024	Human beings		Lactobacillus_rhamnosus B-8238	Animal models
Bifidobacterium_longum subsp.infantis CCFM687	Animal models		Lactobacillus_rhamnosus CCFM1131	Animal models
Bifidobacterium_longum W108	Animal models		Lactobacillus_rhamnosus CGMCC1.3724	Human beings
Bifidobacterium_pseudocatenulatum CECT 7765	Animal models		Lactobacillus_rhamnosus G	Human beings
Bifidobacterium_subtilis PXN® 21	Human beings		Lactobacillus_rhamnosus GG	Animal models
Clostridium_butyricum MIYAIRI 588 (CBM588)	Human beings,Animal models		Lactobacillus_rhamnosus HN001	Human beings
Clostridium_butyricum WZMC1018	Animal models		Lactobacillus_rhamnosus JB-1	Animal models
Enterococcus_faecalis 2001 (EF-2001)	Animal models		Lactobacillus_rhamnosus LR5	Animal models
Enterococcus_fecalis strain EC-12 (EC-12)	Animal models		Lactobacillus_rhamnosus LX11881	Animal models
Faecalibacterium_prausnitzii (ATCC 27766)	Animal models		Lactobacillus_rhamnosus PXN® 54	Human beings
Fermented Milk Containing Lactobacillus_paracasei Strain Shirota (LcS)	Human beings		Lactobacillus_rhamnosus R0011	Animal models
Komagataella_pastoris KM71H	Animal models		Lactobacillus_rhamnosus UBLR58	Human beings
Lactobacillus plantarum DP189	Animal models		Lactobacillus_rhamnosus W71	Animal models
Lactobacillus plantarum PS128TM	Human beings		Lactobacillus_rhamnosus zz-1	Animal models
Lactobacillus_acidophilus	Human beings,Animal models		Lactobacillus_salivarius	Animal models
Lactobacillus_acidophilus LA11873	Animal models		Lactobacillus_salivarius HA-118	Animal models
Lactobacillus_acidophilus PXN® 35	Human beings		Lactobacillus_salivarius Ls-33	Animal models
Lactobacillus_acidophilus T16	Human beings		Lactobacillus_salivarius PXN® 57	Human beings
Lactobacillus_acidophilus W37	Human beings,Animal models		Lactobacillus_salivarius W24	Human beings,Animal models
Lactobacillus_brevis	Animal models		Lactococcus_lactis	Animal models
Lactobacillus_brevis DPC6108	Animal models		Lactococcus_lactis LA 103	Animal models
Lactobacillus_brevis DSM32386	Animal models		Lactococcus_lactis strain WHH2078	Animal models
Lactobacillus_brevis FPA 3709	Animal models		Lactococcus_lactis subsp. cremoris LL95	Animal models
Lactobacillus_brevis J1	Animal models		Lactococcus_lactis W19	Human beings,Animal models
Lactobacillus_brevis W63	Human beings,Animal models		Lactococcus_lactis W58	Human beings,Animal models
Lactobacillus_bulgaricus	Animal models		Pediococcus_acidilactici	Animal models
Lactobacillus_casei	Human beings,Animal models		Prevotella_histicola DSM19854	Animal models
Lactobacillus_casei DG	Animal models		Probiotic NVP-1704	Human beings
Lactobacillus_casei PXN® 37	Human beings		Rhizopus_chinensis 12	Animal models
Lactobacillus_casei strain Shirota (LcS)	Human beings		Streptococcus_cerevisiae S-04	Animal models
Lactobacillus_casei W56	Human beings,Animal models		Streptococcus_cerevisiae var boulardii 17	Animal models
Lactobacillus_cremoris	Animal models		Streptococcus_thermophilus	Human beings,Animal models
Lactobacillus_delbrueckii	Animal models		Streptococcus_thermophilus LA 104	Animal models
Lactobacillus_delbrueckii ssp. bulgaricus PXN® 39	Human beings		Streptococcus_thermophilus PXN® 66	Human beings
Lactobacillus_delbrueckii subsp. Bulgaricus	Human beings,Animal models		Weissella_paramesenteroides WpK4	Animal models

Table S14. Search strategy for electronic databases.

Search strategy for PubMed		Hits
#1	(depress*[Title/Abstract] OR dysthymi*[Title/Abstract] OR mood disorder*[Title/Abstract] OR affective disorder*[Title/Abstract] OR antidepress*[Title/Abstract])	556,593
#2	(microb*[Title/Abstract] OR bacteria*[Title/Abstract] OR metaproteom*[Title/Abstract] OR metagenom*[Title/Abstract] OR "16S rRNA"[Title/Abstract] OR flora[Title/Abstract])	1,075,172
#3	#1 AND #2	5,634
Search strategy for Web of Science		
#1	TS=(depress* OR dysthymi* OR mood disorder* OR affective disorder* OR antidepress*)	502,875
#2	Topic=(microb* OR bacteria* OR metaproteom* OR metagenom* OR "16S rRNA" OR flora)	1,209,922
#3	#1 AND #2	6,594
Search strategy for Cochrane Library		
#1	(depress*):ti OR (dysthymi*):ti OR (mood disorder*):ti OR (affective disorder*):ti OR (antidepress*):ti	36,107
#2	(microb*):ti,ab,kw OR (bacteria*):ti,ab,kw OR (metaproteom*):ti,ab,kw OR (metagenom*):ti,ab,kw OR ("16S rRNA"):ti,ab,kw OR (flora):ti,ab,kw	56,167
#3	#1 AND #2	129
Search strategy for EMBASE-MEDLINE-PsycINFO		
#1	depress*:ab,ti OR dysthymi*:ab,ti OR “mood disorder*”:ab,ti OR “affective disorder*”:ab,ti OR antidepress*:ab,ti	1,063,823
#2	microb*:ab,ti OR bacteria*:ab,ti OR metaproteom*:ab,ti OR metagenom*:ab,ti OR "16S rRNA":ab,ti OR flora: ab,ti	1,300,339
#3	#1 AND #2	7,992

Table S15. Search strategy for microbiota-based interventions of depression from PubMed.

Search strategy for PubMed		Hits
#1	(depress*[Title/Abstract] OR dysthymi*[Title/Abstract] OR mood disorder*[Title/Abstract] OR affective disorder*[Title/Abstract] OR antidepress*[Title/Abstract])	474,410
#2	(microb*[Title/Abstract] OR flora[Title/Abstract] OR probiotic*[Title/Abstract] OR prebiotic*[Title/Abstract] OR synbiotic*[Title/Abstract] OR psychobiotic*[Title/Abstract] OR postbiotic*[Title/Abstract] OR "fecal microbiota transplantation"[Title/Abstract] OR "fecal transplantation"[Title/Abstract])	639,026
#3	#1 AND #2	3,633
